# Supplementary material for: Bayesian reanalysis of early remdesivir for the treatment of COVID-19 in outpatients with high risk of progression to severe disease
Source: PLoS One. 2026 Apr 16;21(4):e0346878. doi: 10.1371/journal.pone.0346878 (PMC13086435; doi:10.1371/journal.pone.0346878)
Supplement: S1 Table — (DOCX) [file pone.0346878.s001.docx]

**S1 Table. Characteristics of the randomized controlled trials selected for calculation of the data-driven mixture priors.**

| **NCT Number** | **Study Title** | N | **Outcome** | **Drug** | **DAA** | **Estimate/95% CI^a^** |
| --- | --- | --- | --- | --- | --- | --- |
| **NCT04510194** | COVID-OUT: Early Outpatient Treatment for SARS-CoV-2 Infection (COVID-19) | 1323 | Severe COVID-19 through 14 days: hypoxemia, ED, hospitalization, death | Metformin | No | OR = 0.47  (0.20 to 1.11) |
| **NCT04510195** |  | 808 |  | Ivermectin | No | OR = 0.73  (0.19 to 2.77) |
| **NCT04510196** |  | 661 |  | Fluvoxamine | No | OR = 1.11  (0.33 to 3.76) |
| **NCT05890586** | ACTIV-6: COVID-19 Study of Repurposed Medications – Arm B (Fluvoxamine) | 1208 | Hospitalization or death through Day 28 | Fluvoxamine | No | HR = 0.51  (0.05 to 5.64) |
| **NCT04322682** | Colchicine Coronavirus SARS-CoV2 Trial (COLCORONA) | 4488 | Composite of death or hospital admission for COVID-19 through Day 30 | Colchicine | No | OR = 0.79  (95.1% CI 0.61 to 1.03) |
| **NCT04518410** | ACTIV-2: A Study for Outpatients With COVID-19 | 837 | Hospitalization and death through Day 28 | BRII-196/  BRII-198 | Yes | RR = 0.22  (0.05 to 0.86) |
| **NCT04960202** | EPIC-HR: Study of Oral PF-07321332/Ritonavir Compared With Placebo in Nonhospitalized High Risk Adults With COVID-19 | 2246 | Covid-19–related hospitalization or death from any cause through day 28 | NIR/r | Yes | RR = 0.14  (0.07 to 0.28) |
| **NCT04545060** | VIR-7831 for the Early Treatment of COVID-19 in Outpatients | 583 | Hospitalization (for >24 hours) for any cause or death within 29 days | Sotrovimab | Yes | RR = 0.15  (97.24% CI 0.04 to 0.66) |
| **NCT04828161** | A Dose Finding, Efficacy and Safety Study of Ensovibep (MP0420) in Ambulatory Adult Patients With Symptomatic COVID-19 | 407 | COVID-19–related hospitalizations, emergency room (ER) visits, and/or all-cause mortality to day 29 | Ensovibep | Yes | RR = 0.22  (0.05 to 0.84) |

^a^ Unless otherwise indicated.

NCT = national clinical trial; N = total sample size of clinical trial; DAA = direct-acting antiviral; CI = confidence interval; SARS-CoV-2 = severe acute respiratory syndrome coronavirus 2; COVID-19 = coronavirus disease 19; OR = odds ratio; HR = hazard ratio; RR = risk ratio; NIR/r = nirmatrelvir/ritonavir
