# Supplementary material for: Bayesian reanalysis of early remdesivir for the treatment of COVID-19 in outpatients with high risk of progression to severe disease
Source: PLoS One. 2026 Apr 16;21(4):e0346878. doi: 10.1371/journal.pone.0346878 (PMC13086435; doi:10.1371/journal.pone.0346878)
Supplement: S1 Appendix — (PDF) [file pone.0346878.s002.pdf]

CLINICAL STUDY PROTOCOL

**Study Title:**	A Phase 3 Randomized, Double-Blind Placebo-Controlled Trial to Evaluate the Efficacy and Safety of Remdesivir (GS-5734™) Treatment of COVID-19 in an Outpatient Setting
**Sponsor:**	Gilead Sciences, Inc.<br>333 Lakeside Drive<br>Foster City, CA 94404
**IND Number:**	147753
**EudraCT Number:**	2020-003510-12
**Clinical Trials.gov Identifier:**	TBD
**Indication:**	COVID-19
**Protocol ID:**	GS-US-540-9012
**Contact Information:**	The medical monitor name and contact information will be provided on the Key Study Team Contact List.
**Protocol Version/Date:**	Original: 21 July 2020

This study will be conducted under United States Food and Drug Administration investigational new drug (IND) regulations (21 Code of Federal Regulations Part 312); however, sites located in the European Economic Area and Switzerland are not included under the IND and are considered non-IND sites.

CONFIDENTIALITY STATEMENT

The information contained in this document, particularly unpublished data, is the property or under control of Gilead Sciences, Inc., and is provided to you in confidence as an investigator, potential investigator, or consultant, for review by you, your staff, and an applicable institutional review board or independent ethics committee. The information is only to be used by you in connection with authorized clinical studies of the investigational drug described in the protocol. You will not disclose any of the information to others without written authorization from Gilead Sciences, Inc., except to the extent necessary to obtain informed consent from those persons to whom the drug may be administered.

TABLE OF CONTENTS

TABLE OF CONTENTS 2
LIST OF IN-TEXT TABLES 4
LIST OF IN-TEXT FIGURES 4
PROTOCOL SYNOPSIS 5
GLOSSARY OF ABBREVIATIONS AND DEFINITION OF TERMS 10
1. INTRODUCTION 12
1.1. Background 12
1.2. Remdesivir (RDV, GS-5734) 12
1.2.1. General Information 12
1.2.2. Additional Clinical Experience with RDV 13
1.3. Rationale for This Study 16
1.4. Rationale for Duration of Treatment 16
1.5. Rationale for Dose Selection of Remdesivir 17
1.6. Risk/Benefit Assessment for the Study 17
1.7. Compliance 18
2. OBJECTIVES 19
3. STUDY DESIGN 20
3.1. Endpoints 20
3.2. Study Design 21
3.3. Study Treatments 21
3.4. Duration of Treatment 21
3.5. Discontinuation Criteria 21
3.6. End of Study 22
3.7. Poststudy Care 22
3.8. Source Data 22
3.9. Biomarker Testing 22
3.9.1. Biomarker Samples for Optional Future Research 22
3.9.2. Biomarker Samples for Optional Genomic Research 23
4. PARTICIPANT POPULATION 24
4.1. Number of Participants and Participant Selection 24
4.1.1. Participant Replacement 24
4.2. Inclusion Criteria 24
4.3. Exclusion Criteria 25
5. INVESTIGATIONAL MEDICINAL PRODUCTS 26
5.1. Randomization, Blinding, and Treatment Codes Access 26
5.1.1. Randomization 26
5.1.2. Blinding 26
5.1.3. Procedures for Breaking Treatment Codes 26
5.2. Description and Handling of Remdesivir 27
5.2.1. Formulation 27
5.2.2. Packaging and Labeling 27
5.2.3. Storage and Handling 27
5.3. Dosage and Administration of Remdesivir 27
5.3.1. Infusion-related Reactions 28
5.4. Prior and Concomitant Medications 28

5.5. Accountability for Investigational Medicinal Product 28
5.5.1. Investigational Medicinal Product Return or Disposal 28
6. STUDY PROCEDURES 30
6.1. Participant Enrollment and Treatment Assignment 30
6.2. Pretreatment Assessments 30
6.2.1. Screening Visit 30
6.3. Treatment Assessments (Baseline/Day 1, Day 2 and Day 3) 31
6.4. Post-treatment Assessments (Day 4 through Day 14) 32
6.5. Day 28 Follow-up Assessment (± 5 Days) 32
6.6. Clinical Laboratory Assessments 33
6.6.1 Blood Samples 33
6.6.2 Pharmacokinetic Assessments 33
6.6.3 Biomarker Assessments (Optional) 34
6.6.4 Virology Assessments 34
6.7. Assessments for Early Discontinuation from Study 34
6.7.1 Criteria for Discontinuation of Study Treatment 34
6.8. End of Study 35
6.9. Poststudy Care 35
6.10. Sample Storage 35
7. ADVERSE EVENTS AND TOXICITY MANAGEMENT 36
7.1. Definitions of Adverse Events and Serious Adverse Events 36
7.1.1. Adverse Events 36
7.1.2. Serious Adverse Events 36
7.2. Assessment of Adverse Events and Serious Adverse Events 37
7.2.1. Assessment of Causality for Study Drugs and Procedures 37
7.2.2. Assessment of Severity 37
7.3. Investigator Requirements and Instructions for Reporting Adverse Events and Serious Adverse Events 38
7.3.2. Adverse Events 38
7.3.3. Serious Adverse Events 38
7.4. Gilead Reporting Requirements 39
7.5. Clinical Laboratory Abnormalities and Other Abnormal Assessments as Adverse Events or Serious Adverse Events 40
7.6. Toxicity Management 40
7.7. Special Situations Reports 40
7.7.1. Definitions of Special Situations 40
7.7.2. Instructions for Reporting Special Situations 41
8. STATISTICAL CONSIDERATIONS 43
8.1. Analysis Objectives and Endpoints 43
8.1.1. Analysis Objectives 43
8.1.2. Primary Endpoint 43
8.1.3. Secondary Endpoint 43
8.1.4. Other Endpoints of Interest 44
8.2. Planned Analyses 44
8.2.1. Interim Analysis 44
8.2.2. Final Analysis 45
8.3. Analysis Conventions 45
8.3.1. Analysis Sets 45
8.3.2. Data Handling Conventions 45
8.4. Demographic and Baseline Characteristics Analysis 46
8.5. Efficacy Analysis 46

8.5.1. Primary Analysis 46
8.5.2. Secondary Analyses 46
8.6. Safety Analysis......47
8.6.1. Extent of Exposure 47
8.6.2. Adverse Events......47
8.6.3. Laboratory Evaluations 47
8.7. Adjustments for Multiplicity......47
8.8. Pharmacokinetic Analysis......48
8.9. Sample Size......48
8.10. Independent Data Monitoring Committee......48

9. RESPONSIBILITIES......49

9.1. Investigator Responsibilities 49
9.1.1. Good Clinical Practice......49
9.1.2. Financial Disclosure 49
9.1.3. Institutional Review Board/Independent Ethics Committee Review and Approval......49
9.1.4. Informed Consent......49
9.1.5. Confidentiality......50
9.1.6. Study Files and Retention of Records 50
9.1.7. Case Report Forms 52
9.1.8. Investigator Inspections......52
9.1.9. Protocol Compliance 52
9.2. Sponsor Responsibilities 52
9.2.1. Protocol Modifications......52
9.2.2. Study Report and Publications 53
9.3. Joint Investigator/Sponsor Responsibilities 53
9.3.1. Payment Reporting......53
9.3.2. Access to Information for Monitoring......53
9.3.3. Access to Information for Auditing or Inspections 54
9.3.4. Study Discontinuation 54

10. REFERENCES 55

11. APPENDICES 56

Appendix 1. Investigator Signature Page......57
Appendix 2. Pandemic Risk Assessment and Mitigation Plan......58
Appendix 3. Study Procedures Table......61
Appendix 4. Pregnancy Precautions, Definition for Female of Childbearing Potential, and Contraceptive Requirements......63

LIST OF IN-TEXT TABLES

Table 1. Most Common Adverse Events Reported for Participants Overall in Study 5773......14
Table 2. Most Common Adverse Events Reported for Participants Overall in Study 5774......15

LIST OF IN-TEXT FIGURES

Figure 1. Study Schema......21

PROTOCOL SYNOPSIS

Gilead Sciences, Inc.
333 Lakeside Drive
Foster City, CA 94404

**Study Title:**	A Phase 3, Randomized Double-Blind Placebo-Controlled Trial to Evaluate the Efficacy and Safety of Remdesivir (GS-5734™) Treatment of COVID-19 in an Outpatient Setting
**IND Number:**<br>**EudraCT Number:**<br>**Clinical Trials.gov Identifier:**	147753<br>2020-003510-12<br>*TBD*
**Study Centers Planned:**	Approximately 100 centers globally
**Objectives:**	The purpose of this trial is to evaluate treatment with intravenous (IV) administered remdesivir (RDV, GS-5734) in an outpatient setting in participants with confirmed coronavirus disease 2019 (COVID-19) who are at risk for disease progression.<br>The primary objectives of this study are as follows:<br>• To evaluate the efficacy of RDV in reducing the rate of hospitalization or death in non-hospitalized patients with early stage COVID-19<br>• To evaluate the safety of RDV administered in an outpatient setting<br>The secondary objectives of this study are as follows:<br>• To determine the antiviral activity of RDV on severe acute respiratory syndrome (SARS)-coronavirus (CoV)-2 viral load<br>• To assess the impact of RDV on symptom duration and severity<br>The exploratory objectives of this study include:<br>• To assess the impact of RDV on other clinical outcomes<br>• To evaluate the emergence of viral resistance to RDV<br>• To identify and assess associations of host biomarkers with disease progression and treatment response<br>• To assess the pharmacokinetic(s) (PK) of RDV and its metabolites in patients with COVID-19

**Study Design:**	This is a Phase 3, randomized, double-blind, placebo controlled, multicenter study of RDV therapy for outpatients with early stage COVID-19 who are at higher risk of disease progression.<br>Participants who meet all eligibility criteria will be randomized in a 1:1 ratio to RDV or placebo. Randomization will be stratified by participants who reside in a skilled nursing facility, by participant's age (<60 vs ≥ 60 years), and by region (United States [US] vs. ex-US):<br>**Treatment Group A**: single dose of IV RDV 200 mg on Day 1 followed by IV RDV 100 mg on Days 2 and 3 (RDV group)<br>**Treatment Group B:** IV placebo to match (PTM) RDV on Days 1 to 3 (PTM group)
Number of Participants Planned:	Approximately 1230 participants
Target Population:	Non-hospitalized participants with early stage COVID-19 and at least one risk factor for disease progression. Up to 30% of enrolled participants may come from skilled nursing facilities.
Duration of Treatment:	Participants will receive study treatment with RDV or PTM for 3 days
Diagnosis and Main Eligibility Criteria:	Adult participants with COVID-19 who meet the following inclusion criteria may be included:<br>• Willing and able to provide written informed consent, or with a legal representative who can provide informed consent<br>• Either:<br>— At least 1 pre-existing risk factor for progression to hospitalization (chronic lung disease, hypertension, cardiovascular or cerebrovascular disease, diabetes, obesity (BMI ≥30), immunocompromised, chronic kidney disease, chronic liver disease, current cancer, or sickle cell disease);<br>— OR aged ≥ 60 years<br>• SARS-CoV-2 infection confirmed by polymerase chain reaction (PCR) ≤ 4 days prior to screening<br>• Presence of ≥ 1 symptom(s) consistent with COVID-19 for ≤ 7 days prior to randomization (such as fever, cough, fatigue, shortness of breath, sore throat, headache, myalgia/arthralgia)<br>• Oxygen saturation (SpO_2) > 94% on room air

	• Not currently requiring hospitalization<br>Exclusion criteria for participation include:<br>• Participation in any other clinical trial of an experimental treatment and prevention for COVID-19<br>• Prior hospitalization for COVID-19<br>• Treatment with other agents with actual or possible direct antiviral activity against SARS-CoV-2<br>• Use of hydroxychloroquine or chloroquine ≤ 7 days prior to screening<br>• Requiring oxygen supplementation<br>• Known hypersensitivity to the study drug, metabolites, or formulation excipient
Study Procedures/ Frequency:	At screening, after the participant has provided informed consent, demographic and baseline characteristics and medical history will be collected. Current clinical history, and concomitant medications will be documented. Physical examination findings, vital signs including temperature, respiratory rate, and SpO_2 on room air will be documented. Women of childbearing potential will have a urine pregnancy test.<br>After screening procedures, eligible participants will be randomized in a 1:1 ratio to receive treatment with RDV or PTM. Randomization will be stratified by residence in a skilled nursing facility, participant's age (<60 vs ≥ 60 years), and region (US vs ex-US).<br>The date of randomization will be considered Day 1 and all participants randomized to receive RDV or PTM should receive their initial dose on Day 1.<br>On study Day 1 through 3, vital signs including respiratory status will be measured, symptom assessment, and adverse events (AEs) and concomitant medications will be documented. Laboratory tests for safety (hematology and chemistry) will be performed on Days 1, 3, 7, and 14. Optional biomarkers will be performed on Days 1, 3 and 14. SARS-CoV-2 RT-qPCR testing will be performed on Day 1, 2, 3, 7 and 14.<br>On study Day 14 physical examination findings, vital signs including temperature, respiratory rate, and SpO_2, symptom assessment, AEs and concomitant medications will be documented.<br>Symptom severity will be assessed daily from Day 1 through Day 14 using the COVID-19 adapted InFLUenza Patient-Reported Outcome

	(FLU-PRO©) questionnaire (if available).<br>On study Day 28, there will be an in-person or phone visit. Physical examination, vital signs including temperature, respiratory rate, and SpO_2, symptom assessment, AEs and concomitant medications will be documented (only symptom assessment, AEs, and concomitant medications needed if performed by phone).<br>Sparse PK assessments will be conducted in all participants at participating sites and approximately 30 participants will have intensive PK assessments. At selected sites, sparse PK samples will be collected at study Day 2 (end of infusion, and optional 2-hour post end of infusion), and Day 3 (pre-dose [within 30 minutes of dosing] and end of infusion). Approximately 30 participants will have intensive PK samples collected at study Day 1 and Day 3 at the following time points relative to the start time of infusion: 0 (pre-dose), 0.5, 0.75, 3, 6, 8, 12 (optional) and 24 hours. All blood samples for PK assessments will be drawn from the arm opposite that the one used to administer RDV or PTM.
**Test Product, Dose, and Mode of Administration:**	Remdesivir for injection, 100 mg, for IV administration
**Reference Therapy, Dose, and Mode of Administration:**	Placebo-to-Match
**Criteria for Evaluation:**	
Safety:	• Proportion of participants with treatment-emergent AEs
Efficacy:	The primary endpoint of this study is:<br>• Composite endpoint of hospitalization or death from any cause by Day 14<br>The secondary efficacy endpoints of this study are:<br>• All-cause mortality at Day 28<br>• Rate of hospitalization by Day 28<br>• Time-weighted average change in SARS-CoV-2 viral load from baseline to Day 7<br>• Time to resolution of COVID-19-related symptoms<br>The exploratory endpoints of this study are:

- Time to symptom resolution of each domain of COVID-19 adapted FLU-PRO
- Change from baseline in COVID-19 adapted FLU-PRO total score and score in each domain
- Time-weighted average change in SARS-CoV-2 viral load from baseline to Day 14
- Time to first negative SARS-CoV-2 PCR
- Emergence of viral resistance to RDV
- Baseline levels and change from baseline for inflammation/immune-related, acute respiratory distress syndrome-related and coagulation-related biomarkers
- Proportion of participants progressing to outpatient oxygen requirement by Day 14
- Proportion of participants admitted to the intensive care unit by Day 28
- Proportion of participants started on mechanical ventilation by Day 28
- The plasma concentrations and PK parameters of RDV and metabolites

Statistical Methods: A sample size of approximately1230 participants (615 in each group 1:1 randomization) achieves > 90% power to detect a ratio of 0.65 (RDV to placebo hospitalization or death rate) using a 2-sided significance level of 0.05 assuming hospitalization or death rate is 20% in the placebo group. This analysis will be stratified by residence in skilled nursing facility, age (<60 vs ≥ 60 years), and region (US vs ex-US).

The primary endpoint will be analyzed using Kaplan-Meier estimates adjusted by stratification factors. The null hypothesis being tested is whether the ratio of hospitalization or death is the same for either RDV or placebo treatment. The ratio and 95% confidence interval will be provided.

Treatment-emergent AEs and laboratory abnormalities will be summarized using descriptive statistics and listed by participant.

This study will be conducted in accordance with the guidelines of Good Clinical Practice including archiving of essential documents.

GLOSSARY OF ABBREVIATIONS AND DEFINITION OF TERMS

ACTT	Adaptive COVID-19 Treatment Trial
AE	adverse event
ALT	alanine aminotransferase
ARDS	acute respiratory distress syndrome
AST	aspartate aminotransferase
CDC	Centers for Disease Control and Prevention
CI	confidence interval
CL_{cr}	creatinine clearance
CoV	coronavirus
COVID-19	coronavirus disease 2019
CRF	case report form
DAIDS	Division of AIDS
DMC	data monitoring committee
eCCGs	eCRF completion guidelines
eCRF	electronic case report form
EDC	electronic data capture
eSAE	electronic serious adverse event
EU	European Union
FDA	Food and Drug Administration
FLU-PRO©	InFLUenza Patient-Reported Outcome
GCP	Good Clinical Practice
Gilead	Gilead Sciences, Inc.
HIV	human immunodeficiency virus
IB	investigator’s brochure
ICH	International Council for Harmonisation (of Technical Requirements for Pharmaceuticals for Human Use)
IDMC	independent data monitoring committee
IEC	independent ethics committee
IND	investigational new drug
IRB	institutional review board
IUD	intrauterine device
IV	intravenous
IXRS	interactive voice/web response system
LLOQ	lower limit of quantitation
NIAID	National Institute of Allergy and Infectious Diseases
O_2	oxygen
PCR	polymerase chain reaction
PI	principal investigator
PK	pharmacokinetic(s)

PTM	placebo-to-match
PVE	Pharmacovigilance and Epidemiology
RDV	remdesivir (GS-5734™)
RNA	ribonucleic acid
SAE	serious adverse event
SARS	severe acute respiratory syndrome
SBECD	sulfobutylether β-cyclodextrin sodium
SDV	source data verification
SOC	standard of care
SOP	standard operating procedure
SpO_2	oxygen saturation
SUSAR	suspected unexpected serious adverse reaction
TEAE	treatment-emergent adverse event
ULN	upper limit of normal
US	United States

1. INTRODUCTION

1.1. Background

Severe acute respiratory syndrome (SARS)-coronavirus (CoV)-2, a single-stranded RNA virus, is identified as the cause of an outbreak of respiratory illness that was first detected in Wuhan, China in December 2019. The virus has now spread globally, resulting in a global pandemic and causing severe respiratory illness throughout the world. Severe cases progress to pneumonia and multi-organ failure, which can lead to death. Currently, there are no drugs approved for the treatment of coronavirus disease 2019 (COVID-19) in the United States (US). Gilead Sciences, Inc. (Gilead) has been working with global health authorities to respond to the ongoing pandemic and to evaluate the utility of intravenous (IV) RDV as a treatment option for COVID-19 through clinical trials. On 01 May 2020, the US Food and Drug Administration (FDA) issued an Emergency Use Authorization (EUA) {U. S. Food and Drug Administration (FDA) 2020} for IV RDV for the treatment of COVID-19 based on data from Adaptive COVID-19 Treatment Trial (ACTT-1), an ongoing, adaptive, randomized, double blind, placebo-controlled, multicenter study evaluating IV RDV versus placebo in hospitalized patients with COVID-19 (sponsored by National Institute of Allergy and Infectious Diseases [NIAID]; Gilead study number CO-US-540-5776) {Beigel 2020} and the randomized, open-label phase (Part A) of an ongoing Gilead-sponsored Phase 3, multicenter study evaluating 2 RDV regimens (5 days versus 10 days) in participants with severe COVID-19 (GS-US-540-5773). Intravenous RDV was subsequently approved for the treatment of COVID-19 in Japan and the European Union (EU).

1.2. Remdesivir (RDV, GS-5734)

Remdesivir (RDV; GS-5734™) is being developed by Gilead Sciences, Inc. (Gilead) and is formulated for intravenous (IV) administration.

1.2.1. General Information

Remdesivir is a nucleotide prodrug that is intracellularly metabolized into an analog of adenosine triphosphate that inhibits viral RNA polymerases and has broad spectrum activity against members of the filoviruses (eg, Ebola virus, Marburg virus, CoVs [eg, SARS-CoV-2, SARS-CoV, Middle East respiratory syndrome-CoV]), and paramyxoviruses (eg, respiratory syncytial virus , Nipah virus, and Hendra virus). For further information on RDV, refer to the current investigator's brochure (IB) for IV RDV. Information in the IB includes:

- Nonclinical pharmacokinetic(s) (PK) and in vitro metabolism
- Nonclinical pharmacology and toxicology
- Clinical experience

Additional relevant information regarding RDV are described below.

1.2.2. Additional Clinical Experience with RDV

1.2.2.1. NIAID ACTT-1 Study in Participants with Mild/Moderate and Severe COVID-19

A randomized, double-blind, placebo-controlled multicenter clinical trial evaluated RDV 200 mg once daily for 1 day followed by RDV 100 mg once daily for 9 days (for a total of up to 10 days of intravenously administered therapy) in hospitalized adult participants with COVID-19 with evidence of lower respiratory tract involvement. The trial enrolled 1062 hospitalized participants: 112 [10.5%] participants with mild/moderate disease and 950 [89.5%] participants with severe disease. A total of 282 participants (26.6%) (n = 129 received RDV) were on mechanical ventilation/ECMO. Participants were randomized in a 1:1 manner, stratified by disease severity at enrollment, to receive RDV (n = 541) or placebo (n = 521), plus standard of care (SOC).

At baseline, mean age was 59 years (with 36% of participants aged 65 or older); 64% of participants were male, 53% were White, 21% were Black, and 13% were Asian; 11% of participants had mild/moderate disease (12% in the RDV group vs 11% in placebo group) and 89% had severe disease (88% in the RDV group vs 89% in placebo group). The most common comorbidities were hypertension (51.0%), obesity (45.0%), and type 2 diabetes mellitus (31.0%)

The primary clinical endpoint was time to recovery within 28 days after randomization, defined as either discharged from the hospital or hospitalized but not requiring supplemental oxygen and no longer requiring ongoing medical care. The median time to recovery was 10 days in the RDV group compared to 14 days in the placebo group (recovery rate ratio 1.31; 95% CI 1.12 to 1.53, $p < 0.001$). Among participants with mild/moderate disease at enrollment (n = 112), the median time to recovery was 5 days in both the RDV and placebo groups (recovery rate ratio 1.16; [95% CI 0.77 to 1.72]). Among participants with severe disease at enrollment (n = 950), the median time to recovery was 11 days in the RDV group compared to 17 days in the placebo group (recovery rate ratio, 1.34; [95% CI, 1.13 to 1.58]; $p < 0.001$).

Overall, the odds of improvement in the ordinal scale were higher in the RDV group at Day 15 when compared to the placebo group (odds ratio, 1.46; [95% CI, 1.15 to 1.86], $p = 0.002$)).

1.2.2.2. Study GS-US-540-5773 (Part A) in Participants with Severe COVID-19

A randomized, open-label, multicenter clinical trial (Study GS-US-540-5773) of participants at least 12 years of age with confirmed SARS-CoV-2 infection, oxygen saturation of ≤ 94% on room air, and radiological evidence of pneumonia compared 200 participants who received RDV for 5 days with 197 participants who received RDV for 10 days. Participants on mechanical ventilation at screening were excluded. All participants received 200 mg of RDV on Day 1 and 100 mg once daily on subsequent days, plus SOC. The primary endpoint was clinical status on Day 14 assessed by a 7-point ordinal scale ranging from hospital discharge to increasing levels of oxygen and ventilatory support to death.

At baseline, the median age of participants was 61 years (range, 20 to 98 years); 64% were male, 75% were White, 12% were Black, and 12% were Asian. More participants in the 10-day group than the 5-day group required invasive mechanical ventilation or ECMO (5% vs 2%), or high-flow oxygen support (30% vs 25%), at baseline. Median duration of symptoms and hospitalization prior to first dose of RDV were similar across treatment groups.

Overall, after adjusting for between-group differences at baseline, participants receiving a 5-day course of RDV had similar clinical status at Day 14 as those receiving a 10-day course (odds ratio for improvement: 0.75; [95% CI 0.51 to 1.12]). In addition, recovery rates were 70% and 58%, and mortality rates were 8% and 11%, in the 5-day and 10-day groups, respectively. There were no significant differences once adjusted for between group differences at baseline. All-cause mortality at Day 28 was 12% vs 14% in the 5- and 10-day treatment groups, respectively. The most common adverse events are recorded in Table 1.

Table 1. Most Common Adverse Events Reported for Participants Overall in Study 5773

n (%)	5 Days N=200	10-Days N=197
Nausea	20 (10)	17 (9)
Acute respiratory failure	12 (6)	21 (11)
Acute kidney injury	4 (2)	16 (8)

1.2.2.3. Study GS-US-540-5774 (Part A) in Participants with Moderate COVID-19

A randomized, open-label multicenter clinical trial (Study GS-US-540-5774) of hospitalized participants at least 12 years of age with confirmed SARS-CoV-2 infection and radiological evidence of pneumonia without reduced oxygen levels compared treatment with RDV for 5 days (n = 191) and treatment with RDV for 10 days (n = 193) with SOC (n = 200). Participants treated with RDV received 200 mg on Day 1 and 100 mg once daily on subsequent days. The primary endpoint was clinical status on Day 11 assessed by a 7-point ordinal scale ranging from hospital discharge to increasing levels of oxygen and ventilatory support to death.

At baseline, the median age of participants was 57 years (range, 12 to 95 years); 61% were male, 61% were white, 19% were black, and 19% were Asian. Baseline clinical status, oxygen support status, and median duration of symptoms and hospitalization prior to first dose of RDV were similar across treatment groups.

Overall, the odds of improvement in the ordinal scale were higher in the 5-day RDV group at Day 11 when compared to those receiving only SOC (odds ratio, 1.65; [95% CI, 1.09 to 2.48], p = 0.017). The odds of improvement in clinical status with the 10-day treatment group when compared to those receiving only SOC were not statistically significant (odds ratio 1.31; [95% CI 0.88 to 1.95]; p = 0.18). At Day 11 observed mortality rates for the 5-day, 10-day, and standard of care groups were 0, 1%, and 2%, respectively. All-cause mortality at Day 28 was

2%, 1%, and 3% in the 5-day, 10-day, and SOC groups, respectively. The most common adverse events are recorded in Table 2.

Table 2. Most Common Adverse Events Reported for Participants Overall in Study 5774

n (%)	5 Days N=200	10 Days N=193	SOC N=200
Nausea	19 (10)	18 (9)	6 (3)

1.2.2.4. Integrated Safety Findings from Clinical Studies

Assessment of adverse reactions is based on data from four Phase 1 studies in 138 healthy adult participants, 4 Phase 3 studies in 1,467 hospitalized participants with COVID-19, and from 163 hospitalized participants with COVID-19 who received RDV in a compassionate use program.

The adverse reactions are listed below by system organ class and frequency. Frequencies are defined as follows: very common (≥ 10%), common (≥ 1% and <10%), uncommon (≥ 0.1% and < 1%), or rare (≥ 0.01% and < 0.1%).

INJURY, POISONING AND PROCEDURAL COMPLICATIONS

Rare: infusion-related reaction

HEPATOBILIARY DISORDERS

Very common: transaminases increased

Description of selected adverse reactions

Transaminases Increased

In healthy volunteer studies, the incidence of increased transaminases was higher in participants who received RDV compared to placebo. In clinical studies of participants with COVID-19, the incidence of increased transaminases was similar in participants treated with RDV compared to placebo or SOC.

Infusion-related Reactions

Infusion-related reactions have been observed following administration of RDV. Signs and symptoms may include hypotension, hypertension, tachycardia, bradycardia, hypoxia, fever, dyspnea, wheezing, angioedema, rash, nausea, diaphoresis, and shivering.

1.3. Rationale for This Study

Remdesivir is a novel antiviral drug that exhibits potent nanomolar antiviral activity against SARS-CoV-2 in primary human airway epithelial cells {Sheahan 2017}. In SARS-CoV-2-infected rhesus monkeys, administration of RDV resulted in a significant reduction in clinical signs of respiratory disease, lung pathology and gross lung lesions, and viral RNA levels compared with vehicle-treated animals {Williamson 2020}. The safety and efficacy of RDV has been demonstrated in clinical studies of patients hospitalized with COVID-19.

The risk of adverse outcomes from COVID-19 increases with age and the presence of chronic health conditions, such as cardiovascular disease, diabetes, and chronic lung disease{Stokes 2020, The Novel Coronavirus Pneumonia Emergency Response Epidemiology Team 2020, World Health Organization (WHO) 2020a}. In acute viral infection with influenza, a short course of antiviral treatment has shown improved clinical outcome {Nicholson 2000}. Treatment intervention in the earlier phase of COVID-19, such as in an outpatient setting, may prevent disease progression to moderate disease requiring hospitalization. The resulting reduction in hospitalizations would significantly benefit patients and would reduce the burden to healthcare systems.

There is currently no approved treatment for patients with COVID-19 who have not been hospitalized. This study will evaluate the potential for a 3-day treatment with IV RDV to change the clinical trajectory of early COVID-19. The double blinded design will allow stringent evaluation of the safety and efficacy of RDV in this population.

Participants in this study will be treated with RDV for a maximum of 3 days receiving a total of 400 mg RDV. This dose corresponds to a total administration of 12 g of sulfobutylether β-cyclodextrin sodium (SBECD) using the lyophilized powder. Based on the European Medicines Agency (EMA) review summarizing the safety of cyclodextrins as excipients {Committee for Medicinal Products for Human Use (CHMP) 2014}, which indicates approximately 250 mg/kg/day of SBECD (12 g/day based on a 48 kg human) is safe, participants weighing more than 48 kg may be enrolled regardless of prior or current renal impairment.

1.4. Rationale for Duration of Treatment

In patients with severe COVID-19 who do not require mechanical ventilation, 5 days of RDV showed similar efficacy to a 10 day regimen. Similarly, 5 days treatment of RDV in participants with moderate COVID-19 was associated with a significant improvement in clinical status compared with SOC and approximately a third of participants were discharged prior to completion of 5 days RDV therapy.

In early viral infection, shorter courses of antivirals are often effective in preventing disease progression {Nicholson 2000}. As such, a shorter duration of 3 days of RDV treatment is proposed in participants with early stage COVID-19 not requiring hospitalization or oxygen supplementation with the goal of preventing disease progression.

1.5. Rationale for Dose Selection of Remdesivir

The dosing of RDV in this study, 200 mg on Day 1 and 100 mg on each of Days 2 and 3 is the initial dosing recommended by US FDA and approved by EMA.

1.6. Risk/Benefit Assessment for the Study

In addition to the established risks associated with IV RDV, potential risks associated with the study include unknown adverse events (AEs) and laboratory abnormalities. There is the potential for resistance to develop with shorter treatment duration.

Remdesivir for up to 10 days is available in the US following the US FDA issuing an EUA {U. S. Food and Drug Administration (FDA) 2020} for IV RDV for the treatment of COVID-19. Intravenous RDV for up to 10 days is approved for the treatment of COVID-19 in Japan and the EU.

The shorter duration of treatment may lower the risk of some adverse findings associated with RDV, e.g., the transient elevations of alanine aminotransferase (ALT) and aspartate aminotransferase (AST). However, other risks may not be ameliorated or may not have been evident in the studies of participants with more severe disease.

An independent data monitoring committee (IDMC) will review safety and efficacy data of the study when 50% of the participants have been enrolled. In addition, a futility interim analysis is planned when approximately 50% of the participants have completed the Day 14 visit to stop a non-efficacious treatment. The IDMC will make a recommendation of stopping enrollment to the study if the pre-specified futility stopping criteria are met.

The risk mitigation strategy for this study includes restriction of the study population to those without a history of significant hepatic disease:

- Exclusion of participants with contraindicated known hepatic or renal disease
- Exclusion of coadministration of other investigational agents against COVID-19
- Serum chemistry assessments, including liver function tests, will be monitored during the study period.

There are currently no investigational agents with demonstrated clinical efficacy or approved treatments for COVID-19 patients not requiring in-patient care. The timely evaluation of a safe and effective antiviral agent with demonstrated safety and efficacy addresses a serious unmet medical need. In consideration of the information included in this protocol, the overall risks to participants are outweighed by the potential benefits of RDV investigational therapy for the treatment of COVID-19. The benefit-risk balance for this study is considered positive.

During a pandemic, additional potential risks to subjects may include adequate study drug availability, interruptions to the study visit schedule, and adherence to protocol-specified safety monitoring or laboratory assessments. Refer to Appendix 2 for further details on the risks and risk mitigation strategy.

1.7. Compliance

This study will be conducted in compliance with this protocol, Good Clinical Practice (GCP), and all applicable regulatory requirements.

2. OBJECTIVES

The purpose of this trial is to evaluate treatment with IV administered RDV in an outpatient setting in participants with confirmed COVID-19 who are at risk for disease progression.

The primary objectives of this study are as follows:

- To evaluate the efficacy of RDV in reducing the rate of hospitalization or death in non-hospitalized participants with early stage COVID-19
- To evaluate the safety of RDV administered in an outpatient setting

The secondary objectives of this study are as follows:

- To determine the antiviral activity of RDV on SARS-CoV-2 viral load
- To assess the impact of RDV on symptom duration and severity

The exploratory objectives of this study include:

- To assess the impact of RDV on other clinical outcomes
- To evaluate the emergence of viral resistance to RDV
- To identify and assess associations of host biomarkers with disease progression and treatment response
- To assess the PK of RDV and its metabolites in participants with COVID-19

3. STUDY DESIGN

3.1. Endpoints

The primary endpoint of this study is as follows:

- Composite endpoint of hospitalization or death from any cause by Day 14
- The proportion of participants with treatment emergent adverse events

The secondary endpoints of this study are as follows:

- All-cause mortality at Day 28
- Rate of hospitalization by Day 28
- Time-weighted average change in SARS-CoV-2 viral load from baseline to Day 7
- Time to resolution of COVID-19-related symptoms

The exploratory endpoints of the study are as follows:

- Time to symptom resolution of each domain of COVID-19 adapted InFLUenza Patient-Reported Outcome (FLU-PRO©)
- Change from baseline in COVID-19-adapted FLU-PRO total score and score in each domain
- Time-weighted average change in SARS-CoV-2 viral load from baseline to Day 14
- Time to first negative SARS-CoV-2 polymerase chain reaction (PCR)
- Emergence of viral resistance to RDV
- Baseline levels and change from baseline for inflammation/immune-related, acute respiratory distress syndrome (ARDS)-related and coagulation-related biomarkers
- Proportion of participants progressing to outpatient oxygen (O_2) requirement by Day 14
- Proportion of participants admitted to the intensive care unit by Day 28
- Proportion of participants started on mechanical ventilation by Day 28
- The plasma concentrations and PK parameters of RDV and metabolites

3.2. Study Design

This is a Phase 3, randomized, double-blind, placebo controlled, multicenter study of RDV therapy for outpatients with early stage COVID-19 who are at increased risk of disease progression.

3.3. Study Treatments

Approximately 1230 participants who meet all eligibility criteria may be randomized in a 1:1 ratio into either treatment group:

Treatment Group A: single dose of IV RDV 200 mg on Day 1 followed by IV RDV 100 mg on Days 2 and 3 (RDV group)

Treatment Group B: IV placebo-to-match (PTM) RDV on Days 1 to 3 (PTM group)

Randomization will be stratified by participants who reside in a skilled nursing facility, by participant’s age (<60 vs ≥ 60 years), and by region (US vs. ex-US). Up to 30% of enrolled participants may come from skilled nursing facilities.

3.4. Duration of Treatment

Participants will receive study treatment with RDV for 3 days (Treatment Group A) or PTM for 3 days (Treatment Group B).

Figure 1. Study Schema

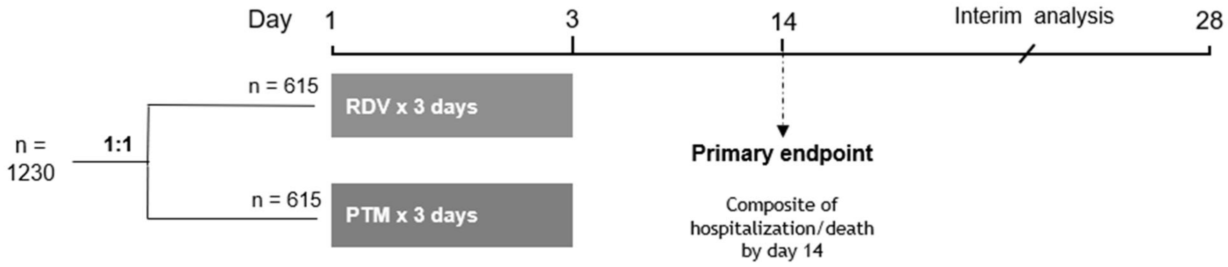

3.5. Discontinuation Criteria

Study drug dosing in an individual participant will be placed on hold and may be discontinued, following a review of all available clinical data by the medical monitor and discussion with the investigator, if any of the following occurs:

- Any serious AE (SAE) or ≥ Grade 3 AE suspected to be related to RDV
- Any elevations in ALT or AST ≥ 5 × upper limit of normal (ULN); or ALT > 3 × ULN and total bilirubin > 2 × ULN, confirmed by immediate repeat testing

Discontinuation of study medication is not a seriousness criterion.

Participants who discontinue the study medication should be encouraged to remain in the study and attend study visits as described in Sections 6.3, 6.4, and 6.5.

3.6. End of Study

The end of the study will be the last participant's last observation (or visit).

3.7. Poststudy Care

The long-term care of the participant will remain the responsibility of their primary treating physician. Remdesivir is being supplied with curative intent. There is no provision for post-study availability.

3.8. Source Data

The source data for this study will be obtained from electronic data capture (EDC), central laboratory, local laboratory, specialty laboratory (for PK and/or pharmacodynamic data), and/or interactive voice/web response system (IXRS) data.

3.9. Biomarker Testing

3.9.1. Biomarker Samples for Optional Future Research

In addition to the study-specific informed consent, participants will be required to document whether they agree to have additional samples collected and for the remainder of their already collected PK specimens for optional future research to be used, in accordance with applicable regulations.

The collection of additional samples and use of the remainder of their already collected specimens for optional future research may be used to advance development of the drug and/or increase our knowledge and understanding of the biology of the disease under investigation and related diseases. These specimens may also be used to study the association of biomarkers with biological pathways, disease pathogenesis, progression and/or treatment outcomes, including efficacy, adverse events, and the processes of drug absorption and disposition. In addition, these specimens may be used to develop biomarker, and/or diagnostic assays and establish the performance characteristics of these assays. The collection and analysis of optional future research specimens may facilitate the design of new pharmaceutical agents and the development of diagnostic tests, which may allow for individualized drug therapy for patients in the future.

Serum and plasma samples will be collected to measure biomarkers which may include but not limited to biomarkers of inflammation, ARDS, and coagulopathy in COVID 19 at Days 1, 3, and 14.

The specimens collected for optional future research will be destroyed no later than 15 years after the end of study or per country requirements.

3.9.2. Biomarker Samples for Optional Genomic Research

In addition to the study-specific informed consent to be signed by each participant in the study, participants will be required to document whether they agree to provide additional samples for optional genomic research. Additional samples will be obtained from participants who agree to participate and provide their additional specific consent. These samples should be collected pre-dose at the Baseline/Day 1 visit, but may be collected at any time during the study or at a separate post study visit, if necessary.

The specimens collected for optional future genomic research may be used to advance the development of the drug and/or increase our knowledge and understanding of the biology of the disease under investigation, or related diseases. These specimens may also be used to study the association of biomarkers with biological pathways, disease pathogenesis, progression and/or treatment outcomes, including efficacy, AEs, and the processes of drug absorption and disposition. In addition, these specimens may be used to develop biomarker and/or diagnostic assays and establish the performance characteristics of these assays. The collection and analysis of optional genomic research specimens may facilitate the design of new pharmaceutical agents and the development of diagnostic tests, which may allow for individualized drug therapy for patients in the future.

The samples collected for optional genomic research will be destroyed no later than 15 years after the end of the study or per country requirements.

4. PARTICIPANT POPULATION

4.1. Number of Participants and Participant Selection

Approximately 1230 participants who meet the eligibility criteria will be enrolled in one of 2 groups. Up to 30% of participants may be residing in skilled nursing facilities. Other participants will receive study drug in an outpatient or home health setting.

4.1.1. Participant Replacement

Participants who discontinue before the end of study will not be replaced.

4.2. Inclusion Criteria

Participants must meet all of the following inclusion criteria to be eligible for participation in this study:

1) Willing and able to provide written informed consent, or with a legal representative who can provide informed consent

2) Either:

   — Age ≥ 18 years with at least 1 of the following pre-existing risk factors for progression to hospitalization:

   a) Chronic lung disease: chronic obstructive pulmonary disease, moderate-to-severe asthma, cystic fibrosis, pulmonary fibrosis

   b) Hypertension: systemic or pulmonary

   c) Cardiovascular or cerebrovascular disease: coronary artery disease, congenital heart disease, heart failure, cardiomyopathy, history of stroke

   d) Diabetes mellitus: Type 1 or 2

   e) Obesity (BMI ≥ 30)

   f) Immunocompromised state

   g) Chronic kidney disease: any stage

   h) Chronic liver disease

   i) Current cancer

   j) Sickle cell disease

   — OR aged ≥ 60 years, regardless of the presence of other pre-existing risk factors for progression

3) SARS-CoV-2 infection confirmed by PCR ≤ 4 days prior to screening

4) Presence of ≥ 1 symptom(s) consistent with COVID-19 for ≤ 7 days prior to randomization (such as fever, cough, fatigue, shortness of breath, sore throat, headache, myalgia/arthralgia) {Stokes 2020, World Health Organization (WHO) 2020b}

5) Oxygen saturation (SpO_2) > 94% on room air

6) Not currently requiring hospitalization

7) Participants of childbearing potential who engage in heterosexual intercourse must agree to use protocol-specified method(s) of contraception as described in Appendix 4

4.3. Exclusion Criteria

Participants who meet *any* of the following exclusion criteria are not eligible to be enrolled in this study:

1) Participation in any other clinical trial of an experimental treatment and prevention for COVID-19

2) Prior hospitalization for COVID-19

3) Treatment with other agents with actual or possible direct antiviral activity against SARS-CoV-2

4) Use of hydroxychloroquine or chloroquine ≤ 7 days prior to screening

5) Requiring oxygen supplementation

6) ALT or AST ≥ 5 × ULN at screening or within 90 days of screening

7) Creatinine clearance < 30 mL/min at screening or < 90 days before screening ONLY if the participant's weight is < 48 kg

8) Breastfeeding female

9) Known hypersensitivity to the study drug, the metabolites, or formulation excipient

10) Use or planned use of exclusionary medications, refer to Section 5.4

5. INVESTIGATIONAL MEDICINAL PRODUCTS

5.1. Randomization, Blinding, and Treatment Codes Access

5.1.1. Randomization

Participants who meet all randomization eligibility criteria will be randomized in a 1:1 ratio to Treatment Group A or Treatment Group B starting on Day 1 and assigned a participant number. Randomization will be stratified by residence in a skilled nursing facility, age (<60 vs ≥ 60), and region (US vs ex-US).

5.1.2. Blinding

During the randomized phase participants and all personnel directly involved in the conduct of the study will be blinded to treatment assignment. Specified personnel may be unblinded based on their study role. Study drug will be dispensed by the study pharmacist, or designee, in a blinded fashion to the participants. The Pharmacokinetics File Administrator, or designee, in Bioanalytical Operations and/or Clinical Data Management, who facilitates the data transfer of PK files between Gilead and vendors, will remain unblinded. Individuals in Clinical Packaging and Labeling or Clinical Supply Management who have an Unblinded Inventory Manager role in the IXRS system for purposes of study drug inventory management will remain unblinded. Individuals in Pharmacovigilance and Epidemiology (PVE) responsible for safety signal detection, investigational new drug (IND) safety reporting, and/or expedited reporting of suspected unexpected serious adverse reactions (SUSARs) may be unblinded to individual case data and/or group level summaries. External (ie, contract research organizations) biostatisticians and programmers will be unblinded for the IDMC, IND safety reporting. Regulatory Quality and Compliance personnel in Research and Development may also be unblinded for purposes of supporting Quality Assurance activities and/or Regulatory Agency inspections.

5.1.3. Procedures for Breaking Treatment Codes

In the event of a medical emergency where breaking the blind is required to provide medical care to the participant, the investigator may obtain treatment assignment directly from the IXRS system for that participant. Gilead recommends but does not require that the investigator contact the Gilead medical monitor before breaking the blind. Treatment assignment should remain blinded unless that knowledge is necessary to determine participant emergency medical care. The rationale for unblinding must be clearly explained in source documentation and on the electronic case report form (eCRF), along with the date on which the treatment assignment was obtained. The investigator is requested to contact the Gilead medical monitor promptly in case of any treatment unblinding.

Blinding of study treatment is critical to the integrity of this clinical study. Therefore, if a participant's treatment assignment is disclosed to the investigator, the participant will have study treatment discontinued. All participants will be followed until study completion unless consent to do so is specifically withdrawn by the participant.

5.2. Description and Handling of Remdesivir

5.2.1. Formulation

Remdesivir for injection, 100 mg, is a preservative-free, white to off-white to yellow, lyophilized solid containing 100 mg of RDV that is to be reconstituted with sterile water for injection and diluted into 0.9% saline prior to administration by IV infusion.

In addition to the active ingredient, RDV for injection, 100 mg, contains the following inactive ingredients: SBECD, water for injection, hydrochloric acid, and sodium hydroxide. Hydrochloric acid and sodium hydroxide are used to adjust the formulation to a pH of 3.0 to 4.0.

The supplied PTM RDV for injection, 100 mg, is identical in physical appearance to the active formulation and contains the same inactive ingredients.

5.2.2. Packaging and Labeling

Remdesivir for injection and PTM, 100 mg, is supplied as a sterile product in a single-use, 30-mL Type I clear glass vial. Each vial is sealed with a rubber stopper and an aluminum over seal with a red, plastic flip-off cap. Following reconstitution, each single-use vial contains sufficient volume to allow withdrawal of 20 mL (100 mg remdesivir or PTM).

Remdesivir for injection and PTM, 100 mg, shall be labeled to meet all applicable requirements of the USFDA, EU Guideline to Good Manufacturing Practice – Annex 13 (Investigational Medicinal Products), the J-GCP (Ministerial Ordinance on Good Clinical Practice for Drugs), as applicable, and/or other local regulations.

5.2.3. Storage and Handling

Remdesivir for injection and PTM, 100 mg, vials should be stored below 30 °C (86 °F) prior to use. Storage conditions are specified on the label. Until dispensed for dosing, all vials of study drugs should be stored in a securely locked area, accessible only to authorized site personnel.

To ensure the sterility, stability, and proper identification, study drug(s) should not be stored in a container other than the container in which they were supplied.

Measures that minimize drug contact with the body should always be considered during handling, preparation, and disposal procedures.

The total storage time of reconstituted solution containing RDV or placebo should not exceed 4 hours at room temperature (20 °C to 25 °C) or 24 hours at refrigerated temperature (2 °C to 8 °C). Any unused reconstituted solution containing RDV or placebo should be discarded.

5.3. Dosage and Administration of Remdesivir

Remdesivir for injection, 100 mg, or PTM will be provided by Gilead.

Participants in Treatment Group A will receive IV RDV 200 mg on Day 1 followed by IV RDV 100 mg on Days 2 and 3. Participants in the Treatment Group B will receive IV PTM on Days 1 to 3.

5.3.1. Infusion-related Reactions

Infusion-related reactions have been observed during and following administration of RDV. Signs and symptoms may include hypotension, hypertension, tachycardia, bradycardia, hypoxia, fever, dyspnea, wheezing, angioedema, rash, nausea, diaphoresis, and shivering. Slower infusion rates, with a maximum infusion time of up to 120 minutes, can be considered to potentially prevent these signs and symptoms. If signs and symptoms of a severe infusion-related reaction occur, immediately discontinue administration of RDV and initiate appropriate treatment. Please refer to Section 7.6.

5.4. Prior and Concomitant Medications

Concomitant use of the following is prohibited in participants receiving RDV:

- Investigational agents for COVID-19 including approved HIV protease inhibitors such as lopinavir/RTV, chloroquine, interferon, etc.
- Use of hydroxychloroquine or chloroquine within 7 days of randomization
- Strong inducers of P-glycoprotein (e.g., rifampin or herbal medications)

Medications will be assessed from screening to the Day 28 Follow-up visit.

5.5. Accountability for Investigational Medicinal Product

The investigator is responsible for ensuring adequate accountability of all used and unused study drug vials. This includes acknowledgment of receipt of each shipment of study drug (quantity and condition).

Each study site must keep accountability records that capture:

- The date received and quantity of study drug vials.
- The date, participant number, and the study drug vial number dispensed.
- The date, quantity of used and unused study drug vials returned, along with the initials of the person recording the information.

5.5.1. Investigational Medicinal Product Return or Disposal

Gilead recommends that used and unused study drug supplies be destroyed at the site. If the site has an appropriate standard operating procedure (SOP) for drug destruction as determined by Gilead, the site may destroy used (empty or partially empty) and unused study drug supplies in

accordance with that site's approved SOP. A copy of the site's approved SOP will be obtained for eTMF. If study drug is destroyed on site, the investigator must maintain accurate records for all study drugs destroyed. Records must show the identification and quantity of each unit destroyed, the method of destruction, and the person who disposed of the study drug. Upon study completion, copies of the study drug accountability records must be filed at the site. Another copy will be returned to Gilead.

If the site does not have an appropriate SOP for drug destruction, used and unused study drug supplies are to be sent to the designated disposal facility for destruction. The study monitor will provide instructions for return.

The study monitor will review study drug supplies and associated records at periodic intervals.

For both disposal options listed above, the study monitor must first perform drug accountability during an on-site or remote monitoring visit.

6. STUDY PROCEDURES

The study procedures to be conducted for each participant enrolled in the study are presented in tabular form in Appendix 3 and described in the text that follows.

The investigator must document any deviation from the protocol procedures and notify Gilead or the contract research organization.

6.1. Participant Enrollment and Treatment Assignment

Entry into screening does not guarantee enrollment into the study. In order to manage the total study enrollment, Gilead, at its sole discretion, may suspend screening and/or enrollment at any site or study-wide at any time. Study visits may be performed in an outpatient setting, at the participant's home via tele-health, virtually or remotely, as permitted by local and institutional regulations.

6.2. Pretreatment Assessments

6.2.1. Screening Visit

Participants will be screened within 2 days before randomization to determine eligibility for participation in the study. The following will be performed and documented at screening:

- Obtain written informed consent
- Obtain medical history including the following information: date of first symptoms, overall symptoms, exposure source, demographics, baseline characteristics, allergies and all other medical history
- Review and document prior and concomitant medications
- Complete physical examination (urogenital/anorectal exam not required) including, vital signs (heart rate, temperature, blood pressure), body weight, and height.
- Respiratory status:
  - Respiratory rate
  - Oxygenation: SpO_2 on room air
- Obtain blood samples for ALT, AST, serum creatinine, and creatinine clearance if prior history of renal or liver disease (if no history of severe renal or severe renal disease but with weight ≥ 48 kg or liver disease or if results available within the prior 3 months, can defer until Day 1)

- Women of childbearing potential will have a urine pregnancy test performed
- Documentation of SARS-CoV-2 infection confirmed by PCR ≤ 4 days prior to screening
- Confirmation of presence of COVID-19 symptoms
- Record any SAEs and all AEs related to protocol-mandated procedures occurring after signing of the consent form

Participants meeting all of the inclusion criteria and none of the exclusion criteria may be enrolled the same day as screening for randomization into the study.

From the time of obtaining informed consent through the first administration of study drug, record all SAEs, as well as any AEs related to protocol-mandated procedures on the Adverse Events eCRF. All other untoward medical occurrences observed during the screening period, including exacerbation or changes in medical history are to be considered medical history. See Section 7 Adverse Events and Toxicity Management for additional details.

6.3. Treatment Assessments (Baseline/Day 1, Day 2 and Day 3)

The date of randomization will be considered Day 1. The following evaluations are to be completed at the Day 1 visit. The investigator must have confirmed eligibility before proceeding with randomization on the Day 1 visit. If the screening and Day 1 visits occur on the same day, no procedures need to be repeated. Participants must complete the following assessments before being administered study drug:

- Vital signs (heart rate, temperature, blood pressure, body weight) pre-infusion, post-infusion, and when post infusion observation is completed
- Respiratory status:
  - Respiratory rate
  - Oxygenation: SpO_2 on room air
- Complete physical examination (Day 1)
- Symptom-directed physical examination (Day 2, Day 3)
- Review of AEs and concomitant medications
- Obtain blood samples as described in Section 6.6 (Day 1, Day 3)
- Nasopharyngeal swab samples for SARS-CoV-2 RT-qPCR testing and possible viral resistance testing
- Completion of the COVID-adapted-FLU-PRO questionnaire (if available)

- Administration of study drug. Instructions on study drug administration are available in a separate manual.

Remdesivir infusions will be administered to participants at the site under close supervision or in the participant's home by a home health service provider. Healthcare professionals administering RDV infusions should have the appropriate medication available for immediate use in case of hypersensitivity or infusion related reactions. The participant should be treated according to the SOC for management of hypersensitivity reaction or infusion related reactions. Post infusion monitoring should be done according to site or home health protocol.

6.4. Post-treatment Assessments (Day 4 through Day 14)

The following evaluations are to be completed at the specified study days. The study visits are to be completed on the protocol-specified visit date (based on the Day 1 visit). The Day 7 and Day 14 visits have a ± 1 day window.

- Vital signs (heart rate, temperature, blood pressure), body weight (Day 7, Day 14)
- Review of AEs and concomitant medications and symptom assessment (Day 7, Day 14)
- Complete physical examination (Day 14)
- Symptom-directed physical examination (Day 7)
- Respiratory status (Day 7 and Day 14):
  - Respiratory rate
  - Oxygenation: SpO_2 on room air
- Obtain blood samples as described in Section 6.6 (Day 7, Day 14)
- Nasopharyngeal swab samples for SARS-CoV-2 RT-qPCR testing and possible viral resistance testing (Day 7, Day 14)
- Completion of the COVID-19-adapted FLU-PRO questionnaire (if available) (Days 4, 5, 6, 7, 8, 9, 10, 11, 12, 13, 14)

6.5. Day 28 Follow-up Assessment (± 5 Days)

The following evaluations are to be completed if the visit is conducted in person. The final evaluation can be performed by phone (only AEs and concomitant medications review needed if done by phone).

- Symptom directed physical examination, vital signs (heart rate, temperature, blood pressure), and body weight
- Respiratory status:
  - Respiratory rate

- Oxygenation: SpO_2 on room air

- Review of AEs and concomitant medications and symptom assessment (participant will be asked about current COVID-19 symptoms and that information will be used to assess AEs and determine the focus of the symptom-directed physical examinations)

6.6. Clinical Laboratory Assessments

Blood will be collected throughout the study as outlined below and in Appendix 3 Study Procedures Table.

6.6.1. Blood Samples

Blood sample collection for the following laboratory analyses will be performed at the specified time points:

- Chemistry profile: alkaline phosphatase, AST, ALT, total bilirubin, total protein, albumin, lactate dehydrogenase, creatine phosphokinase, bicarbonate, blood urea nitrogen, calcium, chloride, creatinine, glucose, lipase, magnesium, phosphorus, potassium, sodium, uric acid (Day 1, Day 3, Day 7, Day 14)

- Estimated glomerular filtration rate (screening [if required] Day 1, Day 3, Day 7, Day 14) according to:

  - Cockcroft-Gault formula for creatinine clearance for participants ≥ 18 years of age

Men:

$$\frac{(140 - \text{age in years}) \times (\text{wt in kg})}{72 \times (\text{serum creatinine in mg/dL})} = \text{CLcr (mL/min)}$$

Women:

$$\frac{(140 - \text{age in years}) \times (\text{wt in kg}) \times 0.85}{72 \times (\text{serum creatinine in mg/dL})} = \text{CLcr (mL/min)}$$

- Hematology profile: complete blood count with differential (Day 1, Day 3, Day 7, Day 14)

- Optional genomic testing (Day 1 or at any time during the study, if additional consent for pharmacogenomics testing is obtained)

6.6.2 Pharmacokinetic Assessments

All blood samples for PK assessments will be drawn from the arm opposite the one used to administer RDV or PTM. Sparse PK assessments will be conducted in all participants at participating sites and approximately 30 participants will have intensive PK assessments.

- Sparse PK assessments

  - Day 2: End of infusion and optional 2 hours post end of infusion

  - Day 3: Pre-dose (within 30 minutes of dosing) and end of infusion

- Intensive PK assessments
  - Day 1 and Day 3: at the following time points relative to the start time of infusion: 0 (pre-dose), 0.5, 0.75, 3, 6, 8, 12 (optional) and 24 hours

6.6.3 Biomarker Assessments (Optional)

Optional blood samples will be collected to measure serum and plasma biomarkers and may include immune-related markers, ARDS-related, and coagulation-related biomarkers and sACE2. DNA extraction for genetic analysis may be performed to evaluate SNPs which may be associated with host viral response, immune response, coagulation-related disorders and respiratory disorders.

- Serum (Day 1, 3, 14)
  - Immune-related biomarkers (IL-6, CRP, ferritin, procalcitonin)
  - ARDS-related (ang2)
- Plasma (Day 1, 3, 14)
  - Abnormal coagulation-related biomarkers (D-dimer, PT, aPTT, fibrinogen)

6.6.4 Virology Assessments

Nasopharyngeal swab samples will be used to assess SARS-CoV-2 viral load by RT-qPCR. Once viral load testing is complete, the remnant samples may be used to evaluate the emergence of viral resistance by SARS-CoV-2 sequencing and/or phenotypic testing.

6.7. Assessments for Early Discontinuation from Study

If a participant discontinues study dosing (for example, as a result of an AE), every attempt should be made to keep the participant in the study and continue to perform the required study-related follow-up and procedures (see Section 6.7.1, Criteria for Discontinuation of Study Treatment). If this is not possible or acceptable to the participant or investigator, the participant may be withdrawn from the study.

6.7.1. Criteria for Discontinuation of Study Treatment

Study medication may be discontinued in the following instances:

- Intercurrent illness that would, in the judgment of the investigator, affect assessments of clinical status to a significant degree. Following resolution of intercurrent illness, the participant may resume study dosing at the discretion of the investigator.

- Unacceptable toxicity, or toxicity that, in the judgment of the investigator, compromises the ability to continue study-specific procedures or is considered to not be in the participant's best interest
- Individual participants may be discontinued if they experience an ALT or AST ≥5 × ULN; or ALT > 3 × ULN and total bilirubin > 2 × ULN
- Other Grade 3 or Grade 4 abnormal laboratory results related to RDV
- Lack of efficacy
- Participant request to discontinue for any reason
- Participant noncompliance
- Discontinuation of the study at the request of Gilead, a regulatory agency or an institutional review board (IRB) or independent ethics committee (IEC)

6.8. End of Study

The end of the study will be the last participant's last observation (or visit).

6.9. Poststudy Care

The long-term care of the participant will remain the responsibility of their primary treating physician. Remdesivir is being supplied with curative intent. There is no provision for post-study availability.

6.10. Sample Storage

The stored biological samples may be used by Gilead or its research partner for future testing to provide additional data to answer questions that relate to the main study. At the end of this study, these samples may be retained in storage by Gilead for a period up to 15 years. If participants provide additional specific consent, residual PK samples may be destroyed no later than 15 years after the end of the study or per country requirements.

Any remaining specimens from nasopharyngeal swab samples collected during the study will be stored and retained for possible future virology-related testing. These stored samples may be used by the sponsor or its research partners for viral genotyping/phenotyping assays or their development, for retesting the amount of virus present in the sample, or for testing to learn more about how the study drug has worked or clinical laboratory testing to provide additional safety data. At the conclusion of this study, these samples may be retained in storage by Gilead for a period up to 15 years.

7. ADVERSE EVENTS AND TOXICITY MANAGEMENT

7.1. Definitions of Adverse Events and Serious Adverse Events

7.1.1. Adverse Events

An AE is any untoward medical occurrence in a clinical study participant administered an investigational product, which does not necessarily have a causal relationship with the treatment. An AE can therefore be any unfavorable and/or unintended sign, symptom, or disease temporally associated with the use of an investigational product, whether or not the AE is considered related to the investigational product. AEs may also include pre- or post-treatment complications that occur as a result of protocol specified procedures or special situations (Section 7.7).

An AE does not include the following:

- Medical or surgical procedures such as surgery, endoscopy, tooth extraction, and transfusion. The condition that led to the procedure may be an AE and must be reported.
- Preexisting diseases, conditions, or laboratory abnormalities present or detected before the screening visit that do not worsen
- Situations where an untoward medical occurrence has not occurred (e.g. hospitalization for elective surgery, social and/or convenience admissions)
- Overdose without clinical sequelae (Section 7.7.1)
- Any medical condition or clinically significant laboratory abnormality with an onset date before the consent form is signed and not related to a protocol-associated procedure is not an AE. It is considered to be preexisting and should be documented as medical history.
- Preexisting events or conditions that increase in severity or change in nature after the consent form is signed or as a consequence of participation in the clinical study will be considered AEs.

7.1.2. Serious Adverse Events

A SAE is defined as an event that, at any dose, results in the following:

- Death
- Life-threatening (Note: The term “life-threatening” in the definition of “serious” refers to an event in which the participant was at risk of death at the time of the event; it does not refer to an event that hypothetically might have caused death if it were more severe.)
- In-patient hospitalization or prolongation of existing hospitalization
- Persistent or significant disability/incapacity

- A congenital anomaly/birth defect
- A medically important event or reaction: such events may not be immediately life-threatening or result in death or hospitalization but may jeopardize the participant or may require intervention to prevent one of the other outcomes constituting SAEs. Medical and scientific judgment must be exercised to determine whether such an event is a reportable under expedited reporting rules. Examples of medically important events include intensive treatment in an emergency room or at home for allergic bronchospasm; blood dyscrasias or convulsions that do not result in hospitalization; and development of drug dependency or drug abuse.

7.2. Assessment of Adverse Events and Serious Adverse Events

The investigator or qualified subinvestigator is responsible for assessing AEs and SAEs for causality and severity, and for final review and confirmation of accuracy of event information and assessments.

7.2.1. Assessment of Causality for Study Drugs and Procedures

The investigator or qualified subinvestigator is responsible for assessing the relationship to study drug using clinical judgment and the following considerations:

- **No**: Evidence exists that the AE has an etiology other than the study drug. For SAEs, an alternative causality must be provided (e.g., preexisting condition, underlying disease, intercurrent illness, or concomitant medication).
- **Yes**: There is reasonable possibility that the event may have been caused by the study drug.

It should be emphasized that ineffective treatment should not be considered as causally related in the context of AE reporting.

The relationship to study procedures (e.g., invasive procedures such as venipuncture or biopsy) should be assessed using the following considerations:

- **No:** Evidence exists that the AE has an etiology other than the study procedure.
- **Yes:** The AE occurred as a result of protocol procedures, (e.g., venipuncture)

7.2.2. Assessment of Severity

The severity of AEs will be graded using the Division of AIDS (DAIDS) Table for Grading the Severity of Adult and Pediatric Adverse Events, Version 2.1 dated July 2017. For each episode, the highest grade attained should be reported as defined in the grading scale. The DAIDS scale is available at the following location:

https://rsc.niaid.nih.gov/sites/default/files/daidsgradingcorrectedv21.pdf

7.3. Investigator Requirements and Instructions for Reporting Adverse Events and Serious Adverse Events

7.3.1 Requirements for Collection Prior to Study Drug Initiation

After informed consent, but prior to initiation of study medication, the following types of events must be reported on the applicable eCRFs: all SAEs and AEs related to protocol-mandated procedures.

7.3.2. Adverse Events

Following initiation of study medication, collect all AEs, regardless of cause or relationship, throughout the duration of the study, including the protocol-defined follow-up period, must be reported on the eCRFs as instructed.

All AEs should be followed up until resolution or until the AE is stable, if possible. Gilead may request that certain AEs be followed beyond the protocol-defined follow-up period.

7.3.3. Serious Adverse Events

All SAEs, regardless of cause or relationship, that occur after the participant first consents to participate in the study (i.e., signing the informed consent) and throughout the duration of the study, including the post-treatment follow-up visit, must be reported on the applicable eCRFs and PVE as instructed below in this section. This also includes any SAEs resulting from protocol-associated procedures performed after informed consent is signed.

Any SAEs and deaths that occur after the post-treatment follow-up visit but within 30 days of the last dose of study drug, regardless of causality, should also be reported.

Investigators are not obligated to actively seek SAEs after the protocol-defined follow-up period; however, if the investigator learns of any SAEs that occur after the protocol-defined follow-up period has concluded and the event is deemed relevant to the use of study drug, the investigator should promptly document and report the event to Gilead PVE.

- All SAEs will be recorded in the case report form (CRF)/eCRF database within 24 hours.
- If the electronic SAE (eSAE) system becomes available after study start, all SAEs previously reported via paper need to be captured in the eSAE system.

7.3.3.1. Electronic Serious Adverse Event Reporting Process

- Site personnel record all SAE data on the applicable eCRFs and from there transmit the SAE information to Gilead PVE within 24 hours of the investigator’s knowledge of the event. Detailed instructions can be found in the eCRF completion guidelines (eCCGs).

- If it is not possible to record and transmit the SAE information electronically, record the SAE on the paper SAE reporting form and submit within 24 hours to:

Gilead PVE
Email: PPD
or
Fax: PPD

- As soon as it is possible to do so, any SAE reported via paper must be transcribed on the applicable eCRFs according to instructions and within the timelines outlined in the eCCGs.

- If an SAE has been reported via a paper form because the eCRF database has been locked, no further action is necessary.

- For fatal or life-threatening events, copies of hospital case reports, autopsy reports, and other documents are also to be submitted by email or fax when requested and applicable. Transmission of such documents should occur without personal participant identification, maintaining the traceability of a document to the participant identifiers.

- Additional information may be requested to ensure the timely completion of accurate safety reports.

- Any medications necessary for treatment of the SAE must be recorded onto the concomitant medication section of the participant's eCRF and the SAE narrative section of the Safety Report Form eCRF.

7.4. Gilead Reporting Requirements

Depending on relevant local legislation or regulations, including the applicable US FDA Code of Federal Regulations, the EU Clinical Trials Directive (2001/20/EC) and relevant updates, and other country-specific legislation or regulations, Gilead may be required to expedite to worldwide regulatory agencies reports of SAEs, serious adverse drug reactions, or SUSARs. In accordance with the EU Clinical Trials Directive (2001/20/EC), Gilead or a specified designee will notify worldwide regulatory agencies and the relevant IEC in concerned Member States of applicable SUSARs as outlined in current regulations.

Assessment of expectedness for SAEs will be determined by Gilead using reference safety information specified in the IB or relevant local label as applicable.

All investigators will receive a safety letter notifying them of relevant SUSAR reports associated with any study drug. The investigator should notify the IRB or IEC of SUSAR reports as soon as is practical, where this is required by local regulatory agencies, and in accordance with the local institutional policy.

7.5. Clinical Laboratory Abnormalities and Other Abnormal Assessments as Adverse Events or Serious Adverse Events

Laboratory abnormalities without clinical significance are not recorded as AEs or SAEs. However, laboratory abnormalities (e.g., clinical chemistry, hematology, and urinalysis) that require medical or surgical intervention or lead to study drug interruption, modification, or discontinuation must be recorded as an AE, as well as an SAE, if applicable. In addition, laboratory or other abnormal assessments (e.g., electrocardiogram, x-rays, vital signs) that are associated with signs and/or symptoms must be recorded as an AE or SAE if they meet the definition of an AE or SAE as described in Sections 7.1.1 and 7.1.2 If the laboratory abnormality is part of a syndrome, record the syndrome or diagnosis (e.g., anemia), not the laboratory result (i.e., decreased hemoglobin).

Severity should be recorded and graded according to the DAIDS Table for Grading the Severity of Adult and Pediatric Adverse Events, Version 2.1 dated July 2017. For AEs associated with laboratory abnormalities, the event should be graded on the basis of the clinical severity in the context of the underlying conditions; this may or may not be in agreement with the grading of the laboratory abnormality.

7.6. Toxicity Management

Remdesivir infusions will be administered to participants at the site under close supervision or in the participant's home by a home health service provider. Healthcare professionals administering RDV infusions should have the appropriate medication available for immediate use in case of hypersensitivity or infusion related reactions. The participant should be treated according to the SOC for management of hypersensitivity reaction or infusion related reactions. Post infusion monitoring should be done according to site or home health protocol.

7.7. Special Situations Reports

7.7.1. Definitions of Special Situations

Special situation reports include all reports of medication error, abuse, misuse, overdose, occupational exposure, drug interactions, exposure via breastfeeding, unexpected benefit, transmission of infectious agents via the product, counterfeit of falsified medicine, and pregnancy regardless of an associated AE.

Medication error is any unintentional error in the prescribing, dispensing, preparation for administration or administration of an investigational product while the medication is in the control of a health care professional, patient, or consumer. Medication errors may be classified as a medication error without an AE, which includes situations of missed dose; medication error with an AE; intercepted medication error; or potential medication error.

Abuse is defined as persistent or sporadic intentional excessive use of an investigational product by a participant.

Misuse is defined as any intentional and inappropriate use of an investigational product that is not in accordance with the protocol instructions or the local prescribing information.

An overdose is defined as an accidental or intentional administration of a quantity of an investigational product given per administration or cumulatively which is above the maximum recommended dose as per protocol or in the product labeling (as it applies to the daily dose of the participant in question). In cases of a discrepancy in drug accountability, overdose will be established only when it is clear that the participant has taken the excess dose(s). Overdose cannot be established when the participant cannot account for the discrepancy except in cases in which the investigator has reason to suspect that the participant has taken the additional dose(s).

Occupational exposure is defined as exposure to an investigational product as a result of one's professional or non-professional occupation.

Drug interaction is defined as any drug/drug, drug/food, or drug/device interaction.

Unexpected benefit is defined as an unintended therapeutic effect where the results are judged to be desirable and beneficial.

Transmission of infectious agents is defined as any suspected transmission of an infected agent through a Gilead investigational product.

Counterfeit or falsified medicine: Any investigational product with a false representation of: a) its identity, b) its source, or c) its history.

7.7.2. Instructions for Reporting Special Situations

7.7.2.1. Instructions for Reporting Pregnancies

The investigator should report pregnancies in female study participants that are identified after initiation of study drug and throughout the study, including the post study drug follow-up period, to Gilead PVE using the pregnancy report form within 24 hours of becoming aware of the pregnancy.

Refer to Section 7.3 for PVE contact information and the eCCGs for full instructions on the mechanism of pregnancy reporting.

The pregnancy itself is not considered an AE nor is an induced elective abortion to terminate a pregnancy without medical reasons.

Any premature termination of pregnancy (e.g., a spontaneous abortion, an induced therapeutic abortion due to complications or other medical reasons) must be reported within 24 hours as an SAE. The underlying medical reason for this procedure should be recorded as the AE term.

A spontaneous abortion is always considered to be an SAE and will be reported as described in Section 7.3.2. Furthermore, any SAE occurring as an adverse pregnancy outcome post study must be reported to Gilead PVE.

The participant should receive appropriate monitoring and care until the conclusion of the pregnancy. The outcome should be reported to Gilead PVE using the pregnancy outcome report form. If the end of the pregnancy occurs after the study has been completed, the outcome should be reported directly to Gilead PVE. Gilead PVE contact information is as follows: email: PPD and fax: PPD.

Pregnancies of female partners of male study participants exposed to Gilead or other study drugs must also be reported and relevant information should be submitted to Gilead PVE using the pregnancy and pregnancy outcome forms within 24 hours. If the end of the pregnancy occurs after the study has been completed, the outcome should be reported directly to Gilead PVE, fax number PPD or email PPD.

Refer to Appendix 4 Pregnancy Precautions, Definition for Female of Childbearing Potential, and Contraceptive Requirements.

7.7.2.2. Reporting Other Special Situations

All other special situation reports must be reported on the special situations report form and forwarded to Gilead PVE within 24 hours of the investigator becoming aware of the situation. These reports must consist of situations that involve study drug and/or Gilead concomitant medications, but do not apply to non-Gilead concomitant medications.

Special situations involving non-Gilead concomitant medications do not need to be reported on the special situations report form; however, special situations that result in AEs due to a non-Gilead concomitant medication, must be reported as an AE.

Any inappropriate use of concomitant medications prohibited by this protocol should not be reported as “misuse,” but may be more appropriately documented as a protocol deviation.

Refer to Section 7.3 for PVE contact information and the eCCGs for instructions on special situation reporting.

All clinical sequelae in relation to these special situation reports will be reported as AEs or SAEs at the same time using the AE eCRF and/or the SAE report form. Details of the symptoms and signs, clinical management, and outcome will be reported, when available.

8. STATISTICAL CONSIDERATIONS

8.1. Analysis Objectives and Endpoints

8.1.1. Analysis Objectives

The primary objectives of this study are as follows:

- To evaluate the efficacy of RDV in reducing the rate of hospitalization or death when given over 3 days to non-hospitalized participants with early stage COVID-19. Hospitalization is defined as ≥ 24 hours of acute care.
- To evaluate the safety of RDV administered in an outpatient setting

The secondary objectives of this study are as follows:

- To determine the antiviral activity of RDV on SARS-CoV-2 viral load
- To assess the impact of RDV on symptom duration and severity

The exploratory objectives of this study are as follows:

- To assess the impact of RDV on other clinical outcomes
- To evaluate the emergence of viral resistance to RDV
- To identify and assess associations of host biomarkers with disease progression and treatment response
- To assess the PK of RDV and its metabolites in participants with COVID-19

8.1.2. Primary Endpoint

The primary endpoint of this study are as follows:

- The composite endpoint of hospitalization or death from any cause by Day 14
- The proportion of participants with treatment-emergent AEs

8.1.3. Secondary Endpoint

The secondary endpoints of this study are as follows:

- All-cause mortality at Day 28
- Rate of hospitalization by Day 28

- Time-weighted average change in SARS-CoV-2 viral load from baseline to Day 7
- Time to resolution of COVID-19-related symptoms

8.1.4. Other Endpoints of Interest

Other endpoints of interest are as follows:

- Time to symptom resolution of each domain of COVID-19-adapted FLU-PRO
- Change from baseline in COVID-19-adapted FLU-PRO total score and score in each domain
- Time-weighted average change in SARS-CoV-2 viral load from baseline to Day 14
- Time to first negative SARS-CoV-2 PCR
- Emergence of viral resistance to RDV
- Baseline levels and change from baseline for inflammation/immune-related, ARDS-related and coagulation-related biomarkers
- Proportion of participants progressing to outpatient O_2 requirement by Day 14
- Proportion of participants admitted to the intensive care unit by Day 28
- Proportion of participants started on mechanical ventilation by Day 28
- The plasma concentrations and PK parameters of RDV and metabolites

8.2. Planned Analyses

8.2.1. Interim Analysis

Prior to the final analysis, interim analyses may be conducted and the analyses may be submitted to regulatory agencies to seek guidance for the overall clinical development program m.

8.2.1.1. Data Monitoring Committee Analysis

An external data monitoring committee (DMC) will review safety data on a regular basis. One futility interim analysis is planned after approximately 50% of participants complete Day 14. Enrollment may pause for the IDMC review if enrollment is faster than projected. The DMC will review the interim safety data and summary of primary efficacy data by treatment group and make recommendation of stopping enrollment to a treatment due to lack of efficacy.

8.2.2. Final Analysis

The final analysis will be performed after all participants have completed the study, outstanding data queries have been resolved or adjudicated as unresolvable, and the data have been cleaned and finalized. The analysis of the primary endpoint of hospitalization or death from any cause by Day 14 will be conducted at the time of the final analysis and will be tested at the 0.05 significance level.

8.3. Analysis Conventions

8.3.1. Analysis Sets

8.3.1.1. Efficacy

The primary analysis set for efficacy analysis is defined as the Full Analysis Set, which will include all participants who (1) are randomized into the study and (2) have received at least 1 dose of study treatment if randomized to the RDV treatment group. Participants will be grouped according to the treatment to which they were randomized.

8.3.1.2. Safety

The primary analysis set for safety analyses is defined as the Safety Analysis Set, which will include all participants who (1) are randomized into the study and (2) have received at least 1 dose of study treatment if randomized to the RDV treatment group. Participants will be grouped according to the treatment to which they received.

8.3.1.3. Pharmacokinetics

The PK Analysis Set will include all randomized participants who received at least 1 dose of RDV and had at least 1 nonmissing PK concentration datum reported by the PK laboratory for each respective analyte.

8.3.2. Data Handling Conventions

Natural logarithm transformation for PK parameters will be applied for PK analysis.

For summary statistics, PK concentration values below the limit of quantitation will be treated as zero at pre-dose and one-half of the lower limit of quantitation (LLOQ) for post dose time points

Laboratory data that are continuous in nature but are less than the LLOQ or above the upper limit of quantitation, will be imputed to the value of the lower or upper limit plus or minus 1 significant digit, respectively (e.g., if the result of a continuous laboratory test is < 20, a value of 19 will be assigned).

Missing data can have an impact upon the interpretation of the trial data. In general, values for missing data will not be imputed. However, a missing pre-treatment laboratory result would be treated as normal (i.e., no toxicity grade) for the laboratory abnormality summary.

All available data for participants that do not complete the study will be included in data listings

8.4. Demographic and Baseline Characteristics Analysis

Demographic and baseline measurements will be summarized using standard descriptive methods. Demographic summaries will include sex, race/ethnicity, randomization stratification group, and age. For categorical demographic and baseline characteristics, a Cochran-Mantel-Haenszel test will be used to compare treatment groups. For continuous demographic and baseline characteristics, a Wilcoxon rank sum test will be used to compare treatment groups.

8.5. Efficacy Analysis

8.5.1. Primary Analysis

The primary endpoint of the study is the composite endpoint of hospitalization or death from any cause by Day 14. The null hypothesis being tested is whether the ratio of proportion of hospitalization or death is the same for the 2 treatment groups (i.e., whether ratio is equal to 1 across all strata).

The proportion of participants hospitalized or who died from the date of randomization up to Day 14 will be estimated using Kaplan-Meier methods for each stratification stratum. The hypothesis test (p-value) will be calculated in following steps:

1. The difference between treatment groups in cumulative proportion in log scale will be calculated for each stratification stratum.

2. Variance for the difference of proportion between treatment groups will be estimated using Greenwood's formula.

3. The differences for each stratum will be combined, weighted by the inverse of the variance of the differences for each stratification stratum, into overall difference between 2 treatment groups.

4. P-value for the test of no difference between treatment groups will be calculated

Participants with missing outcomes for the primary endpoint due to prematurely discontinuation of the study will be censored at the date of last contact.

8.5.2. Secondary Analyses

The secondary endpoint of hospitalization by Day 28 will be analyzed similarly as in the primary analysis. The all-cause mortality at Day 28 will be compared between the 2 treatment groups using a Fisher exact test. other endpoints of interest related to proportion of participants will be compared between treatment groups using a chi-square test or Fisher exact test. Endpoints that are measured as time to first event will be compared between treatment groups using the log-

rank test and continuous endpoints will be compared between treatment groups using a Wilcoxon rank sum test or analysis of variance model.

8.6. Safety Analysis

All safety data collected on or after the randomization date through the Day 28 visit will be summarized by treatment group (according to the study drug received). Data for the pretreatment will be included in data listings.

8.6.1. Extent of Exposure

A participant's extent of exposure to study drug data will be generated from the study drug administration data. Exposure data will be summarized by treatment group.

8.6.2. Adverse Events

Clinical and laboratory AEs will be coded using the Medical Dictionary for Regulatory Activities. System Organ Class, High-Level Group Term, High-Level Term, Preferred Term, and Lower-Level Term will be attached to the clinical database.

Events will be summarized on the basis of the date of onset for the event. A treatment-emergent adverse event will be defined as any adverse event that begins on or after the randomization date up to the date of last dose of study drug plus 30 days.

Summaries (number and percentage of participants) of TEAEs (by system organ class and preferred term) will be provided by treatment group.

8.6.3. Laboratory Evaluations

Selected laboratory data will be summarized using only observed data. Data and change from baseline at all scheduled time points will be summarized.

Graded laboratory abnormalities will be defined using the grading scheme in DAIDS Table for Grading the Severity of Adult and Pediatric Adverse Events, Version 2.1 dated July 2017.

Incidence of treatment-emergent laboratory abnormalities, defined as values that increase at least 1 toxicity grade from baseline at any time post baseline up to the end date of the study, will be summarized by treatment group. If baseline data are missing, then any graded abnormality (ie, at least a Grade 1) will be considered treatment-emergent.

Laboratory abnormalities that occur before the first dose of study drug or after the participant has been discontinued from treatment for more than 30 days will be included in a data listing.

8.7. Adjustments for Multiplicity

No adjustments for multiple comparisons are needed for the primary analysis of this study.

8.8. Pharmacokinetic Analysis

Plasma concentrations and PK parameters for RDV and metabolites will be listed and summarized using descriptive statistics by treatment.

8.9. Sample Size

The sample size computation is based on ratio rate of hospitalization or death by Day 14 for the placebo treatment group. The ratio represents the rate of hospitalization or death for the RDV treatment group relative to the placebo treatment group. The sample size needed to detect a given ratio for a 1:1 randomization using a 2-tailed test at level α is given by:

$$\frac{(z_{\alpha/2} + z_{\beta})^2}{(\phi - 1)^2 p_2^2} [p_1(1 - p_1) + p_2(1 - p_2)]$$

Where ϕ is the ratio (p_1/p_2), p_1 and p_2 are the rate of hospitalization or death for each treatment group, and $z_{\alpha/2}$ and z_{β} are the 1- α /2 and β quantiles of the standard normal distribution.

A sample size of 1230 participants (615 in each group with 1:1 randomization) achieves > 90% power to detect a ratio of 0.65 (RDV to placebo hospitalization or death rate) using a two-sided significance level of 0.05 assuming hospitalization or death rate is 20% in the placebo group and a 5% drop out rate. The power is expected to be similar under the primary analysis using the Kaplan-Meier methods incorporating stratification factors.

The sample size calculation was done using software PASS (Version 14.0, module of Tests for Two Proportions).

8.10. Independent Data Monitoring Committee

An external multidisciplinary IDMC will review the progress of the study, perform interim reviews of safety data on a regular basis and provide recommendation(s) to Gilead whether the nature, frequency, and severity of AEs associated with study treatment warrant the early termination of the study in the best interests of the participants, whether the study should continue as planned, or the study should continue with modifications. The IDMC will review the interim futility analysis and make a recommendation of stopping enrollment to the study if the pre-specified futility stopping criteria are met.

The IDMC may also provide recommendations as needed regarding study design.

The IDMC's specific activities will be defined by a mutually agreed charter, which will define the DMC's membership, conduct and meeting schedule

While the IDMC will be asked to advise Gilead regarding future conduct of the study, including possible early study termination, Gilead retains final decision-making authority on all aspects of the study.

9. RESPONSIBILITIES

9.1. Investigator Responsibilities

9.1.1. Good Clinical Practice

The investigator will ensure that this study is conducted in accordance with International Council for Harmonisation (of Technical Requirements for Pharmaceuticals for Human Use) (ICH) E6(R2) addendum to its guideline for GCP and applicable laws and regulations.

9.1.2. Financial Disclosure

The investigator and subinvestigators will provide prompt and accurate documentation of their financial interest or arrangements with Gilead, or proprietary interests in the investigational drug during the course of a clinical study. This documentation must be provided prior to the investigator's (and any subinvestigator's) participation in the study. The investigator and subinvestigator agree to notify Gilead of any change in reportable interests during the study and for 1 year following completion of the study. Study completion is defined as the date when the last participant completes the protocol-defined activities.

9.1.3. Institutional Review Board/Independent Ethics Committee Review and Approval

The investigator (or Gilead as appropriate according to local regulations) will submit this protocol, informed consent form, and any accompanying material to be provided to the participant (such as advertisements, participant information sheets, or descriptions of the study used to obtain informed consent) to an IRB/IEC. The investigator will not begin any study participant activities until approval from the IRB/IEC has been documented and provided as a letter to the investigator.

Before implementation, the investigator will submit to and receive documented approval from the IRB/IEC any modifications made to the protocol or any accompanying material to be provided to the participant after initial IRB/IEC approval, with the exception of those necessary to reduce immediate risk to study participants.

9.1.4. Informed Consent

The investigator is responsible for obtaining written informed consent from each individual participating in this study after adequate explanation of the aims, methods, objectives, and potential hazards of the study before undertaking any study-related procedures. The investigator must use the most current IRB- or IEC-approved consent form for documenting written informed consent. Each informed consent (or assent as applicable) will be appropriately signed and dated by the participant or the participant's legally authorized representative and the person conducting the consent discussion, and also by an impartial witness if required by IRB or IEC or local requirements.

The consent form will inform participants about genomic testing and/or planned sample retention. In addition to the study-specific informed consent to be signed by each participant in the study, participants will be required to document agreement to provide additional samples or to allow the use of the remainder of their already collected specimens for optional future research, in accordance with applicable regulations. In addition to the study-specific informed consent to be signed by each participant in the study, participants will be required to document agreement to provide additional samples for optional genomic research. The results of the tests done on the samples will not be given to the participant or the investigator.

9.1.5. Confidentiality

The investigator must assure that participants' anonymity will be strictly maintained and that their identities are protected from unauthorized parties. Only an identification code and any other unique identifier(s) as allowed by local law (such as year of birth) will be recorded on any form or biological sample submitted to Gilead, IRB or IEC, or the laboratory. Laboratory specimens must be labeled in such a way as to protect participant identity while allowing the results to be recorded to the proper participant. Refer to specific laboratory instructions. NOTE: The investigator must keep a screening log with details for all participants screened and enrolled in the study, in accordance with the site procedures and regulations. Participant data will be processed in accordance with all applicable regulations.

The investigator agrees that all information received from Gilead, including but not limited to the IB, this protocol, CRF/eCRF, the study drug, and any other study information, remain the sole and exclusive property of Gilead during the conduct of the study and thereafter. This information is not to be disclosed to any third party (except employees or agents directly involved in the conduct of the study or as required by law) without prior written consent from Gilead. The investigator further agrees to take all reasonable precautions to prevent the disclosure by any employee or agent of the study site to any third party or otherwise into the public domain.

9.1.6. Study Files and Retention of Records

The investigator must maintain adequate and accurate records to enable the conduct of the study to be fully documented and the study data to be subsequently verified. These documents should be classified into at least the following 2 categories: (1) investigator's study file, and (2) participant clinical source documents.

The investigator's study file will contain the protocol/amendments, electronic completed participant CRFs, IRB or IEC and governmental approval with correspondence, informed consent form(s), drug records, staff curriculum vitae and authorization forms, and other appropriate documents and correspondence.

The required source data should include sequential notes containing at least the following information for each participant:

- Participant identification
- Documentation that participant meets eligibility criteria, i.e., medical history, physical examination, and confirmation of diagnosis (to support inclusion and exclusion criteria);
- Documentation of the reason(s) a consented participant is not enrolled
- Participation in study (including study number)
- Study discussed and date of informed consent
- Dates of all visits
- Documentation that protocol-specific procedures were performed
- Results of efficacy parameters, as required by the protocol
- Start and end date (including dose regimen) of study drug, including dates of dispensing and return
- Record of all AEs and other safety parameters (start and end date, and including causality and severity), and documentation that adequate medical care has been provided for any AE
- Concomitant medication (including start and end date, dose if relevant; dose changes)
- Date of study completion and reason for early discontinuation, if it occurs

All clinical study documents must be retained by the investigator until at least 2 years or according to local laws, whichever is longer, after the last approval of a marketing application in an ICH region (i.e., US, Europe, or Japan) and until there are no pending or planned marketing applications in an ICH region; or, if no application is filed or if the application is not approved for such indication, until 2 years after the investigation is discontinued and regulatory authorities have been notified. Investigators may be required to retain documents longer if specified by regulatory requirements, by local regulations, or by an agreement with Gilead. The investigator must notify Gilead before destroying any clinical study records.

Should the investigator wish to assign the study records to another party or move them to another location, Gilead must be notified in advance.

If the investigator cannot provide for this archiving requirement at the study site for any or all of the documents, special arrangements must be made between the investigator and Gilead to store these records securely away from the site so that they can be returned sealed to the investigator

in case of an inspection. When source documents are required for the continued care of the participant, appropriate copies should be made for storage away from the site.

9.1.7. Case Report Forms

For each participant consented, an eCRF casebook will be completed by an authorized study staff member whose training for this function is completed in the EDC system. The eCRF casebook will only capture the data required per the protocol schedule of events and procedures. The Inclusion/Exclusion Criteria and Enrollment eCRFs should be completed only after all data related to eligibility have been received. Data entry should be performed in accordance with the eCCGs provided by the sponsor. Subsequent to data entry, a study monitor will perform source data verification (SDV) within the EDC system. System-generated or manual queries will be issued in the EDC system as data discrepancies are identified by the monitor or Gilead staff, who routinely review the data for completeness, correctness, and consistency. The site investigator or site coordinator or other designee is responsible for responding to the queries in a timely manner, within the system, either by confirming the data as correct or updating the original entry, and providing the reason for the update (e.g. data entry error). Original entries as well as any changes to data fields will be stored in the audit trail of the system. At a minimum, prior to any interim time points or database lock (as instructed by Gilead), the investigator will use his/her log in credentials to confirm that the forms have been reviewed, and that the entries accurately reflect the information in the source documents. At the conclusion of the study, Gilead will provide the site investigator with a read-only archive copy of the data entered by that site. This archive must be stored in accordance with the records retention requirements outlined in Section 9.1.6.

9.1.8. Investigator Inspections

The investigator will make available all source documents and other records for this study to Gilead's appointed study monitors, to IRBs/IECs, or to regulatory authority or health authority inspectors.

9.1.9. Protocol Compliance

The investigator is responsible for ensuring the study is conducted in accordance with the procedures and evaluations described in this protocol.

9.2. Sponsor Responsibilities

9.2.1. Protocol Modifications

Protocol modifications, except those intended to reduce immediate risk to study participants, may be made only by Gilead. The investigator must submit all protocol modifications to the IRB/IEC in accordance with local requirements and receive documented IRB/IEC approval before modifications can be implemented.

9.2.2. Study Report and Publications

A clinical study report will be prepared and provided to the regulatory agency. Gilead will ensure that the report meets the standards set out in the ICH Guideline for Structure and Content of Clinical Study Reports (ICH E3). Note that an abbreviated report may be prepared in certain cases.

Investigators in this study may communicate, orally present, or publish in scientific journals or other scholarly media only after the following conditions have been met:

The results of the study in their entirety have been publicly disclosed by or with the consent of Gilead in an abstract, manuscript, or presentation form or the study has been completed at all study sites for at least 2 years

The investigator will submit to Gilead any proposed publication or presentation along with the respective scientific journal or presentation forum at least 30 days before submission of the publication or presentation.

No such communication, presentation, or publication will include Gilead's confidential information (see Section 9.1.5).

The investigator will comply with Gilead's request to delete references to its confidential information (other than the study results) in any paper or presentation and agrees to withhold publication or presentation for an additional 60 days in order to obtain patent protection if deemed necessary.

9.3. Joint Investigator/Sponsor Responsibilities

9.3.1. Payment Reporting

Investigators and their study staff may be asked to provide services performed under this protocol, e.g., attendance at Investigator Meetings. If required under the applicable statutory and regulatory requirements, Gilead will capture and disclose to Federal and State agencies any expenses paid or reimbursed for such services, including any clinical study payments, meal, travel expenses or reimbursements, consulting fees, and any other transfer of value.

9.3.2. Access to Information for Monitoring

The monitor is responsible for routine review of the CRF/eCRF at regular intervals throughout the study to verify adherence to the protocol and the completeness, consistency, and accuracy of the data being entered on them. The monitor should have access to any participant records needed to verify the entries in the CRF/eCRF. The investigator agrees to cooperate with the monitor to ensure that any problems detected through any type of monitoring (central, on site) are resolved.

9.3.3. Access to Information for Auditing or Inspections

Representatives of regulatory authorities or of Gilead may conduct inspections or audits of the clinical study. If the investigator is notified of an inspection by a regulatory authority the investigator agrees to notify the Gilead medical monitor immediately. The investigator agrees to provide to representatives of a regulatory agency or Gilead access to records, facilities, and personnel for the effective conduct of any inspection or audit.

9.3.4. Study Discontinuation

Both Gilead and the investigator reserve the right to terminate the study at any time. Should this be necessary, both parties will arrange discontinuation procedures and notify the participants, appropriate regulatory authority(ies), IRBs, and IECs. In terminating the study, Gilead and the investigator will assure that adequate consideration is given to the protection of the participants' interests.

10. REFERENCES

Beigel JH, Tomashek KM, Dodd LE, Mehta AK, Zingman BS, Kalil AC, et al. Remdesivir for the Treatment of Covid-19 - Preliminary Report. N Engl J Med 2020.

Committee for Medicinal Products for Human Use (CHMP). Background review for cyclodextrins used as excipients in the context of the revision of the guideline on 'Excipients in the label and package leaflet of medicinal products for human use' (CPMP/463/00 Rev. 1). Draft report published in support to the propylene glycol Q&A document. For information only. European Medicines Agency (EMA) 20 November, 2014.

Nicholson KG, Aoki FY, Osterhaus AD, Trottier S, Carewicz O, Mercier CH, et al. Efficacy and safety of oseltamivir in treatment of acute influenza: a randomised controlled trial. Neuraminidase Inhibitor Flu Treatment Investigator Group. Lancet 2000;355 (9218):1845-50.

Sheahan TP, Sims AC, Graham RL, Menachery VD, Gralinski LE, Case JB, et al. Broad-Spectrum Antiviral GS-5734 Inhibits Both Epidemic and Zoonotic Coronaviruses. Science translational medicine 2017;9 (396):eaal3653.

Stokes EK, Zambrano LD, Anderson KN, Marder EP, Raz KM, El Burai Felix S, et al. Coronavirus Disease 2019 Case Surveillance - United States, January 22-May 30, 2020. MMWR. Morbidity and mortality weekly report 2020;69 (24):759-65.

The Novel Coronavirus Pneumonia Emergency Response Epidemiology Team. The Epidemiological Characteristics of an Outbreak of 2019 Novel Coronavirus Diseases (COVID-19) — China, 2020. Chinese Center for Disease Control and Prevention 2020;2 (8):113-22.

U. S. Food and Drug Administration (FDA). Response: Request That the Food and Drug Administration (FDA) Issue an Emergency Use Authorization (EUA) for Emergency Use of Remdesivir for the Treatment of Hospitalized 2019 Coronavirus Disease (COVID-19) Patients. 01 May. 2020:

Williamson BN, Feldmann F, Schwarz B, Meade-White K, Porter DP, Schulz J, et al. Clinical benefit of remdesivir in rhesus macaques infected with SARS-CoV-2. Nature 2020.

World Health Organization (WHO). Coronavirus Disease (COVID-2019) Situation Reports. Available at: https://www.who.int/emergencies/diseases/novel-coronavirus-2019/situation-reports. Accessed: 18 March. 2020a:

World Health Organization (WHO). Report of the WHO-China Joint Mission on Coronavirus Disease 2019 (COVID-19). 16-24 February. 2020b.

11. APPENDICES

Appendix 1. Investigator Signature Page
Appendix 2. Pandemic Risk Assessment and Mitigation Plan
Appendix 3. Study Procedures Table
Appendix 4. Pregnancy Precautions, Definition for Female of Childbearing Potential, and Contraceptive Requirements

Appendix 1. Investigator Signature Page

GILEAD SCIENCES, INC.
333 LAKESIDE DRIVE
FOSTER CITY, CA 94404

STUDY ACKNOWLEDGMENT

A Phase 3 Randomized, Double-Blind Placebo-Controlled Trial to Evaluate the Efficacy and Safety of Remdesivir (GS-5734™) Treatment of COVID-19 in an Outpatient Setting

GS-US-540-9012, Original Protocol, 21 July 2020

This protocol has been approved by Gilead Sciences, Inc. The following signature documents this approval.

PPD
Name (Printed)
Senior Director, Clinical Research

PPD
Signature

21 July 2020
Date

INVESTIGATOR STATEMENT

I have read the protocol, including all appendices, and I agree that it contains all necessary details for me and my staff to conduct this study as described. I will conduct this study as outlined herein and will make a reasonable effort to complete the study within the time designated.

I will provide all study personnel under my supervision copies of the protocol and access to all information provided by Gilead Sciences, Inc. I will discuss this material with them to ensure that they are fully informed about the drugs and the study.

Principal Investigator Name (Printed)

Signature

Date

Site Number

Appendix 2. Pandemic Risk Assessment and Mitigation Plan

During an ongoing pandemic, potential risks associated with subjects being unable to attend study visits have been identified for this study.

These risks can be summarized as follows:

1) Study drug supplies to subjects and sites:

   a) Subjects may be unable to return to the site for a number of visits to get the study drug, or the site may be unable to accept any subject visits. Without study drugs, the subject would not be able to stay on the study drug as planned per protocol.

      Mitigation plan: Study drug supplies may be provided to the subject from the site without a clinic visit, once it is confirmed that the subject may safely continue on study drug as determined by the principal investigator (PI). A virtual study visit, via phone or video conferencing, must be performed prior to remote study drug resupply. At the earliest opportunity, the site will schedule in-person subject visits and return to the protocol's regular schedule of assessments. A qualified courier may be utilized to ship the study drug from sites to study subjects if permitted by local ethic committee (EC)/institutional review boards (IRB)/Regulatory Authority as applicable and with sponsor's approval.

   b) Shipments of study drug could be delayed because of transportation issues. Without study drug subject would not be able to stay on the study drug as planned per protocol.

      Mitigation plan: The sites' study drug inventory should be closely monitored. Site staff should notify the sponsor or delegate if they foresee shortage in study drug inventory or if there is any interruption in local shipping service. The sponsor will continue to monitor inventory at the study drug depot and study sites. Manual shipments will be triggered as necessary.

2) Subject safety monitoring and follow-up:

   a) Subjects may be unable or unwilling to come to the study site for their scheduled study visits as required per protocol.

      Mitigation plan: For subjects who may be unable or unwilling to visit the study site for their scheduled study visits as required per protocol, the PI or qualified delegate will conduct a virtual study visit, via phone or video conferencing, to assess the subject within target visit window date whenever possible. During the virtual study visit, the following information at minimum will be reviewed:

      i) Confirm if subject has experienced any AEs/ SAEs/special situations (including pregnancy) and follow-up on any unresolved AE/SAEs.

      ii) Review current list of concomitant medications and document any new concomitant medications.

iii) If applicable, confirm electronic diary questionnaires and patient-reported outcomes have been completed and transmitted.

iv) If applicable, confirm subjects study drug supply is sufficient to last until the next planned visit date. If study drug resupply is needed it will be provided as described above in (1).

v) If applicable, remind subject to maintain current dosing and to keep all dispensed study drug kits for return at the next on-site visit.

b) Subjects may be unable or unwilling to travel to the site for planned assessments (eg, safety blood draws); hence samples may not be sent for central lab analyses.

Mitigation plan: Local labs may be utilized as appropriate to monitor subject safety until the subject can return to the site for their regular follow-up per protocol. Any laboratory assessments conducted at a local lab due to the pandemic will be documented accordingly. Pregnancy testing may be performed using a home urine pregnancy test if local lab pregnancy testing is not feasible.

c) Subjects may be unable or unwilling to attend the study visit to sign an updated informed consent form version.

Mitigation plan: The site staff will follow their approved consent process and remain in compliance with local EC/IRB and national laws and regulations. Remote consent will be allowed if has been approved by the local EC/IRB. The consent process will be documented and confirmed by normal consent procedure at the earliest opportunity.

3) Protocol and monitoring compliance:

a) Protocol deviations may occur, in case scheduled visits cannot occur as planned per protocol.

Mitigation plan: If it is not possible to complete a required procedure, an unscheduled visit should be conducted as soon as possible when conditions allow. The situation should be recorded and explained as a protocol deviation. Any missed subject visits or deviation to the protocol due to the pandemic must be reported in the eCRF and described in the clinical study report. Any virtual study visits that are conducted in lieu of clinic visits due to the pandemic will be documented as a protocol deviation related to the pandemic.

b) Monitors may be unable to carry out source data review (SDR) or source data verification (SDV), or study drug accountability or assess protocol and GCP compliance. This may lead to delays in SDV, an increase in protocol deviations, or under reporting of AEs.

Mitigation plan: The study monitor is to remain in close communication with the site to ensure data entry and query resolution. In compliance with Gilead policy, a remote SDV should not be arranged). The study monitor is to reference the Study Monitoring Plan for

guidance on how to conduct a remote monitoring visit. The study staff is to save and document all relevant communication in the study files. The status of sites that cannot accept monitoring visits and/or subjects on site, must be tracked centrally and updated on a regular basis.

4) Missing data and data integrity:

   a) There may be an increased amount of missing data due to subjects missing visits/assessments. This could have an impact on the analysis and the interpretation of clinical trial data.

      Mitigation plan: Implications of a pandemic on methodological aspects for the study will be thoroughly assessed and documented, and relevant actions will be taken as appropriate (ie, modification of the statistical analysis plan) and in compliance with Regulatory Authorities' guidance. Overall, the clinical study report will describe the impact of the pandemic on the interpretability of study data.

Risks will be assessed continuously, and temporary measures will be implemented to mitigate these risks as part of a mitigation plan, as described above. These measures will be communicated to the relevant stakeholders as appropriate and are intended to provide alternate methods that will ensure the evaluation and assessment of the safety of subjects who are enrolled in this study.

Since these potential risks are considered mitigated with the implementation of these measures, the expected benefit-risk assessment of RDV in study subjects remains unchanged.

Appendix 3. Study Procedures Table

	Screening[a]	Baseline/ Day 1	Day 2	Day 3	Day 7 (± 1 day)	Day 14 (±1 day)	Day 28 Follow-up (± 5 days)
Written Informed Consent	X						
Medical History[b]	X						
Complete Physical Examination[c]	X	X				X	
Symptom Directed Physical Examination[d]			X	X	X		X
Height	X						
Vital Signs[e] and Weight	X	X	X	X	X	X	
Respiratory Status[f]	X	X	X	X	X	X	X
ALT, AST, Serum Creatinine, and Creatinine Clearance[g]	X						
Chemistry[h] and Hematology Panel[i]		X		X	X	X	
Pharmacogenomic Testing[j]	X						
Urine Pregnancy Test[k]	X						
SARS-CoV-2 RT-qPCR Testing and Potential Resistance Testing [l]		X	X	X	X	X	
Documentation of SARS-CoV-2 Infection	X						
Biomarker Sample Collection[m]		X		X		X	
Sparse PK[n]			X	X			
Intensive PK[o]		X		X			
Symptom Assessment[p]	X	X	X	X	X	X	X
FLU-PRO Questionnaire[q]		X	X	X	X	X	
Study Drug Dosing		X	X	X			
Adverse Events and Concomitant Medications	X	X	X	X	X	X	X

a Study visits may be performed in an outpatient setting, at the participant's home via tele-health, virtually or remotely, as permitted by local and institutional regulations.
b Medical history will include the date of first symptoms, overall symptoms, exposure source, demographics, baseline characteristics, allergies and all other medical history.
c Complete physical examination does not require urogenital/anorectal examination
d Symptom-directed physical examination will include at least cardiac and respiratory evaluation
e Vital signs include heart rate, temperature, and blood pressure
f Respiratory status includes respiratory rate and SpO_2 on room air

g Alanine aminotransferase (ALT) and aspartate aminotransferase (AST), serum creatinine, and creatinine clearance if prior history of renal or liver disease (if no history of severe renal or severe renal disease but with weight $\geq$ 48 kg or liver disease or if results available within the prior 3 months, can defer until Day 1)
h Chemistry: alkaline phosphatase, AST, ALT, total bilirubin, total protein, albumin, lactate dehydrogenase, creatine phosphokinase, bicarbonate, blood urea nitrogen, calcium, chloride, creatinine, glucose, lipase, magnesium, phosphorus, potassium, sodium, uric acid.
i Hematology: Complete blood count with differential
j Optional genomic testing can occur at Day 1. If not collected at Day 1, it can be collected at subsequent visits.
k Urine pregnancy test will only be done for women of childbearing potential.
l Nasopharyngeal swab samples will be collected and stored for SARS-CoV-2 RT-qPCR and potential resistance testing.
m Optional biomarkers: Serum and whole blood will be collected for participants that consent.
n Sparse PK will be collected from participants at participating sites at Day 2: end of infusion and optional 2 hours post end of infusion and Day 3: Pre-dose (within 30 minutes of dosing) and end of infusion.
o Intensive PK will be collected at selected sites and approximately 30 participants will have iPK assessments conducted.
p Participant will be asked about current COVID-19 symptoms and that information will be used to assess AEs and determine the focus of the symptom-directed physical examinations
q FLU-PRO questionnaire should be completed daily from Day 1 through Day 14 (if available)

Appendix 4. Pregnancy Precautions, Definition for Female of Childbearing Potential, and Contraceptive Requirements

1) Definitions

a. Definition of Childbearing Potential

For the purposes of this study, a female born participant is considered of childbearing potential until becoming post-menopausal, unless permanently sterile or with medically documented ovarian failure.

Women are considered to be in a postmenopausal state when they are ≥ 54 years of age with cessation of previously occurring menses for ≥ 12 months without an alternative cause.

Permanent sterilization includes hysterectomy, bilateral oophorectomy, or bilateral salpingectomy in a female participant of any age.

b. Definition of Male Fertility

For the purposes of this study, a male born participant is considered fertile after the initiation of puberty unless the participant is permanently sterile by bilateral orchidectomy or with medical documentation.

2) Contraception Requirements for Female Participants

a. Study Drug Effects on Pregnancy and Hormonal Contraception

Data from nonclinical studies of RDV have demonstrated no adverse effect on fertility or embryo-fetal development. Remdesivir has not yet been studied in pregnant women. Before enrolling into studies with RDV, women of childbearing potential must have pregnancy testing performed at screening.

Available data indicate that RDV potentially causes an interaction with hormonal contraception that is considered of limited significance. Hormonal methods must be used with a barrier method.

b. Contraception Requirements for Female Participants of Childbearing Potential

The inclusion of non-pregnant female participants of childbearing potential requires the use at least an acceptable contraceptive measure. Female participants must agree to 1 of the following from screening until 30 days after the last study drug dose:

- Complete abstinence from intercourse of reproductive potential. Abstinence is an acceptable method of contraception only when it is in line with the participant's preferred and usual lifestyle.

Or

- Consistent and correct use of 1 of the following methods of birth control listed below:
  - Non-hormonal intrauterine device (IUD)Hormonal IUD (must be used in conjunction with a barrier method)
  - Bilateral tubal occlusion (upon medical assessment of surgical success)
  - Vasectomy in the male partner (upon medical assessment of surgical success)

Or

- Female participants who wish to use a hormonally based method must use it in conjunction with a barrier method, preferably a male condom. Hormonal methods are restricted to those associated with the inhibition of ovulation. Hormonally-based contraceptives and barrier methods permitted for use in this protocol are as follows:
  - Hormonal methods (each method must be used with a barrier method, preferably male condom)
    - Oral contraceptives (either combined or progesterone only)
    - Injectable progesterone
    - Subdermal contraceptive implant
    - Transdermal contraceptive patch
    - Contraceptive vaginal ring
  - Barrier methods
    - Male condom (with or without spermicide)
    - Female condom (with or without spermicide)
    - Diaphragm with spermicide
    - Cervical cap with spermicide
    - Sponge with spermicide

Inclusion of methods of contraception in this list of permitted methods does not imply that the method is approved in any country or region. Methods should only be used if locally approved.

Female participants must also refrain from egg donation and in vitro fertilization during treatment and until the end of contraception requirement.

3) Contraception Requirements for Male Participants

During the study male participants with female partners of childbearing potential should use condoms when engaging in intercourse of reproductive potential.

4) Unacceptable Birth Control Methods

Birth control methods that are unacceptable include periodic abstinence (e.g.. calendar, ovulation, symptothermal, post-ovulation methods), withdrawal (coitus interruptus), spermicides only, and lactational amenorrhea method. A female condom and a male condom should not be used together.

5) Procedures to be Followed in the Event of Pregnancy

Participants will be instructed to notify the investigator if they become pregnant at any time during the study, or if they become pregnant within 30 days of last study drug dose. Participants who become pregnant or who suspect that they are pregnant during the study must report the information to the investigator. Participants whose partner has become pregnant or suspects she is pregnant during the study must report the information to the investigator. Instructions for reporting pregnancy, partner pregnancy, and pregnancy outcome are outlined in Section 7.7.2.1.

CLINICAL STUDY PROTOCOL

**Study Title:**	A Phase 3 Randomized, Double-Blind Placebo-Controlled Trial to Evaluate the Efficacy and Safety of Remdesivir (GS-5734™) Treatment of COVID-19 in an Outpatient Setting
**Sponsor:**	Gilead Sciences, Inc.<br>333 Lakeside Drive<br>Foster City, CA 94404
**IND Number:**	147753
**EudraCT Number:**	2020-003510-12
**Clinical Trials.gov Identifier:**	NCT04501952
**Indication:**	COVID-19
**Protocol ID:**	GS-US-540-9012
**Contact Information:**	The medical monitor name and contact information will be provided on the Key Study Team Contact List.

**Protocol Version/Date:**		
	Original:	21 July 2020
	Amendment 1:	11 August 2020
	Amendment 1.1 (United Kingdom):	07 October 2020
	Amendment 1.1 (Germany):	14 October 2020
	Amendment 2:	06 November 2020
	Amendment 3:	12 November 2020
	Amendment 4:	14 January 2021

This study will be conducted under United States Food and Drug Administration investigational new drug (IND) regulations (21 Code of Federal Regulations Part 312); however, sites located in the European Economic Area and Switzerland are not included under the IND and are considered non-IND sites.

CONFIDENTIALITY STATEMENT

The information contained in this document, particularly unpublished data, is the property or under control of Gilead Sciences, Inc., and is provided to you in confidence as an investigator, potential investigator, or consultant, for review by you, your staff, and an applicable institutional review board or independent ethics committee. The information is only to be used by you in connection with authorized clinical studies of the investigational drug described in the protocol. You will not disclose any of the information to others without written authorization from Gilead Sciences, Inc., except to the extent necessary to obtain informed consent from those persons to whom the drug may be administered.

TABLE OF CONTENTS

TABLE OF CONTENTS 2
LIST OF IN-TEXT TABLES 4
LIST OF IN-TEXT FIGURES 4
PROTOCOL SYNOPSIS 5
GLOSSARY OF ABBREVIATIONS AND DEFINITION OF TERMS 11
1. INTRODUCTION 13
1.1. Background 13
1.2. Remdesivir (RDV, GS-5734) 13
1.2.1. General Information 13
1.3. Rationale for This Study 13
1.4. Rationale for Duration of Treatment 14
1.5. Rationale for Dose Selection of Remdesivir 14
1.6. Risk/Benefit Assessment for the Study 15
1.7. Compliance 15
2. OBJECTIVES 16
3. STUDY DESIGN 17
3.1. Endpoints 17
3.2. Study Design 18
3.3. Study Treatments 18
3.4. Duration of Treatment 18
3.5. End of Study 19
3.6. Poststudy Care 19
3.7. Source Data 19
3.8. Biomarker Testing 19
3.8.1. Biomarker Samples for Optional Future Research 19
3.8.2. Biomarker Samples for Optional Genomic Research 20
4. PARTICIPANT POPULATION 21
4.1. Number of Participants and Participant Selection 21
4.1.1. Participant Replacement 21
4.2. Inclusion Criteria 21
4.3. Exclusion Criteria 22
5. INVESTIGATIONAL MEDICINAL PRODUCTS 23
5.1. Randomization, Blinding, and Treatment Codes Access 23
5.1.1. Randomization 23
5.1.2. Blinding 23
5.1.3. Procedures for Breaking Treatment Codes 23
5.2. Description and Handling of Remdesivir 24
5.2.1. Formulation 24
5.2.2. Packaging and Labeling 24
5.2.3. Storage and Handling 24
5.3. Dosage and Administration of Remdesivir 25
5.3.1. Administration Instructions 25
5.3.2. Infusion-related Reactions 25
5.4. Prior and Concomitant Medications 25
5.5. Accountability for Investigational Medicinal Product 26

5.5.1. Investigational Medicinal Product Return or Disposal 26
6. STUDY PROCEDURES 27
6.1. Participant Enrollment and Treatment Assignment 27
6.2. Pretreatment Assessments 27
6.2.1. Screening Visit 27
6.3. Treatment Assessments (Baseline/Day 1, Day 2, and Day 3) 28
6.4. Post-treatment Assessments (Day 4 Through Day 14) 29
6.5. Day 28 Follow-up Assessment (± 5 Days) 30
6.6. Assessments for Early Discontinuation from Study 30
6.7. Procedures and Specifications 31
6.7.1. Complete Physical Examination 31
6.7.2. Clinical Laboratory Assessments 31
6.7.3. Pharmacokinetic Assessments 32
6.7.4. Biomarker Assessments (Optional) 32
6.7.5. Virology Assessments 32
6.8. Criteria for Discontinuation of Study Treatment 33
6.9. End of Study 33
6.10. Poststudy Care 33
6.11. Sample Storage 33
7. ADVERSE EVENTS AND TOXICITY MANAGEMENT 35
7.1. Definitions of Adverse Events and Serious Adverse Events 35
7.1.1. Adverse Events 35
7.1.2. Serious Adverse Events 35
7.2. Assessment of Adverse Events and Serious Adverse Events 36
7.2.1. Assessment of Causality for Study Drugs and Procedures 36
7.2.2. Assessment of Severity 36
7.3. Investigator Requirements and Instructions for Reporting Adverse Events and Serious Adverse Events 37
7.3.1. Requirements for Collection Prior to Study Drug Initiation 37
7.3.2. Adverse Events 37
7.3.3. Serious Adverse Events 37
7.4. Gilead Reporting Requirements 38
7.5. Clinical Laboratory Abnormalities and Other Abnormal Assessments as Adverse Events or Serious Adverse Events 39
7.6. Toxicity Management 39
7.7. Special Situations Reports 39
7.7.1. Definitions of Special Situations 39
7.7.2. Instructions for Reporting Special Situations 40
8. STATISTICAL CONSIDERATIONS 42
8.1. Analysis Objectives and Endpoints 42
8.1.1. Analysis Objectives 42
8.1.2. Primary Endpoint 42
8.1.3. Secondary Endpoint 43
8.1.4. Other Endpoints of Interest 43
8.2. Planned Analyses 44
8.2.1. Interim Analysis 44
8.2.2. Final Analysis 44
8.3. Analysis Conventions 44
8.3.1. Analysis Sets 44
8.3.2. Data Handling Conventions 45
8.4. Demographic and Baseline Characteristics Analysis 45

8.5. Efficacy Analysis ... 45
8.5.1. Primary Analysis ... 45
8.5.2. Secondary Analyses ... 46
8.6. Safety Analysis ... 46
8.6.1. Extent of Exposure ... 46
8.6.2. Adverse Events ... 46
8.6.3. Laboratory Evaluations ... 46
8.7. Adjustments for Multiplicity ... 47
8.8. Pharmacokinetic Analysis ... 47
8.9. Sample Size ... 47
8.9.1. Sample Size Re-estimation ... 48
8.10. Independent Data Monitoring Committee ... 48
9. RESPONSIBILITIES ... 49
9.1. Investigator Responsibilities ... 49
9.1.1. Good Clinical Practice ... 49
9.1.2. Financial Disclosure ... 49
9.1.3. Institutional Review Board/Independent Ethics Committee Review and Approval ... 49
9.1.4. Informed Consent ... 49
9.1.5. Confidentiality ... 50
9.1.6. Study Files and Retention of Records ... 50
9.1.7. Case Report Forms ... 52
9.1.8. Investigator Inspections ... 52
9.1.9. Protocol Compliance ... 52
9.2. Sponsor Responsibilities ... 52
9.2.1. Protocol Modifications ... 52
9.2.2. Study Report and Publications ... 53
9.3. Joint Investigator/Sponsor Responsibilities ... 53
9.3.1. Payment Reporting ... 53
9.3.2. Access to Information for Monitoring ... 53
9.3.3. Access to Information for Auditing or Inspections ... 54
9.3.4. Study Discontinuation ... 54
10. REFERENCES ... 55
11. APPENDICES ... 56

Appendix 1. Investigator Signature Page ... 57
Appendix 2. Study Procedures Table[a] ... 58
Appendix 3. Pregnancy Precautions, Definition for Female of Childbearing Potential, and Contraceptive Requirements ... 60

LIST OF IN-TEXT TABLES

Table 1. Recommended Rate of Infusion—Diluted RDV for Injection Lyophilized Powder in Adults and Pediatric Patients 12 Years of Age and Older and Weighing at Least 40 kg ... 25

LIST OF IN-TEXT FIGURES

Figure 1. Study Schema ... 19

PROTOCOL SYNOPSIS

Gilead Sciences, Inc.
333 Lakeside Drive
Foster City, CA 94404

**Study Title:**	A Phase 3, Randomized Double-Blind Placebo-Controlled Trial to Evaluate the Efficacy and Safety of Remdesivir (GS-5734™) Treatment of COVID-19 in an Outpatient Setting
**IND Number:**	147753
**EudraCT Number:**	2020-003510-12
**Clinical Trials.gov Identifier:**	NCT04501952
**Study Centers Planned:**	Approximately 150 centers globally
**Objectives:**	The purpose of this trial is to evaluate treatment with intravenous (IV) administered remdesivir (RDV, GS-5734) in an outpatient setting in participants with confirmed coronavirus disease 2019 (COVID-19) who are at risk for disease progression.<br>The primary objectives of this study are as follows:<br>• To evaluate the efficacy of RDV in reducing the rate of COVID-19 related hospitalization or all-cause death in non-hospitalized participants with early stage COVID-19<br>• To evaluate the safety of RDV administered in an outpatient setting<br>The secondary objectives of this study are as follows:<br>• To evaluate the efficacy of RDV in reducing the rate of COVID-19 related medically attended visits (MAVs; medical visits attended in person by the participant and a health care professional) or all-cause death in non-hospitalized participants with early stage COVID-19<br>• To determine the antiviral activity of RDV on severe acute respiratory syndrome (SARS)-coronavirus (CoV)-2 viral load<br>• To assess the impact of RDV on symptom duration and severity<br>The exploratory objectives of this study include:<br>• To assess the impact of RDV on other clinical outcomes<br>• To evaluate the emergence of viral resistance to RDV

	• To identify and assess associations of host biomarkers with disease progression and treatment response<br>• To assess the pharmacokinetics (PK) of RDV and its metabolites in patients with COVID-19<br><br>To assess patient-reported outcome using the COVID-19-adapted InFLUenza Patient-Reported Outcome Plus (FLU-PRO Plus©) questionnaire and validate the questionnaire (if available)
**Study Design:**	This is a Phase 3, randomized, double-blind, placebo-controlled, multicenter study of RDV therapy for outpatients with early stage COVID-19 who are at higher risk of disease progression.<br><br>Participants who meet all eligibility criteria may be randomized in a 1:1 ratio to RDV or placebo. Randomization will be stratified by participants who reside in a skilled nursing facility, by participant's age (< 60 vs ≥ 60 years), and by region (United States [US] vs ex-US):<br><br>**Treatment Group A**: single dose of IV RDV 200 mg on Day 1 followed by IV RDV 100 mg on Days 2 and 3 (RDV group)<br><br>**Treatment Group B:** IV placebo-to-match (PTM) RDV on Days 1 to 3 (PTM group)
Number of Participants Planned:	Approximately 1264 participants
Target Population:	Non-hospitalized participants with early stage COVID-19 and at least one risk factor for disease progression.
Duration of Treatment:	Participants will receive study treatment with RDV or PTM for 3 days
Diagnosis and Main Eligibility Criteria:	Participants with COVID-19 who meet the following inclusion criteria may be included:<br><br>• Willing and able to provide written informed consent (age ≥18) or assent (age ≥12 to <18, where locally and nationally approved), or with a legal representative who can provide informed consent (where locally and nationally approved)

	• Either:<br>At least 1 pre-existing risk factor for progression to hospitalization (chronic lung disease, hypertension, cardiovascular or cerebrovascular disease, diabetes, obesity (BMI ≥ 30), immunocompromised, chronic mild or moderate kidney disease, chronic liver disease, current cancer, or sickle cell disease);<br>OR age ≥ 60 years<br>• SARS-CoV-2 infection confirmed by molecular diagnostics (nucleic acid [eg, PCR] or antigen testing) ≤ 4 days prior to screening<br>• Presence of ≥ 1 symptom(s) consistent with COVID-19 for ≤ 7 days prior to randomization (such as fever, cough, fatigue, shortness of breath, sore throat, headache, myalgia/arthralgia)<br>• Not currently receiving, requiring, or expected to require supplemental oxygen<br>• Not currently requiring hospitalization (hospitalization defined as ≥ 24 hours of acute care)<br>Exclusion criteria for participation include:<br>• Participation in any other clinical trial of an experimental treatment and prevention for COVID-19<br>• Prior hospitalization for COVID-19 (hospitalization defined as ≥ 24 hours of acute care)<br>• Treatment with other agents with actual or possible direct antiviral activity against SARS-CoV-2 or administration of any SARS-CoV-2 (or COVID-19) vaccine<br>• Known hypersensitivity to the study drug, metabolites, or formulation excipient
Study Procedures/ Frequency:	At screening, after the participant has provided informed consent, demographic and baseline characteristics and medical history will be collected. Current clinical history, and concomitant medications will be documented. Physical examination findings, vital signs including temperature, respiratory rate, and SpO_2 on room air will be documented. Women of childbearing potential will have a urine pregnancy test.

After screening procedures, eligible participants may be randomized in a 1:1 ratio to receive treatment with RDV or PTM. Randomization will be stratified by residence in a skilled nursing facility, participant's age (<60 vs ≥ 60 years), and region (US vs ex-US).

After randomization, all participants randomized to receive RDV or PTM should receive their initial dose on Day 1.

Nasopharyngeal swabs and sputum samples will be collected on Days 1, 2, 3, 7, and 14 for SARS-CoV-2 quantitative reverse transcriptase polymerase chain reaction viral load testing and possible resistance testing.

On study Days 1 through 3, vital signs including respiratory status will be measured, and adverse events (AEs), medically attended visit information and concomitant medications will be documented. Laboratory tests for safety (hematology, chemistry, and coagulation) will be performed on Days 1, 3, 7, and 14. Optional biomarkers will be performed on Days 1, 3 and 14. On study Day 14 physical examination findings, vital signs including temperature, respiratory rate, and SpO_2, AEs, medically attended visit information and concomitant medications will be documented.

Symptom severity will be assessed daily from Day 1 through Day 14 using the COVID-19-adapted FLU-PRO Plus questionnaire (if available).

On study Day 28, there will be an in-person or phone visit. Physical examination, vital signs including temperature, respiratory rate, and SpO_2, AEs, medically attended visit information and concomitant medications will be documented (only AEs, medically attended visit information and concomitant medications needed if performed by phone).

Sparse PK assessments will be conducted in all participants at participating sites and approximately 30 participants will have intensive PK assessments. At selected sites, sparse PK samples will be collected at study Day 2 (end of infusion, and optional 2-hour post end of infusion), and Day 3 (pre-dose [within 30 minutes of dosing] and end of infusion). Approximately 30 participants will have intensive PK samples collected at study Day 1 and Day 3 at the following time points relative to the start time of infusion: 0 (pre-dose), 0.5, 0.75, 3, 6, 8, 12 (optional) and 24 hours. All blood samples for PK assessments will be drawn from the arm opposite the one used to administer RDV or PTM.

**Test Product, Dose, and Mode of Administration:**	Remdesivir for injection, 100 mg, for IV administration
**Reference Therapy, Dose, and Mode of Administration:**	Placebo-to-Match
**Criteria for Evaluation:**	The primary endpoints of this study are:<br>• Composite endpoint of COVID-19 related hospitalization (defined as at least 24 hours of acute care) or all-cause death by Day 28<br>• Proportion of participants with treatment-emergent AEs<br>The secondary endpoints of this study are:<br>• Composite endpoint of COVID-19 related MAVs (medical visits attended in person by the participant and a health care professional) or all-cause death by Day 28<br>• All-cause mortality at Day 28<br>• Proportion of participants hospitalized by Day 28<br>• Composite endpoint of COVID-19 related hospitalization (defined as at least 24 hours of acute care) or all-cause death by Day 14<br>• Composite endpoint of COVID-19 related MAVs (medical visits attended in person by the participant and a health care professional) or all-cause death by Day 14<br>• Time-weighted average change in SARS-CoV-2 viral load from baseline to Day 7<br>• Time to alleviation (mild or absent) of baseline COVID-19 symptoms as reported on the COVID-19-adapted FLU-PRO Plus<br>• Proportion of participants progressing to requiring oxygen supplementation by Day 28<br>The exploratory endpoints of this study are:<br>• Time to alleviation (mild or absent) of baseline symptoms in each domain of the COVID-19-adapted FLU-PRO Plus<br>• Change from baseline in COVID-19-adapted FLU-PRO Plus total score and score in each domain<br>• Psychometric validity of COVID-19-adapted FLU-PRO Plus questionnaire<br>• Time-weighted average change in SARS-CoV-2 viral load from baseline to Day 14

	• Time to first negative SARS-CoV-2 PCR<br>• Proportion of participants with negative SARS-CoV-2 PCR at each study visit<br>• Emergence of viral resistance to RDV<br>• Baseline levels and change from baseline for inflammation/immune-related, acute respiratory distress syndrome-related and coagulation-related biomarkers<br>• Proportion of participants admitted to the intensive care unit by Day 28<br>• Proportion of participants started on mechanical ventilation by Day 28<br>• The plasma concentrations and PK parameters of RDV and metabolites
**Statistical Methods:**	The total sample size of the study will be approximately 1264 participants.<br>A sample size of approximately 1264 participants (632 in each group with 1:1 randomization) achieves > 90% power to detect a ratio of 0.55 (RDV to placebo in proportion of COVID-19 related hospitalization or all-cause death rate, which is equal to a hazard ratio of 0.534) using a 2-sided significance level of 0.05 assuming the overall hospitalization or death rate is 9.3% (12% in the placebo group and 6.6% in the RDV group) and a 5% drop out rate. The sample size provides approximately 80% power to detect a ratio of 0.60 (RDV to placebo). This analysis will be stratified by residence in skilled nursing facility, age (<60 vs ≥60 years), and region (US vs ex-US). An unblinded sample size re-estimation is planned after 50% participants completed Day 28.<br>The primary endpoint will be analyzed using Cox model with stratification factors as covariates. The null hypothesis being tested is whether the ratio of hospitalization or death is the same for either RDV or placebo treatment. The ratio and 95% confidence interval will be provided.<br>Treatment-emergent AEs and laboratory abnormalities will be summarized using descriptive statistics and listed by participant.

This study will be conducted in accordance with the guidelines of Good Clinical Practice including archiving of essential documents.

GLOSSARY OF ABBREVIATIONS AND DEFINITION OF TERMS

AE	adverse event
ALT	alanine aminotransferase
aPTT	activated partial thromboplastin time
ARDS	acute respiratory distress syndrome
AST	aspartate aminotransferase
ATP	adenosine triphosphate
CI	confidence interval
CL_{cr}	creatinine clearance
CoV	coronavirus
COVID-19	coronavirus disease 2019
CP	conditional power
CRF	case report form
DAIDS	Division of AIDS
DMC	data monitoring committee
eCCGs	eCRF completion guidelines
eCRF	electronic case report form
EDC	electronic data capture
eGFR	estimated glomerular filtration rate
eSAE	electronic serious adverse event
EU	European Union
FDA	Food and Drug Administration
FLU-PRO Plus©	InFLUenza Patient-Reported Outcome Plus
GCP	Good Clinical Practice
Gilead	Gilead Sciences, Inc.
GLPS	Global Patient Safety
HIV	human immunodeficiency virus
IB	investigator’s brochure
ICH	International Council for Harmonisation (of Technical Requirements for Pharmaceuticals for Human Use)
IDMC	independent data monitoring committee
IEC	independent ethics committee
IND	investigational new drug
INR	international normalized ratio
IRB	institutional review board
IV	intravenous
IXRS	interactive voice/web response system
LLOQ	lower limit of quantitation
MAV	medically attended visit
O_2	oxygen

PCR	polymerase chain reaction
PI	principal investigator
PK	pharmacokinetic(s)
PT	prothrombin time
PTM	placebo-to-match
qRT-PCR	quantitative reverse transcriptase polymerase chain reaction
RDV	remdesivir (GS-5734™)
RNA	ribonucleic acid
SAE	serious adverse event
SARS	severe acute respiratory syndrome
SBECD	sulfobutylether β-cyclodextrin sodium
SDV	source data verification
SOC	standard of care
SOP	standard operating procedure
SpO_2	oxygen saturation
SUSAR	suspected unexpected serious adverse reaction
ULN	upper limit of normal
US	United States

1. INTRODUCTION

1.1. Background

Severe acute respiratory syndrome (SARS)-coronavirus (CoV)-2, a single-stranded RNA virus, is identified as the cause of an outbreak of respiratory illness that was first detected in Wuhan, China in December 2019. The virus has now spread globally, resulting in a global pandemic and causing severe respiratory illness throughout the world. Severe cases progress to pneumonia and multi-organ failure, which can lead to death. Gilead Sciences, Inc. (Gilead) has been working with global health authorities to respond to the ongoing pandemic and to evaluate the utility of intravenous (IV) remdesivir (RDV; GS-5734™) as a treatment option for coronavirus disease 2019 (COVID-19) through clinical trials. Remdesivir is approved for the treatment of COVID-19 in the United States (US), European Union (EU), Japan, and other countries for populations including adults and pediatric patients (12 years and older and weighing at least 40 kg).

1.2. Remdesivir (RDV, GS-5734)

Remdesivir is being developed by Gilead and is formulated for IV administration.

1.2.1. General Information

Remdesivir is an adenosine nucleotide prodrug that distributes into cells where it is metabolized to form the pharmacologically active nucleoside triphosphate metabolite. Metabolism of remdesivir to remdesivir triphosphate has been demonstrated in multiple cell types. Remdesivir triphosphate acts as an analog of adenosine triphosphate (ATP) and competes with the natural ATP substrate for incorporation into nascent RNA chains by the SARS-CoV-2 RNA-dependent RNA polymerase, which results in delayed chain termination during replication of the viral RNA.

For further information on RDV, refer to the investigator's brochure (IB) for RDV.

1.3. Rationale for This Study

Remdesivir is a novel antiviral drug that exhibits potent nanomolar antiviral activity against SARS-CoV-2 in primary human airway epithelial cells {Sheahan 2017}. In SARS-CoV-2-infected rhesus monkeys, administration of RDV resulted in a significant reduction in clinical signs of respiratory disease, lung pathology and gross lung lesions, and viral RNA levels compared with vehicle-treated animals {Williamson 2020}. The safety and efficacy of RDV has been demonstrated in clinical studies of patients hospitalized with COVID-19.

The risk of adverse outcomes from COVID-19 increases with age and the presence of chronic health conditions, such as cardiovascular disease, diabetes, and chronic lung disease{Stokes 2020, The Novel Coronavirus Pneumonia Emergency Response Epidemiology Team 2020, World Health Organization (WHO) 2020a}. In acute viral infection with influenza, a short course of antiviral treatment has shown improved clinical outcome {Nicholson 2000}. Treatment intervention in the earlier phase of COVID-19, such as in an outpatient setting, may prevent disease progression to moderate disease requiring hospitalization. The resulting reduction in hospitalizations would significantly benefit patients and would reduce the burden to health care systems.

There is currently no approved treatment for patients with COVID-19 who have not been hospitalized. This study will evaluate the potential for a 3-day treatment with IV RDV to change the clinical trajectory of early COVID-19. The double blinded design will allow stringent evaluation of the safety and efficacy of RDV in this population.

Participants in this study will be treated with RDV for a maximum of 3 days receiving a total of 400 mg RDV. This dose corresponds to a total administration of 12 g of sulfobutylether β-cyclodextrin sodium (SBECD) using the lyophilized powder. Based on the European Medicines Agency (EMA) review summarizing the safety of cyclodextrins as excipients {Committee for Medicinal Products for Human Use (CHMP) 2014}, which indicates approximately 250 mg/kg/day of SBECD (12 g/day based on a 48 kg human) is safe, participants will not be required to discontinue RDV if their creatinine clearance is < 30 mL/min during the dosing period.

1.4. Rationale for Duration of Treatment

In patients with severe COVID-19 who do not require mechanical ventilation, 5 days of RDV showed similar efficacy to a 10 day regimen. Similarly, 5 days treatment of RDV in participants with moderate COVID-19 was associated with a significant improvement in clinical status compared with standard of care (SOC) and approximately a third of participants were discharged prior to completion of 5 days RDV therapy.

In early viral infection, shorter courses of antivirals are often effective in preventing disease progression {Nicholson 2000}. As such, a shorter duration of 3 days of RDV treatment is proposed in participants with early stage COVID-19 not requiring hospitalization or oxygen supplementation with the goal of preventing disease progression.

1.5. Rationale for Dose Selection of Remdesivir

The dosing of RDV in this study, 200 mg on Day 1 and 100 mg on each of Days 2 and 3 is the initial dosing recommended by US Food and Drug Administration (FDA) and approved by EMA for adults and adolescents weighing ≥ 40 kg.

1.6. Risk/Benefit Assessment for the Study

In addition to the established risks associated with IV RDV, potential risks associated with the study include unknown adverse events (AEs) and laboratory abnormalities. There is the potential for resistance to develop with shorter treatment duration.

Intravenous RDV for up to 10 days is approved for the treatment of COVID-19 in the US, Japan, and the EU.

The shorter duration of treatment may lower the risk of some adverse findings associated with RDV, eg, the transient elevations of alanine aminotransferase (ALT) and aspartate aminotransferase (AST). However, other risks may not be ameliorated or may not have been evident in the studies of participants with more severe disease.

An independent data monitoring committee (IDMC) will review safety and efficacy data of the study when 50% of the participants have been enrolled. In addition, a futility interim analysis is planned when approximately 50% of the participants have completed the Day 28 visit to stop a non-efficacious treatment. The IDMC will make a recommendation of stopping enrollment to the study if the pre-specified futility stopping criteria are met.

The risk mitigation strategy for this study includes restriction of the study population to those without a history of significant hepatic or renal disease:

- Exclusion of participants with contraindicated known hepatic or renal disease
- Exclusion of coadministration of other investigational agents against COVID-19
- Serum chemistry assessments, including liver function tests, will be monitored during the study period.

There are currently no investigational agents with demonstrated clinical efficacy or approved treatments for COVID-19 patients not requiring in-patient care. The timely evaluation of a safe and effective antiviral agent with demonstrated safety and efficacy addresses a serious unmet medical need. In consideration of the information included in this protocol, the overall risks to participants are outweighed by the potential benefits of RDV investigational therapy for the treatment of COVID-19. The benefit-risk balance for this study is considered positive.

1.7. Compliance

This study will be conducted in compliance with this protocol, Good Clinical Practice (GCP), and all applicable regulatory requirements.

2. OBJECTIVES

The purpose of this trial is to evaluate treatment with IV administered RDV in an outpatient setting in participants with confirmed COVID-19 who are at higher risk for disease progression.

The primary objectives of this study are as follows:

- To evaluate the efficacy of RDV in reducing the rate of COVID-19 related hospitalization or all-cause death in non-hospitalized participants with early stage COVID-19
- To evaluate the safety of RDV administered in an outpatient setting

The secondary objectives of this study are as follows:

- To evaluate the efficacy of RDV in reducing the rate of COVID-19 related medically attended visits (MAVs; medical visits attended in person by the participant and a health care professional) or all- cause death in non-hospitalized participants with early stage COVID-19
- To determine the antiviral activity of RDV on SARS-CoV-2 viral load
- To assess the impact of RDV on symptom duration and severity

The exploratory objectives of this study include:

- To assess the impact of RDV on other clinical outcomes
- To evaluate the emergence of viral resistance to RDV
- To identify and assess associations of host biomarkers with disease progression and treatment response
- To assess the pharmacokinetics (PK) of RDV and its metabolites in participants with COVID-19
- To assess patient-reported outcome using the COVID-19-adapted InFLUenza Patient-Reported Outcome Plus (FLU-PRO Plus©) questionnaire and validate the questionnaire (if available)

3. STUDY DESIGN

3.1. Endpoints

The primary endpoints of this study are as follows:

- Composite endpoint of COVID-19 related hospitalization (defined as at least 24 hours of acute care) or all-cause death by Day 28
- Proportion of participants with treatment-emergent AEs

The secondary endpoints of this study are as follows:

- Composite endpoint of COVID-19 related MAVs (medical visits attended in person by the participant and a health care professional) or all-cause death by Day 28
- All-cause mortality at Day 28
- Proportion of participants hospitalized by Day 28
- Composite endpoint of COVID-19 related hospitalization (defined as at least 24 hours of acute care) or all-cause death by Day 14
- Composite endpoint of COVID-19 related MAVs (medical visits attended in person by the participant and a health care professional) or all-cause death by Day 14
- Time-weighted average change in SARS-CoV-2 viral load from baseline to Day 7
- Time to alleviation (mild or absent) of baseline COVID-19 symptoms as reported on the COVID-19-adapted FLU-PRO Plus
- Proportion of participants progressing to requiring oxygen supplementation by Day 28

The exploratory endpoints of the study are as follows:

- Time to alleviation (mild or absent) of baseline symptoms in each domain of the COVID-19-adapted FLU-PRO Plus
- Change from baseline in COVID-19-adapted FLU-PRO Plus total score and score in each domain
- Psychometric validity of COVID-19-adapted FLU-PRO Plus questionnaire
- Time-weighted average change in SARS-CoV-2 viral load from baseline to Day 14
- Time to first negative SARS-CoV-2 polymerase chain reaction (PCR)

- Proportion of participants with negative SARS-CoV-2 PCR at each study visit
- Emergence of viral resistance to RDV
- Baseline levels and change from baseline for inflammation/immune-related, acute respiratory distress syndrome (ARDS)-related and coagulation-related biomarkers
- Proportion of participants admitted to the intensive care unit by Day 28
- Proportion of participants started on mechanical ventilation by Day 28
- The plasma concentrations and PK parameters of RDV and metabolites

3.2. Study Design

This is a Phase 3, randomized, double-blind, placebo-controlled, multicenter study of RDV therapy for outpatients with early stage COVID-19 who are at higher risk of disease progression.

3.3. Study Treatments

Approximately 1264 participants who meet all eligibility criteria may be randomized in a 1:1 ratio into either treatment group:

Treatment Group A: single dose of IV RDV 200 mg on Day 1 followed by IV RDV 100 mg on Days 2 and 3 (RDV group)

Treatment Group B: IV placebo-to-match (PTM) RDV on Days 1 to 3 (PTM group)

Randomization will be stratified by participants who reside in a skilled nursing facility, by participant’s age (<60 vs ≥60 years), and by region (US vs ex-US).

3.4. Duration of Treatment

Participants will receive study treatment with RDV for 3 days (Treatment Group A) or PTM for 3 days (Treatment Group B).

Figure 1. Study Schema

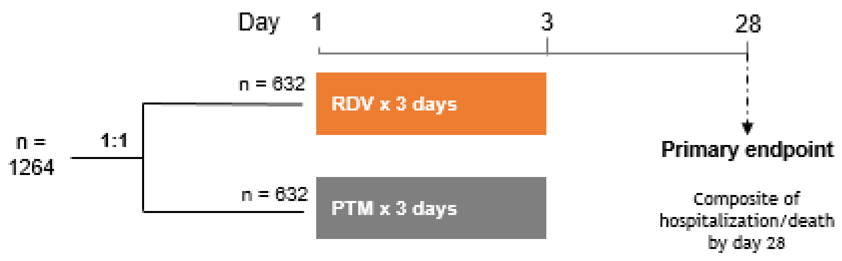

3.5. End of Study

The end of the study will be the last participant's last observation (or visit).

3.6. Poststudy Care

The long-term care of the participant will remain the responsibility of their primary treating physician. Remdesivir is being supplied with curative intent. There is no provision for post-study availability.

3.7. Source Data

The source data for this study will be obtained from electronic data capture (EDC), central laboratory, local laboratory, specialty laboratory (for PK and/or pharmacodynamic data), and/or interactive voice/web response system (IXRS) data.

3.8. Biomarker Testing

3.8.1. Biomarker Samples for Optional Future Research

In addition to the study-specific informed consent, participants will be required to document whether they agree to have additional samples collected and for the remainder of their already collected PK specimens for optional future research to be used, in accordance with applicable regulations.

The collection of additional samples and use of the remainder of their already collected specimens for optional future research may be used to advance development of the drug and/or increase our knowledge and understanding of the biology of the disease under investigation and related diseases. These specimens may also be used to study the association of biomarkers with biological pathways, disease pathogenesis, progression and/or treatment outcomes, including efficacy, AEs, and the processes of drug absorption and disposition. In addition, these specimens may be used to develop biomarker, and/or diagnostic assays and establish the performance characteristics of these assays. The collection and analysis of optional future research specimens

may facilitate the design of new pharmaceutical agents and the development of diagnostic tests, which may allow for individualized drug therapy for patients in the future.

Serum and plasma samples will be collected to measure biomarkers which may include but not limited to biomarkers of inflammation, ARDS, and coagulopathy in COVID 19 at Days 1, 3, and 14.

The specimens collected for optional future research will be destroyed no later than 15 years after the end of study or per country requirements.

3.8.2. Biomarker Samples for Optional Genomic Research

In addition to the study-specific informed consent to be signed by each participant in the study, participants will be required to document whether they agree to provide additional samples for optional genomic research. Additional samples will be obtained from participants who agree to participate and provide their additional specific consent. These samples should be collected pre-dose at the Baseline/Day 1 visit but may be collected at any time during the study or at a separate post study visit, if necessary.

The specimens collected for optional future genomic research may be used to advance the development of the drug and/or increase our knowledge and understanding of the biology of the disease under investigation, or related diseases. These specimens may also be used to study the association of biomarkers with biological pathways, disease pathogenesis, progression and/or treatment outcomes, including efficacy, AEs, and the processes of drug absorption and disposition. In addition, these specimens may be used to develop biomarker and/or diagnostic assays and establish the performance characteristics of these assays. The collection and analysis of optional genomic research specimens may facilitate the design of new pharmaceutical agents and the development of diagnostic tests, which may allow for individualized drug therapy for patients in the future.

The samples collected for optional genomic research will be destroyed no later than 15 years after the end of the study or per country requirements.

4. PARTICIPANT POPULATION

4.1. Number of Participants and Participant Selection

Approximately 1264 participants who meet the eligibility criteria will be enrolled in one of 2 treatment groups. Participants will receive study drug in an outpatient, skilled nursing facility or home health setting.

4.1.1. Participant Replacement

Participants who discontinue before the end of study will not be replaced.

4.2. Inclusion Criteria

Participants must meet all of the following inclusion criteria to be eligible for participation in this study:

1) Willing and able to provide written informed consent (participants ≥ 18 years of age) or assent (participants ≥ 12 and < 18 years of age) prior to performing study procedures. Participants aged ≥ 18 years may be enrolled with the consent of a legal representative where permitted according to local law and approved nationally and by the relevant institutional review board (IRB) or independent ethics committee (IEC). For participants ≥ 12 and < 18 years of age, a parent or legal guardian must be willing and able to provide written informed consent prior to performing study procedures

2) Either:

   Age ≥ 18 years (at all sites) or aged ≥ 12 and < 18 years of age weighing ≥ 40 kg (where permitted according to local law and approved nationally and by the relevant IRB or IEC) with at least 1 of the following pre-existing risk factors for progression to hospitalization:

   a) Chronic lung disease: chronic obstructive pulmonary disease, moderate-to-severe asthma, cystic fibrosis, pulmonary fibrosis

   b) Hypertension: systemic or pulmonary

   c) Cardiovascular or cerebrovascular disease: coronary artery disease, congenital heart disease, heart failure, cardiomyopathy, history of stroke, atrial fibrillation, hyperlipidemia

   d) Diabetes mellitus: Type 1, type 2, or gestational

   e) Obesity (BMI ≥ 30)

   f) Immunocompromised state; having a solid organ transplant, blood, or bone marrow transplant; immune deficiencies; HIV with a low CD4 cell count or not on HIV treatment; prolonged use of corticosteroids; or use of other immune weakening medicines

   g) Chronic mild or moderate kidney disease

   h) Chronic liver disease

i) Current cancer

j) Sickle cell disease

OR age ≥ 60 years, regardless of the presence of other pre-existing risk factors for progression

3) SARS-CoV-2 infection confirmed by molecular diagnostics (nucleic acid [eg, PCR] or antigen testing) ≤ 4 days prior to screening
4) Presence of ≥ 1 symptom(s) consistent with COVID-19 for ≤ 7 days prior to randomization (such as fever, cough, fatigue, shortness of breath, sore throat, headache, myalgia/arthralgia) {Stokes 2020, World Health Organization (WHO) 2020b}
5) Not currently receiving, requiring, or expected to require supplemental oxygen
6) Not currently requiring hospitalization (hospitalization defined as ≥ 24 hours of acute care)
7) Participants of childbearing potential who engage in heterosexual intercourse must agree to use protocol-specified method(s) of contraception as described in Appendix 3

4.3. Exclusion Criteria

Participants who meet *any* of the following exclusion criteria are not eligible to be enrolled in this study:

1) Participation in any other clinical trial of an experimental treatment and prevention for COVID-19
2) Prior hospitalization for COVID-19 (hospitalization defined as ≥ 24 hours of acute care)
3) Treatment with other agents with actual or possible direct antiviral activity against SARS-CoV-2 or administration of any SARS-CoV-2 (or COVID-19) vaccine
4) Criterion removed
5) Requiring oxygen supplementation
6) ALT or AST ≥ 5 × upper limit of normal (ULN) at screening or within 90 days of screening

Note: if per local practice only ALT is routinely measured, exclusion criteria will be evaluated on ALT alone

7) Creatinine clearance < 30 mL/min at screening or within 90 days of screening using the Cockcroft-Gault formula in participants ≥ 18 years of age or estimated glomerular filtration rate (eGFR) < 30 mL/min/1.73m^2 at screening or within 90 days of screening using the Schwartz formula in participants < 18 years of age (see Section 6.7.2)
8) Currently breastfeeding (nursing)
9) Known hypersensitivity to the study drug, the metabolites, or formulation excipient
10) Use or planned use of exclusionary medications, refer to Section 5.4

5. INVESTIGATIONAL MEDICINAL PRODUCTS

5.1. Randomization, Blinding, and Treatment Codes Access

5.1.1. Randomization

Participants who meet all randomization eligibility criteria will be randomized in a 1:1 ratio to Treatment Group A or Treatment Group B and assigned a participant number. Randomization will be stratified by residence in a skilled nursing facility, age (<60 vs ≥ 60), and region (US vs ex-US).

Randomization may occur approximately one day prior to the Day 1 visit.

5.1.2. Blinding

During the randomized phase participants and all personnel directly involved in the conduct of the study will be blinded to treatment assignment. Specified personnel may be unblinded based on their study role. Study drug will be dispensed by the study pharmacist, or designee, in a blinded fashion to the participants. The Pharmacokinetics File Administrator, or designee, in Bioanalytical Operations and/or Clinical Data Management, who facilitates the data transfer of PK files between Gilead and vendors, will remain unblinded. Individuals in clinical virology performing sample selection for resistance analysis may be unblinded. Individuals in Clinical Packaging and Labeling or Clinical Supply Management who have an Unblinded Inventory Manager role in the IXRS system for purposes of study drug inventory management will remain unblinded. Individuals in Global Patient Safety (GLPS) responsible for safety signal detection, investigational new drug (IND) safety reporting, and/or expedited reporting of suspected unexpected serious adverse reactions (SUSARs) may be unblinded to individual case data and/or group level summaries. External (ie, contract research organizations) biostatisticians and programmers will be unblinded for the IDMC, IND safety reporting. Regulatory Quality and Compliance personnel in Research and Development may also be unblinded for purposes of supporting Quality Assurance activities and/or Regulatory Agency inspections.

5.1.3. Procedures for Breaking Treatment Codes

In the event of a medical emergency where breaking the blind is required to provide medical care to the participant, the investigator may obtain treatment assignment directly from the IXRS system for that participant. Gilead recommends but does not require that the investigator contact the Gilead medical monitor before breaking the blind. Treatment assignment should remain blinded unless that knowledge is necessary to determine participant emergency medical care. The rationale for unblinding must be clearly explained in source documentation and on the electronic case report form (eCRF), along with the date on which the treatment assignment was obtained. The investigator is requested to contact the Gilead medical monitor promptly in case of any treatment unblinding.

Blinding of study treatment is critical to the integrity of this clinical study. Therefore, if a participant's treatment assignment is disclosed to the investigator, the participant will have study treatment discontinued. All participants will be followed until study completion unless consent to do so is specifically withdrawn by the participant.

5.2. Description and Handling of Remdesivir

5.2.1. Formulation

Remdesivir for injection, 100 mg, is a preservative-free, white to off-white to yellow, lyophilized solid containing 100 mg of RDV that is to be reconstituted with sterile water for injection and diluted into 0.9% saline prior to administration by IV infusion.

In addition to the active ingredient, RDV for injection, 100 mg, contains the following inactive ingredients: SBECD, water for injection, hydrochloric acid, and sodium hydroxide. Hydrochloric acid and sodium hydroxide are used to adjust the formulation to a pH of 3.0 to 4.0.

The supplied PTM RDV for injection, 100 mg, is identical in physical appearance to the active formulation and contains the same inactive ingredients.

5.2.2. Packaging and Labeling

Remdesivir for injection and PTM, 100 mg, is supplied as a sterile product in a single-use, 30-mL Type I clear glass vial. Each vial is sealed with a rubber stopper and an aluminum over seal with a red, plastic flip-off cap. Following reconstitution, each single-use vial contains sufficient volume to allow withdrawal of 20 mL (100 mg RDV or PTM).

Remdesivir for injection and PTM, 100 mg, shall be labeled to meet all applicable requirements of the US FDA, EU Guideline to Good Manufacturing Practice Annex 13 (Investigational Medicinal Products), the J-GCP (Ministerial Ordinance on Good Clinical Practice for Drugs), as applicable, and/or other local regulations.

5.2.3. Storage and Handling

Remdesivir for injection and PTM, 100 mg, vials should be stored below 30 °C (86 °F) prior to use. Storage conditions are specified on the label. Until dispensed for dosing, all vials of study drugs should be stored in a securely locked area, accessible only to authorized site personnel.

To ensure the sterility, stability, and proper identification, study drug(s) should not be stored in a container other than the container in which they were supplied.

Measures that minimize drug contact with the body should always be considered during handling, preparation, and disposal procedures.

The total storage time of reconstituted solution containing RDV or placebo should not exceed 24 hours at room temperature (20 °C to 25 °C) or 48 hours at refrigerated temperature (2 °C to 8 °C). Any unused reconstituted solution containing RDV or placebo should be discarded.

5.3. Dosage and Administration of Remdesivir

Remdesivir for injection, 100 mg, or PTM will be provided by Gilead.

Participants in Treatment Group A will receive IV RDV 200 mg on Day 1 followed by IV RDV 100 mg on Days 2 and 3. Participants in the Treatment Group B will receive IV PTM on Days 1 to 3.

5.3.1. Administration Instructions

The prepared diluted solution should not be administered simultaneously with any other medication. The compatibility of RDV injection with IV solutions and medications other than 0.9% sodium chloride is not known. Administer RDV via IV infusion over 30 minutes. Slower infusion rates of up to 120 minutes can be considered to potentially prevent signs and symptoms of infusion related reaction. Infusion rates for different infusion volumes and times are described in Table 1.

Table 1. Recommended Rate of Infusion—Diluted RDV for Injection Lyophilized Powder in Adults and Pediatric Patients 12 Years of Age and Older and Weighing at Least 40 kg

Infusion bag volume	Infusion time	Rate of infusion
250 mL	30 min	8.33 mL/min
	60 min	4.17 mL/min
	120 min	2.08 mL/min
100 mL	30 min	3.33 mL/min
	60 min	1.67 mL/min
	120 min	0.83 mL/min

5.3.2. Infusion-related Reactions

Infusion-related reactions have been observed during and following administration of RDV. Signs and symptoms may include hypotension, hypertension, tachycardia, bradycardia, hypoxia, fever, dyspnea, wheezing, angioedema, rash, nausea, diaphoresis, and shivering. Slower infusion rates, with a maximum infusion time of up to 120 minutes, can be considered to potentially prevent these signs and symptoms. If signs and symptoms of a severe infusion-related reaction occur, immediately discontinue administration of RDV and initiate appropriate treatment. Please refer to Section 7.6.

5.4. Prior and Concomitant Medications

Concomitant medications taken within 30 days prior to screening and up to and including 30 days after the last dose of study drug need to be recorded in the source documents and eCRFs.

Concomitant use of the following is prohibited in participants receiving RDV:

- Investigational or approved agents for the SARS-CoV-2 virus including approved HIV protease inhibitors such as lopinavir/ritonavir, interferon, etc. Use of these medications for an approved indication other than SARS-CoV-2 infection is not prohibited
- Use of hydroxychloroquine or chloroquine for any indication
- Strong inducers of P-glycoprotein (eg, rifampin or herbal medications)

5.5. Accountability for Investigational Medicinal Product

The investigator is responsible for ensuring adequate accountability of all used and unused study drug vials. This includes acknowledgment of receipt of each shipment of study drug (quantity and condition).

Each study site must keep accountability records that capture:

- The date received and quantity of study drug vials.
- The date, participant number, and the study drug vial number dispensed.
- The date, quantity of used and unused study drug vials returned, along with the initials of the person recording the information.

5.5.1. Investigational Medicinal Product Return or Disposal

Gilead recommends that used and unused study drug supplies be destroyed at the site. If the site has an appropriate standard operating procedure (SOP) for drug destruction as determined by Gilead, the site may destroy used (empty or partially empty) and unused study drug supplies in accordance with that site's approved SOP. A copy of the site's approved SOP will be obtained for eTMF. If study drug is destroyed on site, the investigator must maintain accurate records for all study drugs destroyed. Records must show the identification and quantity of each unit destroyed, the method of destruction, and the person who disposed of the study drug. Upon study completion, copies of the study drug accountability records must be filed at the site. Another copy will be returned to Gilead.

If the site does not have an appropriate SOP for drug destruction, used and unused study drug supplies are to be sent to the designated disposal facility for destruction. The study monitor will provide instructions for return.

The study monitor will review study drug supplies and associated records at periodic intervals.

For both disposal options listed above, the study monitor must first perform drug accountability during an on-site or remote monitoring visit.

6. STUDY PROCEDURES

The study procedures to be conducted for each participant enrolled in the study are presented in tabular form in Appendix 2 and described in the text that follows.

The investigator must document any deviation from the protocol procedures and notify Gilead or the contract research organization.

6.1. Participant Enrollment and Treatment Assignment

Entry into screening does not guarantee enrollment into the study. In order to manage the total study enrollment, Gilead, at its sole discretion, may suspend screening and/or enrollment at any site or study-wide at any time. Study visits may be performed in an outpatient setting, at the participant's home via tele-health, virtually or remotely, as permitted by local and institutional regulations.

6.2. Pretreatment Assessments

6.2.1. Screening Visit

Participants will be screened within 2 days before randomization to determine eligibility for participation in the study. The following will be performed and documented at screening:

- Obtain written informed consent
- Obtain medical history including the following information: date of first symptoms, overall symptoms, exposure source, demographics, baseline characteristics, allergies and all other medical history
- Review and document prior and concomitant medications
- Complete physical examination including, vital signs (heart rate, temperature, blood pressure), body weight, and height.
- Respiratory status:

    Respiratory rate

    Oxygenation: SpO_2 on room air

- Obtain ALT (and AST where available), serum creatinine, and creatinine clearance/eGFR (calculated using Cockcroft-Gault or Schwartz formula; see Section 6.7.2) if not available within 90 days of screening, using a local laboratory.
- Women of childbearing potential will have a urine pregnancy test performed

- Documentation of SARS-CoV-2 infection confirmed by molecular diagnostics (nucleic acid [eg, PCR] or antigen testing) ≤ 4 days prior to screening
- Confirmation of presence of COVID-19 symptoms
- Record any SAEs and all AEs related to protocol-mandated procedures occurring after signing of the consent form

Participants meeting all of the inclusion criteria and none of the exclusion criteria may be enrolled the same day as screening for randomization into the study.

From the time of obtaining informed consent through the first administration of study drug, record all SAEs, as well as any AEs related to protocol-mandated procedures on the Adverse Events eCRF. All other untoward medical occurrences observed during the screening period, including exacerbation or changes in medical history are to be considered medical history. See Section 7 Adverse Events and Toxicity Management for additional details.

6.3. Treatment Assessments (Baseline/Day 1, Day 2, and Day 3)

The following evaluations are to be completed at the Day 1 visit. The investigator must have confirmed eligibility before proceeding with randomization and the Day 1 visit. If the screening and Day 1 visits occur on the same day, no procedures need to be repeated except for clinical laboratory samples that must be collected locally to confirm eligibility. Clinical laboratory samples must be collected at Day 1 to be sent to the central laboratory, even if completed locally, as described in Section 6.7.2. Participants must complete the following assessments before being administered study drug:

- Vital signs (heart rate, temperature, blood pressure) pre-infusion, postinfusion, and when postinfusion observation is completed
- Respiratory status:

  Respiratory rate

  Oxygenation: SpO_2 on room air
- Complete physical examination (Day 1)
- Clinical symptom-directed physical examination (Day 2, Day 3)
- Review of AEs and concomitant medications
- Review any interactions with health care professionals, other than study staff or designees, including hospitalization; in-person emergency, urgent, or primary care visits; or any other in-person visit attended by the participant and a health care profession. Identify the nature and cause of the visit.

- Obtain blood samples as described in Section 6.7.2 (Day 1, Day 3)
- Nasopharyngeal swab samples for SARS-CoV-2 quantitative reverse transcriptase PCR (RT-qPCR) testing and possible viral resistance testing
- Sputum sample collection from participants with productive cough for SARS-CoV-2 RT-qPCR testing and possible viral resistance testing
- Completion of the COVID-19-adapted FLU-PRO Plus questionnaire (if available)
- Administration of study drug. Instructions on study drug administration are available in a separate manual.

Remdesivir infusions will be administered to participants at the site under close supervision or in the participant's home by a home health service provider. Health care professionals administering RDV infusions will have the appropriate medication available for immediate use in case of hypersensitivity or infusion-related reactions. The participant should be treated according to the SOC for management of hypersensitivity reaction or infusion-related reactions. Postinfusion monitoring should be done according to site or home health protocol. All information related to home administration of RDV will be provided to the investigator by the home health provider, wherever applicable, in a timely manner.

6.4. Post-treatment Assessments (Day 4 Through Day 14)

The following evaluations are to be completed at the specified study days. The study visits are to be completed on the protocol-specified visit date (based on the Day 1 visit). The Day 7 and Day 14 visits have a ± 1 day window.

- Vital signs (heart rate, temperature, blood pressure), body weight (Day 7, Day 14)
- Review of AEs and concomitant medications (Day 7, Day 14)
- Review MAV information, including any interactions with health care professionals other than study staff or designees including hospitalization; in-person emergency, urgent, or primary care visits; or any other in-person visit attended by the participant and a health care profession. Identify the nature and cause of the visit.
- Complete physical examination (Day 14).
- Clinical symptom-directed physical examination (Day 7).
- Respiratory status (Day 7, Day 14):

  Respiratory rate

  Oxygenation: SpO_2 on room air

- Obtain blood samples as described in Section 6.7.2 (Day 7, Day 14)
- Nasopharyngeal swab samples for SARS-CoV-2 RT-qPCR testing and possible viral resistance testing (Day 7, Day 14)
- Sputum sample collection from participants with productive cough for SARS-CoV-2 RT-qPCR testing and possible viral resistance testing (Day 7, Day 14)
- Completion of the COVID-19-adapted FLU-PRO Plus questionnaire (if available) (Days 4, 5, 6, 7, 8, 9, 10, 11, 12, 13, 14)

6.5. Day 28 Follow-up Assessment (± 5 Days)

The following evaluations are to be completed if the visit is conducted in person. The final evaluation can be performed by phone (only AEs, concomitant medications review, and MAV information needed if done by phone).

- Clinical symptom-directed physical examination, vital signs (heart rate, temperature, blood pressure), and body weight.
- Respiratory status:

  Respiratory rate

  Oxygenation: SpO_2 on room air
- Review of AEs and concomitant medications
- Review MAV information, including any interactions with health care professionals, other than study staff or designees, including hospitalization; in-person emergency, urgent, or primary care visits; or any other in-person visit attended by the participant and a health care profession. Identify the nature and cause of the visit.

6.6. Assessments for Early Discontinuation from Study

If a participant discontinues study dosing (for example, as a result of an AE), every attempt should be made to keep the participant in the study and continue to perform the required study-related follow-up and procedures (see Section 6.8, Criteria for Discontinuation of Study Treatment). If this is not possible or acceptable to the participant or investigator, the participant may be withdrawn from the study.

6.7. Procedures and Specifications

6.7.1. Complete Physical Examination

A complete physical examination must include source documentation of general appearance and the following body systems: Head, neck, and thyroid; eyes, ears, nose, throat, mouth, and tongue; chest (excluding breasts); respiratory; cardiovascular; lymph nodes, abdomen; skin, hair, nails; musculoskeletal; and neurological. Urogenital and reproductive examination should only be completed if clinically indicated.

6.7.2. Clinical Laboratory Assessments

Blood sample collection for the following laboratory analyses will be performed at the specified time points:

- Chemistry profile: alkaline phosphatase, AST, ALT, total bilirubin, total protein, albumin, lactate dehydrogenase, creatine phosphokinase, bicarbonate, blood urea nitrogen, calcium, chloride, creatinine, glucose, lipase, magnesium, phosphorus, potassium, sodium, uric acid (Day 1, Day 3, Day 7, Day 14)
- Estimated glomerular filtration rate (screening [if required], Day 1, Day 3, Day 7, Day 14) according to:

    Schwartz formula for participants < 18 years of age, where S_{Cr} is serum creatinine (mg/dL)

Adolescent boys ≥ 12 years of age: $0.70 \times L/ S_{Cr}$ (L is height in cm)

Adolescent girls ≥ 12 years of age: $0.55 \times L/ S_{Cr}$ (L is height in cm)

Height at baseline will be used for all calculations using the Schwartz formula.

    Cockcroft-Gault formula for creatinine clearance (CL_{cr}) for participants ≥ 18 years of age. Weight at screening will be used for Days 1-3.

Men:

$$\frac{(140 \quad \text{age in years}) \times (\text{wt in kg})}{72 \times (\text{serum creatinine in mg/dL})} \quad CL_{cr}\ (\text{mL/min})$$

Women:

$$\frac{(140 \quad \text{age in years}) \times (\text{wt in kg}) \times 0.85}{72 \times (\text{serum creatinine in mg/dL})} \quad CL_{cr}\ (\text{mL/min})$$

- Hematology profile: complete blood count with differential (Day 1, Day 3, Day 7, Day 14)
- Coagulation: international normalized ratio (INR), prothrombin time (PT), activated partial thromboplastin time (aPTT) (Day 1, Day 3, Day 7, Day 14)
- Optional genomic testing (Day 1 or at any time during the study, if additional consent for genomics testing is obtained)

6.7.3. Pharmacokinetic Assessments

All blood samples for PK assessments will be drawn from the arm opposite the one used to administer RDV or PTM. Sparse PK assessments will be conducted in all participants at participating sites and approximately 30 participants will have intensive PK assessments.

- Sparse PK assessments

    Day 2: End of infusion and optional 2 hours post end of infusion

    Day 3: Pre-dose (within 30 minutes of dosing) and end of infusion

- Intensive PK assessments

    Day 1 and Day 3: at the following time points relative to the start time of infusion:
    0 (pre-dose), 0.5, 0.75, 3, 6, 8, 12 (optional) and 24 hours

6.7.4. Biomarker Assessments (Optional)

Optional blood samples will be collected to measure serum and plasma biomarkers and may include immune-related markers, ARDS-related, and coagulation-related biomarkers. DNA extraction for genetic analysis may be performed to evaluate single nucleotide polymorphisms which may be associated with host viral response, immune response, coagulation-related disorders and respiratory disorders.

- Serum (Days 1, 3, 14)

    Immune-related biomarkers (IL-6, CRP, ferritin, procalcitonin)

    ARDS-related (ang2)

- Plasma (Days 1, 3, 14)

    Abnormal coagulation-related biomarkers (D-dimer, fibrinogen)

6.7.5. Virology Assessments

Nasopharyngeal swab and sputum samples will be used to assess SARS-CoV-2 viral load by RT-qPCR. Once viral load testing is complete, the remnant samples may be used to evaluate the emergence of viral resistance by SARS-CoV-2 sequencing and/or phenotypic testing according to the Virology Analysis Plan.

6.8. Criteria for Discontinuation of Study Treatment

Study medication will be discontinued for individual participants in the following instances, unless the potential for resuming dosing is specifically noted:

- Intercurrent illness that would, in the judgment of the investigator, affect assessments of clinical status to a significant degree. Following resolution of intercurrent illness, the participant may resume study dosing at the discretion of the investigator.
- Unacceptable toxicity, or toxicity that, in the judgment of the investigator, compromises the ability to continue study-specific procedures or is considered to not be in the participant's best interest
- ALT or AST ≥ 5 × ULN; or ALT > 3 × ULN and total bilirubin > 2 × ULN, confirmed by immediate repeat testing
- Other Grade 3 or Grade 4 abnormal laboratory results related to RDV
- Lack of efficacy
- Participant request to discontinue for any reason
- Participant noncompliance
- Discontinuation of the study at the request of Gilead, a regulatory agency or an IRB or IEC
- Infusion-related systemic reaction ≥ Grade 2 or infusion-related localized reaction ≥ Grade 3

6.9. End of Study

The end of the study will be the last participant's last observation (or visit).

6.10. Poststudy Care

The long-term care of the participant will remain the responsibility of their primary treating physician. Remdesivir is being supplied with curative intent. There is no provision for post-study availability.

6.11. Sample Storage

The stored biological samples may be used by Gilead or its research partner for future testing to provide additional data to answer questions that relate to the main study. At the end of this study, these samples may be retained in storage by Gilead for a period up to 15 years. If participants provide additional specific consent, residual PK samples may be destroyed no later than 15 years after the end of the study or per country requirements.

Any remaining specimens from nasopharyngeal swab and sputum samples collected during the study will be stored and retained for possible future virology-related testing. These stored samples may be used by the sponsor or its research partners for viral genotyping/phenotyping assays or their development, for retesting the amount of virus present in the sample, or for testing to learn more about how the study drug has worked or clinical laboratory testing to provide additional safety data. At the conclusion of this study, these samples may be retained in storage by Gilead for a period up to 15 years.

7. ADVERSE EVENTS AND TOXICITY MANAGEMENT

7.1. Definitions of Adverse Events and Serious Adverse Events

7.1.1. Adverse Events

An AE is any untoward medical occurrence in a clinical study participant administered an investigational product, which does not necessarily have a causal relationship with the treatment. An AE can therefore be any unfavorable and/or unintended sign, symptom, or disease temporally associated with the use of an investigational product, whether or not the AE is considered related to the investigational product. AEs may also include pre- or post-treatment complications that occur as a result of protocol-specified procedures or special situations (Section 7.7).

An AE does not include the following:

- Medical or surgical procedures such as surgery, endoscopy, tooth extraction, and transfusion. The condition that led to the procedure may be an AE and must be reported.
- Preexisting diseases, conditions, or laboratory abnormalities present or detected before the screening visit that do not worsen
- Situations where an untoward medical occurrence has not occurred (eg, hospitalization for elective surgery, social and/or convenience admissions)
- Overdose without clinical sequelae (Section 7.7.1)
- Any medical condition or clinically significant laboratory abnormality with an onset date before the consent form is signed and not related to a protocol-associated procedure is not an AE. It is considered to be preexisting and should be documented as medical history.
- Preexisting events or conditions that increase in severity or change in nature after the consent form is signed or as a consequence of participation in the clinical study will be considered AEs.

7.1.2. Serious Adverse Events

A SAE is defined as an event that, at any dose, results in the following:

- Death
- Life-threatening (Note: The term “life-threatening” in the definition of “serious” refers to an event in which the participant was at risk of death at the time of the event; it does not refer to an event that hypothetically might have caused death if it were more severe.)
- In-patient hospitalization or prolongation of existing hospitalization
- Persistent or significant disability/incapacity

- A congenital anomaly/birth defect
- A medically important event or reaction: such events may not be immediately life-threatening or result in death or hospitalization but may jeopardize the participant or may require intervention to prevent one of the other outcomes constituting SAEs. Medical and scientific judgment must be exercised to determine whether such an event is a reportable under expedited reporting rules. Examples of medically important events include intensive treatment in an emergency room or at home for allergic bronchospasm; blood dyscrasias or convulsions that do not result in hospitalization; and development of drug dependency or drug abuse.

7.2. Assessment of Adverse Events and Serious Adverse Events

The investigator or qualified subinvestigator is responsible for assessing AEs and SAEs for causality and severity, and for final review and confirmation of accuracy of event information and assessments.

7.2.1. Assessment of Causality for Study Drugs and Procedures

The investigator or qualified subinvestigator is responsible for assessing the relationship to study drug using clinical judgment and the following considerations:

- **No**: Evidence exists that the AE has an etiology other than the study drug. For SAEs, an alternative causality must be provided (eg, preexisting condition, underlying disease, intercurrent illness, or concomitant medication).
- **Yes**: There is reasonable possibility that the event may have been caused by the study drug.

It should be emphasized that ineffective treatment should not be considered as causally related in the context of AE reporting.

The relationship to study procedures (eg, invasive procedures such as venipuncture or biopsy) should be assessed using the following considerations:

- **No:** Evidence exists that the AE has an etiology other than the study procedure.
- **Yes:** The AE occurred as a result of protocol procedures (eg, venipuncture).

7.2.2. Assessment of Severity

The severity of AEs will be graded using the Division of AIDS (DAIDS) Table for Grading the Severity of Adult and Pediatric Adverse Events, Version 2.1 dated July 2017. For each episode, the highest grade attained should be reported as defined in the grading scale. The DAIDS scale is available at the following location:

https://rsc.niaid.nih.gov/sites/default/files/daidsgradingcorrectedv21.pdf

7.3. Investigator Requirements and Instructions for Reporting Adverse Events and Serious Adverse Events

7.3.1. Requirements for Collection Prior to Study Drug Initiation

After informed consent, but prior to initiation of study medication, the following types of events must be reported on the applicable eCRFs: all SAEs and AEs related to protocol-mandated procedures.

7.3.2. Adverse Events

Following initiation of study medication, collect all AEs, regardless of cause or relationship, throughout the duration of the study, including the protocol-defined follow-up period, must be reported on the eCRFs as instructed.

All AEs and clinically significant laboratory abnormalities will be followed until resolution or stability of the abnormality has been demonstrated, if possible. Gilead may request that certain AEs be followed beyond the protocol-defined follow-up period.

7.3.3. Serious Adverse Events

All SAEs, regardless of cause or relationship, that occur after the participant first consents to participate in the study (i.e., signing the informed consent) and throughout the duration of the study, including the post-treatment follow-up visit, must be reported on the applicable eCRFs and to GLPS as instructed below in this section. This also includes any SAEs resulting from protocol-associated procedures performed after informed consent is signed.

Any SAEs and deaths that occur after the post-treatment follow-up visit but within 30 days of the last dose of study drug, regardless of causality, should also be reported.

Investigators are not obligated to actively seek SAEs after the protocol-defined follow-up period; however, if the investigator learns of any SAEs that occur after the protocol-defined follow-up period has concluded and the event is deemed relevant to the use of study drug, the investigator should promptly document and report the event to Gilead GLPS.

- All SAEs will be recorded in the case report form (CRF)/eCRF database within 24 hours.

7.3.3.1. Electronic Serious Adverse Event Reporting Process

- Site personnel record all SAE data on the applicable eCRFs and from there transmit the SAE information to Gilead GLPS within 24 hours of the investigator’s knowledge of the event. Detailed instructions can be found in the eCRF completion guidelines (eCCGs).

- If it is not possible to record and transmit the SAE information electronically, record the SAE on the paper SAE reporting form and submit within 24 hours to:

  Gilead GLPS
  Email: Safety_fc@gilead.com
  or
  Fax: 1-650-522-5477

- As soon as it is possible to do so, any SAE reported via paper must be transcribed on the applicable eCRFs according to instructions and within the timelines outlined in the eCCGs.

- If an SAE has been reported via a paper form because the eCRF database has been locked, no further action is necessary.

- For fatal or life-threatening events, copies of hospital case reports, autopsy reports, and other documents are also to be submitted by email or fax when requested and applicable. Transmission of such documents should occur without personal participant identification, maintaining the traceability of a document to the participant identifiers.

- Additional information may be requested to ensure the timely completion of accurate safety reports.

- Any medications necessary for treatment of the SAE must be recorded onto the concomitant medication section of the participant's eCRF and the SAE narrative section of the Safety Report Form eCRF.

7.4. Gilead Reporting Requirements

Depending on relevant local legislation or regulations, including the applicable US FDA Code of Federal Regulations, the EU Clinical Trials Directive (2001/20/EC) and relevant updates, and other country-specific legislation or regulations, Gilead may be required to expedite to worldwide regulatory agencies reports of SAEs, serious adverse drug reactions, or SUSARs. In accordance with the EU Clinical Trials Directive (2001/20/EC), Gilead or a specified designee will notify worldwide regulatory agencies and the relevant IEC in concerned Member States of applicable SUSARs as outlined in current regulations.

Assessment of expectedness for SAEs will be determined by Gilead using reference safety information specified in the IB or relevant local label as applicable.

All investigators will receive a safety letter notifying them of relevant SUSAR reports associated with any study drug. The investigator should notify the IRB or IEC of SUSAR reports as soon as is practical, where this is required by local regulatory agencies, and in accordance with the local institutional policy.

7.5. Clinical Laboratory Abnormalities and Other Abnormal Assessments as Adverse Events or Serious Adverse Events

Laboratory abnormalities without clinical significance are not recorded as AEs or SAEs. However, laboratory abnormalities (eg, clinical chemistry, hematology, and coagulation) that require medical or surgical intervention or lead to study drug interruption, modification, or discontinuation must be recorded as an AE, as well as an SAE, if applicable. In addition, laboratory or other abnormal assessments (eg, electrocardiogram, x-rays, vital signs) that are associated with signs and/or symptoms must be recorded as an AE or SAE if they meet the definition of an AE or SAE as described in Sections 7.1.1 and 7.1.2. If the laboratory abnormality is part of a syndrome, record the syndrome or diagnosis (eg, anemia), not the laboratory result (i.e., decreased hemoglobin).

Severity should be recorded and graded according to the DAIDS Table for Grading the Severity of Adult and Pediatric Adverse Events, Version 2.1 dated July 2017. For AEs associated with laboratory abnormalities, the event should be graded on the basis of the clinical severity in the context of the underlying conditions; this may or may not be in agreement with the grading of the laboratory abnormality.

7.6. Toxicity Management

Remdesivir infusions will be administered to participants at the site under close supervision or in the participant's home by a home health service provider. Health care professionals administering RDV infusions will have the appropriate medication available for immediate use in case of hypersensitivity or infusion-related reactions. The participant should be treated according to the SOC for management of hypersensitivity reaction or infusion-related reactions. Postinfusion monitoring should be done according to site or home health protocol. All information related to home administration of RDV will be provided to the investigator by the home health provider, wherever applicable, in a timely manner.

7.7. Special Situations Reports

7.7.1. Definitions of Special Situations

Special situation reports include all reports of medication error, abuse, misuse, overdose, occupational exposure, drug interactions, exposure via breastfeeding, unexpected benefit, transmission of infectious agents via the product, counterfeit of falsified medicine, and pregnancy regardless of an associated AE.

Medication error is any unintentional error in the prescribing, dispensing, preparation for administration or administration of an investigational product while the medication is in the control of a health care professional, patient, or consumer. Medication errors may be classified as a medication error without an AE, which includes situations of missed dose; medication error with an AE; intercepted medication error; or potential medication error.

Abuse is defined as persistent or sporadic intentional excessive use of an investigational product by a participant.

Misuse is defined as any intentional and inappropriate use of an investigational product that is not in accordance with the protocol instructions or the local prescribing information.

An overdose is defined as an accidental or intentional administration of a quantity of an investigational product given per administration or cumulatively which is above the maximum recommended dose as per protocol or in the product labeling (as it applies to the daily dose of the participant in question). In cases of a discrepancy in drug accountability, overdose will be established only when it is clear that the participant has taken the excess dose(s). Overdose cannot be established when the participant cannot account for the discrepancy except in cases in which the investigator has reason to suspect that the participant has taken the additional dose(s).

Occupational exposure is defined as exposure to an investigational product as a result of one's professional or non-professional occupation.

Drug interaction is defined as any drug/drug, drug/food, or drug/device interaction.

Unexpected benefit is defined as an unintended therapeutic effect where the results are judged to be desirable and beneficial.

Transmission of infectious agents is defined as any suspected transmission of an infected agent through a Gilead investigational product.

Counterfeit or falsified medicine: Any investigational product with a false representation of: a) its identity, b) its source, or c) its history.

7.7.2. Instructions for Reporting Special Situations

7.7.2.1. Instructions for Reporting Pregnancies

The investigator should report pregnancies in female study participants that are identified after initiation of study drug and throughout the study, including the post study drug follow-up period, to Gilead GLPS using the pregnancy report form within 24 hours of becoming aware of the pregnancy.

Refer to Section 7.3 for GLPS contact information and the eCCGs for full instructions on the mechanism of pregnancy reporting.

The pregnancy itself is not considered an AE nor is an induced elective abortion to terminate a pregnancy without medical reasons.

Any premature termination of pregnancy (eg, a spontaneous abortion, an induced therapeutic abortion due to complications or other medical reasons) must be reported within 24 hours as an SAE. The underlying medical reason for this procedure should be recorded as the AE term.

A spontaneous abortion is always considered to be an SAE and will be reported as described in Section 7.3.2. Furthermore, any SAE occurring as an adverse pregnancy outcome post study must be reported to Gilead GLPS.

The participant should receive appropriate monitoring and care until the conclusion of the pregnancy. The outcome should be reported to Gilead GLPS using the pregnancy outcome report form. If the end of the pregnancy occurs after the study has been completed, the outcome should be reported directly to Gilead GLPS. Gilead GLPS contact information is as follows: email: Safety_FC@gilead.com and fax: +1 (650) 522-5477.

Pregnancies of female partners of male study participants exposed to Gilead or other study drugs must also be reported and relevant information should be submitted to Gilead GLPS using the pregnancy and pregnancy outcome forms within 24 hours. If the end of the pregnancy occurs after the study has been completed, the outcome should be reported directly to Gilead GLPS, fax number +1-650-522-5477 or email Safety_FC@gilead.com.

Refer to Appendix 3 Pregnancy Precautions, Definition for Female of Childbearing Potential, and Contraceptive Requirements.

7.7.2.2. Reporting Other Special Situations

All other special situation reports must be reported on the special situations report form and forwarded to Gilead GLPS within 24 hours of the investigator becoming aware of the situation. These reports must consist of situations that involve study drug and/or Gilead concomitant medications, but do not apply to non-Gilead concomitant medications.

Special situations involving non-Gilead concomitant medications do not need to be reported on the special situations report form; however, special situations that result in AEs due to a non-Gilead concomitant medication, must be reported as an AE.

Any inappropriate use of concomitant medications prohibited by this protocol should not be reported as "misuse," but may be more appropriately documented as a protocol deviation.

Refer to Section 7.3 for GLPS contact information and the eCCGs for instructions on special situation reporting.

All clinical sequelae in relation to these special situation reports will be reported as AEs or SAEs at the same time using the AE eCRF and/or the SAE report form. Details of the symptoms and signs, clinical management, and outcome will be reported, when available.

8. STATISTICAL CONSIDERATIONS

8.1. Analysis Objectives and Endpoints

8.1.1. Analysis Objectives

The primary objectives of this study are as follows:

- To evaluate the efficacy of RDV in reducing the rate of COVID-19 related hospitalization or all-cause death when given over 3 days to non-hospitalized participants with early stage COVID-19
- To evaluate the safety of RDV administered in an outpatient setting

The secondary objectives of this study are as follows:

- To evaluate the efficacy of RDV in reducing the rate of COVID-19 related MAVs (medical visits attended in person by the participant and a health care professional) or all-cause death in non-hospitalized participants with early stage COVID-19. An MAV includes in-person clinic visit, urgent care visit, emergency room visit, or hospitalization
- To determine the antiviral activity of RDV on SARS-CoV-2 viral load
- To assess the impact of RDV on symptom duration and severity

The exploratory objectives of this study are as follows:

- To assess the impact of RDV on other clinical outcomes
- To evaluate the emergence of viral resistance to RDV
- To identify and assess associations of host biomarkers with disease progression and treatment response
- To assess the PK of RDV and its metabolites in participants with COVID-19
- To assess patient-reported outcome using the COVID-19-adapted FLU-PRO Plus questionnaire and validate the questionnaire (if available)

8.1.2. Primary Endpoint

The primary endpoints of this study are as follows:

- Composite endpoint of COVID-19 related hospitalization (defined as at least 24 hours of acute care) or all-cause death by Day 28
- Proportion of participants with treatment-emergent AEs

8.1.3. Secondary Endpoint

The secondary endpoints of this study are as follows:

- Composite endpoint of COVID-19 related MAVs (medical visits attended in person by the participant and a health care professional) or all cause death by Day 28
- All-cause mortality at Day 28
- Proportion of participants hospitalized by Day 28
- Composite endpoint of COVID-19 related hospitalization (defined as at least 24 hours of acute care) or all-cause death by Day 14
- Composite endpoint of COVID-19 related (medical visits attended in person by the participant and a health care professional) or all cause death by Day 14
- Time-weighted average change in SARS-CoV-2 viral load from baseline to Day 7
- Time to alleviation (mild or absent) of baseline COVID-19 symptoms as reported on the COVID-19-adapted FLU-PRO Plus
- Proportion of participants progressing to requiring oxygen supplementation by Day 28

8.1.4. Other Endpoints of Interest

Other endpoints of interest are as follows:

- Time to alleviation (mild or absent) of baseline symptoms in each domain of the COVID-19-adapted FLU-PRO Plus
- Change from baseline in COVID-19-adapted FLU-PRO Plus total score and score in each domain
- Psychometric validity of COVID-19-adapted FLU-PRO Plus questionnaire
- Time-weighted average change in SARS-CoV-2 viral load from baseline to Day 14
- Time to first negative SARS-CoV-2 PCR
- Proportion of participants with negative SARS-CoV-2 PCR at each study visit
- Emergence of viral resistance to RDV
- Baseline levels and change from baseline for inflammation/immune-related, ARDS-related and coagulation-related biomarkers

- Proportion of participants admitted to the intensive care unit by Day 28
- Proportion of participants started on mechanical ventilation by Day 28
- The plasma concentrations and PK parameters of RDV and metabolites

8.2. Planned Analyses

8.2.1. Interim Analysis

Prior to the final analysis, interim analyses may be conducted and the analyses may be submitted to regulatory agencies to seek guidance for the overall clinical development program m.

8.2.1.1. Data Monitoring Committee Analysis

An external data monitoring committee (DMC) will review safety data on a regular basis. One futility interim analysis is planned after approximately 50% of participants complete Day 28. Enrollment may pause for the IDMC review if enrollment is faster than projected. The DMC will review the interim safety data and summary of primary efficacy data by treatment group and make recommendation of stopping enrollment to a treatment due to lack of efficacy and decision on sample size re-estimation.

8.2.2. Final Analysis

The final analysis will be performed after all participants have completed the study, outstanding data queries have been resolved or adjudicated as unresolvable, and the data have been cleaned and finalized. The analysis of the primary endpoint of COVID-19 related hospitalization or all-cause death by Day 28 will be conducted at the time of the final analysis and will be tested at the 0.05 significance level.

8.3. Analysis Conventions

8.3.1. Analysis Sets

8.3.1.1. Efficacy

The primary analysis set for efficacy analysis is defined as the Full Analysis Set, which will include all participants who (1) are randomized into the study and (2) have received at least 1 dose of study treatment. Participants will be grouped according to the treatment to which they were randomized.

8.3.1.2. Safety

The primary analysis set for safety analyses is defined as the Safety Analysis Set, which will include all participants who (1) are randomized into the study and (2) have received at least 1 dose of study treatment. Participants will be grouped according to the treatment to which they received.

8.3.1.3. Pharmacokinetics

The PK Analysis Set will include all randomized participants who received at least 1 dose of RDV and had at least 1 non-missing PK concentration datum reported by the PK laboratory for each respective analyte.

8.3.2. Data Handling Conventions

Natural logarithm transformation for PK parameters will be applied for PK analysis.

For summary statistics, PK concentration values below the limit of quantitation will be treated as zero at pre-dose and one-half of the lower limit of quantitation (LLOQ) for post dose time points

Laboratory data that are continuous in nature but are less than the LLOQ or above the upper limit of quantitation, will be imputed to the value of the lower or upper limit plus or minus 1 significant digit, respectively (eg, if the result of a continuous laboratory test is < 20, a value of 19 will be assigned).

Missing data can have an impact upon the interpretation of the trial data. In general, values for missing data will not be imputed. However, a missing pre-treatment laboratory result would be treated as normal (i.e., no toxicity grade) for the laboratory abnormality summary.

All available data for participants that do not complete the study will be included in data listings

8.4. Demographic and Baseline Characteristics Analysis

Demographic and baseline measurements will be summarized using standard descriptive methods. Demographic summaries will include sex, race/ethnicity, randomization stratification group, and age. For categorical demographic and baseline characteristics, a Cochran-Mantel-Haenszel test will be used to compare treatment groups. For continuous demographic and baseline characteristics, a Wilcoxon rank sum test will be used to compare treatment groups.

8.5. Efficacy Analysis

8.5.1. Primary Analysis

The primary endpoint of the study is the composite endpoint of COVID-19 related hospitalization or all-cause death by Day 28. The null hypothesis being tested is whether the ratio of proportion of COVID-19 related hospitalization or all-cause death is the same for the 2 treatment groups (ie, whether ratio is equal to 1 across all strata).

The hazard ratio, p-value, and 95% CI for the hazard ratio will be calculated using a Cox model with stratification factors as covariates.

Participants with missing outcomes for the primary endpoint due to prematurely discontinuation of the study will be censored at the date of last contact.

8.5.2. Secondary Analyses

The secondary endpoint of COVID-19 related hospitalization or all-cause death by Day 14, COVID-19 related MAVs (medical visits attended in person by the participant and a health care professional) or death by Day 28, and COVID-19 related MAVs (medical visits attended in person by the participant and a health care professional) or death by Day 14 will be analyzed similarly as in the primary analysis. The all-cause mortality at Day 28 will be compared between the 2 treatment groups using a Fisher exact test. The proportion of participants hospitalized by Day 28 and the proportion of participants progressing to requiring oxygen supplementation by Day 28 will be estimated using the Kaplan-Meier method and compared between the 2 treatment groups using a log-rank test. Other endpoints of interest related to proportion of participants will be compared between treatment groups using a chi-square test or Fisher exact test. Endpoints that are measured as time to first event will be compared between treatment groups using the log-rank test and continuous endpoints will be compared between treatment groups using a Wilcoxon rank sum test or analysis of variance model.

8.6. Safety Analysis

All safety data collected on or after the randomization date through the Day 28 visit will be summarized by treatment group (according to the study drug received). Data for the pretreatment will be included in data listings.

8.6.1. Extent of Exposure

A participant's extent of exposure to study drug data will be generated from the study drug administration data. Exposure data will be summarized by treatment group.

8.6.2. Adverse Events

Clinical and laboratory AEs will be coded using the Medical Dictionary for Regulatory Activities. System Organ Class, High-Level Group Term, High-Level Term, Preferred Term, and Lower-Level Term will be attached to the clinical database.

Events will be summarized on the basis of the date of onset for the event. A treatment-emergent AE will be defined as any AE that begins on or after the randomization date up to the date of last dose of study drug plus 30 days.

Summaries (number and percentage of participants) of treatment-emergent AEs (by system organ class and preferred term) will be provided by treatment group.

8.6.3. Laboratory Evaluations

Selected laboratory data will be summarized using only observed data. Data and change from baseline at all scheduled time points will be summarized.

Graded laboratory abnormalities will be defined using the grading scheme in DAIDS Table for Grading the Severity of Adult and Pediatric Adverse Events, Version 2.1 dated July 2017.

Incidence of treatment-emergent laboratory abnormalities, defined as values that increase at least 1 toxicity grade from baseline at any time post baseline up to the end date of the study, will be summarized by treatment group. If baseline data are missing, then any graded abnormality (ie, at least a Grade 1) will be considered treatment-emergent.

Laboratory abnormalities that occur before the first dose of study drug or after the participant has been discontinued from treatment for more than 30 days will be included in a data listing.

8.7. Adjustments for Multiplicity

No adjustments for multiple comparisons are needed for the primary analysis of this study.

8.8. Pharmacokinetic Analysis

Plasma concentrations and PK parameters for RDV and metabolites will be listed and summarized using descriptive statistics by treatment.

8.9. Sample Size

The total sample size of the study will be approximately 1264 participants.

The sample size computation is based on proportions of COVID-19 related hospitalization or all-cause death by Day 28 for the RDV and placebo treatment groups. The sample size needed for a 1:1 randomization using a 2-tailed test at level α is given by:

$$\frac{(z_{1-\alpha/2} + z_{1-\beta})^2}{P(1 - R^2)\sigma^2 B^2}$$

Where P is the overall rate of COVID-19 related hospitalization or all-cause death, B is the log hazard ratio, σ is the standard deviation of treatment indicator variable, R is the R-Squared that is obtained when treatment indicator variable is regressed on the other covariates, and $z_{1\ \alpha/2}$ and $z_{1\ \beta}$ are the 1-α/2 and 1-β quantiles of the standard normal distribution.

A sample size of 1264 participants (632 in each group with 1:1 randomization) achieves > 90% power to detect a ratio of 0.55 (RDV to placebo in proportion of COVID-19 related hospitalization or all-cause death, which is equal to a hazard ratio of 0.534) using a 2-sided significance level of 0.05 assuming the overall hospitalization or death rate is 9.3% (12% in the placebo group and 6.6% in the RDV group) and a 5% drop out rate. The sample size provides approximately 80% power to detect smaller treatment effect size with a ratio of 0.60 (RDV to placebo), assuming a 2-sided significance level of 0.05 and the overall hospitalization or death rate is 9.6% (12% in the placebo group and 7.2% in the RDV group) and a 5% drop out rate.

The sample size calculation was done using software PASS (Version 14.0, module of Cox regression for survival).

8.9.1. Sample Size Re-estimation

A sample size re-estimation is planned at the interim analysis with approximately 50% participants having completed Day 28 visit due to the uncertainty in hospitalization or death rate in the placebo arm. All possible interim results will be partitioned into 3 zones Favorable, Promising, and Unfavorable, depending on the size of observed conditional power (CP) under the current trend at the interim. If the CP falls into the Favorable zone, the trial will continue with the originally planned sample size of 1264 patients. If the CP falls into the Promising zone, the total sample size will be increased (up to 1500) according to the algorithm specified in the adaptation plan. If the CP falls into the Unfavorable zone but the interim result has not met the futility boundary, the trial will also continue with the originally planned sample size of 1264 patients.

The DMC will review the unblinded data at the interim and communicate the decision on sample size to the study team. The overall type I error is controlled at 0.05 using the method proposed by {Muller 2004}. Further details will be included in the DMC charter and an adaptation plan.

8.10. Independent Data Monitoring Committee

An external multidisciplinary IDMC will review the progress of the study, perform interim reviews of safety data on a regular basis and provide recommendation(s) to Gilead whether the nature, frequency, and severity of AEs associated with study treatment warrant the early termination of the study in the best interests of the participants, whether the study should continue as planned, or the study should continue with modifications. The IDMC will review the interim futility analysis and make a recommendation of stopping enrollment to the study if the pre-specified futility stopping criteria are met.

The IDMC may also provide recommendations as needed regarding study design.

The IDMC's specific activities will be defined by a mutually agreed charter, which will define the IDMC's membership, conduct and meeting schedule

While the IDMC will be asked to advise Gilead regarding future conduct of the study, including possible early study termination, Gilead retains final decision-making authority on all aspects of the study.

9. RESPONSIBILITIES

9.1. Investigator Responsibilities

9.1.1. Good Clinical Practice

The investigator will ensure that this study is conducted in accordance with International Council for Harmonisation (of Technical Requirements for Pharmaceuticals for Human Use) (ICH) E6(R2) addendum to its guideline for GCP and applicable laws and regulations.

9.1.2. Financial Disclosure

The investigator and subinvestigators will provide prompt and accurate documentation of their financial interest or arrangements with Gilead, or proprietary interests in the investigational drug during the course of a clinical study. This documentation must be provided prior to the investigator's (and any subinvestigator's) participation in the study. The investigator and subinvestigator agree to notify Gilead of any change in reportable interests during the study and for 1 year following completion of the study. Study completion is defined as the date when the last participant completes the protocol-defined activities.

9.1.3. Institutional Review Board/Independent Ethics Committee Review and Approval

The investigator (or Gilead as appropriate according to local regulations) will submit this protocol, informed consent form, and any accompanying material to be provided to the participant (such as advertisements, participant information sheets, or descriptions of the study used to obtain informed consent) to an IRB/IEC. The investigator will not begin any study participant activities until approval from the IRB/IEC has been documented and provided as a letter to the investigator.

Before implementation, the investigator will submit to and receive documented approval from the IRB/IEC any modifications made to the protocol or any accompanying material to be provided to the participant after initial IRB/IEC approval, with the exception of those necessary to reduce immediate risk to study participants.

9.1.4. Informed Consent

The investigator is responsible for obtaining written informed consent from each individual participating in this study after adequate explanation of the aims, methods, objectives, and potential hazards of the study before undertaking any study-related procedures. The investigator must use the most current IRB- or IEC-approved consent form for documenting written informed consent. Each informed consent (or assent as applicable) will be appropriately signed and dated by the participant or the participant's legally authorized representative and the person conducting the consent discussion, and also by an impartial witness if required by IRB or IEC or local requirements.

The consent form will inform participants about genomic testing and/or planned sample retention. In addition to the study-specific informed consent to be signed by each participant in the study, participants will be required to document agreement to provide additional samples or to allow the use of the remainder of their already collected specimens for optional future research, in accordance with applicable regulations. In addition to the study-specific informed consent to be signed by each participant in the study, participants will be required to document agreement to provide additional samples for optional genomic research. The results of the tests done on the samples will not be given to the participant or the investigator.

9.1.5. Confidentiality

The investigator must assure that participants' anonymity will be strictly maintained and that their identities are protected from unauthorized parties. Only an identification code and any other unique identifier(s) as allowed by local law (such as year of birth) will be recorded on any form or biological sample submitted to Gilead, IRB or IEC, or the laboratory. Laboratory specimens must be labeled in such a way as to protect participant identity while allowing the results to be recorded to the proper participant. Refer to specific laboratory instructions. NOTE: The investigator must keep a screening log with details for all participants screened and enrolled in the study, in accordance with the site procedures and regulations. Participant data will be processed in accordance with all applicable regulations.

The investigator agrees that all information received from Gilead, including but not limited to the IB, this protocol, CRF/eCRF, the study drug, and any other study information, remain the sole and exclusive property of Gilead during the conduct of the study and thereafter. This information is not to be disclosed to any third party (except employees or agents directly involved in the conduct of the study or as required by law) without prior written consent from Gilead. The investigator further agrees to take all reasonable precautions to prevent the disclosure by any employee or agent of the study site to any third party or otherwise into the public domain.

9.1.6. Study Files and Retention of Records

The investigator must maintain adequate and accurate records to enable the conduct of the study to be fully documented and the study data to be subsequently verified. These documents should be classified into at least the following 2 categories: (1) investigator's study file, and (2) participant clinical source documents.

The investigator's study file will contain the protocol/amendments, electronic completed participant CRFs, IRB or IEC and governmental approval with correspondence, informed consent form(s), drug records, staff curriculum vitae and authorization forms, and other appropriate documents and correspondence.

The required source data should include sequential notes containing at least the following information for each participant:

- Participant identification
- Documentation that participant meets eligibility criteria, i.e., medical history, physical examination, and confirmation of diagnosis (to support inclusion and exclusion criteria)
- Documentation of the reason(s) a consented participant is not enrolled
- Participation in study (including study number)
- Study discussed and date of informed consent
- Dates of all visits
- Documentation that protocol-specific procedures were performed
- Results of efficacy parameters, as required by the protocol
- Start and end date (including dose regimen) of study drug, including dates of dispensing and return
- Record of all AEs and other safety parameters (start and end date, and including causality and severity), and documentation that adequate medical care has been provided for any AE
- Concomitant medication (including start and end date, dose if relevant; dose changes)
- Date of study completion and reason for early discontinuation, if it occurs

All clinical study documents must be retained by the investigator until at least 2 years or according to local laws, whichever is longer, after the last approval of a marketing application in an ICH region (i.e., US, Europe, or Japan) and until there are no pending or planned marketing applications in an ICH region; or, if no application is filed or if the application is not approved for such indication, until 2 years after the investigation is discontinued and regulatory authorities have been notified. Investigators may be required to retain documents longer if specified by regulatory requirements, by local regulations, or by an agreement with Gilead. The investigator must notify Gilead before destroying any clinical study records.

Should the investigator wish to assign the study records to another party or move them to another location, Gilead must be notified in advance.

If the investigator cannot provide for this archiving requirement at the study site for any or all of the documents, special arrangements must be made between the investigator and Gilead to store these records securely away from the site so that they can be returned sealed to the investigator in case of an inspection. When source documents are required for the continued care of the participant, appropriate copies should be made for storage away from the site.

9.1.7. Case Report Forms

For each participant consented, an eCRF casebook will be completed by an authorized study staff member whose training for this function is completed in the EDC system. The eCRF casebook will only capture the data required per the protocol schedule of events and procedures. The Inclusion/Exclusion Criteria and Enrollment eCRFs should be completed only after all data related to eligibility have been received. Data entry should be performed in accordance with the eCCGs provided by the sponsor. Subsequent to data entry, a study monitor will perform source data verification (SDV) within the EDC system. System-generated or manual queries will be issued in the EDC system as data discrepancies are identified by the monitor or Gilead staff, who routinely review the data for completeness, correctness, and consistency. The site investigator or site coordinator or other designee is responsible for responding to the queries in a timely manner, within the system, either by confirming the data as correct or updating the original entry, and providing the reason for the update (eg, data entry error). Original entries as well as any changes to data fields will be stored in the audit trail of the system. At a minimum, prior to any interim time points or database lock (as instructed by Gilead), the investigator will use his/her log in credentials to confirm that the forms have been reviewed, and that the entries accurately reflect the information in the source documents. At the conclusion of the study, Gilead will provide the site investigator with a read-only archive copy of the data entered by that site. This archive must be stored in accordance with the records retention requirements outlined in Section 9.1.6.

9.1.8. Investigator Inspections

The investigator will make available all source documents and other records for this study to Gilead's appointed study monitors, to IRBs/IECs, or to regulatory authority or health authority inspectors.

9.1.9. Protocol Compliance

The investigator is responsible for ensuring the study is conducted in accordance with the procedures and evaluations described in this protocol.

9.2. Sponsor Responsibilities

9.2.1. Protocol Modifications

Protocol modifications, except those intended to reduce immediate risk to study participants, may be made only by Gilead. The investigator must submit all protocol modifications to the IRB/IEC in accordance with local requirements and receive documented IRB/IEC approval before modifications can be implemented.

9.2.2. Study Report and Publications

A clinical study report will be prepared and provided to the regulatory agency. Gilead will ensure that the report meets the standards set out in the ICH Guideline for Structure and Content of Clinical Study Reports (ICH E3). Note that an abbreviated report may be prepared in certain cases.

Investigators in this study may communicate, orally present, or publish in scientific journals or other scholarly media only after the following conditions have been met:

The results of the study in their entirety have been publicly disclosed by or with the consent of Gilead in an abstract, manuscript, or presentation form or the study has been completed at all study sites for at least 2 years

The investigator will submit to Gilead any proposed publication or presentation along with the respective scientific journal or presentation forum at least 30 days before submission of the publication or presentation.

No such communication, presentation, or publication will include Gilead's confidential information (see Section 9.1.5).

The investigator will comply with Gilead's request to delete references to its confidential information (other than the study results) in any paper or presentation and agrees to withhold publication or presentation for an additional 60 days in order to obtain patent protection if deemed necessary.

9.3. Joint Investigator/Sponsor Responsibilities

9.3.1. Payment Reporting

Investigators and their study staff may be asked to provide services performed under this protocol, eg, attendance at Investigator Meetings. If required under the applicable statutory and regulatory requirements, Gilead will capture and disclose to Federal and State agencies any expenses paid or reimbursed for such services, including any clinical study payments, meal, travel expenses or reimbursements, consulting fees, and any other transfer of value.

9.3.2. Access to Information for Monitoring

The monitor is responsible for routine review of the CRF/eCRF at regular intervals throughout the study to verify adherence to the protocol and the completeness, consistency, and accuracy of the data being entered on them. The monitor should have access to any participant records needed to verify the entries in the CRF/eCRF. The investigator agrees to cooperate with the monitor to ensure that any problems detected through any type of monitoring (central, on site) are resolved.

9.3.3. Access to Information for Auditing or Inspections

Representatives of regulatory authorities or of Gilead may conduct inspections or audits of the clinical study. If the investigator is notified of an inspection by a regulatory authority the investigator agrees to notify the Gilead medical monitor immediately. The investigator agrees to provide to representatives of a regulatory agency or Gilead access to records, facilities, and personnel for the effective conduct of any inspection or audit.

9.3.4. Study Discontinuation

Both Gilead and the investigator reserve the right to terminate the study at any time. Should this be necessary, both parties will arrange discontinuation procedures and notify the participants, appropriate regulatory authority(ies), IRBs, and IECs. In terminating the study, Gilead and the investigator will assure that adequate consideration is given to the protection of the participants' interests.

10. REFERENCES

Committee for Medicinal Products for Human Use (CHMP). Background review for cyclodextrins used as excipients in the context of the revision of the guideline on 'Excipients in the label and package leaflet of medicinal products for human use' (CPMP/463/00 Rev. 1). Draft report published in support to the propylene glycol Q&A document. For information only. European Medicines Agency (EMA) 20 November, 2014.

Muller HH, Schafer H. A general statistical principle for changing a design any time during the course of a trial. Stat Med 2004;23 (16):2497-508.

Nicholson KG, Aoki FY, Osterhaus AD, Trottier S, Carewicz O, Mercier CH, et al. Efficacy and safety of oseltamivir in treatment of acute influenza: a randomised controlled trial. Neuraminidase Inhibitor Flu Treatment Investigator Group. Lancet 2000;355 (9218):1845-50.

Sheahan TP, Sims AC, Graham RL, Menachery VD, Gralinski LE, Case JB, et al. Broad-Spectrum Antiviral GS-5734 Inhibits Both Epidemic and Zoonotic Coronaviruses. Science translational medicine 2017;9 (396):eaal3653.

Stokes EK, Zambrano LD, Anderson KN, Marder EP, Raz KM, El Burai Felix S, et al. Coronavirus Disease 2019 Case Surveillance - United States, January 22-May 30, 2020. MMWR. Morbidity and mortality weekly report 2020;69 (24):759-65.

The Novel Coronavirus Pneumonia Emergency Response Epidemiology Team. The Epidemiological Characteristics of an Outbreak of 2019 Novel Coronavirus Diseases (COVID-19) China, 2020. Chinese Center for Disease Control and Prevention 2020;2 (8):113-22.

Williamson BN, Feldmann F, Schwarz B, Meade-White K, Porter DP, Schulz J, et al. Clinical benefit of remdesivir in rhesus macaques infected with SARS-CoV-2. Nature 2020.

World Health Organization (WHO). Coronavirus Disease (COVID-2019) Situation Reports. Available at: https://www.who.int/emergencies/diseases/novel-coronavirus-2019/situation-reports. Accessed: 18 March. 2020a:

World Health Organization (WHO). Report of the WHO-China Joint Mission on Coronavirus Disease 2019 (COVID-19). 16-24 February. 2020b.

11. APPENDICES

Appendix 1. Investigator Signature Page
Appendix 2. Study Procedures Table[a]
Appendix 3. Pregnancy Precautions, Definition for Female of Childbearing Potential, and Contraceptive Requirements

Appendix 1. Investigator Signature Page

GILEAD SCIENCES, INC.
333 LAKESIDE DRIVE
FOSTER CITY, CA 94404

STUDY ACKNOWLEDGMENT

A Phase 3 Randomized, Double-Blind Placebo-Controlled Trial to Evaluate the Efficacy and Safety of Remdesivir (GS-5734™) Treatment of COVID-19 in an Outpatient Setting

GS-US-540-9012, Amendment 4, 15 January 2021

This protocol has been approved by Gilead Sciences, Inc. The following signature documents this approval.

PPD

Senior Director, Clinical Research

PPD

14 Jan 2021

Date

INVESTIGATOR STATEMENT

I have read the protocol, including all appendices, and I agree that it contains all necessary details for me and my staff to conduct this study as described. I will conduct this study as outlined herein and will make a reasonable effort to complete the study within the time designated.

I will provide all study personnel under my supervision copies of the protocol and access to all information provided by Gilead Sciences, Inc. I will discuss this material with them to ensure that they are fully informed about the drugs and the study.

Principal Investigator Name (Printed)

Signature

Date

Site Number

Appendix 2. Study Procedures Table[a]

	Screening	Baseline/ Day 1	Day 2	Day 3	Day 7 (± 1 day)	Day 14 (± 1 day)	Day 28[t] Follow-up (± 5 days)
Written Informed Consent	X						
Medical History[b]	X						
Complete Physical Examination[c]	X	X				X	
Clinical Symptom Directed Physical Examination[d]			X	X	X		X
Height	X						
Vital Signs[e] and Weight	X	X[f]	X[f]	X[f]	X	X	X
Respiratory Status[g]	X	X	X	X	X	X	X
ALT, AST, Serum Creatinine, and Creatinine Clearance/eGFR[h]	X						
Chemistry[i], Hematology[j], and Coagulation[k] Panels		X		X	X	X	
Optional Genomic Testing[l]		X					
Urine Pregnancy Test[m]	X						
SARS CoV 2 RT qPCR Testing and Potential Resistance Testing[n]		X	X	X	X	X	
Documentation of SARS CoV 2 Infection	X						
Biomarker Sample Collection[o]		X		X		X	
Sparse PK[p]			X	X			
Intensive PK[q]		X		X			
Medically Attended Visit Information[r]		X	X	X	X	X	X
FLU PRO Plus Questionnaire[s]		X	X	X	X	X	
Study Drug Dosing		X	X	X			
Adverse Events and Concomitant Medications	X	X	X	X	X	X	X

a Study visits may be performed in an outpatient setting, at the participant's home via tele health, virtually or remotely, as permitted by local and institutional regulations. The Day 28 visit may be performed via a phone call.

b Medical history will include the date of first symptoms, overall symptoms, exposure source, demographics, baseline characteristics, allergies and all other medical history.

c A complete physical examination must include source documentation of general appearance and the following body systems: Head, neck, and thyroid; eyes, ears, nose, throat, mouth, and tongue; chest (excluding breasts); respiratory; cardiovascular; lymph nodes, abdomen; skin, hair, nails; musculoskeletal; and neurological. Urogenital and reproductive examination should only be completed if clinically indicated.

d Clinical symptom directed physical examination will include at least cardiac and respiratory evaluation.
e Vital signs include heart rate, temperature, and blood pressure.
f On Days 1, 2 and 3, vital signs to be completed pre infusion, postinfusion, and when postinfusion observation is completed; weight from Screening may be used at Days 1 3.
g Respiratory status includes respiratory rate and SpO_2 on room air.
h Obtain ALT (and AST where available), serum creatinine, and creatinine clearance/eGFR (calculated using Cockcroft Gault or Schwartz formula; see Section 6.7.2) if not available within 90 days of screening, using a local laboratory.
i Chemistry: alkaline phosphatase, AST, ALT, total bilirubin, total protein, albumin, lactate dehydrogenase, creatine phosphokinase, bicarbonate, blood urea nitrogen, calcium, chloride, creatinine, glucose, lipase, magnesium, phosphorus, potassium, sodium, uric acid.
j Hematology: Complete blood count with differential.
k Coagulation: INR, PT, aPTT
l Optional genomic testing can occur at Day 1. If not collected at Day 1, it can be collected at subsequent visits.
m Urine pregnancy test will only be done for women of childbearing potential.
n Nasopharyngeal swab and sputum samples will be collected and stored for SARS CoV 2 RT qPCR and potential resistance testing.
o Optional biomarkers: Serum and whole blood will be collected for participants that consent.
p Sparse PK will be collected from participants at participating sites at Day 2: end of infusion and optional 2 hours post end of infusion and Day 3: Pre dose (within 30 minutes of dosing) and end of infusion.
q Intensive PK will be collected at selected sites and approximately 30 participants will have intensive PK assessments conducted.
r Medically attended visit information includes any interactions with health care professionals other than study staff or designees including hospitalization; in person emergency, urgent, or primary care visits; or any other in person visit attended by the participant and a health care profession. Identify the nature and cause of the visit.
s FLU PRO Plus questionnaire should be completed daily from Day 1 through Day 14 (if available).
t Only AEs, medically attended visit information, and concomitant medications review are needed if the visit is done by phone.

Appendix 3. Pregnancy Precautions, Definition for Female of Childbearing Potential, and Contraceptive Requirements

1) Definitions

a. Definition of Childbearing Potential

For the purposes of this study, a female born participant is considered of childbearing potential following the initiation of puberty (Tanner stage 2) until becoming post-menopausal, unless permanently sterile or with medically documented ovarian failure. No documentation of Tanner stage will be required for people unless deemed prepubescent.

Women are considered to be in a postmenopausal state when they are ≥ 54 years of age with cessation of previously occurring menses for ≥ 12 months without an alternative cause.

Permanent sterilization includes hysterectomy, bilateral oophorectomy, or bilateral salpingectomy in a female participant of any age.

b. Definition of Male Fertility

For the purposes of this study, a male born participant is considered fertile after the initiation of puberty unless the participant is permanently sterile by bilateral orchidectomy or with medical documentation.

2) Contraception Requirements for Female Participants

a. Study Drug Effects on Pregnancy and Hormonal Contraception

Data from nonclinical studies of RDV have demonstrated no adverse effect on fertility or embryo-fetal development. Remdesivir has not yet been studied in pregnant women. Before enrolling into studies with RDV, women of childbearing potential must have pregnancy testing performed at screening.

Available data indicate that RDV potentially causes an interaction with hormonal contraception that is considered of limited significance. Hormonal methods must be used with a barrier method.

b. Contraception Requirements for Female Participants of Childbearing Potential

The inclusion of non-pregnant female participants of childbearing potential requires the use at least an acceptable contraceptive measure. Female participants must agree to 1 of the following from screening until 30 days after the last study drug dose:

- Complete abstinence from intercourse of reproductive potential. Abstinence is an acceptable method of contraception only when it is in line with the participant's preferred and usual lifestyle.

Or

- Consistent and correct use of 1 of the following methods of birth control listed below:

  Non-hormonal intrauterine device (IUD)

  Hormonal IUD (must be used in conjunction with a barrier method)

  Bilateral tubal occlusion (upon medical assessment of surgical success)

  Vasectomy in the male partner (upon medical assessment of surgical success)

Or

- Female participants who wish to use a hormonally-based method must use it in conjunction with a barrier method, preferably a male condom. Hormonal methods are restricted to those associated with the inhibition of ovulation. Hormonally-based contraceptives and barrier methods permitted for use in this protocol are as follows:

  Hormonal methods (each method must be used with a barrier method, preferably male condom)

  - Oral contraceptives (either combined or progesterone only)
  - Injectable progesterone
  - Subdermal contraceptive implant
  - Transdermal contraceptive patch
  - Contraceptive vaginal ring

  Barrier methods

  - Male condom (with or without spermicide)
  - Female condom (with or without spermicide)
  - Diaphragm with spermicide
  - Cervical cap with spermicide
  - Sponge with spermicide

Inclusion of methods of contraception in this list of permitted methods does not imply that the method is approved in any country or region. Methods should only be used if locally approved.

Female participants must also refrain from egg donation and in vitro fertilization during treatment and until the end of contraception requirement.

3) Contraception Requirements for Male Participants

During the study male participants with female partners of childbearing potential should use condoms when engaging in intercourse of reproductive potential.

4) Unacceptable Birth Control Methods

Birth control methods that are unacceptable include periodic abstinence (eg, calendar, ovulation, symptothermal, post-ovulation methods), withdrawal (coitus interruptus), spermicides only, and lactational amenorrhea method. A female condom and a male condom should not be used together.

5) Procedures to be Followed in the Event of Pregnancy

Participants will be instructed to notify the investigator if they become pregnant at any time during the study, or if they become pregnant within 30 days of last study drug dose. Participants who become pregnant or who suspect that they are pregnant during the study must report the information to the investigator. Participants whose partner has become pregnant or suspects she is pregnant during the study must report the information to the investigator. Instructions for reporting pregnancy, partner pregnancy, and pregnancy outcome are outlined in Section 7.7.2.1.

333 Lakeside Drive
Foster City, CA 94404

PROTOCOL AMENDMENT SUMMARY OF CHANGES

PROTOCOL AMENDMENT #1

STUDY GS-US-540-9012

A Phase 3 Randomized, Double-Blind Placebo-Controlled Trial to Evaluate the Efficacy and Safety of Remdesivir (GS-5734™) Treatment of COVID-19 in an Outpatient Setting

**Original Protocol Date:**	21 July 2020
**Amendment 1 Date:**	11 August 2020

**Rationale:**	Herein is a summary of the major changes made to the original protocol dated 21 July 2020 and reflected in Amendment #1 dated 11 August 2020.<br><br>Changes made to the original protocol include modifications for clarification and in response to FDA comments.<br><br>• Added ClinicalTrials.gov identifier<br>• Increased the number of planned study centers to 150<br>• Removed restriction on percentage of participants that may be enrolled from skilled nursing facilities<br>• Decreased minimum age to include adolescent participants ages ≥ 12<br>• Modified inclusion and exclusion criterion<br>• Added sputum samples for SARS-CoV-2 RT-qPCR viral load testing and possible resistance testing<br>• Added study drug administration instructions<br>• Revised Section 7.3.2 Adverse Events<br>• Removed Appendix 2 Pandemic Risk Assessment and Mitigation Plan as it is not applicable for this study<br><br>Specific changes contained in Amendment 1 are presented herein as ***bold and italicized*** or ~~strikethrough~~. These changes were made to enhance the clarity of the protocol and to perform administrative updates.<br>Study synopsis and all applicable sections are updated to align with above mentioned changes in the protocol.<br>In addition, the opportunity is taken to correct typographical or grammatical errors.

**Global Changes:**	• Replaced the word "subject" with "participant" throughout for consistency<br>• Correction of typographical or grammatical errors

**Section:**	Protocol Cover Page, Protocol Synopsis
**Original Text:**	IND Number: 147753<br>EudraCT Number: 2020-003510-12<br>Clinical Trials.gov Identifier: TBD
**Revised Text:**	IND Number: 147753<br>EudraCT Number: 2020-003510-12<br>Clinical Trials.gov Identifier: NCT04501952
**Rationale:**	Addition of clinicaltrials.gov identifier

**Section:**	Protocol Synopsis
**Original Text:**	**Study Centers Planned:** Approximately 100 centers globally
**Revised Text:**	**Study Centers Planned:** Approximately ~~100~~ ***150*** centers globally
**Rationale:**	Increased the number of participating centers in the study to help complete enrollment within planned timelines.

**Section:**	Protocol Synopsis, Section 2. Objectives, Section 8.1.1 Analysis Objectives
**Original Text:**	None
**Revised Text:**	***To assess patient reported outcome using the COVID-19 adapted InFLUenza Patient-Reported Outcome (FLU-PRO©) questionnaire and validate the questionnaire (if available)***
**Rationale:**	The FLU-PRO data may be used to validate the questionnaire when completed by participants.

**Section:**	Protocol Synopsis, Section 3.3 Study Treatments
**Original Text:**	**Target Population:** Non-hospitalized participants with early stage COVID-19 and at least one risk factor for disease progression. Up to 30% of enrolled participants may come from skilled nursing facilities.
**Revised Text:**	**Target Population:** Non-hospitalized participants with early stage COVID-19 and at least one risk factor for disease progression. ~~Up to 30% of enrolled participants may come from skilled nursing facilities.~~
**Rationale:**	Removed the limit in the number of participants that can be enrolled that reside in skilled nursing facilities.

**Section:**	Protocol Synopsis, Section 4.2 Inclusion Criteria
**Original Text:**	• Willing and able to provide written informed consent, or with a legal representative who can provide informed consent
**Revised Text:**	• Willing and able to provide written informed consent ***(age ≥18) or assent (age ≥12 to <18, where locally and nationally approved),*** or with a legal representative who can provide informed consent
**Rationale:**	Allow the enrollment of adolescent participants in the study.

**Section:**	Protocol Synopsis, Section 6 Study Procedures
**Original Text:**	SARS-CoV-2 RT-qPCR testing will be performed on Day 1, 2, 3, 7 and 14.

**Revised Text:**	Nasopharyngeal swabs and sputum samples will be collected on Days 1, 2, 3, 7, and 14 for SARS-CoV-2 RT-qPCR viral load testing and possible resistance testing.
**Rationale:**	Sputum samples will be collected on the same days as nasopharyngeal swabs in participants with a productive cough.

**Section:**	Protocol Synopsis, Section 3.1 Endpoints, Section 8.1.4 Other Endpoints of Interest
**Original Text:**	The secondary efficacy endpoints of this study are:<br>• All-cause mortality at Day 28<br>• Rate of hospitalization by Day 28<br>• Time-weighted average change in SARS-CoV-2 viral load from baseline to Day 7<br>• Time to resolution of COVID-19-related symptoms<br>• The exploratory endpoints of this study are:<br>• Time to symptom resolution of each domain of COVID-19 adapted FLU-PRO<br>• Change from baseline in COVID-19 adapted FLU-PRO total score and score in each domain<br>• Time-weighted average change in SARS-CoV-2 viral load from baseline to Day 14<br>• Time to first negative SARS-CoV-2 PCR<br>• Emergence of viral resistance to RDV<br>• Baseline levels and change from baseline for inflammation/immune-related, acute respiratory distress syndrome-related and coagulation-related biomarkers<br>• Proportion of participants progressing to outpatient oxygen requirement by Day 14<br>• Proportion of participants admitted to the intensive care unit by Day 28<br>• Proportion of participants started on mechanical ventilation by Day 28<br>• The plasma concentrations and PK parameters of RDV and metabolites

<table>
<tr><td>Revised Text:</td><td>The secondary efficacy endpoints of this study are:
• All-cause mortality at Day 28
• Rate of hospitalization by Day 28
• Time-weighted average change in SARS-CoV-2 viral load from baseline to Day 7
• Time to resolution of COVID-19-related symptoms
• Proportion of participants progressing to requiring oxygen supplementation by Day 28
The exploratory endpoints of this study are:
• Time to symptom resolution of each domain of COVID-19 adapted FLU-PRO
• Change from baseline in COVID-19 adapted FLU-PRO total score and score in each domain
• Psychometric validity of COVID-19 FLU-PRO questionnaire
• Time-weighted average change in SARS-CoV-2 viral load from baseline to Day 14
• Time to first negative SARS-CoV-2 PCR
• Emergence of viral resistance to RDV
• Baseline levels and change from baseline for inflammation/immune-related, acute respiratory distress syndrome-related and coagulation-related biomarkers
• <s>Proportion of participants progressing to outpatient oxygen requirement by Day 14</s>
• Proportion of participants admitted to the intensive care unit by Day 28
• Proportion of participants started on mechanical ventilation by Day 28
• The plasma concentrations and PK parameters of RDV and metabolites</td></tr>
<tr><td>Rationale:</td><td>Moved the exploratory endpoint of outpatient oxygen requirement to a secondary endpoint and moved to Day 28 rather than Day 14. Added the validation of the FLU-PRO questionnaire as an exploratory endpoint since validation may be required in this new disease state.</td></tr>
</table>

**Section:**	Section 1.3 Rationale for This Study
**Original Text:**	Based on the European Medicines Agency (EMA) review summarizing the safety of cyclodextrins as excipients {Committee for Medicinal Products for Human Use (CHMP) 2014}, which indicates approximately 250 mg/kg/day of SBECD (12 g/day based on a 48 kg human) is safe, participants weighing more than 48 kg may be enrolled regardless of prior or current renal impairment.

**Revised Text:**	Based on the European Medicines Agency (EMA) review summarizing the safety of cyclodextrins as excipients {Committee for Medicinal Products for Human Use (CHMP) 2014}, which indicates approximately 250 mg/kg/day of SBECD (12 g/day based on a 48 kg human) is safe, participants ~~weighing more than 48 kg may be enrolled regardless of prior or current renal impairment~~ ***will not be required to discontinue RDV if their creatinine clearance is 30 mL/min during the dosing period***.
**Rationale:**	Modified language as the protocol now excludes participants with creatinine clearance < 30 mL/min but does not require discontinuation of eligible participants once study medication is initiated due to the risk-benefit ratio.

**Section:**	Section 1.6 Risk/Benefit Assessment for the Study, Appendix 2: Pandemic Risk Assessment and Mitigation Plan
**Original Text:**	During a pandemic, additional potential risks to subjects may include adequate study drug availability, interruptions to the study visit schedule, and adherence to protocol-specified safety monitoring or laboratory assessments. Refer to Appendix 2 for further details on the risks and risk mitigation strategy.
**Revised Text:**	~~During a pandemic, additional potential risks to subjects may include adequate study drug availability, interruptions to the study visit schedule, and adherence to protocol-specified safety monitoring or laboratory assessments. Refer to Appendix 2 for further details on the risks and risk mitigation strategy.~~
**Rationale:**	Removed text associated with the pandemic as the study is designed to address a current pandemic and the template language is not applicable. In addition, the study allows for the use of home health services and telehealth visits to avoid missed data.

**Section:**	Section 3.5 Discontinuation Criteria, Section 6.7.1 Criteria for Discontinuation of Study Treatment
**Original Text:**	Study drug dosing in an individual participant will be placed on hold and may be discontinued, following a review of all available clinical data by the medical monitor and discussion with the investigator, if any of the following occurs:<br>• Any serious AE (SAE) or ≥ Grade 3 AE suspected to be related to RDV<br>• Any elevations in ALT or AST ≥ 5 × upper limit of normal (ULN); or ALT > 3 × ULN and total bilirubin > 2 × ULN, confirmed by immediate repeat testing
**Revised Text:**	Study drug dosing in an individual participant will be placed on hold and may be discontinued, following a review of all available clinical data by the medical monitor and discussion with the investigator, if any of the following occurs:<br>• Any serious AE (SAE) or ≥ Grade 3 AE suspected to be related to RDV<br>• Any elevations in ALT or AST ≥ 5 × upper limit of normal (ULN); or ALT > 3 × ULN and total bilirubin > 2 × ULN, confirmed by immediate repeat testing<br>• ***Infusion-related systemic reaction ≥ Grade 2 or infusion-related localized reaction ≥ Grade 3***

**Rationale:**	This additional discontinuation criterion was added to ensure participants' safety.

**Section:**	Protocol Synopsis, Section 4.2 Inclusion Criteria
**Original Text:**	Participants must meet all of the following inclusion criteria to be eligible for participation in this study:<br>1) Willing and able to provide written informed consent, or with a legal representative who can provide informed consent<br>2) Either:<br>Age ≥ 18 years with at least 1 of the following pre-existing risk factors for progression to hospitalization:<br>a) Chronic lung disease: chronic obstructive pulmonary disease, moderate-to-severe asthma, cystic fibrosis, pulmonary fibrosis<br>b) Hypertension: systemic or pulmonary<br>c) Cardiovascular or cerebrovascular disease: coronary artery disease, congenital heart disease, heart failure, cardiomyopathy, history of stroke<br>d) Diabetes mellitus: Type 1 or 2<br>e) Obesity (BMI ≥ 30)<br>f) Immunocompromised state<br>g) Chronic kidney disease: any stage<br>h) Chronic liver disease<br>i) Current cancer<br>j) Sickle cell disease<br>OR aged ≥ 60 years, regardless of the presence of other pre-existing risk factors for progression<br>3) SARS-CoV-2 infection confirmed by PCR ≤ 4 days prior to screening

**Revised Text:**	Participants must meet all of the following inclusion criteria to be eligible for participation in this study:<br>1) Willing and able to provide written informed consent, or with a legal representative who can provide informed consent (participants ≥ 18 years of age) or assent (participants ≥ 12 and < 18 years of age) prior to performing study procedures. For participants ≥ 12 and < 18 years of age, a parent or legal guardian must be willing and able to provide written informed consent prior to performing study procedures<br>2) Either:<br>Age ≥ 18 years (at all sites) or aged ≥ 12 and < 18 years of age weighing ≥ 40 kg (where permitted according to local law and approved nationally and by the relevant institutional review board [IRB] or independent ethics committee [IEC]) with at least 1 of the following pre-existing risk factors for progression to hospitalization:<br>a) Chronic lung disease: chronic obstructive pulmonary disease, moderate-to-severe asthma, cystic fibrosis, pulmonary fibrosis<br>b) Hypertension: systemic or pulmonary<br>c) Cardiovascular or cerebrovascular disease: coronary artery disease, congenital heart disease, heart failure, cardiomyopathy, history of stroke<br>d) Diabetes mellitus: Type 1 or 2<br>e) Obesity (BMI ≥ 30)<br>f) Immunocompromised state<br>g) Chronic ***mild or moderate*** kidney disease~~: any stage~~<br>h) Chronic liver disease<br>i) Current cancer<br>j) Sickle cell disease<br>OR aged ≥ 60 years, regardless of the presence of other pre-existing risk factors for progression<br>3) SARS-CoV-2 infection confirmed by PCR ≤ 4 days prior to screening
**Rationale:**	Revised inclusion criteria to allow the enrollment of adolescents where permitted by local law and revised chronic kidney disease inclusion criterion to be consistent with renal exclusion criterion.

**Section:**	Protocol Synopsis, Section 4.3 Exclusion Criteria
**Original Text:**	Participants who meet *any* of the following exclusion criteria are not eligible to be enrolled in this study:<br>1) Participation in any other clinical trial of an experimental treatment and prevention for COVID-19<br>2) Prior hospitalization for COVID-19<br>3) Treatment with other agents with actual or possible direct antiviral activity against SARS-CoV-2<br>4) Use of hydroxychloroquine or chloroquine ≤ 7 days prior to screening<br>5) Requiring oxygen supplementation<br>6) ALT or AST ≥ 5 × ULN at screening or within 90 days of screening<br>7) Creatinine clearance < 30 mL/min at screening or < 90 days before screening ONLY if the participant's weight is < 48 kg<br>8) Breastfeeding female
**Revised Text:**	Participants who meet *any* of the following exclusion criteria are not eligible to be enrolled in this study:<br>1) Participation in any other clinical trial of an experimental treatment and prevention for COVID-19<br>2) Prior hospitalization for COVID-19<br>3) Treatment with other agents with actual or possible direct antiviral activity against SARS-CoV-2<br>4) Use of hydroxychloroquine or chloroquine ≤ 7 days prior to screening<br>5) Requiring oxygen supplementation<br>6) ALT or AST ≥ 5 × ULN at screening or within 90 days of screening ***Note: if per local practice only ALT is routinely measured, exclusion criteria will be evaluated on ALT alone***<br>7) Creatinine clearance < 30 mL/min at screening or within 90 days of screening using the Cockcroft-Gault formula in participants ≥ 18 years of age or the Schwartz formula in participants < 18 years of age (see Section 6.6.1) ~~< 90 days before screening ONLY if the participant's weight is < 48 kg~~<br>8) Currently breastfeeding (nursing) ~~female~~
**Rationale:**	Revised exclusion criterion for clarity and alignment with clinical practice.

**Section:**	Section 5.1.1 Randomization
**Original Text:**	Participants who meet all randomization eligibility criteria will be randomized in a 1:1 ratio to Treatment Group A or Treatment Group B starting on Day 1 and assigned a participant number. Randomization will be stratified by residence in a skilled nursing facility, age (<60 vs ≥ 60), and region (US vs ex-US).

**Revised Text:**	Participants who meet all randomization eligibility criteria will be randomized in a 1:1 ratio to Treatment Group A or Treatment Group B ~~starting on Day 1~~ and assigned a participant number. Randomization will be stratified by residence in a skilled nursing facility, age (<60 vs ≥ 60), and region (US vs ex-US).<br>***Randomization may occur approximately one day prior to the Day 1 visit.***
**Rationale:**	To allow for the participant to be randomized prior to Day 1 for the dispensation of study drug.

**Section:**	Section 5.1.2 Blinding
**Original Text:**	During the randomized phase participants and all personnel directly involved in the conduct of the study will be blinded to treatment assignment. Specified personnel may be unblinded based on their study role. Study drug will be dispensed by the study pharmacist, or designee, in a blinded fashion to the participants. The Pharmacokinetics File Administrator, or designee, in Bioanalytical Operations and/or Clinical Data Management, who facilitates the data transfer of PK files between Gilead and vendors, will remain unblinded. Individuals in Clinical Packaging and Labeling or Clinical Supply Management who have an Unblinded Inventory Manager role in the IXRS system for purposes of study drug inventory management will remain unblinded. Individuals in Pharmacovigilance and Epidemiology (PVE) responsible for safety signal detection, investigational new drug (IND) safety reporting, and/or expedited reporting of suspected unexpected serious adverse reactions (SUSARs) may be unblinded to individual case data and/or group level summaries. External (ie, contract research organizations) biostatisticians and programmers will be unblinded for the IDMC, IND safety reporting. Regulatory Quality and Compliance personnel in Research and Development may also be unblinded for purposes of supporting Quality Assurance activities and/or Regulatory Agency inspections.

**Revised Text:**	During the randomized phase participants and all personnel directly involved in the conduct of the study will be blinded to treatment assignment. Specified personnel may be unblinded based on their study role. Study drug will be dispensed by the study pharmacist, or designee, in a blinded fashion to the participants. The Pharmacokinetics File Administrator, or designee, in Bioanalytical Operations and/or Clinical Data Management, who facilitates the data transfer of PK files between Gilead and vendors, will remain unblinded. ***Individuals in clinical virology performing sample selection for resistance analysis may be unblinded.*** Individuals in Clinical Packaging and Labeling or Clinical Supply Management who have an Unblinded Inventory Manager role in the IXRS system for purposes of study drug inventory management will remain unblinded. Individuals in Pharmacovigilance and Epidemiology (PVE) responsible for safety signal detection, investigational new drug (IND) safety reporting, and/or expedited reporting of suspected unexpected serious adverse reactions (SUSARs) may be unblinded to individual case data and/or group level summaries. External (ie, contract research organizations) biostatisticians and programmers will be unblinded for the IDMC, IND safety reporting. Regulatory Quality and Compliance personnel in Research and Development may also be unblinded for purposes of supporting Quality Assurance activities and/or Regulatory Agency inspections.
**Rationale:**	Added virology as a functional group that may be unblinded when resistance analysis is performed.

**Section:**	Section 5.3.1 Administration Instructions
**Original Text:**	N/A
**Revised Text:**	(see below)

5.3.1 Administration Instructions

The prepared diluted solution should not be administered simultaneously with any other medication. The compatibility of RDV injection with IV solutions and medications other than 0.9% sodium chloride is not known. Administer the diluted solution with the infusion rate described in Table 2.

Table 2: Recommended Rate of Infusion—Diluted RDV for Injection Lyophilized Powder in Adults and Pediatric Patients 12 Years of Age and Older and Weighing at Least 40 kg

Infusion bag volume	Infusion time	Rate of infusion
250 mL	30 min	8.33 mL/min
	60 min	4.17 mL/min
	120 min	2.08 mL/min
100 mL	30 min	3.33 mL/min
	60 min	1.67 mL/min
	120 min	0.83 mL/min

**Rationale:**	Added the recommended infusion duration.

**Section:**	Section 6.2.1 Screening Visit, Study Procedures Table
**Original Text:**	Participant*s* will be screened within 2 days before randomization to determine eligibility for participation in the study. The following will be performed and documented at screening:<br>• Obtain written informed consent<br>• Obtain medical history including the following information: date of first symptoms, overall symptoms, exposure source, demographics, baseline characteristics, allergies and all other medical history<br>• Review and document prior and concomitant medications<br>• Complete physical examination (urogenital/anorectal exam not required) including, vital signs (heart rate, temperature, blood pressure), body weight, and height.<br>• Respiratory status:<br>Respiratory rate<br>Oxygenation: SpO_2 on room air<br>• Obtain blood samples for ALT, AST, serum creatinine, and creatinine clearance if prior history of renal or liver disease (if no history of severe renal or severe renal disease but with weight ≥ 48 kg or liver disease or if results available within the prior 3 months, can defer until Day 1)
**Revised Text:**	Participant*s* will be screened within 2 days before randomization to determine eligibility for participation in the study. The following will be performed and documented at screening:<br>• Obtain written informed consent<br>• Obtain medical history including the following information: date of first symptoms, overall symptoms, exposure source, demographics, baseline characteristics, allergies and all other medical history<br>• Review and document prior and concomitant medications<br>• Complete physical examination (urogenital/anorectal exam not required) including, vital signs (heart rate, temperature, blood pressure), body weight, and height<br>• Respiratory status:<br>Respiratory rate<br>Oxygenation: SpO_2 on room air<br>• Obtain ALT (and AST where available), creatinine and creatinine clearance (calculated using Cockcroft-Gault or Schwartz formula; (see Section 6.6.1) if not available within 90 days of screening ~~Obtain blood samples for ALT, AST, serum creatinine, and creatinine clearance if prior history of renal or liver disease (if no history of severe renal or severe renal disease but with weight ≥ 48 kg or liver disease or if results available within the prior 3 months, can defer until Day 1)~~
**Rationale:**	Revised procedure for clarity and to align with revised eligibility criteria.

**Section:**	Section 6.3 Treatment Assessments (Baseline/Day 1, Day 2, and Day 3), Study Procedures Table
**Original Text:**	The date of randomization will be considered Day 1. The following evaluations are to be completed at the Day 1 visit. The investigator must have confirmed eligibility before proceeding with randomization on the Day 1 visit. If the screening and Day 1 visits occur on the same day, no procedures need to be repeated. Participants must complete the following assessments before being administered study drug:<br>• Vital signs (heart rate, temperature, blood pressure, body weight) pre-infusion, post-infusion, and when post infusion observation is completed<br>• Respiratory status:<br>Respiratory rate<br>Oxygenation: SpO_2 on room air<br>• Complete physical examination (Day 1)<br>• Symptom-directed physical examination (Day 2, Day 3)<br>• Review of AEs and concomitant medications<br>• Obtain blood samples as described in Section 6.6 (Day 1, Day 3)<br>• Nasopharyngeal swab samples for SARS-CoV-2 RT-qPCR testing and possible viral resistance testing<br>• Completion of the COVID-adapted-FLU-PRO questionnaire (if available)<br>• Administration of study drug. Instructions on study drug administration are available in a separate manual.

**Revised Text:**	~~The date of randomization will be considered Day 1.~~ The following evaluations are to be completed at the Day 1 visit. The investigator must have confirmed eligibility before proceeding with randomization ~~at~~ ***and*** the Day 1 visit. If the screening and Day 1 visits occur on the same day, no procedures need to be repeated. Participants must complete the following assessments before being administered study drug:<br>• Vital signs (heart rate, temperature, blood pressure~~, body weight~~) pre-infusion, post-infusion, and when post infusion observation is completed<br>• ***Body weight***<br>• Respiratory status:<br>Respiratory rate<br>Oxygenation: SpO_2 on room air<br>• Complete physical examination (Day 1)<br>• Symptom-directed physical examination (Day 2, Day 3)<br>• Review of AEs and concomitant medications<br>• Obtain blood samples as described in Section 6.6 (Day 1, Day 3)<br>• Nasopharyngeal swab samples for SARS-CoV-2 RT-qPCR testing and possible viral resistance testing<br>• ***Sputum sample collection from participants with productive cough for SARS-CoV-2 quantitative reverse transcriptase polymerase chain reaction (RT-qPCR) testing and possible viral resistance testing***<br>• Completion of the COVID-adapted FLU-PRO questionnaire (if available)<br>• Administration of study drug. Instructions on study drug administration are available in a separate manual.
**Rationale:**	Edited for clarification that randomization may occur prior to Day 1 and body weight should only be performed once prior to the study drug being administered. Added the sputum sample collection for testing.

**Section:**	Section 6.4 Post-treatment Assessments (Day 4 Through Day 14), Study Procedures Table
**Original Text:**	N/A
**Revised Text:**	• ***Sputum sample collection from participants with productive cough for SARS-CoV-2 RT-qPCR testing and possible viral resistance testing (Day 7, Day 14)***
**Rationale:**	Addition of sputum collection in participants with a productive cough for testing.

**Section:**	Section 6.6.1 Blood Samples
**Original Text:**	• Estimated glomerular filtration rate (screening [if required] Day 1, Day 3, Day 7, Day 14) according to:<br>Cockcroft-Gault formula for creatinine clearance for participants ≥ 18 years of age<br>Men: $\frac{(140 \quad \text{age in years}) \times (\text{wt in kg})}{72 \times (\text{serum creatinine in mg/dL})}$ CLcr (mL/min)<br>Women: $\frac{(140 \quad \text{age in years}) \times (\text{wt in kg}) \times 0.85}{72 \times (\text{serum creatinine in mg/dL})}$ CLcr (mL/min)
**Revised Text:**	• Estimated glomerular filtration rate (screening [if required] Day 1, Day 3, Day 7, Day 14) according to:<br>Schwartz Formula for participants < 18 years of age, where S_{Cr} is serum creatinine (mg/dL)<br>***Adolescent boys ≥ 12 years of age: 0.70 × L/ S_{Cr} (L is height in cm)***<br>***Adolescent girls ≥ 12 years of age: 0.55 × L/ S_{Cr} (L is height in cm)***<br>Cockcroft-Gault formula for creatinine clearance (CL_{cr}) for participants ≥ 18 years of age<br>Men: $\frac{(140 \quad \text{age in years}) \times (\text{wt in kg})}{72 \times (\text{serum creatinine in mg/dL})}$ CLcr (mL/min)<br>Women: $\frac{(140 \quad \text{age in years}) \times (\text{wt in kg}) \times 0.85}{72 \times (\text{serum creatinine in mg/dL})}$ CLcr (mL/min)
**Rationale:**	Revised to include formula to be used for participants < 18 years of age due to updated eligibility criteria.

**Section:**	Section 6.6.4 Virology Assessments, Section 6.10 Sample Storage
**Original Text:**	Nasopharyngeal swab samples will be used to assess SARS-CoV-2 viral load by RT-qPCR. Once viral load testing is complete, the remnant samples may be used to evaluate the emergence of viral resistance by SARS-CoV-2 sequencing and/or phenotypic testing.
**Revised Text:**	Nasopharyngeal swab ***and sputum*** samples will be used to assess SARS-CoV-2 viral load by RT-qPCR. Once viral load testing is complete, the remnant samples may be used to evaluate the emergence of viral resistance by SARS-CoV-2 sequencing and/or phenotypic testing ***according to the Virology Analysis Plan***.
**Rationale:**	Revised for clarity and update with the sputum sample being collected.

**Section:**	Section 7.3.2 Adverse Events
**Original Text:**	All AEs should be followed up until resolution or until the AE is stable, if possible. Gilead may request that certain AEs be followed beyond the protocol-defined follow-up period.
**Revised Text:**	All AEs ***and clinically significant laboratory abnormalities will be*** ~~should be~~ followed ~~up~~ until resolution or ***stability of the abnormality has been demonstrated*** ~~until the AE is stable~~, if possible. Gilead may request that certain AEs be followed beyond the protocol-defined follow-up period.
**Rationale:**	Revised so that all adverse events, SAEs, and clinically significant laboratory abnormalities are followed until resolution or until stability of the abnormality has been demonstrated.

**Section:**	Section 8.5.2 Secondary Analyses
**Original Text:**	The secondary endpoint of hospitalization by Day 28 will be analyzed similarly as in the primary analysis. The all-cause mortality at Day 28 will be compared between the 2 treatment groups using a Fisher exact test. other endpoints of interest related to proportion of participants will be compared between treatment groups using a chi-square test or Fisher exact test. Endpoints that are measured as time to first event will be compared between treatment groups using the log-rank test and continuous endpoints will be compared between treatment groups using a Wilcoxon rank sum test or analysis of variance model.
**Revised Text:**	The secondary endpoint of hospitalization by Day 28 will be analyzed similarly as in the primary analysis. The all-cause mortality at Day 28 will be compared between the 2 treatment groups using a Fisher exact test. ***The proportion of participants progressing to requiring oxygen supplementation by Day 28 will be estimated using the Kaplan-Meier method and compared between the 2 treatment groups using a log-rank test.*** Other endpoints of interest related to proportion of participants will be compared between treatment groups using a chi-square test or Fisher exact test. Endpoints that are measured as time to first event will be compared between treatment groups using the log-rank test and continuous endpoints will be compared between treatment groups using a Wilcoxon rank sum test or analysis of variance model.
**Rationale:**	Addition of the oxygen supplementation secondary endpoint required updating the analysis methods.

**Section:**	Pregnancy Precautions, Definition for Female of Childbearing Potential, and Contraceptive Requirements
**Original Text:**	For the purposes of this study, a female born participant is considered of childbearing potential until becoming post-menopausal, unless permanently sterile or with medically documented ovarian failure.

**Revised Text:**	For the purposes of this study, a female born participant is considered of childbearing potential ***following the initiation of puberty (Tanner stage 2) until becoming post-menopausal, unless permanently sterile or with medically documented ovarian failure. No documentation of Tanner stage will be required for people unless deemed prepubescent*** ~~until becoming post-menopausal, unless permanently sterile or with medically documented ovarian failure~~.
**Rationale:**	Modified definition of childbearing potential as adolescents are now allowed to enroll in the study.

"I have read and understand the above and agree to this protocol amendment as written."	
**Principal Investigator**	**Date**

333 Lakeside Drive
Foster City, CA 94404

PROTOCOL AMENDMENT SUMMARY OF CHANGES

PROTOCOL AMENDMENT #2

STUDY GS-US-540-9012

A Phase 3 Randomized, Double-Blind Placebo-Controlled Trial to Evaluate the Efficacy and Safety of Remdesivir (GS-5734™) Treatment of COVID-19 in an Outpatient Setting

**Original Protocol Date:**	21 July 2020
**Amendment 1 Date:**	11 August 2020
**Amendment 1.1 (United Kingdom):**	07 October 2020
**Amendment 1.1 (Germany):**	14 October 2020
**Amendment 2 Date:**	06 November 2020

**Rationale:**	Herein is a summary of the major changes made to the protocol amendment #1 dated 11 August 2020 and reflected in Amendment #2 dated 06 November 2020.<br><br>The majority of changes reflect updates to the endpoints of the study made in response to evolving treatment paradigms and understanding of COVID-19. Compared to the available data at the time the study was designed, diagnosed patients with risk factors for developing severe COVID-19 are less likely to be immediately hospitalized and are more likely to receive care in an outpatient setting. In addition, mortality for symptomatically treated COVID-19 appears to have been reduced both in hospitalized patients and those who have not been hospitalized. The revised endpoints allow the overall potential for treatment with remdesivir (RDV) to reduce the burden throughout the health care system to be assessed by including all medically attended visits, those visits where the participant and a health care professional are physically present, to be included in the composite endpoint.<br><br>In addition, endpoints have been aligned with recently reported studies of potential COVID-19 treatment, eg, {Chen 2020} and {Regeneron 2020}, to better allow potential inter-study comparisons to be made in the absence of direct comparative trials.<br><br>Additional changes made to Amendment #2 include modifications for clarification and in response to FDA and other regulatory agencies' comments.

	• Updated General Information section to refer to the latest IB<br>• Addition, update, and clarification of study endpoints<br>• Addition of coagulation panel<br>• Clarification and/or update of inclusion and exclusion criteria<br>• Addition of complete physical examination requirements section<br><br>Specific changes contained in Amendment #2 are presented herein as ***bold and italicized*** or ~~strikethrough~~.<br><br>The protocol synopsis, study procedures table, and all applicable sections were updated to align with above-mentioned changes in the protocol.<br><br>In addition, the opportunity was taken to correct typographical or grammatical errors.

**Global Changes:**	• Replaced *Pharmacovigilance and Epidemiology* (*PVE)* with ***Global Patient Safety (GLPS)*** throughout the protocol to capture the change in name of the department<br>• Updated *FLU-PRO* questionnaire references throughout the protocol with ***FLU-PRO Plus*** to note appropriate terminology<br>• Expansion of primary objective and associated endpoints to evaluate the efficacy of a 3-day course of RDV in reducing the rate of ***all-cause medically attended visits (medical visits attended in person by the participant and a health care professional)*** rather than hospitalization only.

**Section:**	Section 1 Introduction
**Original Text:**	**1.1. Background**<br>Severe acute respiratory syndrome (SARS)-coronavirus (CoV)-2, a single-stranded RNA virus, is identified as the cause of an outbreak of respiratory illness that was first detected in Wuhan, China in December 2019. The virus has now spread globally, resulting in a global pandemic and causing severe respiratory illness throughout the world. Severe cases progress to pneumonia and multi-organ failure, which can lead to death. Currently, there are no drugs approved for the treatment of coronavirus disease 2019 (COVID-19) in the United States (US). Gilead Sciences, Inc. (Gilead) has been working with global health authorities to respond to the ongoing pandemic and to evaluate the utility of intravenous (IV) remdesivir (RDV; GS-5734™) as a treatment option for COVID-19 through clinical trials. On 01 May 2020, the US Food and Drug Administration (FDA) issued an Emergency Use Authorization (EUA) {U. S. Food and Drug Administration (FDA) 2020} for IV RDV for the treatment of COVID-19 based on data from Adaptive COVID-19 Treatment Trial (ACTT-1), an

ongoing, adaptive, randomized, double-blind, placebo-controlled, multicenter study evaluating IV RDV versus placebo in hospitalized patients with COVID-19 (sponsored by National Institute of Allergy and Infectious Diseases [NIAID]; Gilead study number CO-US-540-5776) {Beigel 2020} and the randomized, open-label phase (Part A) of an ongoing Gilead-sponsored Phase 3, multicenter study evaluating 2 RDV regimens (5 days versus 10 days) in participants with severe COVID-19 (GS-US-540-5773). Intravenous RDV was subsequently approved for the treatment of COVID-19 in Japan and the European Union (EU).

1.2. Remdesivir (RDV, GS-5734)

Remdesivir is being developed by Gilead Sciences, Inc. (Gilead) and is formulated for IV administration.

1.2.1. General Information

Remdesivir is a nucleotide prodrug that is intracellularly metabolized into an analog of adenosine triphosphate that inhibits viral RNA polymerases and has broad spectrum activity against members of the filoviruses (eg, Ebola virus, Marburg virus, CoVs [eg, SARS-CoV-2, SARS-CoV, Middle East respiratory syndrome-CoV]), and paramyxoviruses (eg, respiratory syncytial virus , Nipah virus, and Hendra virus). For further information on RDV, refer to the current investigator's brochure (IB) for IV RDV. Information in the IB includes:

- Nonclinical pharmacokinetic(s) (PK) and in vitro metabolism
- Nonclinical pharmacology and toxicology
- Clinical experience

Additional relevant information regarding RDV are described below.

1.2.2. Additional Clinical Experience with RDV

1.2.2.1. NIAID ACTT-1 Study in Participants with Mild/Moderate and Severe COVID-19

A randomized, double-blind, placebo-controlled multicenter clinical trial evaluated RDV 200 mg once daily for 1 day followed by RDV 100 mg once daily for 9 days (for a total of up to 10 days of intravenously administered therapy) in hospitalized adult participants with COVID-19 with evidence of lower respiratory tract involvement. The trial enrolled 1062 hospitalized participants: 112 [10.5%] participants with mild/moderate disease and 950 [89.5%] participants with severe disease. A total of 282 participants (26.6%) (n = 129 received RDV) were on mechanical ventilation/ECMO. Participants were randomized in a 1:1 manner, stratified by disease severity at enrollment, to receive RDV (n = 541) or placebo (n = 521), plus standard of care (SOC).

	At baseline, mean age was 59 years (with 36% of participants aged 65 or older); 64% of participants were male, 53% were White, 21% were Black, and 13% were Asian; 11% of participants had mild/moderate disease (12% in the RDV group vs 11% in placebo group) and 89% had severe disease (88% in the RDV group vs 89% in placebo group). The most common comorbidities were hypertension (51.0%), obesity (45.0%), and type 2 diabetes mellitus (31.0%)<br>The primary clinical endpoint was time to recovery within 28 days after randomization, defined as either discharged from the hospital or hospitalized but not requiring supplemental oxygen and no longer requiring ongoing medical care. The median time to recovery was 10 days in the RDV group compared to 14 days in the placebo group (recovery rate ratio 1.31; 95% CI 1.12 to 1.53, $p < 0.001$). Among participants with mild/moderate disease at enrollment (n = 112), the median time to recovery was 5 days in both the RDV and placebo groups (recovery rate ratio 1.16; [95% CI 0.77 to 1.72]). Among participants with severe disease at enrollment (n = 950), the median time to recovery was 11 days in the RDV group compared to 17 days in the placebo group (recovery rate ratio, 1.34; [95% CI, 1.13 to 1.58]; $p < 0.001$).<br>Overall, the odds of improvement in the ordinal scale were higher in the RDV group at Day 15 when compared to the placebo group (odds ratio, 1.46; [95% CI, 1.15 to 1.86], p = 0.002)).<br>1.2.2.2. Study GS-US-540-5773 (Part A) in Participants with Severe COVID-19<br>A randomized, open-label, multicenter clinical trial (Study GS-US-540-5773) of participants at least 12 years of age with confirmed SARS-CoV-2 infection, oxygen saturation of ≤ 94% on room air, and radiological evidence of pneumonia compared 200 participants who received RDV for 5 days with 197 participants who received RDV for 10 days. Participants on mechanical ventilation at screening were excluded. All participants received 200 mg of RDV on Day 1 and 100 mg once daily on subsequent days, plus SOC. The primary endpoint was clinical status on Day 14 assessed by a 7-point ordinal scale ranging from hospital discharge to increasing levels of oxygen and ventilatory support to death.<br>At baseline, the median age of participants was 61 years (range, 20 to 98 years); 64% were male, 75% were White, 12% were Black, and 12% were Asian. More participants in the 10-day group than the 5-day group required invasive mechanical ventilation or ECMO (5% vs 2%), or high-flow oxygen support (30% vs 25%), at baseline. Median duration of symptoms and hospitalization prior to first dose of RDV were similar across treatment groups.

Overall, after adjusting for between-group differences at baseline, participants receiving a 5-day course of RDV had similar clinical status at Day 14 as those receiving a 10-day course (odds ratio for improvement: 0.75; [95% CI 0.51 to 1.12]). In addition, recovery rates were 70% and 58%, and mortality rates were 8% and 11%, in the 5-day and 10-day groups, respectively. There were no significant differences once adjusted for between-group differences at baseline. All-cause mortality at Day 28 was 12% vs 14% in the 5- and 10-day treatment groups, respectively. The most common adverse events (AEs) are recorded in Table 1.

Table 1. Most Common Adverse Events Reported for Participants Overall in Study 5773

n (%)	5 Days N=200	10-Days N=197
Nausea	20 (10)	17 (9)
Acute respiratory failure	12 (6)	21 (11)
Acute kidney injury	4 (2)	16 (8)

1.2.2.3. Study GS-US-540-5774 (Part A) in Participants with Moderate COVID-19

A randomized, open-label multicenter clinical trial (Study GS-US-540-5774) of hospitalized participants at least 12 years of age with confirmed SARS-CoV-2 infection and radiological evidence of pneumonia without reduced oxygen levels compared treatment with RDV for 5 days (n = 191) and treatment with RDV for 10 days (n = 193) with SOC (n = 200). Participants treated with RDV received 200 mg on Day 1 and 100 mg once daily on subsequent days. The primary endpoint was clinical status on Day 11 assessed by a 7-point ordinal scale ranging from hospital discharge to increasing levels of oxygen and ventilatory support to death.

At baseline, the median age of participants was 57 years (range, 12 to 95 years); 61% were male, 61% were white, 19% were black, and 19% were Asian. Baseline clinical status, oxygen support status, and median duration of symptoms and hospitalization prior to first dose of RDV were similar across treatment groups.

Overall, the odds of improvement in the ordinal scale were higher in the 5-day RDV group at Day 11 when compared to those receiving only SOC (odds ratio, 1.65; [95% CI, 1.09 to 2.48], p = 0.017). The odds of improvement in clinical status with the 10-day treatment group when compared to those receiving only SOC were not statistically significant (odds ratio 1.31; [95% CI 0.88 to 1.95]; p = 0.18). At Day 11, observed mortality rates for the 5-day, 10-day, and SOC groups were 0, 1%, and 2%, respectively. All-cause mortality at Day 28 was 2%, 1%, and 3% in the 5-day, 10-day, and SOC groups, respectively. The most common AEs are recorded in Table 2.

Table 2. Most Common Adverse Events Reported for Participants Overall in Study 5774

n (%)	5 Days N=200	10 Days N=193	SOC N=200
Nausea	19 (10)	18 (9)	6 (3)

1.2.2.4. Integrated Safety Findings from Clinical Studies

Assessment of adverse reactions is based on data from four Phase 1 studies in 138 healthy adult participants, 4 Phase 3 studies in 1467 hospitalized participants with COVID-19, and from 163 hospitalized participants with COVID-19 who received RDV in a compassionate use program.

The adverse reactions are listed below by system organ class and frequency. Frequencies are defined as follows: very common (≥ 10%), common (≥ 1% and <10%), uncommon (≥ 0.1% and < 1%), or rare (≥ 0.01% and < 0.1%).

INJURY, POISONING AND PROCEDURAL COMPLICATIONS

Rare: infusion-related reaction

HEPATOBILIARY DISORDERS

Very common: transaminases increased

<u>Description of selected adverse reactions</u>

Transaminases Increased

In healthy volunteer studies, the incidence of increased transaminases was higher in participants who received RDV compared to placebo. In clinical studies of participants with COVID-19, the incidence of increased transaminases was similar in participants treated with RDV compared to placebo or SOC.

Infusion-related Reactions

Infusion-related reactions have been observed following administration of RDV. Signs and symptoms may include hypotension, hypertension, tachycardia, bradycardia, hypoxia, fever, dyspnea, wheezing, angioedema, rash, nausea, diaphoresis, and shivering.

Revised Text:

1.1. Background

Severe acute respiratory syndrome (SARS)-coronavirus (CoV)-2, a single-stranded RNA virus, is identified as the cause of an outbreak of respiratory illness that was first detected in Wuhan, China in December 2019. The virus has now spread globally, resulting in a global pandemic and causing severe respiratory illness throughout the world. Severe cases progress to pneumonia and multi-organ failure, which can lead to death. ~~Currently, there are no drugs approved for the treatment of coronavirus disease 2019 (COVID-19) in the United States (US).~~ Gilead Sciences, Inc. (Gilead) has been working with global health authorities to respond to the ongoing pandemic and to evaluate the utility of intravenous (IV) remdesivir (RDV; GS-5734™) as a treatment option for COVID-19 through clinical

trials. ~~On 01 May 2020, the US Food and Drug Administration (FDA) issued an Emergency Use Authorization (EUA) {U. S. Food and Drug Administration (FDA) 2020} for IV RDV for the treatment of COVID-19 based on data from Adaptive COVID-19 Treatment Trial (ACTT-1), an ongoing, adaptive, randomized, double-blind, placebo-controlled, multicenter study evaluating IV RDV versus placebo in hospitalized patients with COVID-19 (sponsored by National Institute of Allergy and Infectious Diseases [NIAID]; Gilead study number CO-US-540-5776) {Beigel 2020} and the randomized, open-label phase (Part A) of an ongoing Gilead-sponsored Phase 3, multicenter study evaluating 2 RDV regimens (5 days versus 10 days) in participants with severe COVID-19 (GS-US-540-5773). Intravenous RDV was subsequently approved for the treatment of COVID-19 in Japan and the European Union (EU).~~ ***Remdesivir is approved for the treatment of coronavirus disease 2019 (COVID-19) in the United States (US), European Union (EU), Japan, and other countries for populations including adults and pediatric patients (12 years and older and weighing at least 40 kg).***

1.2. Remdesivir (RDV, GS-5734)

Remdesivir is being developed by ~~Gilead Sciences, Inc. (~~Gilead~~)~~ and is formulated for IV administration.

1.2.1. General Information

Remdesivir is an adenosine nucleotide prodrug that distributes into cells where it is metabolized to form the pharmacologically active nucleoside triphosphate metabolite. Metabolism of remdesivir to remdesivir triphosphate has been demonstrated in multiple cell types. Remdesivir triphosphate acts as an analog of adenosine triphosphate (ATP) and competes with the natural ATP substrate for incorporation into nascent RNA chains by the SARS-CoV-2 RNA-dependent RNA polymerase, which results in delayed chain termination during replication of the viral RNA.

For further information on RDV, refer to the investigator's brochure (IB) for RDV.

~~Remdesivir is a nucleotide prodrug that is intracellularly metabolized into an analog of adenosine triphosphate that inhibits viral RNA polymerases and has broad spectrum activity against members of the filoviruses (eg, Ebola virus, Marburg virus, CoVs [eg, SARS-CoV-2, SARS-CoV, Middle East respiratory syndrome CoV]), and paramyxoviruses (eg, respiratory syncytial virus , Nipah virus, and Hendra virus). For further information on RDV, refer to the current investigator's brochure (IB) for IV RDV. Information in the IB includes:~~

- ~~Nonclinical pharmacokinetic(s) (PK) and in vitro metabolism~~
- ~~Nonclinical pharmacology and toxicology~~
- ~~Clinical experience~~

~~Additional relevant information regarding RDV are described below.~~

	**1.2.2. Additional Clinical Experience with RDV**<br>1.2.2.1. NIAID ACTT-1 Study in Participants with Mild/Moderate and Severe COVID-19<br>A randomized, double-blind, placebo-controlled multicenter clinical trial evaluated RDV 200 mg once daily for 1 day followed by RDV 100 mg once daily for 9 days (for a total of up to 10 days of intravenously administered therapy) in hospitalized adult participants with COVID-19 with evidence of lower respiratory tract involvement. The trial enrolled 1062 hospitalized participants: 112 [10.5%] participants with mild/moderate disease and 950 [89.5%] participants with severe disease. A total of 282 participants (26.6%) (n = 129 received RDV) were on mechanical ventilation/ECMO. Participants were randomized in a 1:1 manner, stratified by disease severity at enrollment, to receive RDV (n = 541) or placebo (n = 521), plus standard of care (SOC).<br>At baseline, mean age was 59 years (with 36% of participants aged 65 or older); 64% of participants were male, 53% were White, 21% were Black, and 13% were Asian; 11% of participants had mild/moderate disease (12% in the RDV group vs 11% in placebo group) and 89% had severe disease (88% in the RDV group vs 89% in placebo group). The most common comorbidities were hypertension (51.0%), obesity (45.0%), and type 2 diabetes mellitus (31.0%)<br>The primary clinical endpoint was time to recovery within 28 days after randomization, defined as either discharged from the hospital or hospitalized but not requiring supplemental oxygen and no longer requiring ongoing medical care. The median time to recovery was 10 days in the RDV group compared to 14 days in the placebo group (recovery rate ratio 1.31; 95% CI 1.12 to 1.53, p < 0.001). Among participants with mild/moderate disease at enrollment (n = 112), the median time to recovery was 5 days in both the RDV and placebo groups (recovery rate ratio 1.16; [95% CI 0.77 to 1.72]). Among participants with severe disease at enrollment (n = 950), the median time to recovery was 11 days in the RDV group compared to 17 days in the placebo group (recovery rate ratio, 1.34; [95% CI, 1.13 to 1.58]; p < 0.001).<br>Overall, the odds of improvement in the ordinal scale were higher in the RDV group at Day 15 when compared to the placebo group (odds ratio, 1.46; [95% CI, 1.15 to 1.86], p = 0.002)).<br>1.2.2.2. Study GS-US-540-5773 (Part A) in Participants with Severe COVID-19<br>A randomized, open-label, multicenter clinical trial (Study GS-US-540-5773) of participants at least 12 years of age with confirmed SARS-CoV-2 infection, oxygen saturation of ≤ 94% on room air, and radiological evidence of pneumonia compared 200 participants who received RDV for 5 days with 197 participants who received RDV for 10 days. Participants on mechanical ventilation at screening were excluded. All participants received 200 mg of RDV on Day 1 and 100 mg once daily on subsequent days, plus SOC. The primary endpoint was clinical status on

Day 14 assessed by a 7-point ordinal scale ranging from hospital discharge to increasing levels of oxygen and ventilatory support to death.

At baseline, the median age of participants was 61 years (range, 20 to 98 years); 64% were male, 75% were White, 12% were Black, and 12% were Asian. More participants in the 10-day group than the 5-day group required invasive mechanical ventilation or ECMO (5% vs 2%), or high-flow oxygen support (30% vs 25%), at baseline. Median duration of symptoms and hospitalization prior to first dose of RDV were similar across treatment groups.

Overall, after adjusting for between-group differences at baseline, participants receiving a 5-day course of RDV had similar clinical status at Day 14 as those receiving a 10-day course (odds ratio for improvement: 0.75; [95% CI 0.51 to 1.12]). In addition, recovery rates were 70% and 58%, and mortality rates were 8% and 11%, in the 5-day and 10-day groups, respectively. There were no significant differences once adjusted for between-group differences at baseline. All-cause mortality at Day 28 was 12% vs 14% in the 5- and 10-day treatment groups, respectively. The most common adverse events (AEs) are recorded in Table 1.

Table 1. Most Common Adverse Events Reported for Participants Overall in Study 5773

n (%)	5 Days N=200	10 Days N=197
Nausea	20 (10)	17 (9)
Acute respiratory failure	12 (6)	21 (11)
Acute kidney injury	4 (2)	16 (8)

1.2.2.3. Study GS-US-540-5774 (Part A) in Participants with Moderate COVID-19

A randomized, open-label multicenter clinical trial (Study GS-US-540-5774) of hospitalized participants at least 12 years of age with confirmed SARS-CoV-2 infection and radiological evidence of pneumonia without reduced oxygen levels compared treatment with RDV for 5 days (n = 191) and treatment with RDV for 10 days (n = 193) with SOC (n = 200). Participants treated with RDV received 200 mg on Day 1 and 100 mg once daily on subsequent days. The primary endpoint was clinical status on Day 11 assessed by a 7-point ordinal scale ranging from hospital discharge to increasing levels of oxygen and ventilatory support to death.

At baseline, the median age of participants was 57 years (range, 12 to 95 years); 61% were male, 61% were white, 19% were black, and 19% were Asian. Baseline clinical status, oxygen support status, and median duration of symptoms and hospitalization prior to first dose of RDV were similar across treatment groups.

	~~Overall, the odds of improvement in the ordinal scale were higher in the 5-day RDV group at Day 11 when compared to those receiving only SOC (odds ratio, 1.65; [95% CI, 1.09 to 2.48], p = 0.017). The odds of improvement in clinical status with the 10-day treatment group when compared to those receiving only SOC were not statistically significant (odds ratio 1.31; [95% CI 0.88 to 1.95]; p = 0.18). At Day 11, observed mortality rates for the 5-day, 10-day, and SOC groups were 0, 1%, and 2%, respectively. All-cause mortality at Day 28 was 2%, 1%, and 3% in the 5-day, 10-day, and SOC groups, respectively. The most common AEs are recorded in Table 2.~~<br><br>**~~Table 2. Most Common Adverse Events Reported for Participants Overall in Study 5774~~**<br><br>~~n (%)~~ | ~~5 Days N=200~~ | ~~10 Days N=193~~ | ~~SOC N=200~~<br>~~Nausea~~ | ~~19 (10)~~ | ~~18 (9)~~ | ~~6 (3)~~<br><br>~~1.2.2.4. Integrated Safety Findings from Clinical Studies~~<br><br>~~Assessment of adverse reactions is based on data from four Phase 1 studies in 138 healthy adult participants, 4 Phase 3 studies in 1467 hospitalized participants with COVID-19, and from 163 hospitalized participants with COVID-19 who received RDV in a compassionate use program.~~<br><br>~~The adverse reactions are listed below by system organ class and frequency. Frequencies are defined as follows: very common (≥ 10%), common (≥ 1% and <10%), uncommon (≥ 0.1% and < 1%), or rare (≥ 0.01% and < 0.1%).~~<br><br>~~INJURY, POISONING AND PROCEDURAL COMPLICATIONS~~<br><br>~~Rare: infusion-related reaction~~<br><br>~~HEPATOBILIARY DISORDERS~~<br><br>~~Very common: transaminases increased~~<br><br>~~Description of selected adverse reactions~~<br><br>*~~Transaminases Increased~~*<br><br>~~In healthy volunteer studies, the incidence of increased transaminases was higher in participants who received RDV compared to placebo. In clinical studies of participants with COVID-19, the incidence of increased transaminases was similar in participants treated with RDV compared to placebo or SOC.~~<br><br>*~~Infusion-related Reactions~~*<br><br>~~Infusion-related reactions have been observed following administration of RDV. Signs and symptoms may include hypotension, hypertension, tachycardia, bradycardia, hypoxia, fever, dyspnea, wheezing, angioedema, rash, nausea, diaphoresis, and shivering.~~
**Rationale:**	Information is now contained in the current IB.

**Section:**	Protocol Synopsis, Section 2 Objectives, Section 8.1.1 Analysis Objectives
**Original Text:**	The primary objectives of this study are as follows:<br>• To evaluate the efficacy of RDV in reducing the rate of hospitalization or death when given over 3 days to non-hospitalized participants with early stage COVID-19.<br>• To evaluate the safety of RDV administered in an outpatient setting
**Revised Text:**	The primary objectives of this study are as follows:<br>• To evaluate the efficacy of RDV in reducing the rate of ***all-cause medically attended visits (MAVs; medical visits attended in person by the participant and a health care professional)*** ~~hospitalization~~ or death when given over 3 days to non-hospitalized participants with early stage COVID-19.<br>• To evaluate the safety of RDV administered in an outpatient setting
**Rationale:**	Primary objectives updated to align with updates to endpoints.

**Sections:**	Protocol Synopsis, Section 3.1 Endpoints, Section 8.1.2 Primary Endpoints, Section 8.1.3 Secondary Endpoints, 8.14 Other Endpoints of Interest
**Original Text:**	The primary endpoint of this study is as follows:<br>• Composite endpoint of hospitalization or death from any cause by Day 14<br>• The proportion of participants with treatment-emergent AEs<br>The secondary endpoints of this study are as follows:<br>• All-cause mortality at Day 28<br>• Rate of hospitalization by Day 28<br>• Time-weighted average change in SARS-CoV-2 viral load from baseline to Day 7<br>• Time to resolution of COVID-19-related symptoms<br>• Proportion of participants progressing to requiring oxygen supplementation by Day 28<br>The exploratory endpoints of the study are as follows:<br>• Time to symptom resolution of each domain of COVID-19 adapted FLU-PRO<br>• Change from baseline in COVID-19-adapted FLU-PRO total score and score in each domain<br>• Psychometric validity of COVID-19 adapted FLU-PRO questionnaire<br>• Time-weighted average change in SARS-CoV-2 viral load from baseline to Day 14<br>• Time to first negative SARS-CoV-2 polymerase chain reaction (PCR)<br>• Emergence of viral resistance to RDV

	• Baseline levels and change from baseline for inflammation/immune-related, acute respiratory distress syndrome (ARDS)-related and coagulation-related biomarkers<br>• Proportion of participants admitted to the intensive care unit by Day 28<br>• Proportion of participants started on mechanical ventilation by Day 28<br>• The plasma concentrations and PK parameters of RDV and metabolites
**Revised Text:**	The primary endpoint***s*** of this study ***are***~~ is~~ as follows:<br>• Composite endpoint of all-cause medically attended visits (MAVs) (medical visits attended in person by the participant and a health care professional)~~ hospitalization~~ or death ~~from any cause~~ by Day ~~14~~28<br>• ~~PThe p~~roportion of participants with treatment-emergent AEs<br>The secondary endpoints of this study are as follows:<br>• All-cause mortality at Day 28<br>• ~~Rate of hospitalization~~ ***Proportion of participants hospitalized*** by Day 28<br>• ***Composite endpoint of all-cause MAVs (medical visits attended in person by the participant and a health care professional) or death by Day 14***<br>• Time-weighted average change in SARS-CoV-2 viral load from baseline to Day 7<br>~~• Time to resolution of COVID-19-related symptoms~~<br>• Proportion of participants progressing to requiring oxygen supplementation by Day 28<br>The exploratory endpoints of the study are as follows:<br>• Time to symptom ***alleviation (mild or absent) of COVID-19 symptoms and in*** ~~resolution of~~ each domain of the COVID-19-adapted FLU-PRO ***Plus***<br>• Change from baseline in COVID-19-adapted FLU-PRO ***Plus*** total score and score in each domain<br>• Psychometric validity of COVID-19 adapted FLU-PRO ***Plus*** questionnaire<br>• Time-weighted average change in SARS-CoV-2 viral load from baseline to Day 14<br>• Time to first negative SARS-CoV-2 polymerase chain reaction (PCR)<br>• ***Proportion of participants with negative SARS-CoV-2 PCR at each study visit***<br>• Emergence of viral resistance to RDV

	• Baseline levels and change from baseline for inflammation/immune-related, acute respiratory distress syndrome (ARDS)-related and coagulation-related biomarkers<br>• Proportion of participants admitted to the intensive care unit by Day 28<br>• Proportion of participants started on mechanical ventilation by Day 28<br>• The plasma concentrations and PK parameters of RDV and metabolites
**Rationale:**	The endpoints of the study have been updated to better reflect the current burden of clinical care of participants. The proportion of care of COVID-19 patients who are hospitalized appears to have decreased since the study was designed; care of those with risk factors for progressing to more severe disease is occurring more frequently in an outpatient setting. Data from the CDC COVIDView report shows weekly hospitalization rates for COVID-19 per 100,000 population are down approximately 75% as of 08 October 2020 compared to April 2020.[1] The COVIDView report also demonstrates that the COVID-19 hospitalization rate per 100,000 population is lower amongst all age groups, even those 65 years and older who are known to be at higher risk for hospitalization from COVID-19. Interim data from monoclonal antibody trials released this fall show a low MAV rate of < 10%. The placebo group of Lilly's BLAZE-1 Phase 2 study experienced a hospitalization rate of 6.3% by Day 28,[2] which is 50% lower than the 14% US COVID-19 hospitalization rate reported in spring 2020.[3] The placebo group of Regeneron's Phase 2 monoclonal antibody study experienced a MAV rate of 6.5% by Day 29 (MAV defined as telemedicine, outpatient, urgent care, emergency room visits, and hospitalization). In light of these changing clinical patterns, we have broadened the primary endpoint in the current outpatient RDV trial to a composite of all-cause MAVs (medical visits attended in person by the participant and a health care professional) or death by Day 28.<br>References:<br>1. Centers for Disease Control and Prevention (CDC). Coronavirus Disease 2019 (COVID-19) COVIDView: Key Updates for Week 43, ending October 24, 2020. Available at: https://www.cdc.gov/coronavirus/2019-ncov/covid-data/covidview/index.html. Accessed: 06 November 2020. Last Updated: 30 October 2020.<br>2. Chen P, Nirula A, Heller B, Gottlieb RL, Boscia J, Morris J, et al. SARS-CoV-2 Neutralizing Antibody LY-CoV555 in Outpatients with Covid-19. N Engl J Med 2020.<br>3. Stokes EK, Zambrano LD, Anderson KN, Marder EP, Raz KM, El Burai Felix S, et al. Coronavirus Disease 2019 Case Surveillance - United States, January 22-May 30, 2020. MMWR. Morbidity and mortality weekly report 2020;69 (24):759-65.<br>4. Regeneron. REGN-COV2 Antibody Cocktail Program Update. Available at: https://investor.regeneron.com/events-and-presentations. 29 September. 2020:

Section:	Section 3.5 Discontinuation Criteria
Original Text:	**3.5 Discontinuation Criteria**<br>Study drug dosing in an individual participant will be placed on hold and may be discontinued, following a review of all available clinical data by the medical monitor and discussion with the investigator, if any of the following occurs:<br>• Any serious AE (SAE) or ≥ Grade 3 AE suspected to be related to RDV<br>• Any elevations in ALT or AST ≥ 5 × upper limit of normal (ULN); or ALT > 3 × ULN and total bilirubin > 2 × ULN, confirmed by immediate repeat testing<br>• Infusion-related systemic reaction ≥ Grade 2 or infusion-related localized reaction ≥ Grade 3<br>Discontinuation of study medication is not a seriousness criterion. Participants who discontinue the study medication should be encouraged to remain in the study and attend study visits as described in Sections 6.3, 6.4, and 6.5.
Revised Text:	N/A
Rationale:	Removed section, as the information presented is more clearly noted in Section 6.8 Criteria for Discontinuation of Study Treatment

Section:	Protocol Synopsis, Section 4.2 Inclusion Criteria
Original Text:	**4.2 Inclusion Criteria**<br>Participants must meet all of the following inclusion criteria to be eligible for participation in this study:<br>1) Willing and able to provide written informed consent, or with a legal representative who can provide informed consent (participants ≥ 18 years of age) or assent (participants ≥ 12 and < 18 years of age) prior to performing study procedures. For participants ≥ 12 and < 18 years of age, a parent or legal guardian must be willing and able to provide written informed consent prior to performing study procedures<br>2) Either:<br>Age ≥ 18 years (at all sites) or aged ≥ 12 and < 18 years of age weighing ≥ 40 kg (where permitted according to local law and approved nationally and by the relevant institutional review board [IRB] or independent ethics committee [IEC]) with at least 1 of the following pre-existing risk factors for progression to hospitalization:<br>a) Chronic lung disease: chronic obstructive pulmonary disease, moderate-to-severe asthma, cystic fibrosis, pulmonary fibrosis<br>b) Hypertension: systemic or pulmonary<br>c) Cardiovascular or cerebrovascular disease: coronary artery disease, congenital heart disease, heart failure, cardiomyopathy, history of stroke

	d) Diabetes mellitus: Type 1 or 2<br>e) Obesity (BMI ≥ 30)<br>f) Immunocompromised state<br>g) Chronic mild or moderate kidney disease<br>h) Chronic liver disease<br>i) Current cancer<br>j) Sickle cell disease<br>OR age ≥ 60 years, regardless of the presence of other pre-existing risk factors for progression<br>3) SARS-CoV-2 infection confirmed by PCR ≤ 4 days prior to screening<br>4) Presence of ≥ 1 symptom(s) consistent with COVID-19 for ≤ 7 days prior to randomization (such as fever, cough, fatigue, shortness of breath, sore throat, headache, myalgia/arthralgia) {Stokes 2020, World Health Organization (WHO) 2020b}<br>5) Oxygen saturation (SpO_2) > 94% on room air<br>6) Not currently requiring hospitalization<br>7) Participants of childbearing potential who engage in heterosexual intercourse must agree to use protocol-specified method(s) of contraception as described in Appendix 3.
**Revised Text:**	**4.2 Inclusion Criteria**<br>Participants must meet all of the following inclusion criteria to be eligible for participation in this study:<br>1) Willing and able to provide written informed consent~~, or with a legal representative who can provide informed consent~~ (participants ≥ 18 years of age) or assent (participants ≥ 12 and < 18 years of age) prior to performing study procedures. ***Participants aged ≥ 18 years may be enrolled with the consent of a legal representative where permitted according to local law and approved nationally and by the relevant institutional review board (IRB) or independent ethics committee (IEC).*** For participants ≥ 12 and < 18 years of age, a parent or legal guardian must be willing and able to provide written informed consent prior to performing study procedures<br>2) Either:<br>Age ≥ 18 years (at all sites) or aged ≥ 12 and < 18 years of age weighing ≥ 40 kg (where permitted according to local law and approved nationally and by the relevant ~~institutional review board [~~IRB~~]~~ or ~~independent ethics committee [~~IEC~~]~~) with at least 1 of the following pre-existing risk factors for progression to hospitalization:<br>a) Chronic lung disease: chronic obstructive pulmonary disease, moderate-to-severe asthma, cystic fibrosis, pulmonary fibrosis<br>b) Hypertension: systemic or pulmonary

	c) Cardiovascular or cerebrovascular disease: coronary artery disease, congenital heart disease, heart failure, cardiomyopathy, history of stroke, ***atrial fibrillation, hyperlipidemia***<br>d) Diabetes mellitus: Type 1***,*** ~~or~~ ***type*** 2***, or gestational***<br>e) Obesity (BMI ≥ 30)<br>f) Immunocompromised state***; having a solid organ transplant, blood, or bone marrow transplant; immune deficiencies; HIV with a low CD4 cell count or not on HIV treatment; prolonged use of corticosteroids; or use of other immune weakening medicines***<br>g) Chronic mild or moderate kidney disease<br>h) Chronic liver disease<br>i) Current cancer<br>j) Sickle cell disease<br>OR age ≥ 60 years, regardless of the presence of other pre-existing risk factors for progression<br>3) SARS-CoV-2 infection confirmed by ~~PCR~~ ***molecular diagnostics (nucleic acid [eg, PCR]or antigen testing)*** ≤ 4 days prior to screening<br>4) Presence of ≥ 1 symptom(s) consistent with COVID-19 for ≤ 7 days prior to randomization (such as fever, cough, fatigue, shortness of breath, sore throat, headache, myalgia/arthralgia) {Stokes 2020, World Health Organization (WHO) 2020b}<br>5) ~~Oxygen saturation (SpO_2) > 94% on room air~~ ***Not currently receiving, requiring, or expected to require supplemental oxygen***<br>6) Not currently requiring hospitalization ***(hospitalization defined as ≥ 24 hours of acute care)***<br>7) Participants of childbearing potential who engage in heterosexual intercourse must agree to use protocol-specified method(s) of contraception as described in Appendix 3
**Rationale:**	Revised inclusion criteria for clarity. By changing the method of SARS-CoV-2 confirmation to "by molecular diagnostics," sites can now utilize either antigen testing or PCR to confirm infection.

**Section:**	Protocol Synopsis, Section 4.3 Exclusion Criteria
**Original Text:**	**4.3 Exclusion Criteria**<br>Participants who meet *any* of the following exclusion criteria are not eligible to be enrolled in this study:<br>1) Participation in any other clinical trial of an experimental treatment and prevention for COVID-19<br>2) Prior hospitalization for COVID-19

	3) Treatment with other agents with actual or possible direct antiviral activity against SARS-CoV-2<br>4) Use of hydroxychloroquine or chloroquine ≤ 7 days prior to screening<br>5) Requiring oxygen supplementation<br>6) ALT or AST ≥ 5 × ULN at screening or within 90 days of screening<br>*Note: if per local practice only ALT is routinely measured, exclusion criteria will be evaluated on ALT alone*<br>7) Creatinine clearance < 30 mL/min at screening or within 90 days of screening using the Cockcroft-Gault formula in participants ≥ 18 years of age or the Schwartz formula in participants < 18 years of age (see Section 6.6.1)<br>8) Currently breastfeeding (nursing)<br>9) Known hypersensitivity to the study drug, the metabolites, or formulation excipient<br>10) Use or planned use of exclusionary medications, refer to Section 5.4
**Revised Text:**	**4.3 Exclusion Criteria**<br>Participants who meet *any* of the following exclusion criteria are not eligible to be enrolled in this study:<br>1) Participation in any other clinical trial of an experimental treatment and prevention for COVID-19<br>2) Prior hospitalization for COVID-19 ***(hospitalization defined as ≥ 24 hours of acute care)***<br>3) Treatment with other agents with actual or possible direct antiviral activity against SARS-CoV-2<br>~~4)~~ ***Criterion removed*** ~~Use of hydroxychloroquine or chloroquine ≤ 7 days prior to screening~~<br>~~5)~~ Requiring oxygen supplementation<br>6) ALT or AST ≥ 5 × ***upper limit of normal (***ULN***)*** at screening or within 90 days of screening<br>*Note: if per local practice only ALT is routinely measured, exclusion criteria will be evaluated on ALT alone*<br>7) Creatinine clearance < 30 mL/min at screening or within 90 days of screening using the Cockcroft-Gault formula in participants ≥ 18 years of age or ***< 30 mL/min/1.73m^2 at screening or within 90 days of screening using*** the Schwartz formula in participants < 18 years of age (see Section 6.7.2)<br>8) Currently breastfeeding (nursing)<br>9) Known hypersensitivity to the study drug, the metabolites, or formulation excipient<br>10) Use or planned use of exclusionary medications, refer to Section 5.4

**Rationale:**	Revised exclusion criterion for clarity and to remove hydroxychloroquine or chloroquine usage requirements prior to study participation.

**Section:**	Section 5.3.1 Administration Instructions
**Original Text:**	The prepared diluted solution should not be administered simultaneously with any other medication. The compatibility of RDV injection with IV solutions and medications other than 0.9% sodium chloride is not known. Administer the diluted solution with the infusion rate described in Table 3.
**Revised Text:**	The prepared diluted solution should not be administered simultaneously with any other medication. The compatibility of RDV injection with IV solutions and medications other than 0.9% sodium chloride is not known. ***Administer RDV via IV infusion over 30 minutes. Slower infusion rates of up to 120 minutes can be considered to potentially prevent signs and symptoms of infusion related reaction. Infusion rates for different infusion volumes and times are*** ~~Administer the diluted solution with the infusion rate~~ described in Table *1*~~3~~.
**Rationale:**	To clarify preferred infusion administration time.

**Section:**	Section 5.4 Prior and Concomitant Medications
**Original Text:**	**5.4 Prior and Concomitant Medications**<br>Concomitant use of the following is prohibited in participants receiving RDV:<br>• Investigational agents for COVID-19 including approved HIV protease inhibitors such as lopinavir/RTV, chloroquine, interferon, etc.<br>• Use of hydroxychloroquine or chloroquine within 7 days of randomization<br>• Strong inducers of P-glycoprotein (e.g., rifampin or herbal medications)<br>Medications will be assessed from screening to the Day 28 Follow-up visit.
**Revised Text:**	**5.4 Prior and Concomitant Medications**<br>***Concomitant medications taken within 30 days prior to screening and up to and including 30 days after the last dose of study drug need to be recorded in the source documents and eCRFs.***<br>Concomitant use of the following is prohibited in participants receiving RDV:<br>• Investigational ***or approved*** agents for ***the SARS-CoV-2 virus*** ~~COVID-19~~ including approved HIV protease inhibitors such as lopinavir/~~RTV~~ ***ritonavir***, ~~chloroquine,~~ interferon, etc. ***Use of these medications for an approved indication other than SARS-CoV-2 infection is not prohibited***<br>• Use of hydroxychloroquine or chloroquine ~~within 7 days of randomization~~ ***for any indication***<br>• Strong inducers of P-glycoprotein (~~e.g.,~~, rifampin or herbal medications)

**Rationale:**	To clarify study documentation and specifics of prior and concomitant medications.

**Section:**	Section 6.3 Treatment Assessments (Baseline/Day 1, Day 2, and Day 3)
**Original Text:**	The following evaluations are to be completed at the Day 1 visit. The investigator must have confirmed eligibility before proceeding with randomization and the Day 1 visit. If the screening and Day 1 visits occur on the same day, no procedures need to be repeated. Participants must complete the following assessments before being administered study drug:<br>• Vital signs (heart rate, temperature, blood pressure) pre-infusion, post-infusion, and when post infusion observation is completed<br>• Body weight<br>• Respiratory status:<br>Respiratory rate<br>Oxygenation: SpO_2 on room air<br>• Complete physical examination (Day 1)<br>• Symptom-directed physical examination (Day 2, Day 3)<br>• Review of AEs and concomitant medications<br>• Obtain blood samples as described in Section 6.6 (Day 1, Day 3)<br>• Nasopharyngeal swab samples for SARS-CoV-2 quantitative reverse transcriptase polymerase chain reaction (RT-qPCR) testing and possible viral resistance testing<br>• Sputum sample collection from participants with productive cough for SARS-CoV-2 RT-qPCR testing and possible viral resistance testing<br>• Completion of the COVID-19-adapted FLU-PRO questionnaire (if available)<br>• Administration of study drug. Instructions on study drug administration are available in a separate manual.
**Revised Text:**	The following evaluations are to be completed at the Day 1 visit. The investigator must have confirmed eligibility before proceeding with randomization and the Day 1 visit. If the screening and Day 1 visits occur on the same day, no procedures need to be repeated ***except for clinical laboratory samples that must be collected locally to confirm eligibility. Clinical laboratory samples must be collected at Day 1 to be sent to the central laboratory, even if completed locally, as described in Section 6.7.2***. Participants must complete the following assessments before being administered study drug:<br>• Vital signs (heart rate, temperature, blood pressure) pre-infusion, postinfusion, and when postinfusion observation is completed<br>~~• Body weight~~<br>• Respiratory status:<br>Respiratory rate

	Oxygenation: SpO_2 on room air<br>• Complete physical examination (Day 1)<br>• ***Clinical*** ~~S~~*s*ymptom-directed physical examination (Day 2, Day 3)<br>• Review of AEs and concomitant medications<br>• ***Review any interactions with health care professionals, other than study staff or designees, including hospitalization; in-person emergency, urgent, or primary care visits; or any other in-person visit attended by the participant and a health care profession. Identify the nature and cause of the visit.***<br>• Obtain blood samples as described in Section 6.7.2 (Day 1, Day 3)<br>• Nasopharyngeal swab samples for SARS-CoV-2 quantitative reverse transcriptase polymerase chain reaction (RT-qPCR) testing and possible viral resistance testing<br>• Sputum sample collection from participants with productive cough for SARS-CoV-2 RT-qPCR testing and possible viral resistance testing<br>• Completion of the COVID-19-adapted FLU-PRO ***Plus*** questionnaire (if available)<br>• Administration of study drug. Instructions on study drug administration are available in a separate manual.
**Rationale:**	To clarify laboratory samples requirements when screening labs for eligibility are performed on the same day as a Day 1 visit. To include additional assessment that will specifically delineate the data to be collected to allow the composite endpoint of all medically attended visits to be evaluated.

**Section:**	Section 6.4 Post-treatment Assessments (Day 4 Through Day 14),<br>Section 6.5 Day 28 Follow-up Assessment (± 5 Days)
**Original Text:**	N/A
**Revised Text:**	***Review MAV information, including any interactions with health care professionals, other than study staff or designees, including hospitalization; in-person emergency, urgent, or primary care visits; or any other in-person visit attended by the participant and a health care profession. Identify the nature and cause of the visit.***
**Rationale:**	Text updated to specifically delineate the data to be collected to allow the composite endpoint of all medically attended visits to be evaluated.

**Section:**	Section 6.3 Treatment Assessments (Baseline/Day 1, Day 2, and Day 3), Section 7.6 Toxicity Management
**Original Text:**	Remdesivir infusions will be administered to participants at the site under close supervision or in the participant's home by a home health service provider. Health care professionals administering RDV infusions should have the appropriate medication available for immediate use in case of hypersensitivity or infusion-related reactions. The participant should be treated according to the SOC for management of hypersensitivity reaction or infusion-related reactions. Post infusion monitoring should be done according to site or home health protocol.
**Revised Text:**	Remdesivir infusions will be administered to participants at the site under close supervision or in the participant's home by a home health service provider. Health care professionals administering RDV infusions ***must*** ~~should~~ have the appropriate medication available for immediate use in case of hypersensitivity or infusion-related reactions. The participant should be treated according to the SOC for management of hypersensitivity reaction or infusion-related reactions. Post-infusion monitoring should be done according to site or home health protocol. ***All information related to home administration of RDV will be provided to the investigator by the home health provider, wherever applicable, in a timely manner.***
**Rationale:**	To clarify home health specifics and to address regulatory agencies' comments.

**Section:**	Section 6.7.1 Complete Physical Examination
**Original Text:**	N/A
**Revised Text:**	***A complete physical examination must include source documentation of general appearance and the following body systems: Head, neck, and thyroid; eyes, ears, nose, throat, mouth, and tongue; chest (excluding breasts); respiratory; cardiovascular; lymph nodes, abdomen; skin, hair, nails; musculoskeletal; and neurological. Urogenital and reproductive examination should only be completed if clinically indicated.***
**Rationale:**	Added section to clarify complete physical examination specifics.

**Section:**	Section 6.7.2 Clinical Laboratory Assessments
**Original Text:**	Blood will be collected throughout the study as outlined below and in Appendix 2 Study Procedures Table.<br>**6.6.1 Blood Samples**<br>Blood sample collection for the following laboratory analyses will be performed at the specified time points:<br>• Chemistry profile: alkaline phosphatase, AST, ALT, total bilirubin, total protein, albumin, lactate dehydrogenase, creatine phosphokinase, bicarbonate, blood urea nitrogen, calcium, chloride, creatinine, glucose, lipase, magnesium, phosphorus, potassium, sodium, uric acid (Day 1, Day 3, Day 7, Day 14)

	• Estimated glomerular filtration rate (screening [if required] Day 1, Day 3, Day 7, Day 14) according to:<br>Schwartz formula for participants < 18 years of age, where S_{Cr} is serum creatinine (mg/dL)<br>Adolescent boys ≥ 12 years of age: 0.70 × L/ S_{Cr} (L is height in cm)<br>Adolescent girls ≥ 12 years of age: 0.55 × L/ S_{Cr} (L is height in cm)<br>Cockcroft-Gault formula for creatinine clearance (CL_{cr}) for participants ≥ 18 years of age<br>Men: $\frac{(140 - \text{age in years}) \times (\text{wt in kg})}{72 \times (\text{serum creatinine in mg/dL})}$ CL_{cr} (mL/min)<br>Women: $\frac{(140 - \text{age in years}) \times (\text{wt in kg}) \times 0.85}{72 \times (\text{serum creatinine in mg/dL})}$ CL_{cr} (mL/min)<br>• Hematology profile: complete blood count with differential (Day 1, Day 3, Day 7, Day 14)<br>• Optional genomic testing (Day 1 or at any time during the study, if additional consent for genomics testing is obtained)
**Revised Text:**	~~**6.6.1 Blood Samples**~~<br>***6.7.2 Clinical Laboratory Assessments***<br>~~Blood will be collected throughout the study as outlined below and in Appendix 2 Study Procedures Table.~~<br>Blood sample collection for the following laboratory analyses will be performed at the specified time points:<br>• Chemistry profile: alkaline phosphatase, AST, ALT, total bilirubin, total protein, albumin, lactate dehydrogenase, creatine phosphokinase, bicarbonate, blood urea nitrogen, calcium, chloride, creatinine, glucose, lipase, magnesium, phosphorus, potassium, sodium, uric acid (Day 1, Day 3, Day 7, Day 14)<br>• Estimated glomerular filtration rate (screening [if required] Day 1, Day 3, Day 7, Day 14) according to:<br>Schwartz formula for participants < 18 years of age, where S_{Cr} is serum creatinine (mg/dL)<br>Adolescent boys ≥ 12 years of age: 0.70 × L/ S_{Cr} (L is height in cm)<br>Adolescent girls ≥ 12 years of age: 0.55 × L/ S_{Cr} (L is height in cm)<br>***Height at baseline will be used for all calculations using the Schwartz formula.***<br>Cockcroft-Gault formula for creatinine clearance (CL_{cr}) for participants ≥ 18 years of age. ***Weight at screening will be used for Days 1-3.***<br>Men: $\frac{(140 - \text{age in years}) \times (\text{wt in kg})}{72 \times (\text{serum creatinine in mg/dL})}$ CL_{cr} (mL/min)

	Women: $\frac{(140 - \text{age in years}) \times (\text{wt in kg}) \times 0.85}{72 \times (\text{serum creatinine in mg/dL})} = CL_{cr}$ (mL/min)<br>• Hematology profile: complete blood count with differential (Day 1, Day 3, Day 7, Day 14)<br>• ***Coagulation: international normalized ratio (INR), prothrombin time (PT), activated partial thromboplastin time (APTT) (Day 1, Day 3, Day 7, Day 14)***<br>• Optional genomic testing (Day 1 or at any time during the study, if additional consent for genomics testing is obtained)
**Rationale:**	Addition of coagulation panel to monitor potential increases in international normalized ratio, prothrombin time, activated partial thromboplastin time. Height and weight details added for clarity.

**Section:**	Section 6.7.4 Biomarker Assessments (Optional)
**Original Text:**	Optional blood samples will be collected to measure serum and plasma biomarkers and may include immune-related markers, ARDS-related, and coagulation-related biomarkers and sACE2. DNA extraction for genetic analysis may be performed to evaluate single nucleotide polymorphisms which may be associated with host viral response, immune response, coagulation-related disorders and respiratory disorders.<br>• Serum (Days 1, 3, 14)<br>Immune-related biomarkers (IL-6, CRP, ferritin, procalcitonin)<br>ARDS-related (ang2)<br>• Plasma (Days 1, 3, 14)<br>Abnormal coagulation-related biomarkers (D-dimer, PT, aPTT, fibrinogen)
**Revised Text:**	Optional blood samples will be collected to measure serum and plasma biomarkers and may include immune-related markers, ARDS-related, and coagulation-related biomarkers~~ and sACE2~~. DNA extraction for genetic analysis may be performed to evaluate single nucleotide polymorphisms which may be associated with host viral response, immune response, coagulation-related disorders and respiratory disorders.<br>• Serum (Days 1, 3, 14)<br>Immune-related biomarkers (IL-6, CRP, ferritin, procalcitonin)<br>ARDS-related (ang2)<br>• Plasma (Days 1, 3, 14)<br>Abnormal coagulation-related biomarkers (D-dimer, ~~PT, aPTT,~~ fibrinogen)
**Rationale:**	To remove PT and aPTT time from the optional biomarkers panel as it will now be included as part of required testing.

**Section:**	Section 6.8 Criteria for Discontinuation of Study Treatment
**Original Text:**	Study medication may be discontinued in the following instances:<br>• Intercurrent illness that would, in the judgment of the investigator, affect assessments of clinical status to a significant degree. Following resolution of intercurrent illness, the participant may resume study dosing at the discretion of the investigator.<br>• Unacceptable toxicity, or toxicity that, in the judgment of the investigator, compromises the ability to continue study-specific procedures or is considered to not be in the participant's best interest<br>• Individual participant may be discontinued if they experience an ALT or AST ≥ 5 × ULN; or ALT > 3 × ULN and total bilirubin > 2 × ULN<br>• Other Grade 3 or Grade 4 abnormal laboratory results related to RDV<br>• Lack of efficacy<br>• Participant request to discontinue for any reason<br>• Participant noncompliance<br>• Discontinuation of the study at the request of Gilead, a regulatory agency or an IRB or independent ethics committee (IEC)<br>• Infusion-related systemic reaction ≥ Grade 2 or infusion-related localized reaction ≥ Grade 3
**Revised Text:**	Study medication ~~may~~ ***will*** be discontinued ***for individual participants*** in the following instances***, unless the potential for resuming dosing is specifically noted:***<br>• Intercurrent illness that would, in the judgment of the investigator, affect assessments of clinical status to a significant degree. Following resolution of intercurrent illness, the participant may resume study dosing at the discretion of the investigator.<br>• Unacceptable toxicity, or toxicity that, in the judgment of the investigator, compromises the ability to continue study-specific procedures or is considered to not be in the participant's best interest<br>• ~~Individual participant may be discontinued if they experience an~~ ALT or AST ≥ 5 × ULN; or ALT > 3 × ULN and total bilirubin > 2 × ULN***, confirmed by immediate repeat testing***<br>• Other Grade 3 or Grade 4 abnormal laboratory results related to RDV<br>• Lack of efficacy<br>• Participant request to discontinue for any reason<br>• Participant noncompliance<br>• Discontinuation of the study at the request of Gilead, a regulatory agency or an IRB or ~~independent ethics committee (~~IEC~~)~~<br>• Infusion-related systemic reaction ≥ Grade 2 or infusion-related localized reaction ≥ Grade 3
**Rationale:**	To clarify and to address regulatory agencies' requirements.

**Section:**	8.3.1.1 Efficacy
**Original Text:**	The primary analysis set for efficacy analysis is defined as the Full Analysis Set, which will include all participants who (1) are randomized into the study and (2) have received at least 1 dose of study treatment if randomized to the RDV treatment group. Participants will be grouped according to the treatment to which they were randomized.
**Revised Text:**	The primary analysis set for efficacy analysis is defined as the Full Analysis Set, which will include all participants who (1) are randomized into the study***,*** ~~and~~ (2) have received at least 1 dose of study treatment, ***and (3) enrolled under protocol amendment 2.*** ~~if randomized to the RDV treatment group.~~ Participants will be grouped according to the treatment to which they were randomized.
**Rationale:**	Updated to exclude patients who will not have data collected for MAV.

**Section:**	Protocol Synopsis, 8.5.1 Primary Analysis, 8.52 Secondary Analyses
**Original Text:**	**8.5.1 Primary Analysis**<br>The primary endpoint of the study is the composite endpoint of hospitalization or death from any cause by Day 14. The null hypothesis being tested is whether the ratio of proportion of hospitalization or death is the same for the 2 treatment groups (i.e., whether ratio is equal to 1 across all strata).<br>The proportion of participants hospitalized or who died from the date of randomization up to Day 14 will be estimated using Kaplan-Meier methods for each stratification stratum. The hypothesis test (p-value) will be calculated in following steps:<br>1. The difference between treatment groups in cumulative proportion in log scale will be calculated for each stratification stratum.<br>2. Variance for the difference of proportion between treatment groups will be estimated using Greenwood's formula.<br>3. The differences for each stratum will be combined, weighted by the inverse of the variance of the differences for each stratification stratum, into overall difference between 2 treatment groups.<br>4. P-value for the test of no difference between treatment groups will be calculated.<br>Participants with missing outcomes for the primary endpoint due to prematurely discontinuation of the study will be censored at the date of last contact.

	**8.5.2 Secondary Analyses**<br>The secondary endpoint of hospitalization by Day 28 will be analyzed similarly as in the primary analysis. The all-cause mortality at Day 28 will be compared between the 2 treatment groups using a *Fisher* exact test. The proportion of participants progressing to requiring oxygen supplementation by Day 28 will be estimated using the Kaplan-Meier method and compared between the 2 treatment groups using a log-rank test. Other endpoints of interest related to proportion of participants will be compared between treatment groups using a chi-square test or Fisher exact test. Endpoints that are measured as time to first event will be compared between treatment groups using the log-rank test and continuous endpoints will be compared between treatment groups using a Wilcoxon rank sum test or analysis of variance model.
**Revised Text:**	**8.5.1 Primary Analysis**<br>The primary endpoint of the study is the composite endpoint of ~~hospitalization~~ ***all-cause MAV*** or death ~~from any cause~~ by Day ***28*** ~~14~~. The null hypothesis being tested is whether the ratio of proportion of ***MAV*** ~~hospitalization~~ or death is the same for the 2 treatment groups (i~~.e.~~, whether ratio is equal to 1 across all strata).<br>~~The proportion of participants hospitalized or who died from the date of randomization up to Day 14 will be estimated using Kaplan-Meier methods for each stratification stratum. The hypothesis test (p-value) will be calculated in following steps:~~<br>***The hazard ratio, p-value, and 95% CI for the hazard ratio will be calculated using a Cox model with stratification factors as covariates.***<br>~~1. The difference between treatment groups in cumulative proportion in log scale will be calculated for each stratification stratum.~~<br>~~2. Variance for the difference of proportion between treatment groups will be estimated using Greenwood's formula.~~<br>~~3. The differences for each stratum will be combined, weighted by the inverse of the variance of the differences for each stratification stratum, into overall difference between 2 treatment groups.~~<br>~~4. P-value for the test of no difference between treatment groups will be calculated.~~<br>Participants with missing outcomes for the primary endpoint due to prematurely discontinuation of the study will be censored at the date of last contact.

	**8.5.2 Secondary Analyses**<br>The secondary endpoint of ~~hospitalization~~ ***MAV or death*** by Day ~~28~~***14*** will be analyzed similarly as in the primary analysis. The all-cause mortality at Day 28 will be compared between the 2 treatment groups using a Fisher exact test. The ***proportion of participants hospitalized by Day 28 and the*** proportion of participants progressing to requiring oxygen supplementation by Day 28 will be estimated using the Kaplan-Meier method and compared between the 2 treatment groups using a log-rank test. Other endpoints of interest related to proportion of participants will be compared between treatment groups using a chi-square test or Fisher exact test. Endpoints that are measured as time to first event will be compared between treatment groups using the log-rank test and continuous endpoints will be compared between treatment groups using a Wilcoxon rank sum test or analysis of variance model.
**Rationale:**	Updated to align with new and modified endpoints.

**Section:**	Protocol Synopsis, 8.9 Sample Size, 8.9.1 Sample Size Re-estimation
**Original Text:**	**8.9 Sample Size**<br>The sample size computation is based on ratio rate of hospitalization or death by Day 14 for the placebo treatment group. The ratio represents the rate of hospitalization or death for the RDV treatment group relative to the placebo treatment group. The sample size needed to detect a given ratio for a 1:1 randomization using a 2-tailed test at level α is given by:<br>$$\frac{(._{\alpha/2} + z_{\beta})^2}{(\phi - 1)^2 p_2^2}[p_1(1 - p_1) + p_2(1 - p_2)]$$<br>Where ϕ is the ratio (p_1/p_2), p_1 and p_2 are the rate of hospitalization or death for each treatment group, and $z_{\alpha/2}$ and z_{β} are the 1- α /2 and β quantiles of the standard normal distribution.<br>A sample size of 1230 participants (615 in each group with 1:1 randomization) achieves > 90% power to detect a ratio of 0.65 (RDV to placebo hospitalization or death rate) using a two-sided significance level of 0.05 assuming hospitalization or death rate is 20% in the placebo group and a 5% drop out rate. The power is expected to be similar under the primary analysis using the Kaplan-Meier methods incorporating stratification factors.<br>The sample size calculation was done using software PASS (Version 14.0, module of Tests for Two Proportions).

Revised Text:

8.9 Sample Size

The total sample size of the study will be approximately 1264 participants, including 60 participants currently enrolled in the study and the number of participants needed for the new primary endpoint under protocol amendment 2 (n = 1204).

The sample size computation is based on ***proportion*** ~~ratio rate~~ of ~~hospitalization~~ **MAV** or death by Day 14 for the ***RDV and*** placebo treatment group***s***. ~~The ratio represents the rate of hospitalization or death for the RDV treatment group relative to the placebo treatment group.~~ The sample size needed ~~to detect a given ratio~~ for a 1:1 randomization using a 2-tailed test at level α is given by:

$$\frac{(\cdot_{\alpha/2} + z_{\beta})^2}{(\phi - 1)^2 p_2^2}[p_1(1-p_1) + p_2(1-p_2)]$$

$$\frac{(z_{1-\alpha/2} + z_{1-\beta})^2}{P(1-R^2)\sigma^2 B^2}$$

Where ~~ϕ is the ratio (p_1/p_2), p_1 and p_2 are the rate of hospitalization or death for each treatment group, and $z_{\alpha/2}$ and z_{β} are the 1- α /2 and β~~ ***P is the overall rate of MAV or death , B is the log hazard ratio, σ is the standard deviation of treatment indicator variable, R is the R-Squared that is obtained when treatment indicator variable is regressed on the other covariates, and $z_{1-\alpha/2}$ and $z_{1-\beta}$ are the 1-α/2 and 1-β quantiles of the standard normal distribution.***

A sample size of 12~~30~~***04*** participants (6~~15~~**02** in each group with 1:1 randomization) achieves > 90% power to detect a ratio of 0.65 (RDV to placebo ***in proportion of MAV*** ~~hospitalization~~ or death ~~rate~~***, which is equals to a hazard ratio of 0.624)***) using a two-sided significance level of 0.05 assuming ***the overall MAV*** ~~hospitalization~~ or death rate is ***16.5% (***20% in the placebo group and ***13% in the RDV group)*** a 5% drop out rate. ~~The power is expected to be similar under the primary analysis using the Kaplan-Meier methods incorporating stratification factors.~~

The sample size calculation was done using software PASS (Version 14.0, module of ~~Tests for Two Proportions~~ ***Cox regression for survival***).

8.9.1 Sample Size Re-estimation

A sample size re-estimation is planned at the interim analysis with 50% participants having completed Day 28 visit due to the uncertainty in MAV or death rate in the placebo arm. All possible interim results will be partitioned into 3 zones – Favorable, Promising, and Unfavorable, depending on the size of observed conditional power (CP) under the current trend at the interim. If the CP falls into the Favorable zone, the trial will continue with the originally planned sample size of 1204 patients. If the CP falls into the Promising zone, the total sample size will be increased (up to 1500) according to the algorithm specified in the adaptation plan. If the CP falls into the Unfavorable zone but the interim result has not met the futility boundary, the trial will also continue with the originally planned sample size of 1204 patients.

	The DMC will review the unblinded data at the interim and communicate the decision on sample size to the study team. The overall type I error is controlled at 0.05 using the method proposed by {Müller and Schäfer (2004)}. Further details will be included in the DMC charter and an adaptation plan.
**Rationale:**	Updated to align with new primary endpoint and added sample size re-estimation for uncertainty in the placebo event rate.

**Section:**	Appendix 2. Study Procedures Table
**Original Text:**	N/A
**Revised Text:**	Addition of Coagulation Panel and Medically Attended Visit Information<br>Changes to/Addition of footnotes a, c, d, f, h, k, n, r, s, t, u<br>Correction of Optional Genomic Testing to be noted in *Baseline/Day 1* instead of *Screening* as previously noted.<br>Correction of Clinical Symptom Assessment to be noted only for visits noted in the Study Procedures section of the protocol.
**Rationale:**	To align with protocol amendments and to correct typographical errors.

"I have read and understand the above and agree to this protocol amendment as written."	
**Principal Investigator**	**Date**

333 Lakeside Drive
Foster City, CA 94404

PROTOCOL AMENDMENT SUMMARY OF CHANGES

PROTOCOL AMENDMENT #3

STUDY GS-US-540-9012
A Phase 3 Randomized, Double-Blind Placebo-Controlled Trial to Evaluate the Efficacy and Safety of Remdesivir (GS-5734™) Treatment of COVID-19 in an Outpatient Setting

**Original Protocol Date:**	21 July 2020
**Amendment 1 Date:**	11 August 2020
**Amendment 1.1 (United Kingdom):**	07 October 2020
**Amendment 1.1 (Germany):**	14 October 2020
**Amendment 2 Date:**	06 November 2020
**Amendment 3 Date:**	12 November 2020

**Rationale:**	Herein is a summary of the changes made to the protocol amendment #2 dated 06 November 2020 and reflected in Amendment #3 dated 12 November 2020.<br><br>Specific changes contained in Amendment #3 are presented herein as ***bold and italicized*** or ~~strikethrough~~.<br><br>The protocol synopsis, study procedures table, and all applicable sections were updated to align with above-mentioned changes in the protocol.<br><br>In addition, the opportunity was taken to correct typographical or grammatical errors.

**Global Changes:**	Removed all references to "symptom assessment" as a procedure as this will be captured via AE assessment, symptom-directed or complete physical exams and/or the COVID-19-adapted FLU-PRO Plus questionnaire.

**Sections:**	Protocol Synopsis, Section 3.1 Endpoints, Section 8.1.3 Secondary Endpoints, 8.14 Other Endpoints of Interest
**Original Text:**	The primary endpoints of this study are as follows:<br>• Composite endpoint of all-cause MAVs (medical visits attended in person by the participant and a health care professional) or death by Day 28<br>• Proportion of participants with treatment-emergent AEs

	The secondary endpoints of this study are as follows:<br>• All-cause mortality at Day 28<br>• Proportion of participants hospitalized by Day 28<br>• Composite endpoint of all-cause MAVs (medical visits attended in person by the participant and a health care professional) or death by Day 14<br>• Time-weighted average change in SARS-CoV-2 viral load from baseline to Day 7<br>• Proportion of participants progressing to requiring oxygen supplementation by Day 28<br>The exploratory endpoints of the study are as follows:<br>• Time to symptom alleviation (mild or absent) of COVID-19 symptoms and in each domain of the COVID-19-adapted FLU-PRO Plus<br>• Change from baseline in COVID-19-adapted FLU-PRO Plus total score and score in each domain<br>• Psychometric validity of COVID-19-adapted FLU-PRO Plus questionnaire<br>• Time-weighted average change in SARS-CoV-2 viral load from baseline to Day 14<br>• Time to first negative SARS-CoV-2 polymerase chain reaction (PCR)<br>• Proportion of participants with negative SARS-CoV-2 PCR at each study visit<br>• Emergence of viral resistance to RDV<br>• Baseline levels and change from baseline for inflammation/immune-related, acute respiratory distress syndrome (ARDS)-related and coagulation-related biomarkers<br>• Proportion of participants admitted to the intensive care unit by Day 28<br>• Proportion of participants started on mechanical ventilation by Day 28<br>• The plasma concentrations and PK parameters of RDV and metabolites
**Revised Text:**	The primary endpoints of this study are as follows:<br>• Composite endpoint of all-cause MAVs (medical visits attended in person by the participant and a health care professional) or death by Day 28<br>• Proportion of participants with treatment-emergent AEs<br>The secondary endpoints of this study are as follows:<br>• All-cause mortality at Day 28<br>• Proportion of participants hospitalized by Day 28<br>• Composite endpoint of all-cause MAVs (medical visits attended in person by the participant and a health care professional) or death by Day 14<br>• Time-weighted average change in SARS-CoV-2 viral load from baseline to Day 7<br>• ***Time to alleviation (mild or absent) of baseline COVID-19 symptoms as reported in the COVID-19-adapted FLU-PRO Plus***

	• Proportion of participants progressing to requiring oxygen supplementation by Day 28<br>The exploratory endpoints of the study are as follows:<br>• Time to ~~symptom~~ alleviation (mild or absent) of ***baseline*** ~~COVID-19~~ *symptoms* ~~and~~ in each domain of the COVID-19-adapted FLU-PRO Plus<br>• Change from baseline in COVID-19-adapted FLU-PRO Plus total score and score in each domain<br>• Psychometric validity of COVID-19-adapted FLU-PRO Plus questionnaire<br>• Time-weighted average change in SARS-CoV-2 viral load from baseline to Day 14<br>• Time to first negative SARS-CoV-2 polymerase chain reaction (PCR)<br>• Proportion of participants with negative SARS-CoV-2 PCR at each study visit<br>• Emergence of viral resistance to RDV<br>• Baseline levels and change from baseline for inflammation/immune-related, acute respiratory distress syndrome (ARDS)-related and coagulation-related biomarkers<br>• Proportion of participants admitted to the intensive care unit by Day 28<br>• Proportion of participants started on mechanical ventilation by Day 28<br>• The plasma concentrations and PK parameters of RDV and metabolites
**Rationale:**	The secondary endpoint of time to alleviation of COVID-19 symptoms was returned back to secondary from exploratory after further consideration. We believe reduction in symptom burden has potential to be a meaningful endpoint for patients.

**Section:**	Section 6.3 Treatment Assessments (Baseline/Day 1, Day 2, and Day 3), Section 7.6 Toxicity Management
**Original Text:**	Remdesivir infusions will be administered to participants at the site under close supervision or in the participant's home by a home health service provider. Health care professionals administering RDV infusions must have the appropriate medication available for immediate use in case of hypersensitivity or infusion-related reactions. The participant should be treated according to the SOC for management of hypersensitivity reaction or infusion-related reactions. Post infusion monitoring should be done according to site or home health protocol.

**Revised Text:**	Remdesivir infusions will be administered to participants at the site under close supervision or in the participant's home by a home health service provider. Health care professionals administering RDV infusions ***will*** ~~must~~ have the appropriate medication available for immediate use in case of hypersensitivity or infusion-related reactions. The participant should be treated according to the SOC for management of hypersensitivity reaction or infusion-related reactions. Post-infusion monitoring should be done according to site or home health protocol. All information related to home administration of RDV will be provided to the investigator by the home health provider, wherever applicable, in a timely manner.
**Rationale:**	To align with language utilized for UK country specific amendment.

**Section:**	Appendix 2. Study Procedures Table
**Original Text:**	N/A
**Revised Text:**	Correction of Vital Signs to be noted on *Day 28* to align with protocol language.<br>Removal of Clinical Symptom Assessment as a procedure and associated footnote.
**Rationale:**	To align with protocol amendments and to correct typographical errors.

"I have read and understand the above and agree to this protocol amendment as written."	
**Principal Investigator**	**Date**

333 Lakeside Drive
Foster City, CA 94404

PROTOCOL AMENDMENT SUMMARY OF CHANGES

PROTOCOL AMENDMENT #4

STUDY GS-US-540-9012

A Phase 3 Randomized, Double-Blind Placebo-Controlled Trial to Evaluate the Efficacy and Safety of Remdesivir (GS-5734™) Treatment of COVID-19 in an Outpatient Setting

**Original Protocol Date:**	21 July 2020
**Amendment 1 Date:**	11 August 2020
**Amendment 1.1 (United Kingdom):**	07 October 2020
**Amendment 1.1 (Germany):**	14 October 2020
**Amendment 2 Date:**	06 November 2020
**Amendment 3 Date:**	12 November 2020
**Amendment 4 Date:**	14 January 2021

**Rationale:**	Herein is a summary of the changes made to the protocol amendment #3 dated 12 November 2020 and reflected in amendment #4 dated 14 January 2021:<br><br>• Update to primary and secondary study objectives<br>• Update to primary and secondary study endpoints<br>• Update to exclusion criterion #3<br>• Update to study drugs' storage and handling requirements<br>• Update to statistical methods<br><br>Specific changes contained in Amendment #4 are presented herein as ***bold and italicized*** or ~~strikethrough~~.<br><br>The protocol synopsis and all applicable sections were updated to align with above-mentioned changes in the protocol.<br><br>In addition, the opportunity was taken to correct typographical or grammatical errors.

**Sections:**	Protocol Synopsis, Section 2 Objectives, Section 8.1.1 Analysis Objectives
**Original Text:**	The primary objectives of this study are as follows:<br>• To evaluate the efficacy of RDV in reducing the rate of all-cause medically attended visits (MAVs; medical visits attended in person by the participant and a health care professional) or death in non-hospitalized participants with early stage COVID-19<br>• To evaluate the safety of RDV administered in an outpatient setting<br>The secondary objectives of this study are as follows:<br>• To determine the antiviral activity of RDV on SARS-CoV-2 viral load<br>• To assess the impact of RDV on symptom duration and severity
**Revised Text:**	The primary objectives of this study are as follows:<br>• To evaluate the efficacy of RDV in reducing the rate of ***COVID-19 related hospitalization or*** all-cause ~~medically attended visits (MAVs; medical visits attended in person by the participant and a health care professional) or~~ death in non-hospitalized participants with early stage COVID-19<br>• To evaluate the safety of RDV administered in an outpatient setting<br>The secondary objectives of this study are as follows:<br>• ***To evaluate the efficacy of RDV in reducing the rate of COVID-19 related medically attended visits (MAVs; medical visits attended in person by the participant and a health care professional) or all-cause death in non-hospitalized participants with early stage COVID-19***<br>• To determine the antiviral activity of RDV on SARS-CoV-2 viral load<br>• To assess the impact of RDV on symptom duration and severity
**Rationale:**	The study objectives were updated to align with the updated study endpoints.

**Sections:**	Protocol Synopsis, Section 3.1 Endpoints, Section 8.1.2 Primary Endpoints, Section 8.1.3 Secondary Endpoints
**Original Text:**	The primary endpoints of this study are as follows:<br>• Composite endpoint of all-cause MAVs (medical visits attended in person by the participant and a health care professional) or death by Day 28<br>• Proportion of participants with treatment-emergent AEs<br>The secondary endpoints of this study are as follows:<br>• All-cause mortality at Day 28<br>• Proportion of participants hospitalized by Day 28

	• Composite endpoint of all-cause MAVs (medical visits attended in person by the participant and a health care professional) or death by Day 14<br>• Time-weighted average change in SARS-CoV-2 viral load from baseline to Day 7<br>• Time to alleviation (mild or absent) of baseline COVID-19 symptoms as reported on the COVID-19-adapted FLU-PRO Plus<br>• Proportion of participants progressing to requiring oxygen supplementation by Day 28
**Revised Text:**	The primary endpoints of this study are as follows:<br>• Composite endpoint of ***COVID-19 related hospitalization (defined as at least 24 hours of acute care) or*** all-cause ~~MAVs (medical visits attended in person by the participant and a health care professional) or~~ death by Day 28<br>• Proportion of participants with treatment-emergent AEs<br>The secondary endpoints of this study are as follows:<br>• ***Composite endpoint of COVID-19 related MAVs (medical visits attended in person by the participant and a health care professional) or all-cause death by Day 28***<br>• All-cause mortality at Day 28<br>• Proportion of participants hospitalized by Day 28<br>• ***Composite endpoint of COVID-19 related hospitalization (defined as at least 24 hours of acute care) or all-cause death by Day 14***<br>• Composite endpoint of ~~all-cause~~ ***COVID-19 related*** MAVs (medical visits attended in person by the participant and a health care professional) or ***all-cause*** death by Day 14<br>• Time-weighted average change in SARS-CoV-2 viral load from baseline to Day 7<br>• Time to alleviation (mild or absent) of baseline COVID-19 symptoms as reported on the COVID-19-adapted FLU-PRO Plus<br>• Proportion of participants progressing to requiring oxygen supplementation by Day 28
**Rationale:**	To address US regulatory agency comments.

<table>
<tr><td>Section:</td><td>Section 4.3 Exclusion Criteria</td></tr>
<tr><td>Original Text:</td><td>Participants who meet any of the following exclusion criteria are not eligible to be enrolled in this study:
1) Participation in any other clinical trial of an experimental treatment and prevention for COVID-19
2) Prior hospitalization for COVID-19 (hospitalization defined as ≥ 24 hours of acute care)
3) Treatment with other agents with actual or possible direct antiviral activity against SARS-CoV-2
4) Criterion removed
5) Requiring oxygen supplementation
6) ALT or AST ≥ 5 × upper limit of normal (ULN) at screening or within 90 days of screening
Note: if per local practice only ALT is routinely measured, exclusion criteria will be evaluated on ALT alone
7) Creatinine clearance < 30 mL/min at screening or within 90 days of screening using the Cockcroft-Gault formula in participants ≥ 18 years of age or estimated glomerular filtration rate (eGFR) < 30 mL/min/1.73m^2 at screening or within 90 days of screening using the Schwartz formula in participants < 18 years of age (see Section 6.7.2)
8) Currently breastfeeding (nursing)
9) Known hypersensitivity to the study drug, the metabolites, or formulation excipient
10) Use or planned use of exclusionary medications, refer to Section 5.4</td></tr>
<tr><td>Revised Text:</td><td>Participants who meet any of the following exclusion criteria are not eligible to be enrolled in this study:
1) Participation in any other clinical trial of an experimental treatment and prevention for COVID-19
2) Prior hospitalization for COVID-19 (hospitalization defined as ≥ 24 hours of acute care)
3) Treatment with other agents with actual or possible direct antiviral activity against SARS-CoV-2 or administration of any SARS-CoV-2 (or COVID-19) vaccine
4) Criterion removed
5) Requiring oxygen supplementation
6) ALT or AST ≥ 5 × upper limit of normal (ULN) at screening or within 90 days of screening</td></tr>
</table>

	Note: if per local practice only ALT is routinely measured, exclusion criteria will be evaluated on ALT alone<br>7) Creatinine clearance < 30 mL/min at screening or within 90 days of screening using the Cockcroft-Gault formula in participants ≥ 18 years of age or estimated glomerular filtration rate (eGFR) < 30 mL/min/1.73m^2 at screening or within 90 days of screening using the Schwartz formula in participants < 18 years of age (see Section 6.7.2)<br>8) Currently breastfeeding (nursing)<br>9) Known hypersensitivity to the study drug, the metabolites, or formulation excipient<br>10) Use or planned use of exclusionary medications, refer to Section 5.4
**Rationale:**	To clarify exclusion of COVID-19 vaccines.

**Section:**	Section 5.2.3 Storage and Handling
**Original Text:**	Remdesivir for injection and PTM, 100 mg, vials should be stored below 30 °C (86 °F) prior to use. Storage conditions are specified on the label. Until dispensed for dosing, all vials of study drugs should be stored in a securely locked area, accessible only to authorized site personnel.<br>To ensure the sterility, stability, and proper identification, study drug(s) should not be stored in a container other than the container in which they were supplied.<br>Measures that minimize drug contact with the body should always be considered during handling, preparation, and disposal procedures.<br>The total storage time of reconstituted solution containing RDV or placebo should not exceed 4 hours at room temperature (20 °C to 25 °C) or 24 hours at refrigerated temperature (2 °C to 8 °C). Any unused reconstituted solution containing RDV or placebo should be discarded.
**Revised Text:**	Remdesivir for injection and PTM, 100 mg, vials should be stored below 30 °C (86 °F) prior to use. Storage conditions are specified on the label. Until dispensed for dosing, all vials of study drugs should be stored in a securely locked area, accessible only to authorized site personnel.<br>To ensure the sterility, stability, and proper identification, study drug(s) should not be stored in a container other than the container in which they were supplied.<br>Measures that minimize drug contact with the body should always be considered during handling, preparation, and disposal procedures.<br>The total storage time of reconstituted solution containing RDV or placebo should not exceed ***2***4 hours at room temperature (20 °C to 25 °C) or ***48*** ~~24~~ hours at refrigerated temperature (2 °C to 8 °C). Any unused reconstituted solution containing RDV or placebo should be discarded.
**Rationale:**	To include recent update to the study drugs' storage and handling conditions.

**Section:**	Section 8.3.1.1 Efficacy
**Original Text:**	The primary analysis set for efficacy analysis is defined as the Full Analysis Set, which will include all participants who (1) are randomized into the study, (2) have received at least 1 dose of study treatment, and (3) enrolled under protocol amendment 2. Participants will be grouped according to the treatment to which they were randomized.
**Revised Text:**	The primary analysis set for efficacy analysis is defined as the Full Analysis Set, which will include all participants who (1) are randomized into the study~~,~~ ***and*** (2) have received at least 1 dose of study treatment.~~, and (3) enrolled under protocol amendment 2.~~ Participants will be grouped according to the treatment to which they were randomized.
**Rationale:**	Third criterion no longer necessary per updated endpoints.

**Section:**	Section 8.9 Sample Size
**Original Text:**	The total sample size of the study will be approximately 1264 participants, including 60 participants currently enrolled in the study and the number of participants needed for the new primary endpoint under protocol amendment 2 (n 1204).<br>The sample size computation is based on proportions of MAV or death by Day 28 for the RDV and placebo treatment groups. The sample size needed for a 1:1 randomization using a 2-tailed test at level α is given by:<br>$$\frac{(z_{1-\alpha/2}+z_{1-\beta})^2}{P(1-R^2)\sigma^2 B^2}$$<br>Where P is the overall rate of MAV or death, B is the log hazard ratio, σ is the standard deviation of treatment indicator variable, R is the R-Squared that is obtained when treatment indicator variable is regressed on the other covariates, and $z_{1\ \alpha/2}$ and $z_{1\ \beta}$ are the 1-α/2 and 1-β quantiles of the standard normal distribution.<br>For the new primary endpoint of MAV or death under protocol amendment 2, a sample size of 1204 participants (602 in each group with 1:1 randomization) achieves > 90% power to detect a ratio of 0.65 (RDV to placebo in proportion of MAV or death, which is equal to a hazard ratio of 0.624) using a two-sided significance level of 0.05 assuming the overall MAV or death rate is 16.5% (20% in the placebo group and 13% in the RDV group) and a 5% drop out rate. .<br>The sample size calculation was done using software PASS (Version 14.0, module of Cox regression for survival).

**Revised Text:**	The total sample size of the study will be approximately 1264 participants~~, including 60 participants currently enrolled in the study and the number of participants needed for the new primary endpoint under protocol amendment 2 (n = 1204)~~.<br><br>The sample size computation is based on proportions of ***COVID-19 related hospitalization*** ~~MAV~~ or ***all-cause*** death by Day 28 for the RDV and placebo treatment groups. The sample size needed for a 1:1 randomization using a 2-tailed test at level α is given by:<br><br>$$\frac{(z_{1-\alpha/2} + z_{1-\beta})^2}{P(1-R^2)\sigma^2 B^2}$$<br><br>Where P is the overall rate of ***COVID-19 related hospitalization*** ~~MAV~~ or ***all-cause*** death, B is the log hazard ratio, σ is the standard deviation of treatment indicator variable, R is the R-Squared that is obtained when treatment indicator variable is regressed on the other covariates, and $z_{1\ \alpha/2}$ and $z_{1\ \beta}$ are the 1-α/2 and 1-β quantiles of the standard normal distribution.<br><br>~~For the new primary endpoint of MAV or death under protocol amendment 2, a~~ ***A*** sample size of ***1264***~~1204~~ participants (***632***~~602~~ in each group with 1:1 randomization) achieves > 90% power to detect a ratio of ***0.55***~~0.65~~ (RDV to placebo in proportion of ***COVID-19 related hospitalization*** ~~MAV~~ or ***all-cause*** death, which is equal to a hazard ratio of ***0.534***~~0.624~~) using a ***2***~~two~~-sided significance level of 0.05 assuming the overall ***hospitalization***~~MAV~~ or death rate is ***9.3***~~16.5~~% (***12***~~20~~% in the placebo group and ***6.6***~~13~~% in the RDV group) and a 5% drop out rate. ***The sample size provides approximately 80% power to detect smaller treatment effect size with a ratio of 0.60 (RDV to placebo), assuming a 2-sided significance level of 0.05 and the overall hospitalization or death rate is 9.6% (12% in the placebo group and 7.2% in the RDV group) and a 5% drop out rate.*** The sample size calculation was done using software PASS (Version 14.0, module of Cox regression for survival).
**Rationale:**	To align with updated endpoints.

"I have read and understand the above and agree to this protocol amendment as written."	
**Principal Investigator**	**Date**

STATISTICAL ANALYSIS PLAN

**Study Title:**	A Phase 3 Randomized, Double-Blind Placebo-Controlled Trial to Evaluate the Efficacy and Safety of Remdesivir (GS-5734™) Treatment of COVID-19 in an Outpatient Setting
**Name of Test Drug:**	Remdesivir
**Study Number:**	GS-US-540-9012
**Protocol Version (Date):**	Amendment 4: 14 January 2021
**Analysis Type:**	Final Analysis
**Analysis Plan Version:**	1.0
**Analysis Plan Date:**	12 August 2021
**Analysis Plan Author(s):**	PPD

CONFIDENTIAL AND PROPRIETARY INFORMATION

TABLE OF CONTENTS

STATISTICAL ANALYSIS PLAN 1
TABLE OF CONTENTS 2
LIST OF IN-TEXT TABLES 4
LIST OF IN-TEXT FIGURES 4
LIST OF ABBREVIATIONS 5
PHARMACOKINETIC ABBREVIATIONS 7
1. INTRODUCTION 8
1.1. Study Objectives 8
1.2. Study Design 9
1.3. Sample Size and Power 12
2. TYPE OF PLANNED ANALYSIS 13
2.1. Interim Analyses 13
2.1.1. DMC Analysis 13
2.2. Final Analysis 13
2.3. Changes from Protocol-Specified Analyses 13
3. GENERAL CONSIDERATIONS FOR DATA ANALYSES 14
3.1. Analysis Sets 14
3.1.1. All Randomized Analysis Set 14
3.1.2. Full Analysis Set 14
3.1.3. Modified Full Analysis Set 14
3.1.4. Safety Analysis Set 15
3.1.5. Pharmacokinetic Analysis Set 15
3.1.6. Pharmacokinetic Substudy Analysis Set 15
3.1.7. Virology Analysis Set 15
3.2. Subject Grouping 15
3.3. Strata and Covariates 15
3.4. Examination of Subject Subgroups 16
3.5. Multiple Comparisons 16
3.6. Missing Data and Outliers 17
3.6.1. Missing Data 17
3.6.2. Outliers 17
3.7. Data Handling Conventions and Transformations 17
3.8. Analysis Visit Windows 19
3.8.1. Definition of Study Day 19
3.8.2. Analysis Visit Windows 19
3.8.3. Selection of Data in the Event of Multiple Records in an Analysis Visit Window 21
4. SUBJECT DISPOSITION 24
4.1. Subject Enrollment and Disposition 24
4.2. Extent of Study Drug Exposure and Adherence 24
4.3. Protocol Deviations 24
5. BASELINE CHARACTERISTICS 26
5.1. Demographics and Baseline Characteristics 26
5.2. Other Baseline Characteristics 26
5.3. Medical History 27

6. EFFICACY ANALYSES 28
6.1. Primary Efficacy Endpoint....28
6.1.1. Definition of the Primary Efficacy Endpoint 28
6.1.2. Statistical Hypothesis for the Primary Efficacy Endpoint....28
6.1.3. Primary Analysis of the Primary Efficacy Endpoint....28
6.1.4. Secondary Analyses of the Primary Efficacy Endpoint 28
6.2. Secondary Endpoints....29
6.2.1. Analysis of Secondary Endpoints....31
6.3. Exploratory Endpoints 31
6.3.1. Analysis of Exploratory Endpoints 33
6.4. Change from Protocol Specified Efficacy Analyses 33
7. SAFETY ANALYSES....34
7.1. Adverse Events and Deaths....34
7.1.1. Adverse Event Dictionary 34
7.1.2. Adverse Event Severity....34
7.1.3. Relationship of Adverse Events to Study Drug....34
7.1.4. Relationship of Adverse Events to Study Procedure....34
7.1.5. Serious Adverse Events....35
7.1.6. Treatment-Emergent Adverse Events....35
7.1.6.1. Definition of Treatment-Emergent Adverse Events 35
7.1.6.2. Incomplete Dates 35
7.1.7. Summaries of Adverse Events and Deaths....36
7.2. Laboratory Evaluations 37
7.2.1. Summaries of Numeric Laboratory Results 37
7.2.2. Graded Laboratory Values 38
7.2.2.1. Treatment-Emergent Laboratory Abnormalities....38
7.2.2.2. Summaries of Laboratory Abnormalities....38
7.3. Body Weight, Vital Signs and Respiratory Status....39
7.4. Prior and Concomitant Medications....39
7.5. Other Safety Measures 40
7.6. Changes from Protocol-Specified Safety Analyses....40
8. PHARMACOKINETIC (PK) ANALYSES....41
8.1. PK Sample Collection 41
8.2. PK Analyses Related to Intensive PK Sampling....41
8.2.1. Estimation of PK Parameters 41
8.2.2. PK Parameters....42
8.3. PK Analyses Related to Sparse PK Sampling....43
9. REFERENCES 44
10. SOFTWARE....45
11. SAP REVISION....46
12. APPENDICES 47

LIST OF IN-TEXT TABLES

Table 3-1. Analysis Visit Windows for Vital signs 20
Table 3-2. Analysis Visit Windows for Weight, SpO_2 and Respiratory status 20
Table 3-3. Analysis Visit Windows for PCR 20
Table 3-4. Analysis Visit Windows for Hematology, Coagulation and Chemistry Laboratory Tests 21
Table 8-1. Pharmacokinetic Parameters for Each Analyte 42

LIST OF IN-TEXT FIGURES

Figure 1-1. Study Schema 11

LIST OF ABBREVIATIONS

AE	adverse event
ALT	alanine aminotransferase
AST	aspartate aminotransferase
BLQ	below the limit of quantitation
BMI	body mass index
CI	confidence interval
CMH	Cochran-Mantel-Haenszel
CoV	coronavirus
COVID-19	coronavirus disease 2019
CRF	case report form
CSR	clinical study report
DAIDS	Division of AIDS
DAVG	Time-weighted average change from baseline
DMC	data monitoring committee
ED	Emergency department
ET	early termination
FAS	Full Analysis Set
FLU-PRO©	InFLUenza Patient-Reported Outcome
Hb	Hemoglobin
HLT	high-level term
HLGT	high-level group term
ICH	International Conference on Harmonization (of Technical Requirements for Registration of Pharmaceuticals for Human Use)
IV	Intravenous
IXRS	interactive voice or web response system
LLT	lower-level term
LOD	Limit of detection
LOQ	limit of quantitation
MedDRA	Medical Dictionary for Regulatory Activities
PCR	polymerase chain reaction
PK	pharmacokinetics
PT	preferred term
PTM	Placebo to match
Q1, Q3	first quartile, third quartile
RDV	remdesivir (GS-5734™)
SAE	serious adverse event
SAP	statistical analysis plan
SARS	severe acute respiratory syndrome
SD	standard deviation

SI (units)	international system of units
SOC	system organ class
SpO_2	oxygen saturation
TEAE	treatment-emergent adverse event
TFLs	tables, figures, and listings
ULN	upper limit of normal
WHO	World Health Organization

PHARMACOKINETIC ABBREVIATIONS

AUC_{0-24}	area under the concentration versus time curve from time zero to 24 hours
AUC_{last}	area under the concentration versus time curve from time zero to the last quantifiable concentration
C_{last}	last observed quantifiable concentration of the drug
C_{max}	maximum observed concentration of drug
$t_{1/2}$	estimate of the terminal elimination half-life of the drug, calculated by dividing the natural log of 2 by the terminal elimination rate constant (λ_z)
T_{last}	time (observed time point) of C_{last}
T_{max}	time (observed time point) of C_{max}
λz	terminal elimination rate constant, estimated by linear regression of the terminal elimination phase of the concentration of drug versus time curve

1. INTRODUCTION

This statistical analysis plan (SAP) describes the statistical analysis methods and data presentations to be used in tables, figures, and listings (TFLs) in the clinical study report (CSR) for Study GS-US-540-9012. This SAP is based on the study protocol amendment 4 dated 14 January 2021 and the electronic case report form (eCRF). The SAP will be finalized before database finalization. Any changes made after the finalization of the SAP will be documented in the CSR.

1.1. Study Objectives

The primary objectives of this study are as follows:

- To evaluate the efficacy of RDV in reducing the rate of COVID-19 related hospitalization or all-cause death in non-hospitalized participants with early stage COVID-19
- To evaluate the safety of RDV administered in an outpatient setting

The secondary objectives of this study are as follows:

- To evaluate the efficacy of RDV in reducing the rate of COVID-19 related medically attended visits (MAVs; medical visits attended in person by the participant and a health care professional) or all-cause death in non-hospitalized participants with early stage COVID-19
- To determine the antiviral activity of RDV on SARS-CoV-2 viral load
- To assess the impact of RDV on symptom duration and severity

The exploratory objectives of this study include:

- To assess the impact of RDV on other clinical outcomes
- To evaluate the emergence of viral resistance to RDV
- To identify and assess associations of host biomarkers with disease progression and treatment response
- To assess the PK of RDV and its metabolites in participants with COVID-19
- To assess patient-reported outcome using the COVID-19 adapted InFLUenza Patient-Reported Outcome (FLU-PRO Plus©) questionnaire and validate the questionnaire (if available)

1.2. Study Design

This is a Phase 3, randomized, double-blind, placebo-controlled, multicenter study of RDV therapy for outpatients with early stage COVID-19 who are at higher risk of disease progression.

Participants who meet all eligibility criteria may be randomized in a 1:1 ratio to RDV or placebo. Randomization will be stratified by participants who reside in a skilled nursing facility, by participant's age (< 60 vs ≥60 years), and by region (United States [US] vs. ex-US):

Treatment Group A: single dose of IV RDV 200 mg on Day 1 followed by IV RDV 100 mg on Days 2 and 3 (RDV group)

Treatment Group B: IV placebo-to-match (PTM) RDV on Days 1 to 3 (PTM group)

Participants must meet all of the following inclusion criteria to be eligible for participation in this study:

1) Willing and able to provide written informed consent (participants ≥ 18 years of age) or assent (participants ≥ 12 and < 18 years of age) prior to performing study procedures. Participants aged ≥ 18 years may be enrolled with the consent of a legal representative where permitted according to local law and approved nationally and by the relevant institutional review board (IRB) or independent ethics committee (IEC). For participants ≥ 12 and < 18 years of age, a parent or legal guardian must be willing and able to provide written informed consent prior to performing study procedures

2) Either:

    — Age ≥ 18 years (at all sites) or aged ≥ 12 and < 18 years of age weighing ≥ 40 kg (where permitted according to local law and approved nationally and by the relevant IRB or IEC) with at least 1 of the following pre-existing risk factors for progression to hospitalization:

    a) Chronic lung disease: chronic obstructive pulmonary disease, moderate-to-severe asthma, cystic fibrosis, pulmonary fibrosis

    b) Hypertension: systemic or pulmonary

    c) Cardiovascular or cerebrovascular disease: coronary artery disease, congenital heart disease, heart failure, cardiomyopathy, history of stroke, atrial fibrillation, hyperlipidemia

    d) Diabetes mellitus: Type 1, type 2, or gestational

    e) Obesity (BMI ≥ 30)

f) Immunocompromised state; having a solid organ transplant, blood, or bone marrow transplant; immune deficiencies; HIV with a low CD4 cell count or not on HIV treatment; prolonged use of corticosteroids; or use of other immune weakening medicines

g) Chronic mild or moderate kidney disease

h) Chronic liver disease

i) Current cancer

j) Sickle cell disease

— OR age ≥ 60 years, regardless of the presence of other pre-existing risk factors for progression

3) SARS-CoV-2 infection confirmed by molecular diagnostics (nucleic acid [eg, PCR] or antigen testing) ≤ 4 days prior to screening

4) Presence of ≥ 1 symptom(s) consistent with COVID-19 for ≤ 7 days prior to randomization (such as fever, cough, fatigue, shortness of breath, sore throat, headache, myalgia/arthralgia)

5) Not currently receiving, requiring, or expected to require supplemental oxygen

6) Not currently requiring hospitalization (hospitalization defined as ≥ 24 hours of acute care)

7) Participants of childbearing potential who engage in heterosexual intercourse must agree to use protocol-specified method(s) of contraception

Exclusion criteria for participation include:

1) Participation in any other clinical trial of an experimental treatment and prevention for COVID-19

2) Prior hospitalization for COVID-19 (hospitalization defined as ≥ 24 hours of acute care)

3) Treatment with other agents with actual or possible direct antiviral activity against SARS-CoV-2 or administration of any SARS-CoV-2 (or COVID-19) vaccine

4) Criterion removed

5) Requiring oxygen supplementation

6) ALT or AST ≥ 5 × upper limit of normal (ULN) at screening or within 90 days of screening
Note: if per local practice only ALT is routinely measured, exclusion criteria will be evaluated on ALT alone

7) Creatinine clearance < 30 mL/min at screening or within 90 days of screening using the Cockcroft-Gault formula in participants ≥ 18 years of age or estimated glomerular filtration rate (eGFR) < 30 mL/min/1.73m^2 at screening or within 90 days of screening using the Schwartz formula in participants < 18 years of age (see Section 6.7.2 of protocol)

8) Currently breastfeeding (nursing)

9) Known hypersensitivity to the study drug, metabolites, or formulation excipient

10) Use or planned use of exclusionary medications (refer to Section 5.4 of protocol)

Participants will receive study treatment with RDV for 3 days (Treatment Group A) or PTM for 3 days (Treatment Group B).

Figure 1-1. Study Schema

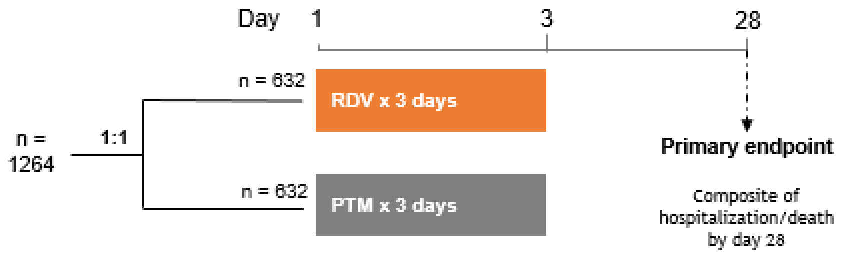

On study Days 1 through 3, vital signs including respiratory status and SpO_2 will be measured, and adverse events (AEs) and concomitant medications will be documented. Laboratory tests for safety (hematology, coagulation and chemistry) will be performed on Days 1, 3, 7, and 14. Nasopharyngeal swabs and sputum samples will be collected on Days 1, 2, 3, 7, and 14 for SARS-CoV-2 quantitative reverse transcriptase polymerase chain reaction viral load testing and possible resistance testing.

On study Day 14 physical examination findings, vital signs including temperature, respiratory rate, and SpO_2, AEs and concomitant medications will be documented.

Symptom severity will be assessed daily from Day 1 through Day 14 using the COVID-19 adapted FLU-PRO Plus questionnaire (if available). On study Day 28, there will be an in-person or phone visit. Physical examination, vital signs including temperature, respiratory rate, and SpO_2, AEs, MAV information and concomitant medications will be documented (only AEs, MAV information and concomitant medications needed if performed by phone).

Sparse PK assessments will be conducted in all participants at participating sites and approximately 30 participants will have intensive PK assessments.

1.3. Sample Size and Power

The total sample size of the study will be approximately 1264 participants.

The sample size computation is based on proportions of COVID-19 related hospitalization or all-cause death by Day 28 for the RDV and placebo treatment groups. The sample size needed for a 1:1 randomization using a 2-tailed test at level α is given by:

$$\frac{(z_{1-\alpha/2} + z_{1-\beta})^2}{P(1 - R^2)\sigma^2 B^2}$$

Where P is the overall rate of COVID-19 related hospitalization or all-cause death, B is the log hazard ratio, σ is the standard deviation of treatment indicator variable, R is the R-Squared that is obtained when treatment indicator variable is regressed on the other covariates, and $z_{1-\alpha/2}$ and $z_{1-\beta}$ are the 1-α/2 and 1-β quantiles of the standard normal distribution.

A sample size of 1264 participants (632 in each group with 1:1 randomization) achieves > 90% power to detect a ratio of 0.55 (RDV to placebo) in proportion of COVID-19 related hospitalization or all-cause death, which is equal to a hazard ratio of 0.534) using a two-sided significance level of 0.05 assuming the overall COVID-19 related hospitalization or all-cause death rate is 9.3% (12% in the placebo group and 6.6% in the RDV group) and a 5% drop out rate. The sample size provides approximately 80% power to detect a smaller treatment effect size with a ratio of 0.60 (RDV to placebo), assuming a 2-sided significance level of 0.05 and the overall COVID-19 related hospitalization or all-cause death rate is 9.6% (12% in the placebo group and 7.2% in the RDV group) and a 5% drop out rate. The proportion of patients with COVID-19-related hospitalizations or emergency department (ED) visits was 13.5% in high risk patients (age ≥ 65 or BMI ≥ 35) who received placebo{Gottlieb 2021}, 12% is assumed for the study to account for decrease in hospitalization rate in recent months.

The sample size calculation was done using software PASS (Version 14.0, module of Cox regression for survival).

2. TYPE OF PLANNED ANALYSIS

2.1. Interim Analyses

Prior to the final analysis, interim analyses may be conducted, and the analyses may be submitted to regulatory agencies to seek guidance for the overall clinical development program.

2.1.1. DMC Analysis

One external multidisciplinary DMC was planned to review the progress of the study and to perform interim reviews of the efficacy (futility assessment) and safety data. However, this DMC analysis was not performed due to the stop of study enrollment after 584 participants randomized and prior to reaching the planned DMC analysis schedule (ie, approximately 50% of the total 1264 planned participants have completed Day 28 assessment).

2.2. Final Analysis

The final analysis will be performed after all participants have completed or discontinued from the study, outstanding data queries have been resolved or adjudicated as unresolvable, and the data have been cleaned and finalized. This SAP describes the analysis plan for the final analysis.

2.3. Changes from Protocol-Specified Analyses

The DMC analysis was not performed due to the stop of enrollment on 08 April 2021 after less than 50% of the participants were randomized. The reasons of stopping enrollment were due to significant changes in the epidemiology of COVID-19 from a combination of lower hospitalization rates and high vaccine rates in high-risk patients, and a change in the unmet patient need for convenient at home options for non-hospitalized patients with COIVD-19. The decsion to stop study ernollment was documented on 06-APR-2021 Study Management Team meeting minutes.

3. GENERAL CONSIDERATIONS FOR DATA ANALYSES

Analysis results will be presented using descriptive statistics. For categorical variables, the number and percentage of participants in each category will be presented; for continuous variables, the number of participants (n), mean, standard deviation (SD) or standard error (SE), median, first quartile (Q1), third quartile (Q3), minimum, and maximum will be presented.

All statistical tests will be 2-sided and performed at the 5% significance level unless otherwise specified.

By-subject listings will be presented for all participants in the All Randomized Analysis Set and sorted by subject ID number, visit date, and time (if applicable). Data collected on log forms, such as AEs, will be presented in chronological order within the participant. The treatment group to which participants were randomized will be used in the listings. Age, sex at birth, race, and ethnicity will be included in the listings, as space permits.

3.1. Analysis Sets

Analysis sets define the participants to be included in an analysis. Analysis sets and their definitions are provided in this section. Participants included in each analysis set will be determined before the study blind is broken for analysis. The analysis set will be identified and included as a subtitle of each table, figure, and listing.

For each analysis set, the number and percentage of participants eligible for inclusion, will be summarized by treatment group.

A listing of reasons for exclusion from analysis sets will be provided by participant.

3.1.1. All Randomized Analysis Set

All Randomized Analysis Set includes all participants who were randomized in the study.

3.1.2. Full Analysis Set

The primary analysis set for efficacy analysis is defined as the Full Analysis Set (FAS), which will include all participants who (1) are randomized into the study, and (2) have received at least 1 dose of study treatment. Participants will be grouped according to the treatment to which they were randomized.

3.1.3. Modified Full Analysis Set

A modified full analysis set (mFAS) includes all participants who (1) are randomized into the study, and (2) have received at least 1 dose of study treatment, and (3) enrolled under protocol amendment 2 or later. Participants will be grouped according to the treatment to which they were randomized.

3.1.4. Safety Analysis Set

The primary analysis set for safety analyses is defined as the Safety Analysis Set, which will include all participants who (1) are randomized into the study and (2) have received at least 1 dose of study treatment. Participants will be grouped according to the treatment which they received.

3.1.5. Pharmacokinetic Analysis Set

The PK Analysis Set will include all randomized participants who received at least 1 dose of RDV and had at least 1 non-missing PK concentration datum reported by the PK laboratory for each respective analyte (RDV, GS-704277, GS-441524).

3.1.6. Pharmacokinetic Substudy Analysis Set

The Pharmacokinetic (PK) Substudy Analysis Set will include all randomized participants who took at least 1 dose of study drug, participated in the intensive PK collection, and have at least 1 nonmissing postdose concentration for each respective analyte (RDV, GS-704277, GS-441524). This is the primary analysis set for detailed PK analysis of intensive PK sampling.

3.1.7. Virology Analysis Set

The Virology Analysis Set will include all participants who (1) are randomized into the study, (2) have received at least 1 dose of study treatment, and (3) have positive SARS-CoV-2 viral load at baseline (result of 'No SARS-CoV-2 detected' is considered as negative, results of 'Inconclusive', "<2228cp/mL SARSCoV2 detected" and numerical results are considered as positive).

3.2. Subject Grouping

For analyses based on the All Randomized Analysis Set, FAS, mFAS or Virology Analysis Set, participants will be grouped according to the treatment to which they were randomized. For the safety analysis set, the actual treatment received will differ from the randomized treatment only when their actual treatment differs from randomized treatment for the entire treatment duration.

For the PK Analysis Set and the PK Substudy Analysis Set, participants will be grouped according to the actual treatment they received.

3.3. Strata and Covariates

Participants will be randomly assigned to treatment groups via the interactive voice or web response system (IXRS) in a 1:1 ratio using a stratified randomization schedule. Stratification will be based on the following variables:

- Region: US vs. ex-US
- Participant's age: < 60 vs ≥ 60 years
- Resident of skilled nursing facility: Yes vs. No

Stratification discrepancies will be reviewed and assessed. The values recorded in the clinical database will be used for analyses in case there are discrepancies between the IXRS and the clinical database. Based on the assessment of stratification discrepancies, a sensitivity analysis of the primary endpoint may be performed.

The primary efficacy endpoint will be evaluated in a stratified analysis based on the same 3 factors used for randomization, as specified in Section 6.1.3. In addition, observed imbalances between treatment groups in other baseline characteristics may be considered as covariates in sensitivity analyses of efficacy endpoints.

3.4. Examination of Subject Subgroups

The primary endpoint will be examined for the following participant subgroups:

- region (US vs. ex-US)
- participant's age (<18, 18≤ - < 60, ≥ 60 years),
- participants who reside in a skilled nursing facility (Yes, No),
- Sex at birth: (a) male and (b) female
- Race: (a) Asian, (b) Black, (c)White, (d) other
- Baseline risk factor: Chronic lung disease (Yes, No), Hypertension (Yes, No), Cardiovascular or cerebrovascular disease (Yes, No), Diabetes mellitus (Yes, No), Obesity (Yes, No), Immunocompromised state (Yes, No), Chronic mild or moderate kidney disease (Yes, No), Chronic liver disease (Yes, No), Current cancer (Yes, No), Sickle cell disease (Yes, No)
- Common COVID-19 symptoms at baseline: absence or presence for each of the following symptoms: Stuffy or runny nose; Sore throat; Shortness of breath (difficulty breathing); Cough; Low energy or tiredness; Muscle or body aches; Headache; Chills or shivering; Feeling hot or feverish; Nausea; Vomit; Diarrhea; Loss of Smell; Loss of taste.

In addition, other subgroups may be considered based on imbalances between treatment groups observed in other baseline characteristics.

3.5. Multiple Comparisons

There will be no multiplicity adjustment in the final analysis. Efficacy will be evaluated using the primary efficacy endpoint at the significance level of 0.05. All other efficacy endpoints are exploratory in nature and will be tested using 2-sided tests at the 5% significance level without multiplicity adjustment.

3.6. Missing Data and Outliers

3.6.1. Missing Data

In general, missing data will not be imputed unless methods for handling missing data are specified. Exceptions are presented in this document.

Participants with missing outcomes for the primary endpoint due to premature discontinuation of the study will be censored at last study date.

In this study, a missing pre-treatment laboratory result would be treated as normal (ie, no toxicity grade) for the laboratory abnormality summary. The handling of missing or incomplete dates for AE onset is described in Section 7.1.6.2, and for prior and concomitant medications in Section 7.4.

3.6.2. Outliers

Outliers will be identified during the data management and data analysis process. No sensitivity analyses to evaluate the impact of outliers on efficacy or safety outcomes are planned. Unless specified otherwise, all data will be included in the analyses.

3.7. Data Handling Conventions and Transformations

In general, age collected at Day 1 (in years) will be used for analyses and presented in listings. If age at Day 1 is not available for a participant, then age derived based on date of birth and the first dose date will be used instead. If an enrolled participant was not dosed with any study drug, the randomization date will be used instead of the Day 1 visit date. For screen failures, the date the first informed consent was signed will be used for the age derivation. Age required for longitudinal and temporal calculations and analyses (e.g., estimates of creatinine clearance, age at date of AE) will be based on age derived from date of birth and the date of the measurement or event, unless otherwise specified.

The following conventions will be used for the imputation of date of birth when it is partially missing or not collected:

- If only year of birth is collected, then “01 July” will be imputed as the day and month of birth
- If year of birth is missing, then date of birth will not be imputed.

Laboratory data that are continuous in nature but are less than the lower limit of quantitation (LOQ) or above the upper LOQ will be imputed as follows:

- A value that is 1 unit less than the LOQ will be used to calculate descriptive statistics if the datum is reported in the form of "< x" (where x is considered the LOQ). For example, if the values are reported as < 50 and < 5.0, values of 49 and 4.9, respectively, will be used to calculate summary statistics. An exception to this rule is any value reported as < 1 or < 0.1, etc. For values reported as < 1 or < 0.1, a value of 0.9 or 0.09, respectively, will be used to calculate summary statistics.

- A value that is 1 unit above the LOQ will be used to calculate descriptive statistics if the datum is reported in the form of "> x" (where x is considered the LOQ). Values with decimal points will follow the same logic as above.

- The LOQ will be used to calculate descriptive statistics if the datum is reported in the form of "≤ x" or "≥ x" (where x is considered the LOQ).

SARS-COV2 viral load results that are below LOQ but have a positive signal will be reported as "<2228cp/mL SARSCoV2 detected" and those that are below lower limit of detection(LOD) and negative will be reported as "No SARS-CoV2 detected". The data will be imputed as follows:

- A value of 1114 copies/ml (half of the LOQ 2228 copies/ml) will be used to calculated descriptive statistics if the datum is reported as "<2228cp/mL SARSCoV2 detected".

- A value of 746.5 copies/ml (1/2 of the LOD 1493 copies/ml) will be used to calculated descriptive statistics if the datum is reported as "No SARS-CoV2 detected"

Any SARS-CoV-2 viral load samples collected on or after the participants are receiving additional COVID-19 treatments (see Appendix 2) will be excluded from the viral load analysis.

Base 10 logarithm transformation will be used for analyzing SARS-CoV-2 viral load.

Sparse PK concentration values that are below the limit of quantitation (BLQ) will be presented as "BLQ" in the data listing.

Natural logarithm transformation will be used for analyzing concentrations and PK parameters in intensive PK samples. Concentration values that are BLQ will be presented as "BLQ" in the concentration data listing. For intensive PK concentration, values that are BLQ will be treated as 0 at predose time points, and one-half the value of the LOQ at postdose time points for summary purpose. For predose or postdose timepoint of sparse PK and single anytime PK, values that are BLQ will be treated as one-half the value of the LLOQ for summary purpose.

The following conventions will be used for the presentation of summary and order statistics for intensive PK concentrations:

- If at least 1 participant has a concentration value of BLQ for the time point, the minimum value will be displayed as "BLQ."

- If more than 25% of the participants have a concentration data value of BLQ for a given time point, the minimum and Q1 values will be displayed as "BLQ."
- If more than 50% of the participants have a concentration data value of BLQ for a given time point, the minimum, Q1, and median values will be displayed as "BLQ."
- If more than 75% of the participants have a concentration data value of BLQ for a given time point, the minimum, Q1, median, and Q3 values will be displayed as "BLQ."
- If all participants have concentration data values of BLQ for a given time point, all order statistics (minimum, Q1, median, Q3, and maximum) will be displayed as "BLQ."

PK parameters that are BLQ will be imputed as one-half LOQ before log transformation or statistical model fitting.

3.8. Analysis Visit Windows

3.8.1. Definition of Study Day

Study day will be calculated from the first dosing date of study drug and derived as follows:

- For postdose study days: Assessment Date – First Dosing Date + 1
- For days prior to the first dose: Assessment Date – First Dosing Date

Therefore, study day 1 is the day of first dose of study drug administration.

Last Dose Date is defined as the maximum, nonmissing, nonzero dose end date of treatment recorded on the Study Drug Administration eCRF form with "Study Drug Permanently Withdrawn" box checked for participants who prematurely discontinued or completed study drug according to the Study Drug Completion eCRF. Refer to Appendix 2 for missing date imputation, if necessary.

Last Study Date is the latest of the study drug start dates and end dates, the in-person or phone visit dates, the vital sign or respiratory status collection dates, Flu-PRO Plus questionnaire collection date and the laboratory collection dates, including the 28-day follow-up visit date, and the death date (if applicable, only for participants who prematurely discontinued study according to the Study Completion eCRF).

Baseline value is defined as the last value obtained on or prior to the first dose date (and time, if available) unless otherwise specified (see Section 3.8.3).

3.8.2. Analysis Visit Windows

Participant visits might not occur on protocol-specified days. Therefore, for the purpose of analysis, observations will be assigned to analysis windows.

The analysis windows for Vital signs are provided in Table 3-1.

Table 3-1. Analysis Visit Windows for Vital signs

Nominal Visit	Nominal Study Day	Visit Window Study Day	
		Lower Limit	Upper Limit
Baseline	1	(none)	1 (Pre Dose)*
Day 1	1	1 (Post Dose) *	1 (Post Dose)
Day 2#	2	2	2
Day 3#	3	3	4
Day 7	7	5	9
Day 14	14	10	21
Day 28	28	22	(none)

* For baseline, the upper limit includes values collected at or prior to the first dose date/time. For Day 1, the lower limit includes values collected after the first dose date/time on Day 1.

\# The pre dose and post dose timepoints for Days 2 and 3 will be based on nominal time point. The analysis windows for Weight, and Respiratory status are provided in Table 3-2.

Table 3-2. Analysis Visit Windows for Weight, SpO_2 and Respiratory status

Nominal Visit	Nominal Study Day	Visit Window Study Day	
		Lower Limit	Upper Limit
Baseline	1	(none)	1 (Pre Dose)*
Day 2	2	1 (Post Dose) *	2
Day 3	3	3	4
Day 7	7	5	9
Day 14	14	10	21
Day 28	28	22	(none)

* For baseline, the upper limit includes values collected at or prior to the first dose date/time. For Day 2, the lower limit includes values collected after the first dose date/time on Day 1.

The analysis windows for PCR are provided in Table 3-3.

Table 3-3. Analysis Visit Windows for PCR

Nominal Visit	Nominal Study Day	Visit Window Study Day	
		Lower Limit	Upper Limit
Baseline	1	(none)	1 (Pre Dose)
Day 2	2	1 (Post Dose)	2
Day 3	3	3	4
Day 7	7	5	9
Day 14	14	10	21
Post Day 14	22	22	none

The analysis windows for Chemistry and Hematology laboratory parameters are provided in Table 3-4.

Table 3-4. Analysis Visit Windows for Hematology, Coagulation and Chemistry Laboratory Tests

Nominal Visit	Nominal Study Day	Visit Window Study Day	
		Lower Limit	Upper Limit
Baseline	1	(none)	1 (Pre Dose)
Day 3	3	1 (Post Dose)	4
Day 7	7	5	9
Day 14	14	10	(none)

FLU-PRO Plus Questionnaire were to be collected daily from Day 1 through Day 14; therefore, windows are not assigned and results will be summarized for each Study Day.

3.8.3. Selection of Data in the Event of Multiple Records in an Analysis Visit Window

Depending on the statistical analysis method, single values may be required for each analysis window. For example, change from baseline by visit usually requires a single value, whereas a time-to-event analysis would use all data regardless of analysis window.

If multiple valid, nonmissing measurements exist in an analysis window, records will be chosen based on the following rules if a single value is needed:

- For baseline, the last nonmissing value on or prior to the first dosing date (and time, if available) of study drug will be selected, unless specified differently. If there are multiple records with the same time or no time recorded on the same day, the baseline value will be the average of the measurements for continuous data, or the measurement with the lowest severity for categorical data.
  - For baseline, if both local lab and central lab results are available, central lab results will be selected first for baseline, local lab results will be used when central lab results are missing.
  - For FLU-PRO Plus data, if there are multiple records on Day 1, the baseline value will be selected as follows:
    - The record closest to the dosing day will be selected.
    - If there are more than 1 records on the selected day with different times, the least severe score will be selected as baseline. If there are multiple records with same severity, the measurement with the lowest severity at later time will be selected.

- For postbaseline values (other than PCR negative confirmation and PCR for time-weighted average change from baseline (DAVG)):
  - The record closest to the nominal day for that visit will be selected.
  - If there are 2 records that are equidistant from the nominal day, the later record will be selected.
  - If there is more than 1 record on the selected day, values will be selected for analysis as follows:
    - For PCR, if there is more than 1 record on the selected day, the latest value will be selected. If there are multiple records with the same time or no time recorded on the same day, the geometric mean value (copies/mL) will be taken. For laboratory values (other than PCR) and SpO_2, if there is more than 1 record on the selected day, the average value will be selected.
    - For FLU-PRO Plus data, if there are more than 1 record on the same day, the worst value will be selected.
    - For RBC Morphology result, if there is more than 1 record on the selected day, all results will be selected.
    - For vital signs, the pre dose and post dose timepoints for Days 2 and 3 will be summarized by nominal time point. If there is more than 1 record on a time point, the average will be taken.
    - For other parameters, if there is more than 1 record on the selected day, the average will be taken for continuous data and the worst severity will be taken for categorical data, unless otherwise specified.
- For postbaseline values of PCR negative confirmation:
  - All available data will be used to derive negative confirmation except if there are multiple records on the same day, the worst value will be used for negative confirmation for that day
  - The record closest to the nominal day for that visit will be selected.
  - If there are 2 records that are equidistant from the nominal day, the later record will be selected.
- For postbaseline values PCR for DAVG calculation:
  - All values from different days within an analysis windows will be used for DAVG calculation.

— If there is more than 1 record on a study day, the latest value will be selected. If there are multiple records with the same time or no time recorded on the same day, the geometric mean value (copies/mL) will be taken.

4. SUBJECT DISPOSITION

4.1. Subject Enrollment and Disposition

The number and percentage of participants randomized at each investigator site will be summarized by treatment group and overall using the Safety Analysis Set and All Randomized Analysis Set. The number and percentage of participants enrolled by randomized stratum will be summarized using stratum assignment captured in the IXRS. Discrepancy between IXRS and clinical database will be noted if applicable. The denominator for this calculation will be the number of participants in the Safety Analysis Set.

The summary of subject disposition will be provided by treatment group and overall for all screened participants. This summary will include the number of participants screened, screen failure participants who were not randomized, participants who met all eligibility criteria and were not randomized, participants randomized, participants randomized but never treated, participants in the Safety Analysis Set, participants in the modified FAS and participants in the FAS.

In addition, the number and percentage of the participants in the following categories will be summarized:

- Completed randomized treatment on study drug as recorded on the Study Drug Completion form
- Prematurely discontinuing study drug prior to completion with summary of reasons for discontinuing study drug as recorded on the Study Drug Completion form
- Completed study
- Prematurely discontinuing from study (with summary of reasons for discontinuing study) as recorded on the Study Completion form

The denominator for the percentages of participants in each category will be the number of participants in the Safety Analysis Set.

No inferential statistics will be generated. A data listing of reasons for premature study drug/study discontinuation will be provided.

4.2. Extent of Study Drug Exposure and Adherence

Number of doses received will be summarized by treatment group for the Safety Analysis Set.

4.3. Protocol Deviations

A listing will be provided for all randomized participants who violated at least 1 inclusion or exclusion criterion. The listing will include the criteria not met.

Protocol deviations occurring after participants entered the study are documented during routine monitoring. The number and percentage of participants with important protocol deviations and the total number of important protocol deviations by deviation reason (e.g., nonadherence to study drug, violation of select inclusion/exclusion criteria) will be summarized by treatment group for the FAS. A by-subject listing will be provided for those participants with important protocol deviation.

5. BASELINE CHARACTERISTICS

5.1. Demographics and Baseline Characteristics

Participant demographic data (eg, sex, race/ethnicity, age, and age group (<18, ≥18 - < 60, ≥ 60) and baseline characteristics (eg, body weight, height, and body mass index [BMI]) will be summarized by treatment group and overall using descriptive statistics (n, mean, SD, median, Q1, Q3, minimum, and maximum) for continuous data and by the number and percentage of participants for categorical data. The summaries of demographic data and baseline participant characteristics will be provided for the Safety Analysis Set.

For categorical data, the Cochran-Mantel-Haenszel (CMH) test (ie, general association statistic for nominal data and row mean scores for ordinal data) will be used to compare the 2 treatment groups. For continuous data, the 2-sided Wilcoxon rank sum test will be used to compare the 2 treatment groups.

Similar summaries will be produced for the following subgroups: region (US vs. ex-US), participant's age (< 60 vs ≥ 60 years), and participants who reside in a skilled nursing facility (Yes, No).

A by-subject demographic listing will be provided.

5.2. Other Baseline Characteristics

The following baseline disease characteristics will be summarized by treatment group and overall using descriptive statistics:

- Baseline risk factor as defined in Section 3.4
- Duration of symptoms prior to first dose of study drug
- Duration from SARS-CoV-2 nucleic acid/antigen confirmation to first dose of study drug
- Alanine aminotransferase (ALT)
- Aspartate aminotransferase (AST)
- Baseline SARS-CoV-2 viral Load (as a continuous variable, and a categorical variable with categories of < median and ≥ median)
- Baseline respiration rate

For categorical data, the CMH test (general association statistic for nominal data) will be used to compare the 2 treatment groups. For continuous data, the 2-sided Wilcoxon rank sum test will be used to compare the 2 treatment groups. Similar summaries will be produced for the following

subgroups: region (US vs. ex-US), participant's age (< 60 vs ≥60 years), and participants who reside in a skilled nursing facility (Yes, No).

Categorical baseline COVID-19 symptoms from Flu-PRO Plus will be summarized by treatment group separately from other baseline characteristics.

5.3. Medical History

Any medical condition or clinically significant laboratory abnormality with an onset date before the consent form is signed and not related to a protocol-associated procedure is considered to be preexisting and should be documented as medical history. General medical history data will be collected at screening. It will be coded using the current version of MedDRA. A summary table will present the percentages of participants reporting each medical history preferred term, sorted first in alphabetical order by system organ class (SOC) and then by preferred term (PT) in descending order of total frequency within SOC.

6. EFFICACY ANALYSES

6.1. Primary Efficacy Endpoint

6.1.1. Definition of the Primary Efficacy Endpoint

The primary endpoint of the study is the composite endpoint of COVID-19 related hospitalization (defined as at least 24 hours of acute care) or all-cause death by Day 28. The endpoint will be derived by combining the available all-cause death and COVID-19 related hospitalization reported by the site. The first COVID-19 related hospitalization will be used for the proportion of COVID-19 related hospitalization or all-cause death.

6.1.2. Statistical Hypothesis for the Primary Efficacy Endpoint

Null hypothesis: The hazard ratio of COVID-19 related hospitalization or all-cause death by Day 28 between the 2 treatment groups equals to 1.

Alternative hypothesis: The hazard ratio of COVID-19 related hospitalization or all-cause death by Day 28 between the 2 treatment groups does not equal to 1.

6.1.3. Primary Analysis of the Primary Efficacy Endpoint

The hazard ratio of COVID-19 related hospitalization or all-cause death between the two treatment groups will be estimated using a Cox model with stratification factors as covariates.

If a participant prematurely discontinues from the study prior to Day 28 or the hospitalization status is missing, the participant is censored at last study date or day 28 whichever is earlier. If a participant has a COVID-19 related hospitalization first and then dies, then date of the COVID-19 related hospitalization and status will be used for the primary analysis for this participant. If a participant has a non COVID-19 related hospitalization first and then dies without experiencing a COVID-19 related hospitalization, then date of the death and status will be used for the primary analysis for this participant.

The hazard ratio, p-value, 95% CI for the hazard ratio from Cox model, and proportion of COVID-19 related hospitalization or all-cause death at Day 28 from Kaplan-Meier estimate will be provided.

The FAS will be the primary analysis set for efficacy endpoint evaluation. The primary endpoint may be analyzed for each of the subgroups defined in Section 3.3.

6.1.4. Secondary Analyses of the Primary Efficacy Endpoint

Sensitivity analyses maybe conducted using the following alternative approaches for the primary endpoint.

- A CMH test including baseline stratification factors as strata for the statistical comparison between the two treatment groups. If a participant prematurely discontinues from the study

prior to Day 28 with no event before discontinuation or the hospitalization/death status is missing, the participant will be considered as with no hospitalization/death.

The analysis is for the purposes of evaluating the robustness of the estimates of the primary analysis.

6.2. Secondary Endpoints

The other endpoints of interest include:

- Composite endpoint of COVID-19 related MAVs (medical visits attended in person by the participant and a health care professional) or all cause death by Day 28
- All-cause mortality by Day 28
- Proportion of participants hospitalized (COVID-19 related) by Day 28
- Composite endpoint of COVID-19 related hospitalization (defined as at least 24 hours of acute care) or all-cause death by Day 14
- Composite endpoint of COVID-19 related MAVs (medical visits attended in person by the participant and a health care professional) or death by Day 14
- Time-weighted average change in SARS-CoV-2 viral load from baseline to Day 7
  - — The time-weighted average change from baseline to study Day 7 ($DAVG_7$) in SARS-CoV-2 viral load ($\log_{10}$ copies/mL) is defined as the time-weighted average between the first postbaseline value through the last available value up to Day 7 minus the baseline value in SARS-CoV-2 viral load ($\log_{10}$ copies/mL). $DAVG_7$ is calculated using the trapezoidal rule and the area-under-the-curve (AUC) concept as follows:

$$DAVG_7 = \frac{AUC_{t_1-t_7}}{(t_7 - t_1)} - Y_0$$

The AUC between t_1 and t_n in a time (ie, day) versus SARS-CoV-2 viral load (log10 copies/mL) plot is calculated as

$$AUC_{t_1-t_n} = \sum_{i=1\ to\ (n-1)} \frac{1}{2}(Y_i + Y_{i+1}) \times (t_{i+1} - t_i)$$

Where Y_i is the SARS-CoV-2 viral load (log10 copies/mL) at time t_i, t_1 is the first postbaseline time, and Y_0 is the baseline SARS-CoV-2 viral load (log10 copies/mL).

  - — For subject with SARS-CoV-2 viral load data only available up to Day x ($x < 7$), $DAVG_7$ is defined as

$$DAVG_7 = \frac{AUC_{t_1 - t_x}}{(t_x - t_1)} - Y_0$$

— If baseline viral load is missing or there is no post-baseline data, then the participant will be excluded from the analysis. If there is one post-baseline data value Y_i, $DAVG_7 = Y_i - Y_0$. If a participant receives additional COVID-19 treatments (see Appendix 2), the viral load from the start of additional treatment will be excluded.

- Time to alleviation (mild or absent) of baseline COVID-19 symptoms as reported on the COVID-19-adapted FLU-PRO Plus

— The alleviation of baseline COVID-19 symptoms, i.e. overall alleviation of all symptoms scored 1 or higher at baseline for each participant, is defined as:

  - Symptoms scored as 2 or higher at baseline are scored as 0 or 1 at postbaseline, AND
  - Symptoms scored as 1 at baseline are scored as 0 at postbaseline, AND
  - for two consecutive days.

— Time to alleviation of baseline COVID-19 symptoms is defined (in days) as:

First Date of the two consecutive dates achieving alleviation - First dose Date + 1

If a participant has not achieved symptom alleviation at last FLU-PRO Plus assessment or early discontinuation of study, the participant will be censored at last FLU-PRO Plus assessment date. Last assessment date is the last date when at least one of the baseline symptoms is assessed.

Similar endpoints of time to alleviation of all symptoms or selected symptoms as reported on the COVID-19-adapted FLU-PRO Plus may be included.

- Worsening after alleviation of baseline COVID-19 symptoms

— The worsening after alleviation of baseline COVID-19 symptoms is defined as:

  - For a participant who has achieved alleviation of baseline COVID-19 symptoms
  - If any symptom scored as 2 or higher at baseline is scored as 2 or higher postbaseline after achieved alleviation, or
  - Any symptom scored as 1 at baseline are scored as 1 or higher postbaseline after achieved alleviation.

- Proportion of participants progressing to requiring oxygen supplementation by Day 28

6.2.1. Analysis of Secondary Endpoints

The FAS will be the primary analysis set for secondary endpoints. The mFAS will be used for secondary endpoints of composite endpoint of COVID-19 related MAVs or death.

All-cause mortality by Day 28 will be compared between the 2 treatment groups using the Fisher exact test. If a participant prematurely discontinues from the study prior to Day 28 or the Death status is missing, the participant will not be included in the analysis.

The secondary endpoint of COVID-19 related hospitalization or all-cause death by Day 14, COVID-19 related MAVs or death by Day 28, and COVID-19 related MAVs or death by Day 14 will be analyzed using the same method as used for the primary endpoint, described in Section 6.1.3.

The proportion of participants hospitalized (COVID-19 related) by Day 28 will be estimated using the Kaplan-Meier method and compared between the 2 treatment groups using a log-rank test. Hazard ratio and two-sided 95% CI estimated using the Cox regression with baseline stratification factors as covariates will be provided.

Number and percentage of participants progressing to requiring oxygen supplementation by Day 28 will be summarized and compared between treatment groups using the Fisher Exact test. Participants discontinued from the study before progressing to requiring oxygen supplementation will be considered as not requiring oxygen supplementation.

The Kaplan-Meier product limit method will be used to estimate and log-rank test will be used to compare treatment groups for the time to alleviation of baseline COVID-19 symptoms. Hazard ratio and two-sided 95% CI estimated using the Cox regression with baseline stratification factors as covariates will be provided. Number and proportion of participants with worsening after alleviation of baseline COVID-19 symptoms will be summarized.

Time-weighted average change in SARS-CoV-2 viral load from baseline to Day 7 will be summarized by treatment groups and compared between treatment groups using an ANCOVA model with baseline viral load as covariate, the analysis will be based on the Virology Analysis Set.

6.3. Exploratory Endpoints

- Time to alleviation (mild or absent) of baseline symptoms in each domain of COVID-19-adapted FLU-PRO Plus
  - The alleviation of baseline symptoms in each domain is defined as:
    - Symptoms scored as 2 or higher at baseline are scored as 0 or 1 at postbaseline, AND
    - Symptoms scored as 1 at baseline are scored as 0 at postbaseline, AND
    - for two consecutive days.

If a participant has no symptom in a domain, the alleviation status is missing for the domain.

- Time to alleviation of symptoms in each domain is defined (in days) as:

  First Date of achieving alleviation - First dose Date + 1

  If a participant has not achieved symptom alleviation at last FLU-PRO Plus assessment or early discontinuation of study, the participant will be censored at last FLU-PRO Plus assessment date of the domain.

- Change from baseline in COVID-19-adapted FLU-PRO Plus total score and score in each domain

- Time-weighted average change in SARS-CoV-2 viral load from baseline to Day 14

- Time to first negative SARS-CoV-2 PCR

- Negative SARS-CoV-2 PCR based on RT-PCR viral load is defined as:

  (1) the results reported as ‘No SARS-CoV-2 detected’, AND

  (2) two consecutive negative results, or negative at last available sample for participants who completed or discontinued from study.

- The time to negative SARS-CoV-2 PCR is defined (in days) as the number of days to first confirmed negative:

  First date of two consecutive dates achieving negative result – First dose date +1.

Participants without negative SARS-CoV-2 PCR will be censored at last non-missing SAS-CoV-2 viral load date.

- Proportion of participants with negative SARS-CoV-2 PCR at each study visit

- Proportion of participants admitted to the intensive care unit by Day 28

- Proportion of participants started on mechanical ventilation by Day 28, a listing instead of summary table will be provided due to very small number of participants started on mechanical ventilation.

- Psychometric validity of COVID-19-adapted FLU-PRO Plus questionnaire, Emergence of viral resistance to RDV and Baseline levels and change from baseline for inflammation/immune-related, acute respiratory distress syndrome (ARDS)-related and coagulation-related biomarkers will be analyzed separately

6.3.1. Analysis of Exploratory Endpoints

The Virology Analysis Set will be the primary analysis set for SARS-CoV-2 endpoints, the FAS will be the primary analysis set for the rest of exploratory endpoints. Analyses may be repeated for the mFAS.

The Kaplan-Meier product limit method will be used to estimate and log-rank test will be used to compare treatment groups for the time to first negative SARS-CoV-2 PCR and time to alleviation (mild or absent) of baseline symptoms in each domain. Hazard ratio and two-sided 95% CI estimated using the Cox regression with baseline stratification factors as covariates will be provided. Time-weighted average change in SARS-CoV-2 viral load from baseline to Day 14 will be summarized by treatment groups and compared between treatment groups using an ANCOVA model. Count and percent of participants admitted to the intensive care unit by Day 28 will be summarized and compared between treatment groups using the Fisher Exact test. A listing will be provided for participants started on mechanical ventilation by Day 28.

Change from baseline in COVID-19-adapted FLU-PRO Plus total score and score in each domain from Day 1 to Day 14 at each day will be provided. Change in SARS-CoV-2 viral load from baseline at each postbaseline analysis window will be provided. Descriptive statistics will be provided by treatment group as follows: (1) Baseline values, (2) Values at each postbaseline analysis window, (3) Change from baseline at each postbaseline analysis window.

Count and percentage of participants with negative SARS-CoV-2 viral load at each visit will be summarized by treatment group and compared between treatment groups using the Fisher Exact test.

6.4. Change from Protocol Specified Efficacy Analyses

The protocol states that the proportion of participants progressing to requiring oxygen supplementation by Day 28 will be estimated using the Kaplan-Meier method and compared between the 2 treatment groups using a log-rank test. In the SAP, the number and percentage of participants progressing to requiring oxygen supplementation by Day 28 will be summarized and compared between treatment groups using the Fisher Exact test due to very small number of participants progressing to requiring oxygen supplementation.

7. SAFETY ANALYSES

7.1. Adverse Events and Deaths

Safety data will be summarized for the participants in the safety analysis set. All safety data collected on or after the date that study drug was first dispensed through 30 days after last dose will be summarized by treatment group for the Safety Analysis Set, unless specified otherwise. All safety data will be included in data listings.

7.1.1. Adverse Event Dictionary

Clinical and laboratory adverse events (AEs) will be coded using the current version of MedDRA. System organ class (SOC), high-level group term (HLGT), high-level term (HLT), preferred term (PT), and lower-level term (LLT) will be provided in the AE dataset.

7.1.2. Adverse Event Severity

The severity of AEs will be graded using the Division of AIDS (DAIDS) Table for Grading the Severity of Adult and Pediatric Adverse Events, Version 2.1 dated July 2017. For each episode, the highest grade attained should be reported as defined in the grading scale. The DAIDS scale is available at the following location:

https://rsc.niaid.nih.gov/sites/default/files/daidsgradingcorrectedv21.pdf

Adverse events are graded by the investigator as Grade 1 (mild), Grade 2 (moderate), Grade 3 (severe), Grade 4 (life threatening) or Grade 5 (fatal) according to toxicity criteria specified in the document above. The severity grade of events for which the investigator did not record severity will be left as “missing” for data listings.

7.1.3. Relationship of Adverse Events to Study Drug

Related AEs are those for which the investigator selected “Related” on the AE CRF to the question of “Related to Study Treatment.” Relatedness will always default to the investigator’s choice, not that of the medical monitor. Events for which the investigator did not record relationship to study drug will be considered related to study drug for summary purposes. However, by-subject data listings will show the relationship as missing.

7.1.4. Relationship of Adverse Events to Study Procedure

Study procedure-related AEs are those for which the investigator selected “Yes” on the AE CRF to the question of “Related to Study Procedures.” Events for which the investigator did not record relationship to study procedure will be considered as missing. No summary table for relationship of AE to study procedure will be presented.

7.1.5. Serious Adverse Events

Serious adverse events (SAEs) will be identified and captured as SAEs if the AEs met the definitions of SAEs that were specified in the study protocol. SAEs captured and stored in the clinical database will be reconciled with the SAE database from the Gilead Global Patient Safety (GLPS) Department before data finalization.

7.1.6. Treatment-Emergent Adverse Events

Proportion of participants with treatment-emergent AEs (TEAEs) is the primary safety endpoint of the study. No statistical comparison will be provided for proportion of participants with TEAE.

7.1.6.1. Definition of Treatment-Emergent Adverse Events

Treatment-emergent adverse events (TEAEs) are defined as 1 or both of the following:

- Any AEs with an onset date on or after the study drug start date and no later than 30 days after permanent discontinuation of study drug
- Any AEs leading to premature discontinuation of study drug.

7.1.6.2. Incomplete Dates

If the onset date of the AE is incomplete and the AE stop date is not prior to the first dosing date of study drug, then the month and year (or year alone if month is not recorded) of onset determine whether an AE is treatment emergent. The event is considered treatment emergent if both of the following 2 criteria are met:

- The AE onset date is the same as or after the month and year (or year) of the first dosing date of study drug, and
- The AE onset date is the same as or before the month and year (or year) of the date corresponding to 30 days after the date of the last dose of study drug

An AE with completely missing onset and stop dates, or with the onset date missing and a stop date later than the first dosing date of study drug, or with the onset date missing and the AE is marked as ongoing, will be considered to be treatment emergent. In addition, an AE with the onset date missing and incomplete stop date with the same or later month and year (or year alone if month is not recorded) as the first dosing date of study drug will be considered treatment emergent.

7.1.7. Summaries of Adverse Events and Deaths

Treatment-emergent AEs will be summarized based on the Safety Analysis Set.

The number and percentage of participants who experienced at least 1 TEAE will be provided and summarized by SOC, High lever term (HLT), PT, and treatment group. For other AEs described below, summaries will be provided by system organ class, PT, and treatment group:

- Grade 3 or higher treatment-emergent AEs
- All treatment-emergent study drug-related AEs
- Grade 3 or higher treatment-emergent study drug-related AEs
- All treatment-emergent SAEs
- All treatment-emergent study drug-related SAEs
- All treatment-emergent AEs that caused premature discontinuation from study drug

TEAE and study drug related TEAE will summarized by system organ class, PT, Severity and treatment group.

A brief, high-level summary of the number and percentage of participants who experienced at least 1 TEAE in the categories described above, and treatment emergent deaths will be provided by treatment group. Treatment-emergent death refers to deaths that occurred between the first dose date and the last dose date plus 30 days (inclusive).

Multiple events will be counted only once per participant in each summary. Adverse events will be summarized and listed first in alphabetic order of SOC and HLT within each SOC (if applicable), and then by PT in descending order of total frequency within each SOC. For summaries by severity grade, the most severe grade will be used for those AEs that occurred more than once in an individual participant during the study.

In addition to the above summary tables, all treatment-emergent AEs, treatment-emergent AEs with Grade 3 or higher, treatment-emergent study drug-related AEs, and treatment-emergent study drug-related AEs with Grade 3 or higher, will be summarized by PT only, in descending order of total frequency.

In addition, data listings will be provided for the following:

- All AEs
- Study-Drug-Related AEs
- AEs with severity of Grade 3 or higher
- SAEs

- Study-Drug-Related SAEs
- Deaths
- AEs leading to premature discontinuation of study drug

7.2. Laboratory Evaluations

Laboratory data collected during the study will be analyzed and summarized using both quantitative and qualitative methods. Summaries of laboratory data will be provided for the Safety Analysis Set and will include data collected up to the last dose of study drug plus 30 days. The analysis will be based on values reported in conventional units. When values are below the LOQ, they will be listed as such, and the closest imputed value will be used for the purpose of calculating summary statistics as specified in Section 3.7.. SAR-COV-2 PCR result of "Inconclusive" will not be included in numeric summary and will be considered as not negative result for PCR negative confirmation.

A by-subject listing for laboratory test results will be provided by subject ID number and visit in chronological order for hematology, coagulation, and serum chemistry separately. Values falling outside of the relevant reference range and/or having a severity grade of 1 or higher on the DAIDS Grading Scale will be flagged in the data listings, as appropriate.

7.2.1. Summaries of Numeric Laboratory Results

Descriptive statistics will be provided by treatment group for each laboratory test specified in the study protocol as follows:

- Baseline values
- Values at each postbaseline analysis window
- Change from baseline at each postbaseline analysis window
- Percentage change from baseline to each postbaseline analysis window (if specified)

A baseline laboratory value will be defined as the last nonmissing measurement obtained on or prior to the date/time of first dose of study drug. Change from baseline to a postbaseline visit will be defined as the visit value minus the baseline value. The mean, median, Q1, Q3, minimum, and maximum values will be displayed to the reported number of digits; SD values will be displayed to the reported number of digits plus 1.

In the case of multiple values in an analysis window, data will be selected for analysis as described in Section 3.8.3.

7.2.2. Graded Laboratory Values

The DAIDS Table for Grading the Severity of Adult and Pediatric Adverse Event, Version 2.1 (July 2017) will be used for assigning toxicity grades (0 to 4) to laboratory results for analysis. Grade 0 includes all values that do not meet the criteria for an abnormality of at least Grade 1. For laboratory tests with criteria for both increased and decreased levels, analyses for each direction (i.e., increased, decreased) will be presented separately.

For baseline, if both local lab and central lab results are available, central lab results will be selected first for baseline toxicity grade, local lab results will be used when central lab results are missing.

7.2.2.1. Treatment-Emergent Laboratory Abnormalities

Treatment-emergent laboratory abnormalities are defined as values that increase at least 1 toxicity grade from baseline at any postbaseline time point, up to and including the date of last dose of study drug plus 30 days. If the relevant baseline laboratory value is missing, any abnormality of at least Grade 1 observed within the time frame specified above will be considered treatment emergent with the exception of Glucose (See Appendix 2 for details.)

7.2.2.2. Summaries of Laboratory Abnormalities

Laboratory data that are categorical will be summarized using the number and percentage of participants in the study with the given response at baseline and each scheduled postbaseline visit.

The following summaries (number and percentage of participants) for treatment-emergent laboratory abnormalities will be provided by lab test and treatment group; participants will be categorized according to the most severe postbaseline abnormality grade for a given lab test:

- Treatment-emergent laboratory abnormalities
- Treatment-emergent Grade 3 or 4 laboratory abnormalities

For all summaries of laboratory abnormalities, the denominator is the number of participants with nonmissing postbaseline values up to 30 days after last dosing date.

A by-subject listing of all treatment-emergent laboratory analyses and treatment-emergent Grade 3 or 4 laboratory abnormalities will be provided by subject ID number and visit in chronological order.

Abnormalities in coagulation parameters will be graded for international normalized ratio (INR) and aPTT.

For the international normalized ratio (INR) of prothrombin time (PT) and aPTT, protocol specified toxicity grading scale depends on the upper limit of normal range (ULN). While the ULN of INR and aPTT depends on whether the subject is taking anticoagulant medication or not (ie, Not taking oral anticoagulant: 0.8 – 1.2; Taking oral anticoagulant: 2.0 – 3.0), this information is not collected by the reference laboratory. As a result, INR and aPTT will be graded by assuming subject is not taking an oral anticoagulant, which is a conservative approach that may lead to over-reporting of abnormalities for INR and aPTT.

7.3. Body Weight, Vital Signs and Respiratory Status

Descriptive statistics will be provided by treatment group for body weight and vital signs (including heart rate, respiratory rate on room air and blood pressure) as follows:

- Baseline value
- Values at each postbaseline analysis window for rate respiratory rate on room air or at each timepoint for heart rate and blood pressure
- Change from baseline at each postbaseline analysis window for rate respiratory rate on room air or at each timepoint for heart rate and blood pressure

Similar descriptive statistics will be provided by treatment group for SpO_2 on room air.

Two types of baseline will be defined for vital signs. An overall baseline value will be defined as the last available value collected on or prior to the date/time of first dose of study drug. Day 1 pre-dose baseline is defined the same as the overall baseline. A pre-dose baseline per day for each of dosing days 2-3 will use the “Pre-infusion” record on that day as baseline. Change from overall baseline to a postbaseline visit and change from pre-dose baseline for dosing days will be defined as the postbaseline value minus the respective baseline value.

In the case of multiple values in an analysis window, data will be selected for analysis as described in Section 3.8.3. No formal statistical testing is planned.

A by-subject listing of body weight, BMI, vital signs (including heart rate, temperature, and blood pressure), and respiratory status (including respiratory rate and SpO_2) will be provided by subject ID number and visit in chronological order.

7.4. Prior and Concomitant Medications

Medications collected at screening and during the study will be coded using the current version of the Gilead-modified World Health Organization (WHO) Drug dictionary.

Concomitant medications are defined as medications taken while a participant took study drug. Use of concomitant medications will be summarized by preferred name using the number and percentage of participants for each treatment group. A participant reporting the same medication more than once will be counted only once when calculating the number and percentage of participants who received that medication. The summary will be ordered by preferred term in

descending overall frequency. For drugs with the same frequency, sorting will be done alphabetically.

For the purposes of analysis, any medications with a start date prior to or on the first dosing date of study drug and continued to be taken after the first dosing date, or started after the first dosing date but prior to or on the last dosing date of study drug will be considered concomitant medications. Medications started and stopped on the same day as the first dosing date or the last dosing date of study drug will also be considered concomitant. Medications with a stop date prior to the date of first dosing date of study drug or a start date after the last dosing date of study drug will be excluded from the concomitant medication summary. If a partial stop date is entered, any medication with the month and year (if day is missing) or year (if day and month are missing) prior to the date of first study drug administration will be excluded from the concomitant medication summary. If a partial start date is entered, any medication with the month and year (if day is missing) or year (if day and month are missing) after the study drug stop date will be excluded from the concomitant medication summary. Medications with completely missing start and stop dates will be included in the concomitant medication summary, unless otherwise specified. Summaries will be based on the Safety Analysis Set. No formal statistical testing is planned.

7.5. Other Safety Measures

A data listing will be provided for participants experiencing pregnancy during the study.

7.6. Changes from Protocol-Specified Safety Analyses

There are no deviations from the protocol-specified safety analyses.

8. PHARMACOKINETIC (PK) ANALYSES

8.1. PK Sample Collection

Sparse PK assessments will be conducted in all participants at participating sites and approximately 30 participants will have intensive PK assessments.

- Sparse PK assessments
  - Day 2: End of infusion and optional 2 hours post end of infusion
  - Day 3: Pre-dose (within 30 minutes of dosing) and end of infusion
- Intensive PK assessments

  Day 1 and Day 3: at the following time points relative to the start time of infusion: 0 (pre-dose), 0.5, 0.75, 3, 6, 8, 12 (optional) and 24 hours.

8.2. PK Analyses Related to Intensive PK Sampling

PK parameters will be determined for subjects in the PK Substudy Analysis Set. Concentrations of RDV and their metabolites in plasma will be determined using validated bioanalytical assays.

8.2.1. Estimation of PK Parameters

PK parameters will be estimated using Phoenix WinNonlin® software using standard noncompartmental methods. The linear/log trapezoidal rule will be used in conjunction with the appropriate noncompartmental model, with input values for dose level, dosing time, plasma concentration, and corresponding real time values, based on drug dosing times whenever possible.

All predose sample times before time-zero will be converted to 0.

For area under the curve (AUC), samples BLQ of the bioanalytical assays occurring prior to the achievement of the first quantifiable concentration will be assigned a concentration value of 0 to prevent overestimation of the initial AUC. Samples that are BLQ at all other time points will be treated as missing data in WinNonlin. The nominal time point for a key event or dosing interval (τ) may be used to permit direct calculation of AUC over specific time intervals. The appropriateness of this approach will be assessed by the PK scientist on a profile by profile basis.

Pharmacokinetic parameters such as $AUC_{0\text{-}24}$, λz and t1/2 are dependent on an accurate estimation of the terminal elimination phase of drug. The appropriateness of calculating these parameters will be evaluated upon inspection of PK data on a profile-by-profile basis by the PK scientist.

8.2.2. PK Parameters

PK parameters will be generated for all subjects in the PK Substudy Analysis Set. RDV, GS-704277, and GS-441524 will be evaluated if data are available.

The analytes and parameters presented in Table 8-1 will be used to evaluate the PK objectives of the study. The PK parameters to be estimated in this study are listed and defined in the PK Abbreviations section.

Table 8-1. Pharmacokinetic Parameters for Each Analyte

Analyte	Parameters
RDV	$AUC_{0\text{-}24}$, AUC_{last}, $t_{1/2}$, C_{max}, T_{max}, C_{last}, T_{last}, λ_z
GS-704277	$AUC_{0\text{-}24}$, AUC_{last}, $t_{1/2}$, C_{max}, T_{max}, C_{last}, T_{last}, λ_z
GS-441524	$AUC_{0\text{-}24}$, AUC_{last}, $t_{1/2}$, C_{max}, T_{max}, C_{last}, T_{last}, λ_z

Individual subject concentration data and individual subject PK parameters for RDV and their metabolites will be listed and summarized using descriptive statistics by treatment. Summary statistics (n, mean, SD, coefficient of variation [%CV], median, min, max, Q1, and Q3) will be presented for both individual subject concentration data by time point and individual subject PK parameters by treatment. Moreover, the geometric mean, 95% CI, and the mean and SD of the natural log-transformed values will be presented for individual subject PK parameter data.

Individual concentration data listings and summaries will include all subjects with concentration data. The sample size for each time point will be based on the number of subjects with nonmissing concentration data at that time point. The number of subjects with concentration BLQ will be presented for each time point. For summary statistics, BLQ values will be treated as 0 at predose and one-half of the lower LOQ for postdose time points.

Individual PK parameter data listings and summaries will include all subjects for whom PK parameter(s) can be derived. The sample size for each PK parameter will be based on the number of subjects with nonmissing data for that PK parameter.

The following tables will be provided for each analyte by treatment:

- Individual subject concentration data and summary statistics
- Individual subject plasma PK parameters and summary statistics

The following figures may be provided for each analyte by treatment:

- Mean (± SD) concentration data versus time (on linear and semilogarithmic scales)
- Median (Q1, Q3) concentration data versus time (on linear and semilogarithmic scales)

Individual, mean, and median postdose concentration values that are ≤ LOQ will not be displayed in the figures and remaining points connected.

PK sampling details by subject, including procedures, differences in scheduled and actual draw times, and sample age will be provided in listings.

8.3. PK Analyses Related to Sparse PK Sampling

Individual subject concentration data from Participants in the PK Analysis Set for RDV and their metabolites will be listed and summarized using descriptive statistics. Summary statistics (n, mean, SD, coefficient of variation [%CV], median, min, max, Q1, and Q3) will be presented by day and by nominal time point.

Sparse and intensive PK sampling data from this study may be combined with data from other studies in a meta-population analysis using mixed-effect modeling techniques. Details of the population PK analysis will be provided in a separate document.

9. REFERENCES

Gottlieb RL, Nirula A, Chen P, Boscia J, Heller B, Morris J, et al. Effect of Bamlanivimab as Monotherapy or in Combination With Etesevimab on Viral Load in Patients With Mild to Moderate COVID-19: A Randomized Clinical Trial. JAMA 2021.

Muller HH, Schafer H. A general statistical principle for changing a design any time during the course of a trial. Stat Med 2004;23 (16):2497-508.

10. SOFTWARE

SAS® Software Version 9.4. SAS Institute Inc., Cary, NC, USA.

Phoenix WinNonlin® 8.2. Pharsight Corporation, Princeton, NJ, USA.

11. SAP REVISION

Revision Date (DD MMM YYYY)	Section	Summary of Revision	Reason for Revision

12. APPENDICES

Appendix 1. Study Procedures Table[a]
Appendix 2. Programming Specifications

Appendix 1. Study Procedures Table[a]

	Screening	Baseline/ Day 1	Day 2	Day 3	Day 7 (± 1 day)	Day 14 (± 1 day)	Day 28[t] Follow-up (± 5 days)
Written Informed Consent	X						
Medical History[b]	X						
Complete Physical Examination[c]	X	X				X	
Clinical Symptom-Directed Physical Examination[d]			X	X	X		X
Height	X						
Vital Signs[e] and Weight	X	X[f]	X[f]	X[f]	X	X	X
Respiratory Status[g]	X	X	X	X	X	X	X
ALT, AST, Serum Creatinine, and Creatinine Clearance/eGFR[h]	X						
Chemistry[i], Hematology[j], and Coagulation[k] Panels		X		X	X	X	
Optional Genomic Testing[l]		X					
Urine Pregnancy Test[m]	X						
SARS-CoV-2 RT-qPCR Testing and Potential Resistance Testing[n]		X	X	X	X	X	
Documentation of SARS-CoV-2 Infection	X						
Biomarker Sample Collection[o]		X		X		X	
Sparse PK[p]			X	X			
Intensive PK[q]		X		X			
Medically Attended Visit Information[r]		X	X	X	X	X	X
FLU-PRO Plus Questionnaire[s]		X	X	X	X	X	
Study Drug Dosing		X	X	X			
Adverse Events and Concomitant Medications	X	X	X	X	X	X	X

a Study visits may be performed in an outpatient setting, at the participant's home via tele-health, virtually or remotely, as permitted by local and institutional regulations. The Day 28 visit may be performed via a phone call.
b Medical history will include the date of first symptoms, overall symptoms, exposure source, demographics, baseline characteristics, allergies and all other medical history.
c A complete physical examination must include source documentation of general appearance and the following body systems: Head, neck, and thyroid; eyes, ears, nose, throat, mouth, and tongue; chest (excluding breasts); respiratory; cardiovascular; lymph nodes, abdomen; skin, hair, nails; musculoskeletal; and neurological. Urogenital and reproductive examination should only be completed if clinically indicated.
d Clinical symptom-directed physical examination will include at least cardiac and respiratory evaluation.
e Vital signs include heart rate, temperature, and blood pressure.

f On Days 1, 2 and 3, vital signs to be completed pre-infusion, postinfusion, and when postinfusion observation is completed; weight from Screening may be used at Days 1-3.
g Respiratory status includes respiratory rate and SpO_2 on room air.
h Obtain ALT (and AST where available), serum creatinine, and creatinine clearance (calculated using Cockcroft-Gault or Schwartz formula; see Protocol Section 6.7.2) if not available within 90 days of screening, using a local laboratory.
i Chemistry: alkaline phosphatase, AST, ALT, total bilirubin, total protein, albumin, lactate dehydrogenase, creatine phosphokinase, bicarbonate, blood urea nitrogen, calcium, chloride, creatinine, glucose, lipase, magnesium, phosphorus, potassium, sodium, uric acid.
j Hematology: Complete blood count with differential.
k Coagulation: INR, PT, aPTT
l Optional genomic testing can occur at Day 1. If not collected at Day 1, it can be collected at subsequent visits.
m Urine pregnancy test will only be done for women of childbearing potential.
n Nasopharyngeal swab and sputum samples will be collected and stored for SARS-CoV-2 RT-qPCR and potential resistance testing.
o Optional biomarkers: Serum and whole blood will be collected for participants that consent.
p Sparse PK will be collected from participants at participating sites at Day 2: end of infusion and optional 2 hours post end of infusion and Day 3: Pre-dose (within 30 minutes of dosing) and end of infusion.
q Intensive PK will be collected at selected sites and approximately 30 participants will have intensive PK assessments conducted.
r Medically attended visit information includes any interactions with health care professionals other than study staff or designees including hospitalization; in-person emergency, urgent, or primary care visits; or any other in-person visit attended by the participant and a health care profession. Identify the nature and cause of the visit.
s FLU-PRO Plus questionnaire should be completed daily from Day 1 through Day 14 (if available).
t Only AEs, symptom assessment, medically attended visit information, and concomitant medications review are needed if the visit is done by phone.

Appendix 2. Programming Specifications

1) If the age from the Day 1 eCRF is not available, age will be calculated as follows:

   Only year is provided for the date of birth (DOB). Use July 1 for the month and day.

   a) AGE (years) is calculated from the number of days between the DOB and Study Day 1,

   b) Use the SAS INTCK function to determine the number of "1st-of-month days" (eg, January 1st, February 1st, March 1st) between DOB and Day 1 (inclusive),

   c) Divide the result in (b) by 12,

   AGE = the integer of the result in (c),

   Age for laboratory test reference range will be based on the age at the sample collection date.

2) All screened participants refer to all participants who are screened (ie, with non-missing screening date) and have a screening number. For summaries the same participant is counted only once.

3) Screen failure participants are the participants who were screened and answered "No" for any inclusion criteria or "Yes" for any exclusion criteria regardless of which version of protocol the participant was consented to.

4) Participants in the randomized analysis set are defined as participants randomized into the study. IXRSRAND is the source to determine whether the subject is randomized (ie, participant with non-missing RGMNDTN in the IXRSRAND dataset), and confirmed by the eCRF ENROLL dataset (ie, ENROLLYN = "Yes" in ENROLL dataset).

5) Randomized treatment (ie, TRT01P in ADSL) is derived from IXRSRAND, while actual treatment received (ie, TRT01A in ADSL) is assigned as the randomized treatment if participant took at least 1 dose of study drug and assigned as blank if the participant was never dosed.

6) In the disposition table, the reasons for premature discontinuation are displayed in the order as they appear on the eCRF.

7) Body mass index (BMI)

   BMI will be calculated only at baseline as follows:

   — BMI = (weight [kg]) / (height [meters]^2)

   Baseline height and weight will be used for this calculation if available.

8) For demographics tables, "Not Permitted" will be excluded from percentage for detailed race categories summary (i.e. race categories collected on eCRF). For combined Race Category (e.g. Asian, White, Black, Other), "Not Permitted" is included in "Other" and "Other" will be include in the count of percentage.

9) TEAE

Events with Missing Onset Day and/or Month

An event is considered treatment emergent if all of the following 3 criteria are met:

i. The month and year (or year) of onset date is the same as or after the month and year (or year) of the first dose of study drug, and

ii. The month and year (or year) of the onset date is the same as or before the month and year (or year) of the 30th day after the date of the last dose of study drug, and

iii. End date is as follows:

The (complete) end date is on or after the first dose date, or

The month and year (or year) of end date is the same or after the month and year (or year) of the first dose of study drug, or

End date is completely missing

Events with Completely Missing Onset Date

An AE with a completely missing onset date is defined as TEAE if end date meets any of the 3 criteria specified above.

10) The precision in reporting numerical values should be as follows:

Raw measurements will be reported the same as the data captured electronically or on the eCRF for all but hematology and chemistry values.

Standard deviation and standard error will be reported to one more significant decimal place than the raw measurement.

Mean, median, minimum, Q1, Q3, and maximum will be reported to the same number of decimal places of the raw measurements.

Exceptions may be considered; for example, if more than 4 significant digits are provided for the measurement.

Precision for derived parameters as follows:

Parameters	Decimal Places
log 10 of SARS-CoV-2 viral load	2
Time-weighted average change in SARS-CoV-2 viral load	2
BMI	2
PK concentration	2

11) Last dose date is not expected to be missing. However, if last dose date is missing, it will be imputed using the maximum of non-missing, non-zero dose, study drug start and stop dates.

12) Incomplete death dates will be imputed as the maximum of the study drug start dates, study drug end dates , clinic or phone visit dates (lvis28dt), laboratory visit dates (llab28dt), including the 28-day follow-up visit date plus 1.

13) Censoring rules

Time to hospitalization/death: If a participant prematurely discontinues from the study prior to Day 28 or the hospitalization status is missing, the participant is censored at last study date or day 28 whichever is earlier.

Time to negative SARS-CoV-2 viral load, participants are censored at the last assessment day and participants are required to have at least one postbaseline assessment.

Time to alleviation of baseline COVID-19 symptoms:

- For overall baseline symptom alleviation, participants are censored at the last assessment day that at least one of the baseline symptoms was assessed. For baseline symptom alleviation for each domain, participants are censored at the last assessment day that at least one of the baseline symptoms within that domain was assessed

14) Graded Laboratory Abnormalities Summary

The following labels will be used for laboratory abnormalities and Grade 3 or 4 laboratory abnormalities summary tables and listings:

Battery	Lab Test Label Used in l-labtox Listing	Toxicity Direction	Lab Test Label Used in t-labtox Table
Hematology	Hemoglobin	Decrease	Hemoglobin (Decreased)
	Lymphocytes	Decrease	Lymphocytes (Decreased)
	Neutrophils	Decrease	Neutrophils (Decreased)
	Platelets	Decrease	Platelets (Decreased)
	WBC	Decrease	WBC (Decreased)
Chemistry	Albumin	Decrease	Albumin (Decreased)
	Alkaline Phosphatase	Increase	Alkaline Phosphatase (Increased)
	ALT	Increase	ALT (Increased)
	AST	Increase	AST (Increased)
	Bicarbonate	Decrease	Bicarbonate (Decreased)
	Corrected Calcium	Increase	Corrected Calcium (Hypercalcemia)
	Corrected Calcium	Decrease	Corrected Calcium (Hypocalcemia)
	Creatine Kinase (CK)	Increase	Creatine Kinase (Increased)
	Creatinine	Increase	Creatinine (Increased)
	Creatinine Clearance	Decrease	Creatinine Clearance (Decreased)
	Lipase	Increase	Lipase (Increased)
	Magnesium	Decrease	Magnesium (Hypomagnesemia)
	Phosphate	Decrease	Phosphate (Hypophosphatemia)
	Serum Glucose (Fasting)	Increase	Serum Glucose (Fasting, Hyperglycemia)
	Serum Glucose (Nonfasting)	Increase	Serum Glucose (Nonfasting, Hyperglycemia)
	Serum Glucose	Decrease	Serum Glucose (Hypoglycemia)
	Serum Potassium	Increase	Serum Potassium (Hyperkalemia)
	Serum Potassium	Decrease	Serum Potassium (Hypokalemia)
	Serum Sodium	Increase	Serum Sodium (Hypernatremia)
	Serum Sodium	Decrease	Serum Sodium (Hyponatremia)
	Total Bilirubin	Increase	Total Bilirubin (Hyperbilirubinemia)
	Uric Acid	Increase	Uric Acid (Hyperuricemia)
Coagulation	Prothrombin Intl. Normalized Ratio (INR)	Increase	Prothrombin Intl. Normalized Ratio (Increased)
	Activated partial thromboplastin time (aPTT)	Increase	Activated partial thromboplastin time (Increased)

15) SAS code for ANCOVA:

```
ods output ParameterEstimates=out1 LSMeans=out2 LSMeanCL=out3
LSMeanDiffCL=out4;
proc glm data=dat1 plots=none;
  class trt01pn;
  model DAVG = trt01pn base / solution;
  lsmeans trt01pn / stderr cl pdiff;
  run;
```

```
ods output close; quit;
```

16) Symptom scores in the FLU-PRO questionnaire raw data start from 1 to n (total number of responses in a question), in the FLU-PRO user manual, symptom scores start from 0. In the analysis dataset, symptom scores will be re-mapped to match the scoring in the FLU-PRO user manual for proper calculation of total score and domain score.

17) FLU-PRO Total Score and Domain Score:

The presence and severity of influenza signs and symptoms are assessed across 7 body systems: Nose (4 items), Throat (3 items), Eyes (3 items), Chest/Respiratory (7 items), Gastrointestinal (4 items), Body/Systemic (11 items), and Sense (2 itmes). For 29 of the items, the severity scale is as follows: 0 (“Not at all), 1 (“A little bit”), 2 (“Somewhat”), 3 (“Quite a bit”), and 4 (“Very much”). For 5 items, severity is assessed in terms of frequency, i.e., vomiting or diarrhea (0 times, 1 time, 2 times, 3 times, or 4 or more times); with frequency coughed up mucus or phlegm evaluated on a scale from 0 (“Never”) to 4 (“Always”). For 2 items, severity is assessed as 0 (“No”) or 1 (“Yes”).

Domain	Items	Scoring	Minimum Data Requirement
Nose	Runny or dripping nose<br>Congested or stuffy nose<br>Sneezing<br>Sinus pressure	Arithmetic mean of 4 items within **Nose** domain	Daily score for 3 of 4 items must be present to calculate domain score
Throat	Scratchy or itchy throat<br>Sore or painful throat<br>Difficulty swallowing	Arithmetic mean of 3 items within **Throat** domain	Daily score for 2 of 3 items must be present to calculate domain score
Eyes	Teary or watery eyes<br>Sore or painful eyes<br>Eyes sensitive to light	Arithmetic mean of 3 items within **Eyes** domain	Daily score for 2 of 3 items must be present to calculate domain score
Chest/Respiratory	Trouble breathing<br>Chest congestion<br>Chest tightness<br>Dry or hacking cough<br>Wet or loose cough<br>Coughing<br>Coughed up mucus or phlegm	Arithmetic mean of 7 items within **Chest/Respiratory** domain	Daily score for 5 of 7 items must be present to calculate domain score
Gastrointestinal	Felt nauseous<br>Stomach ache<br>How many times did you vomit?<br>How many times did you have diarrhea?	Arithmetic mean of 4 items within **Gastrointestinal** domain	Daily score for 3 of 4 items must be present to calculate domain score
Body/Systemic	Headache<br>Head congestion<br>Felt dizzy<br>Lack of appetite<br>Sleeping more than usual	Arithmetic mean of 11 items within **Body/Systemic** domain	Daily score for 8 of 11 items must be present to calculate domain score

Domain	Items	Scoring	Minimum Data Requirement
	Body aches or pains<br>Weak or tired<br>Chills or shivering<br>Felt cold<br>Felt hot<br>Sweating		
Sense*	Loss Smell<br>Loss Taste	Arithmetic mean of 2 items within **Sense** domain	Daily score for 1 of 2 items must be present to calculate domain score
Total	All above 34 items	Arithmetic mean of all 34 items within FLU-PRO	In the presence of missing data, the above conditions for the calculation of all domain scores must be met in order to calculate the FLU-PRO total score.

* Added for COVID-19

18) Symptom alleviation and time to baseline symptom alleviation

1. Baseline symptoms: Baseline symptoms are the symptoms collected prior to dosing and score ≥1.

   - Item 1 to 34 of the FLUPRO are symptoms. Global assessments (last 6 items in the questionnaire) are not symptoms.
   - Each subject's baseline symptoms including number of symptoms likely are different.
   - For alleviation of baseline symptoms, only need to follow the symptoms presented (≥1) at baseline
     - If a participant has 5 baseline symptoms, only the 5 symptoms will be followed to derive alleviation.
     - If a symptom (e.g. coughing) is not one of the baseline symptoms and has a score >1 post baseline, the symptom will be not considered to derive the subject's baseline symptom alleviation.
     - If participant has not symptom ≥1 at baseline, alleviation status for the participant is missing for all visit.
     - Baseline symptoms ≥1, symptoms from Day 2 and later are all missing, the alleviation status for the symptom is missing for all visits.

2. Alleviation of a symptom:

   - Symptom scored as 2 or higher at baseline are scored as 0 or 1 at postbaseline
   - Symptom scored as 1 at baseline are scored as 0 at postbaseline
   - for two consecutive days - need to be confirmed with 2 visits

- one missing day between two visits (that meets the alleviation definition) is allowed, but 2 or more days of missing is not considered reach alleviation
- for Day 14 (or last assessment on Day x), if the symptom meets definition of alleviation, and day 13 (or Day x -1) also meets the definition of alleviation, then it is considered as confirmed. One missing day is allowed for the confirmation of Day 14 or last assessment, i.e if day 13 (or Day x-1) is missing and Day 12 (or Day x-2) meets the definition of alleviation, then it is considered as confirmed.

Examples:

	Day														Alleviation for a symptom on day	
**Example**	**1**	**2**	**3**	**4**	**5**	**6**	**7**	**8**	**9**	**10**	**11**	**12**	**13**	**14**		
1	2	2	2	1	1	1	0	0	0	0	0	0	0	0	4	*baseline 2 or higher needs to be 1 or 0*
2	2	2	2	0	1	1	0	0	0	0	0	0	0	0	4	*baseline 2 or higher needs to be 1 or 0*
3	2	2	2	0	2	1	1	0	0	0	0	0	0	0	6	*day 4 is not confirmed*
4	3	2	2	1	1	1	0	2	1	1	0	0	0	0	4	*still on day 4 if worsen after confirmation*
5	1	1	1	0	0	0	1	0	1	1	0	0	0	0	4	*Worsening on day7*
6	1	1	1	1	0	0	0	0	0	0	0	0	0	0	5	*baseline 1 needs to be 0*
7	2	2	2	1	.	1	0	0	0	0	0	0	0	0	4	*allow one missing*
8	2	2	2	1	.	.	1	1	.	0	0	0	0	0	7	*day 4 is not confirmed if there are two or more days' response missing, re-start the check for confirmation*
9	2	2	2	2	2	2	2	2	4	2	2	2	2	1	14	*censor*
10	2	2	2	2	2	2	2	2	2	2	2	2	1	.	13	*censor*
11	2	2	2	2	2	2	2	2	2	2	2	2	1	1	13	*Confirmed on day 13 and 14*
12	2	2	2	2	2	2	2	2	1	.	.	.	.	.	9	*censor*
13	2	2	2	2	2	2	2	2	1	1	.	.	.	.	9	*Confirmed on day 9 and 10*

Yellow - Confirmed alleviation, Tan - Not confirmed, Blue - Worsening

3. Alleviation of baseline symptoms for a participant

The alleviation status for a participant at each visit is

- Yes, if all confirmed baseline symptom alleviation status are Yes.
- if > 25% of symptoms are missing, then alleviation status is No, else ignore missing.
- No, if any baseline symptom alleviation status is No.

4. Tim to alleviation of baseline symptoms

First day of participant level alleviation status equals Yes. If a participant has not achieved symptom alleviation at last FLuPRO assessment, the participant will be censored at the date of last FLUpro assessment. For symptom alleviation of a domain, the last assessment date is only among those baseline symptoms within the domain.

19) Covid-19 medications include:

Chloroquine, Hydroxychloroquine, IV remdesivir, Bamlanivimab, Casirivimab/Imdevimab, Molnupiravir, Lopinavir-ritonavir, Ribavirin. Details of COVID-19 medications are provided in table below.

**Drug name**	**ATC Code**	**WHODRUG Preferred Term**
Chloroquine	P01BA	CHLOROQUINE
Hydroxychloroquine	P01BA	HYDROXYCHLOROQUINE
Lopinavir/ritonavir	J05AR	LOPINAVIR/RITONAVIR
Ribavirin	J05AP	RIBAVIRIN
Remdesivir	J05AB	REMDESIVIR
Bamlanivimab	--	BAMLANIVIMAB
Casirivimab/Imdevimab	--	CASIRIVIMAB/IMDEVIMAB
Molnupiravir	--	MOLNUPIRAVIR

ATC = anatomical therapeutic chemical

Additional medication may be included during final review of Concomitant medications prior to data finalization.

20) Baseline risk factor

Selected medical history will be summarized as baseline risk factor and be included as subgroups in analysis. A risk factor for a participant is defined as a medical history of one of these diseases:

— At least 1 ongoing medical history record with MedDRA PT (mh.MDRPT) in the following selected PT listing for the corresponding disease with start date on or prior to the first dose date.

— At least 1 ongoing AE record with MedDRA PT (ae.MDRPT) in the following selected PT listing for the corresponding disease with start date on or prior to the first dose date

If the start date is incomplete but the month and year (or year alone) of the start date is the same as or before the month and year (or year alone) of the first dosing date of study drug, the event will be included. If the start date is completely missing, the event will be included.

A variable for each of the risk factors will be added to raw Medical History and Adverse Events datasets. A medical history or an AE record will be flagged for a risk factor if its MedDRA PT

included in the prespecified PT list for the corresponding disease of interest, which include all PTs from the narrow or broad search of the following SMQs under MedDRA 24.0 provided by Gilead GLPS and reviewed by Gilead medical monitors.

Disease of Interest	SMQ or MedDRA HLT Source
Chronic lung disease	HLT of bronchospasm and obstruction
Hypertension	Hypertension (SMQ)
Cardiovascular or cerebrovascular disease	Ischaemic central nervous system vascular conditions (SMQ); Myocardial infarction (SMQ);<br>Other ischaemic heart disease (SMQ)
Diabetes mellitus	Hyperglycaemia/new onset diabetes Mellitus (SMQ)
Immunocompromised state	HLGT for “Immunodeficiency syndromes”
Chronic mild or moderate kidney disease	Chronic kidney disease (SMQ)
Chronic liver disease	search name “Chronic liver disease excluding transient_acute events and nonspecific signs_symptoms
Current cancer	Malignancies (SMQ)
Sickle cell disease	HLGT “Haemoglobinopathies”

Risk factor of obesity will be defined as BMI >=30 kg/m^2 at baseline based on CRF data.

21) Common COVID-19 symptoms at baseline:

Common COVID-19 symptoms at baseline for each symptom will be considered as present if any of the corresponding FLU-PRO questionnaire items score 1 or higher at baseline.

Common COVID-19 symptoms	FLU-PRO questionnaire item
Stuffy or runny nose	Runny or dripping nose<br>Congested or stuffy nose
Sore throat	Sore or painful throat
Shortness of breath (difficulty breathing)	Trouble breathing
Cough	Coughing
Low energy or tiredness	Weak or tired
Muscle or body aches	Body aches or pains
Headache	Headache
Chills or shivering	Chills or shivering
Feeling hot or feverish	Felt hot
Nausea	Felt nauseous (feeling like you wanted to throw-up)

Vomit	How many times did you vomit?
Diarrhea	How many times did you have diarrhea?
Loss of Smell	Loss of smell
Loss of taste	Loss of taste

22) SpO2 and respiration rate: -

Summary of SpO2 and respiration rate will include measurements obtained while participants are on room air. SpO2 and respiration rate obtained while participants are on supplemental oxygen will be listed.

23) Glucose:

Fasting is not required per protocol for collecting Glucose sample, mixed fasting Glucose and non-fasting Glucose results are presented in the dataset. Lab abnormality for Glucose will be summarized as follows:

- if fasting glucose is collected at baseline, max TE lab toxicity grade is selected for post-baseline fasting glucose, max post-baseline toxicity grade (i.e. not TE toxicity grade) is selected for the post-baseline glucose regardless of fasting status.
- if non-fasting glucose is collected at baseline, TE lab toxicity grade is selected for post-baseline non-fasting glucose, max post-baseline toxicity grade is selected for the post-baseline glucose regardless of fasting status.
- Max TE lab toxicity grade for fasting glucose and non-fasting glucose, max post-baseline toxicity grade for fasting glucose and non-fasting glucose will be summarized separately for Hyperglycemia, and Serum Glucose (regardless fasting or non-fasting status) for Hypoglycemia in the lab abnormality summary table.
- In the any TE lab toxicity section of the lab abnormality table, max post-baseline toxicity grade will be included.

24) SARs-CoV2 PCR data

a) For numeric SARS-CoV2 summary (e.g, mean viral load, change from baseline etc.), "Inconclusive" SARs-CoV2 result is set to missing.

b) For categorical SARS-CoV2 summary, 3 categories will be included if appropriate, i.e. Positive, Inconclusive, Negative.

i) Positive = any numeric result or "<LLOQ SARSCoV2 detected" - LLOQ could vary by sample type

ii) Negative = "No SARS-CoV2 detected"

iii) Inconclusive = "Inconclusive"

c) For Negative SARS-CoV2 confirmation, "Inconclusive" SARs-CoV2 result will not be considered as missing thus a "Negative" followed by a "Inconclusive" is NOT confirmed.

d) 2 negative results from the same day do not equal confirmation.

e) if last PCR sample is negative for participants completed the study or for participant withdrawn early from the study, then one negative PCR of last sample is considered as confirmed negative.

f) Categorical SARS-CoV2 and SARS-CoV2 negative summary will be separated out by sample type. There will be no combined Positive or negative category using 2 or more sample types.

25) SAS code for Cox model with Covariates

```
proc phreg;
class trt01p (ref="Placebo to match RDV") strat1 strat2 strat3;
model aval*cnsr(1) = trt01p strat1 strat2 strat3 /risklimits = wald;
ods output  ParameterEstimates=Est1 ModelANOVA = pvout1;
run;
```

Hazard ratio is the hazardratio, 95% Cis are HRLowerCL and HRUpperCL where Parameter = "TRT01P" from EST1 dataset; P-value is ProbChiSq where Effect = "TRT01P" from pvout1 dataset.
