# Supplementary material for: Bayesian reanalysis of early remdesivir for the treatment of COVID-19 in outpatients with high risk of progression to severe disease
Source: PLoS One. 2026 Apr 16;21(4):e0346878. doi: 10.1371/journal.pone.0346878 (PMC13086435; doi:10.1371/journal.pone.0346878)
Supplement: S2 Appendix — The Cox proportional hazards model, the Bayesian model including development of the reference and data drive priors, and the Monte Carlo estimation of the parameters of interest are described. (PDF) [file pone.0346878.s003.pdf]

# PINETREE Bayesian Reanalysis - Supplementary Appendix A

Suppose $T$ is a random variable representing a survival time, with a smooth cumulative distribution function (cdf) $F_T(t)$, and a realization $t$:

$$F_T(t) = P(T \leq t)$$

Therefore, its probability density fuction (pdf) would be

$$p_T(t) = \frac{d}{dt}F_T(t)$$

The survival function $S(t)$ is the probability of surviving until at least time $t$, which is the complement of the cdf of the survival random variable $T$,

$$S(t) = 1 - F_T(t)$$

The hazard function $h(t)$ is the instantaneous risk of not surviving past time $t$ assuming survival until time $t$ given by

$$h(t) = \frac{p_T(t)}{S(t)} = \frac{p_T(t)}{1 - F_T(t)}$$

The cumulative hazard function $H(t)$ is defined to be the accumulated hazard over time

$$H(t) = \int_0^t h(u)\,\mathrm{d}u$$

There are many parametric survival models (e.g., the exponential) that specify explicit parametric distributions over the survival times. A semi-parametric survival model was introduced by Cox that is specified in terms of a hazard function $h(t)$ rather than a distribution over survival times [1]. This well-known and frequently utilized model does not model the full hazard function rather models the proportional differences in hazards among subjects.

Let $\boldsymbol{x}_n \in \mathbb{R}^K$ be a row vector of covariates for participant $n$. The covariance matrix (often called design matrix) is compilation of each of these rows. If there are $N$ participants, the matrix $\boldsymbol{X}$ will contain $N$ rows and $K$ columns, i.e., $\boldsymbol{X} \in \mathbb{R}^{N \times K}$. In the Cox proportional hazard model, the hazard function for

participant $n$ is conditional on its covariants $x_n$ and a parameter vector $\boldsymbol{\theta} \in \mathbb{R}^K$ with the same number of dimensions as the number of covariates:

$$h(t \mid \boldsymbol{x}_n, \boldsymbol{\beta}) = h_0(t) \exp(\boldsymbol{x}_n \boldsymbol{\theta})$$

Note that $h_0(t)$ is a baseline hazard function shared by all participants and $\boldsymbol{\beta} \in \mathbb{R}^K$ are the parameters of interest.

A partial likelihood (the probability of the data given the parameters $P(D \mid \boldsymbol{\beta})$) can be derived that accounts for the order of the survival times. With the details of the derivation omitted, the partial likelihood for each observed participant (i.e., participants who had an observed survival time) $n \in 1$ to $N^{\text{obs}}$ as defined by Breslow [2] is

$$P(D \mid \boldsymbol{\beta}) = P[\text{observed failures ordered } 1, \ldots, N^{\text{obs}} \mid \boldsymbol{x}, \boldsymbol{\beta}] = \prod_{n=1}^{N^{\text{obs}}} \frac{\exp(\boldsymbol{x}_n \boldsymbol{\beta})}{\sum_{n'=n}^{N} \exp(\boldsymbol{x}_{n'} \boldsymbol{\beta})}$$

The denominator includes both the observed and censored observations.

Our Bayesian survival model incorporated this likelihood in order to calculate the posterior probability distributions over the parameters: (1) treatment (i.e., remdesivir or placebo) and the three binary stratification factors (2) age (<60 years or ≥60 years), (3) country (United States or outside the United States), and (4) residence in a skilled nursing facility (SNF) (yes or no):

$$h(t \mid \boldsymbol{x}_n, \boldsymbol{\beta}) = h_0(t) \exp(x_{\texttt{treatment}} \beta_{\texttt{treatment}} + x_{\texttt{age}} \beta_{\texttt{age}} + x_{\texttt{country}} \beta_{\texttt{country}} + x_{\texttt{SNF}} \beta_{\texttt{SNF}})$$

The likelihood was then combined with the prior distribution $P(\boldsymbol{\beta})$ to yield posterior distribution, which is the probability of the parameters given the data:

$$P(\boldsymbol{\beta} \mid D) = \frac{P(D \mid \boldsymbol{\beta}) P(\boldsymbol{\beta})}{P(D)}$$

The prior distribution on the parameters for age, country, and SNF were left as minimally informative with only the prior on the parameter for treatment being changed for each analysis.

$$\beta_{\texttt{age}}, \beta_{\texttt{country}}, \beta_{\texttt{SNF}} \sim \mathcal{N}(0, 3)$$

The following prior distributions over $\beta_{\texttt{treatment}}$ were used:

| **Reference Priors** | **Mean HR** | **SD,** log **HR** | **Representation** |
|---|---|---|---|
| Minimally informative | 1 | 3 | $\mathcal{N}(0, 3)$ |
| Weakly skeptical | 1 | 1 | $\mathcal{N}(0, 1)$ |
| Moderately skeptical | 1 | 0.5 | $\mathcal{N}(0, 0.5)$ |
| Weakly pessimistic | 1.25 | 1 | $\mathcal{N}(\log 1.25, 1)$ |
| Weakly optimistic | 0.75 | 1 | $\mathcal{N}(\log 0.75, 1)$ |
| **Data-driven Priors** | | | |
| Mixture of all prior trial data | 0.43 | 0.92 | Mixture distribution |
| Mixture of DAA trial data | 0.14 | 0.41 | Mixture distribution |
| Mixture of non-DAA trial data | 0.70 | 0.62 | Mixture distribution |

HR = hazard ratio; SD = standard deviation; $\mathcal{N}(\mu, \sigma)$ = normal distribution with mean $\mu$ and standard deviation $\sigma$; DAA = direct-acting antivirals

Mixtures were calculated as follows. Using the metformin study as an example, we know that the metformin study has OR = 0.47 (95% CI, 0.20 to 1.11). We can generate this normal distribution with the 2.5th percentile at 0.2, the 97.5th percentile at 1.11 and the mean at 0.47. Recall that we work on the log odds scale. Therefore, $\mu = \log(0.47)$ and the standard deviation is the distance between $\log(0.2)$ and $\log(1.11)$ scaled by the standard normal distance $1.96 + 1.96$:

$$\mu_{\texttt{metformin}} = \log(0.47) \qquad \sigma_{\texttt{metformin}} = \frac{\log(1.11) - \log(0.2))}{3.919928}$$

Using this same method, we obtained the means and standard deviations for each of the curves we needed for the data driven priors. The densities of these normal distributions were then mixed, weighting the distributions using the sample sizes (total number of participants) of the trials from which they originated. Markov chain Monte Carlo was used to estimate the mixture using RStan [3], and code for the model was inspired by code in the Stan User's Guide [4]

Since the joint posterior distribution does not have a closed form, posterior samples were used to calculate the posterior probability of the HR being less than or greater than some value after the MCMC sampling. Let $\hat{\theta}^{(b)}$ be the $b$th sample for $b = 1, \ldots, B$ where $B$ is the number of iterations after the warm-up period. The Monte Carlo estimate of the posterior probability of the HR, e.g. $P(\hat{\theta} < 0.1 \mid D)$, is computed as

$$P(\hat{\theta} < 0.1) = \frac{1}{B} \sum_{b=1}^{B} \mathbb{I}(\theta^{(b)} < 0.1),$$

where $\mathbb{I}(\cdot)$ is an indicator function.

# References


[1] David R Cox. "Regression models and life-tables". In: *Journal of the Royal Statistical Society: Series B (Methodological)* 34.2 (1972), pp. 187–202.

[2] Norman E Breslow. "Analysis of survival data under the proportional hazards model". In: *International Statistical Review/Revue Internationale de Statistique* (1975), pp. 45–57.

[3] Stan Development Team. *RStan: the R interface to Stan.* Version 2.32.6. 2024. URL: `https://mc-stan.org` (visited on 02/28/2025).

[4] Stan Development Team. *Stan User's Guide.* Version 2.37. 2025. URL: `https://mc-stan.org` (visited on 12/03/2025).
