# Supplementary material for: Bayesian reanalysis of early remdesivir for the treatment of COVID-19 in outpatients with high risk of progression to severe disease
Source: PLoS One. 2026 Apr 16;21(4):e0346878. doi: 10.1371/journal.pone.0346878 (PMC13086435; doi:10.1371/journal.pone.0346878)
Supplement: S4 Appendix — The New England Journal of Medicine publication of the PINETREE trial titled “Early Remdesivir to Prevent Progression to Severe Covid-19 in Outpatients” (reference 9). (PDF) [file pone.0346878.s005.pdf]

The NEW ENGLAND JOURNAL of MEDICINE

ESTABLISHED IN 1812 JANUARY 27, 2022 VOL. 386 NO. 4

Early Remdesivir to Prevent Progression to Severe Covid-19 in Outpatients

R.L. Gottlieb, C.E. Vaca, R. Paredes, J. Mera, B.J. Webb, G. Perez, G. Oguchi, P. Ryan, B.U. Nielsen, M. Brown, A. Hidalgo, Y. Sachdeva, S. Mittal, O. Osiyemi, J. Skarbinski, K. Juneja, R.H. Hyland, A. Osinusi, S. Chen, G. Camus, M. Abdelghany, S. Davies, N. Behenna-Renton, F. Duff, F.M. Marty,* M.J. Katz, A.A. Ginde, S.M. Brown, J.T. Schiffer, and J.A. Hill, for the GS-US-540-9012 (PINETREE) Investigators†

ABSTRACT

BACKGROUND

Remdesivir improves clinical outcomes in patients hospitalized with moderate-to-severe coronavirus disease 2019 (Covid-19). Whether the use of remdesivir in symptomatic, nonhospitalized patients with Covid-19 who are at high risk for disease progression prevents hospitalization is uncertain.

METHODS

We conducted a randomized, double-blind, placebo-controlled trial involving nonhospitalized patients with Covid-19 who had symptom onset within the previous 7 days and who had at least one risk factor for disease progression (age ≥60 years, obesity, or certain coexisting medical conditions). Patients were randomly assigned to receive intravenous remdesivir (200 mg on day 1 and 100 mg on days 2 and 3) or placebo. The primary efficacy end point was a composite of Covid-19–related hospitalization or death from any cause by day 28. The primary safety end point was any adverse event. A secondary end point was a composite of a Covid-19–related medically attended visit or death from any cause by day 28.

RESULTS

A total of 562 patients who underwent randomization and received at least one dose of remdesivir or placebo were included in the analyses: 279 patients in the remdesivir group and 283 in the placebo group. The mean age was 50 years, 47.9% of the patients were women, and 41.8% were Hispanic or Latinx. The most common coexisting conditions were diabetes mellitus (61.6%), obesity (55.2%), and hypertension (47.7%). Covid-19–related hospitalization or death from any cause occurred in 2 patients (0.7%) in the remdesivir group and in 15 (5.3%) in the placebo group (hazard ratio, 0.13; 95% confidence interval [CI], 0.03 to 0.59; P=0.008). A total of 4 of 246 patients (1.6%) in the remdesivir group and 21 of 252 (8.3%) in the placebo group had a Covid-19–related medically attended visit by day 28 (hazard ratio, 0.19; 95% CI, 0.07 to 0.56). No patients had died by day 28. Adverse events occurred in 42.3% of the patients in the remdesivir group and in 46.3% of those in the placebo group.

CONCLUSIONS

Among nonhospitalized patients who were at high risk for Covid-19 progression, a 3-day course of remdesivir had an acceptable safety profile and resulted in an 87% lower risk of hospitalization or death than placebo. (Funded by Gilead Sciences; PINETREE ClinicalTrials.gov number, NCT04501952; EudraCT number, 2020-003510-12.)

The authors' full names, academic degrees, and affiliations are listed in the Appendix. Dr. Gottlieb can be contacted at robert.gottlieb@bswhealth.org or at Baylor University Medical Center, Center for Advanced Heart and Lung Disease, 3410 Worth St., Ste. 250, Dallas, TX 75246.

*Deceased.

†A complete list of the GS-US-540-9012 (PINETREE) Investigators is provided in the Supplementary Appendix, available at NEJM.org.

This article was published on December 22, 2021, at NEJM.org.

N Engl J Med 2022;386:305-15.
DOI: 10.1056/NEJMoa2116846
Copyright © 2021 Massachusetts Medical Society.

CME at NEJM.org

The New England Journal of Medicine is produced by NEJM Group, a division of the Massachusetts Medical Society.
Downloaded from nejm.org at Gilead Sciences on June 25, 2025. For personal use only.
No other uses without permission. Copyright © 2022 Massachusetts Medical Society. All rights reserved.

Coronavirus disease 2019 (Covid-19), the illness caused by severe acute respiratory syndrome coronavirus 2 (SARS-CoV-2), was first identified in December 2019 and rapidly progressed to a global pandemic.[1] Older persons and those with coexisting conditions such as obesity, cardiovascular disease, and diabetes mellitus are at increased risk for hospitalization or death from Covid-19.[2,3] Some treatments such as monoclonal antibodies reduce the risk of progression of Covid-19. Additional therapeutic options could benefit patients and ease the burden on health care systems.

Remdesivir is a direct-acting nucleotide prodrug inhibitor of the SARS-CoV-2 RNA-dependent RNA polymerase; it has potent nanomolar activity in primary human airway epithelial cells.[4,5] A phase 3 trial of remdesivir showed that both a 10-day course and a 5-day course of remdesivir shortened the recovery time in patients hospitalized with Covid-19.[6,7] Early treatment of other acute viral infections improves clinical outcomes and reduces mortality,[8-11] and this strategy has also been proposed for Covid-19.[12] We hypothesized that earlier initiation of a short course of remdesivir treatment in outpatient settings would reduce hospitalizations and mortality. Here, we report the results of a double-blind, randomized, placebo-controlled trial that evaluated the efficacy and safety of a 3-day course of remdesivir in high-risk, nonhospitalized patients with Covid-19.

METHODS

PATIENTS

Eligible patients were 12 years of age or older and had at least one preexisting risk factor for progression to severe Covid-19 or were 60 years of age or older, regardless of whether they had other risk factors. Risk factors included hypertension, cardiovascular or cerebrovascular disease, diabetes mellitus, obesity (a body-mass index [BMI; the weight in kilograms divided by the square of the height in meters] of ≥30), immune compromise, chronic mild or moderate kidney disease, chronic liver disease, chronic lung disease, current cancer, or sickle cell disease. Eligible patients had at least one ongoing symptom consistent with Covid-19, with onset of the first symptom within 7 days before randomization (given that hospitalization typically occurs at or after 7 days of symptoms).[13,14] Eligible patients had SARS-CoV-2 infection confirmed by a molecular diagnostic assay within 4 days before screening (which corresponds with the period characterized by the highest viral loads).[15]

Patients were ineligible if they were receiving or were expected to receive supplemental oxygen or hospital care at the time of screening. Patients were also ineligible if they had had a previous hospitalization for Covid-19, had previously received treatment for Covid-19 (including investigational agents), or had received a SARS-CoV-2 vaccine. Full details are provided in the protocol and the statistical analysis plan (available with the full text of this article at NEJM.org).

TRIAL DESIGN AND OVERSIGHT

From September 18, 2020, through April 8, 2021, patients were enrolled at 64 sites in the United States, Spain, Denmark, and the United Kingdom. Trial sites included outpatient infusion facilities and skilled nursing facilities, and some participants received infusions at home. Patients were randomly assigned in a 1:1 ratio to receive intravenous remdesivir (200 mg on day 1 followed by 100 mg on days 2 and 3) or placebo. Randomization was stratified according to residence in a skilled nursing facility (yes or no), age (<60 years or ≥60 years), and country (United States or outside the United States). All patients and trial personnel were unaware of the trial-group assignments. Before undergoing trial procedures, the patients provided written informed consent. Assent and parental or guardian consent were obtained if the patients were younger than 18 years of age.

The trial was approved by the institutional review board or ethics committee at each trial site and was conducted in accordance with the Declaration of Helsinki, Good Clinical Practice guidelines, and local regulations. The trial was designed and conducted by the sponsor (Gilead Sciences) in collaboration with the principal investigators and in accordance with the protocol and amendments. The sponsor collected the data, monitored the trial conduct, and performed the statistical analyses. An interim analysis to be performed by the data and safety monitoring board was planned for when 50% enrollment was reached. The manuscript was prepared by a writer who was employed by Gilead Sciences, with input from all authors. All the authors had data confidentiality agreements with the spon-

The New England Journal of Medicine is produced by NEJM Group, a division of the Massachusetts Medical Society.
Downloaded from nejm.org at Gilead Sciences on June 25, 2025. For personal use only.
No other uses without permission. Copyright © 2022 Massachusetts Medical Society. All rights reserved.

sor. All the authors vouch for the accuracy and completeness of the data and for the fidelity of the trial to the protocol.

TRIAL ASSESSMENTS

Assessments included physical examinations, reporting of adverse events, blood testing, and the collection of nasopharyngeal swabs to quantify the SARS-CoV-2 viral load with the use of reverse-transcriptase–polymerase-chain-reaction (RT-PCR) assay at prespecified intervals. The electronic Covid-19–adapted Influenza Patient-Reported Outcome (FLU-PRO) Plus questionnaire (Evidera–PPD) was used to assess patient-reported symptoms. Patients completed the first questionnaire before the first infusion on day 1 and then daily through day 14. The symptom questionnaire was first available on October 21, 2020 (1 month after the start of enrollment). The full schedule of trial procedures is provided in the protocol.

END POINTS

The primary efficacy end point was a composite of hospitalization related to Covid-19 (as determined by site investigators, who were unaware of trial-group assignments, and defined as ≥24 hours of acute care) or death from any cause by day 28. The primary efficacy end point was initially a composite of hospitalization for any cause or death from any cause by day 14 and was modified on January 14, 2021, in response to comments from the Food and Drug Administration; trial blinding was maintained. The primary safety end point was any adverse event.

Secondary end points included the composite of Covid-19–related medically attended visits or death from any cause by days 14 and 28, Covid-19–related hospitalization by days 14 and 28, the time-weighted average change in nasopharyngeal SARS-CoV-2 viral load from baseline to day 7, and the time to alleviation of baseline Covid-19 symptoms (with alleviation defined as mild or absent symptoms) as compared with those reported on the baseline FLU-PRO Plus questionnaire completed before the first infusion. Post hoc analyses were also conducted of hospitalization for any cause by day 28 and time to alleviation of baseline Covid-19 symptoms as reported on the FLU-PRO Plus questionnaire completed on the day of the first infusion, either before or after the infusion.

STATISTICAL ANALYSIS

Assuming that 9.3% of patients would die or have a Covid-19–related hospitalization and 5% would drop out of the trial, we determined that a sample of 1264 patients would provide the trial with more than 90% power to detect a hazard ratio for Covid-19–related hospitalization or death from any cause of 0.55 for the comparison of remdesivir with placebo, with a two-sided significance level of 0.05. All patients who underwent randomization and received at least one infusion were included in the analyses (the full analysis set). Demographic characteristics, baseline measurements, adverse events, and laboratory abnormalities were summarized descriptively.

Hazard ratios, rate ratios, and two-sided 95% confidence intervals for the primary and secondary end points were calculated with the use of a Cox proportional-hazards model adjusted for the stratification factors of residence in a skilled nursing facility (yes or no), age (<60 years or ≥60 years), and country (United States or outside the United States). The P value for the primary efficacy end point was calculated with the use of the same method. The percentage of patients who were hospitalized with Covid-19 by day 28 was estimated with the use of a Kaplan–Meier analysis. The time to alleviation of baseline Covid-19 symptoms in the prespecified and post hoc analyses was estimated with the use of the Kaplan–Meier product-limit method. The time-weighted average change in viral load from baseline to day 7 was assessed with the use of analysis-of-covariance, with baseline viral load as a covariate. The widths of all calculated confidence intervals were not adjusted for multiplicity.

On April 6, 2021, an orderly closure of trial enrollment was announced by the sponsor because of administrative reasons related to a decrease in the incidence of SARS-CoV-2 infections, ethical concerns regarding assigning patients to placebo in the context of increased access to emergency-use–authorized treatments such as monoclonal antibodies, and increasing vaccination rates among high-risk persons. The last patient was enrolled on April 8, 2021. Of the 1264 patients who were expected to enroll, 562 (44.5%) had undergone randomization and had begun the trial regimen by the time enrollment was stopped. The data and safety monitoring board was informed that the trial had been stopped, and no interim analyses were performed before trial

The New England Journal of Medicine is produced by NEJM Group, a division of the Massachusetts Medical Society.
Downloaded from nejm.org at Gilead Sciences on June 25, 2025. For personal use only.
No other uses without permission. Copyright © 2022 Massachusetts Medical Society. All rights reserved.

discontinuation. Double blinding was maintained until finalization of the data.

RESULTS

PATIENTS

Of the 630 patients who were screened, 584 underwent randomization; 292 patients were assigned to each group. A total of 22 patients (13 who were assigned to receive remdesivir and 9 who were assigned to receive placebo) did not receive an infusion. The 562 patients who underwent randomization and received at least one infusion of remdesivir or placebo were included in the efficacy and safety analyses (279 patients in the remdesivir group and 283 in the placebo group) (Fig. S1 in the Supplementary Appendix, available at NEJM.org). Demographic and baseline clinical characteristics were balanced between the two groups (Table 1). Overall, 80.4% of the patients were White, 7.5% were Black, 6.4% were American Indian or Alaska Native, 47.9% were women, and 41.8% were Hispanic or Latinx; the mean age was 50 years, and the mean BMI was 31.0. A total of 30.2% of patients were 60 years of age or older, and the most common coexisting conditions were diabetes mellitus (61.6%), obesity (55.2%), and hypertension (47.7%). Eight patients (1.4%) younger than 18 years of age were enrolled. The median duration of symptoms before the first infusion was 5 days (interquartile range, 3 to 6), and the median time from SARS-CoV-2 confirmation to the time of screening was 2 days (interquartile range, 1 to 4). A total of 16.5% of the patients received at least one infusion at home, and 2.7% received at least one infusion in a skilled nursing facility.

EFFICACY

A total of 2 of 279 patients (0.7%) in the remdesivir group and 15 of 283 (5.3%) in the placebo group had a Covid-19–related hospitalization by day 28 (Table S1). All Covid-19–related hospitalizations occurred by day 14. No patients in either group died by day 28. The risk of Covid-19–related hospitalization or death from any cause by day 28 was 87% lower in the remdesivir group than in the placebo group (hazard ratio, 0.13; 95% confidence interval [CI], 0.03 to 0.59; P=0.008) (Table 2 and Fig. 1A). In prespecified subgroup analyses, the incidence of a primary efficacy end-point event was lower in the remdesivir group than in the placebo group (Fig. 2).

The incidence of Covid-19–related medically attended visits or death from any cause by day 28 was also lower in the remdesivir group than in the placebo group: 4 of 246 patients (1.6%) in the remdesivir group and 21 of 252 (8.3%) in the placebo group had a medically attended visit (hazard ratio, 0.19; 95% CI, 0.07 to 0.56) (Fig. 1B). By day 14, a total of 2 of 246 patients (0.8%) in the remdesivir group and 20 of 252 (7.9%) in the placebo group had a medically attended visit (hazard ratio, 0.10; 95% CI, 0.02 to 0.43).

A total of 5 of 279 patients (1.8%) in the remdesivir group and 18 of 283 (6.4%) in the placebo group were hospitalized for any reason by day 28. Results of a post hoc analysis showed a 72% lower risk of hospitalization for any cause by day 28 in the remdesivir group than in the placebo group (hazard ratio, 0.28; 95% CI, 0.10 to 0.75).

TIME TO ALLEVIATION OF SYMPTOMS

Of the 126 patients who completed the baseline FLU-PRO Plus questionnaire before the first infusion, 23 of 66 patients (34.8%) in the remdesivir group and 15 of 60 (25.0%) in the placebo group reported alleviation of symptoms by day 14 (rate ratio, 1.41; 95% CI, 0.73 to 2.69) (Fig. S2A). In a post hoc analysis involving patients who completed the baseline questionnaire any time on the day of the first infusion (either before or after the infusion), 61 of 169 patients (36.1%) in the remdesivir group and 33 of 165 (20.0%) in the placebo group reported alleviation of symptoms by day 14 (rate ratio, 1.92; 95% CI, 1.26 to 2.94) (Fig. S2B).

ANALYSES OF VIRAL LOAD

The mean (±SD) baseline nasopharyngeal viral load was 6.29±1.77 $\log_{10}$ copies per milliliter. In the remdesivir group, the mean nasopharyngeal SARS-CoV-2 viral load decreased from 6.31±1.75 $\log_{10}$ copies per milliliter at baseline to 4.11±1.36 $\log_{10}$ copies per milliliter at day 7. In the placebo group, the mean viral load decreased from 6.28±1.79 $\log_{10}$ copies per milliliter at baseline to 4.06±1.19 $\log_{10}$ copies per milliliter at day 7 (Fig. S3). The time-weighted average change in viral load from baseline to day 7 did not differ substantially between the two groups (−1.24 $\log_{10}$ copies per milliliter in the remdesivir group and −1.14 $\log_{10}$ copies per milliliter in the placebo

The New England Journal of Medicine is produced by NEJM Group, a division of the Massachusetts Medical Society.
Downloaded from nejm.org at Gilead Sciences on June 25, 2025. For personal use only.
No other uses without permission. Copyright © 2022 Massachusetts Medical Society. All rights reserved.

Table 1. Demographic and Clinical Characteristics of the Patients at Baseline.*

Characteristic	Remdesivir (N=279)	Placebo (N=283)	Total (N=562)
Age — yr	50±15	51±15	50±15
Age category — no. (%)			
≥60 yr	83 (29.7)	87 (30.7)	170 (30.2)
<18 yr	3 (1.1)	5 (1.8)	8 (1.4)
Female sex — no. (%)	131 (47.0)	138 (48.8)	269 (47.9)
Residence in the United States — no. (%)	264 (94.6)	267 (94.3)	531 (94.5)
Race or ethnic group — no. (%)†			
White	228 (81.7)	224 (79.2)	452 (80.4)
Black	20 (7.2)	22 (7.8)	42 (7.5)
American Indian or Alaska Native	15 (5.4)	21 (7.4)	36 (6.4)
Asian, Native Hawaiian, or Pacific Islander	7 (2.5)	7 (2.5)	14 (2.5)
Hispanic or Latinx	123 (44.1)	112 (39.6)	235 (41.8)
Other	3 (1.1)	2 (0.7)	5 (0.9)
Body-mass index	31.2±6.7	30.8±5.8	31.0±6.2
Coexisting conditions — no. (%)			
Diabetes mellitus	173 (62.0)	173 (61.1)	346 (61.6)
Obesity	154 (55.2)	156 (55.1)	310 (55.2)
Hypertension	138 (49.5)	130 (45.9)	268 (47.7)
Chronic lung disease	67 (24.0)	68 (24.0)	135 (24.0)
Current cancer	12 (4.3)	18 (6.4)	30 (5.3)
Cardiovascular or cerebrovascular disease	20 (7.2)	24 (8.5)	44 (7.8)
Immune compromise	14 (5.0)	9 (3.2)	23 (4.1)
Chronic kidney disease, mild or moderate	7 (2.5)	11 (3.9)	18 (3.2)
Chronic liver disease	1 (0.4)	1 (0.4)	2 (0.4)
Residence in skilled nursing facility — no. (%)	8 (2.9)	7 (2.5)	15 (2.7)
Median duration of symptoms before first infusion (IQR) — days	5 (3–6)	5 (4–6)	5 (3–6)
Median time since RT-PCR confirmation of SARS-CoV-2 (IQR) — days	2 (1–3)	3 (1–4)	2 (1–4)
Mean SARS-CoV-2 RNA nasopharyngeal viral load — $\log_{10}$ copies/ml‡	6.31±1.75	6.28±1.79	6.29±1.77

* Plus–minus values are means ±SD. IQR denotes interquartile range, RT-PCR reverse transcriptase–polymerase chain reaction, and SARS-CoV-2 severe acute respiratory syndrome coronavirus 2.
† Race and ethnic group were reported by the patients. Patients could have had more than one race or ethnic group.
‡ Data are shown for the virologic analysis set, which is defined in the statistical analysis plan (available with the protocol at NEJM.org): 215 of 279 patients (77.1%) in the remdesivir group and 213 of 283 patients (75.3%) in the placebo group.

group; least-squares mean difference, 0.07; 95% CI, −0.10 to 0.24).

SAFETY

By day 28, adverse events had occurred in 118 of 279 patients (42.3%) in the remdesivir group and in 131 of 283 (46.3%) in the placebo group (Table 3). The most common nonserious adverse events that occurred in at least 5% of patients in both groups were nausea, headache, and cough. Adverse events that were determined by the investigators to be related to the trial regimen occurred in 34 of 279 patients (12.2%) in the remdesivir group and in 25 of 283 (8.8%) in the

The New England Journal of Medicine is produced by NEJM Group, a division of the Massachusetts Medical Society.
Downloaded from nejm.org at Gilead Sciences on June 25, 2025. For personal use only.
No other uses without permission. Copyright © 2022 Massachusetts Medical Society. All rights reserved.

Table 2. Efficacy Calculated with the Use of a Cox Proportional-Hazards Model with Baseline Stratification Factors as Covariates.*

End Point	Remdesivir (N=279)	Placebo (N=283)	Hazard Ratio (95% CI)	P Value
Primary efficacy end point				
Covid-19–related hospitalization or death from any cause by day 28 — no. (%)†	2 (0.7)	15 (5.3)	0.13 (0.03 to 0.59)	0.008
Secondary efficacy end points				
Covid-19–related hospitalization or death from any cause by day 14 — no. (%)	2 (0.7)	15 (5.3)	0.13 (0.03 to 0.59)	
Covid-19–related medically attended visit or death from any cause — no./total no. (%)‡				
Day 14	2/246 (0.8)	20/252 (7.9)	0.10 (0.02 to 0.43)	
Day 28	4/246 (1.6)	21/252 (8.3)	0.19 (0.07 to 0.56)	
Death from any cause by day 28 — no.	0	0	NC	
Hospitalization for any cause by day 28 — no. (%)§	5 (1.8)	18 (6.4)	0.28 (0.10 to 0.75)	
Time-weighted average change in nasopharyngeal SARS-CoV-2 viral load from baseline to day 7 — $\log_{10}$ copies/ml	−1.24	−1.14	0.07 (−0.10 to 0.24)¶	
Alleviated baseline Covid-19 symptoms, according to FLU-PRO Plus questionnaire — no./total no. (%)‖				
Questionnaire completed before infusion on day 1	23/66 (34.8)	15/60 (25.0)	1.41 (0.73 to 2.69)**	
Questionnaire completed on day 1, either before or after infusion — no./total no. (%)§	61/169 (36.1)	33/165 (20.0)	1.92 (1.26 to 2.94)**	

* Baseline stratification factors were residence in a skilled nursing facility (yes or no), age (<60 years or ≥60 years), and country (United States or outside the United States). Covid-19 denotes coronavirus disease 2019, and NC not calculated.

† Of the eight patients who were adolescents, none had a Covid-19–related hospitalization or death from any cause by day 28.

‡ Data are shown for patients who underwent randomization, received at least one infusion of remdesivir or placebo, and met eligibility criteria as defined in protocol amendment 2 or later.

§ The analysis was conducted post hoc.

¶ The value is the least-squares mean.

‖ On the FLU-PRO (Influenza Patient-Reported Outcome) Plus questionnaire, which was adapted for patients with Covid-19, alleviation of Covid-19 symptoms was defined as mild or absent symptoms.

** The value is the rate ratio.

placebo group. Fewer patients in the remdesivir group than in the placebo group had serious adverse events (5 of 279 patients [1.8%] vs. 19 of 283 patients [6.7%]).

By day 28, laboratory abnormalities of grade 3 or higher had occurred in 29 of 279 patients (10.4%) in the remdesivir group and in 23 of 283 (8.1%) in the placebo group. At day 14, the mean (±SD) change from baseline in creatinine clearance was minimal (0.26±21.2 ml per minute in the remdesivir group and 1.9±18.6 ml per minute in the placebo group). Similarly, at day 14, the mean change from baseline in alanine aminotransferase levels was minimal (−3.0±21.6 U per liter in the remdesivir group and −1.0±27.4 U per liter in the placebo group).

DISCUSSION

In this trial involving nonhospitalized patients with Covid-19 who were at high risk for severe disease, we found that patients who received a 3-day course of remdesivir had an 87% lower risk of Covid-19–related hospitalization or death from any cause by day 28 and an 81% lower risk of Covid-19–related medically attended visits or death from any cause by day 28 than patients who received placebo. Results of subgroup analyses regarding the primary and secondary end points according to diabetes status, obesity, male sex, hypertension, and non–Hispanic or Latinx ethnic group were similar to those in the overall trial population and showed that in these sub-

The New England Journal of Medicine is produced by NEJM Group, a division of the Massachusetts Medical Society.
Downloaded from nejm.org at Gilead Sciences on June 25, 2025. For personal use only.
No other uses without permission. Copyright © 2022 Massachusetts Medical Society. All rights reserved.

A Covid-19–Related Hospitalization or Death from Any Cause

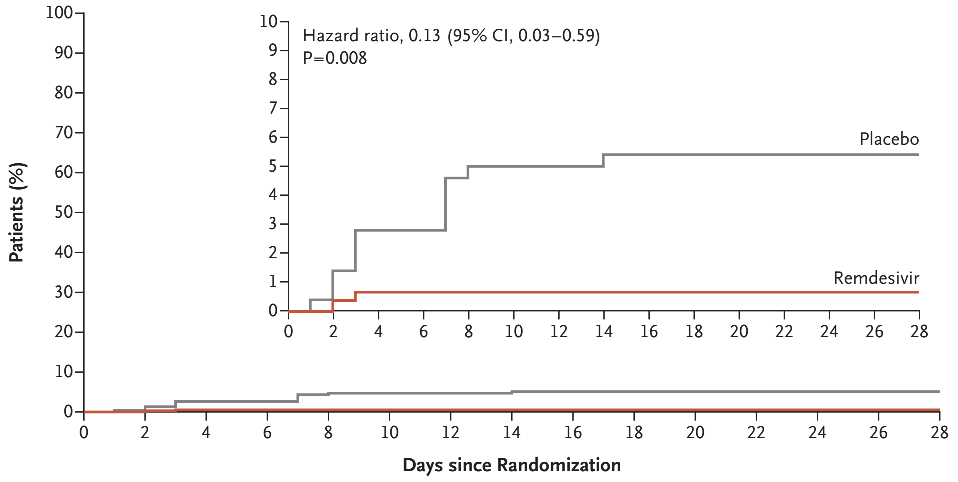

No. at Risk															
Placebo	283	280	272	271	265	264	264	263	262	261	261	260	256	250	227
Remdesivir	279	276	272	272	271	268	268	268	264	264	264	264	260	252	226

B Covid-19–Related Medically Attended Visit or Death from Any Cause

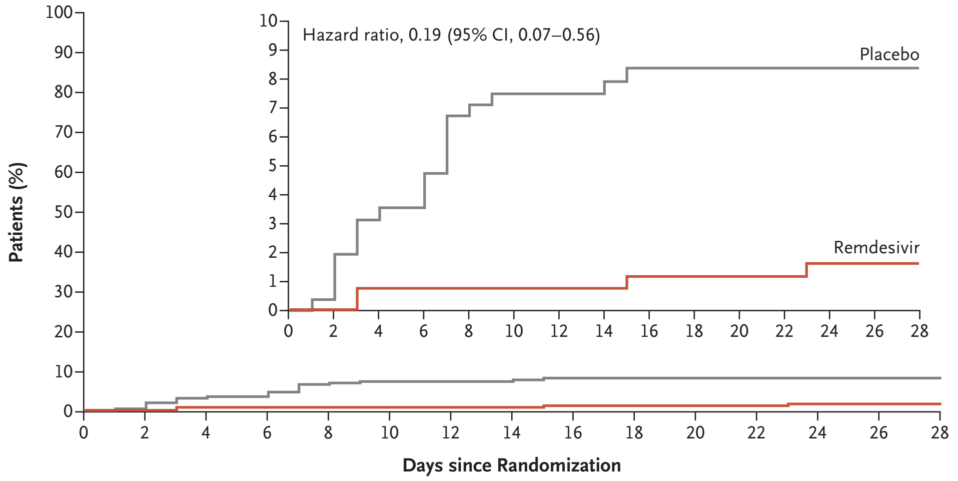

No. at Risk															
Placebo	252	249	241	239	230	228	228	227	225	224	224	223	219	213	193
Remdesivir	246	243	239	239	239	237	237	237	232	232	232	232	227	220	197

Figure 1. Primary Efficacy and Secondary End Points.

Panel A shows the Kaplan–Meier estimate of the time to hospitalization related to coronavirus disease 2019 (Covid-19) or death from any cause by day 28 (the primary efficacy end point). Panel B shows the Kaplan–Meier estimate of the time to a Covid-19–related medically attended visit or death from any cause by day 28 (a secondary end point); this end point was assessed in the modified full analysis set, which is defined in the statistical analysis plan (available with the protocol at NEJM.org). The hazard ratios, two-sided 95% confidence intervals, and P value were estimated with the use of Cox regression with the baseline stratification factors as covariates: residence in a skilled nursing facility (yes or no), age (<60 years or ≥60 years), and country (United States or outside the United States). Insets show the same data on an enlarged y axis.

groups, remdesivir resulted in a lower risk of Covid-19–related hospitalization or death from any cause by day 28 than placebo (Fig. 2). All instances of Covid-19–related hospitalization among patients with cancer, chronic lung disease, cardiovascular disease, and Hispanic or Latinx ethnicity

The New England Journal of Medicine is produced by NEJM Group, a division of the Massachusetts Medical Society.
Downloaded from nejm.org at Gilead Sciences on June 25, 2025. For personal use only.
No other uses without permission. Copyright © 2022 Massachusetts Medical Society. All rights reserved.

Subgroup	Remdesivir	Placebo	Hazard Ratio (95% CI)
	no./total no. of patients (%)		
Residence in the United States	2/264 (0.8)	12/267 (4.5)	0.17 (0.04–0.76)
Age ≥60 yr	1/83 (1.2)	9/87 (10.3)	0.11 (0.01–0.86)
Male sex	1/148 (0.7)	9/145 (6.2)	0.11 (0.01–0.84)
Diabetes mellitus	2/173 (1.2)	14/173 (8.1)	0.14 (0.03–0.63)
Obesity	1/154 (0.6)	9/156 (5.8)	0.11 (0.01–0.88)
Hypertension	2/138 (1.4)	10/130 (7.7)	0.17 (0.04–0.76)
Ethnic group			
Not Hispanic or Latinx	2/146 (1.4)	8/158 (5.1)	0.26 (0.06–1.22)
Hispanic or Latinx	0/123	6/112 (5.4)	—
Chronic lung disease	0/67	4/68 (5.9)	—
Cardiovascular or cerebrovascular disease	0/20	2/24 (8.3)	—
Current cancer	0/12	2/18 (11.1)	—

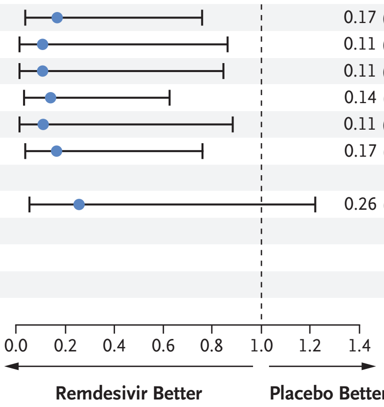

Figure 2. Covid-19–Related Hospitalization or Death from Any Cause at Day 28 in More Than 5% of the Trial Population, According to Demographic and Clinical Characteristics at Baseline.

Hazard ratios and two-sided 95% confidence intervals (I bars) were estimated with the use of Cox regression with the baseline stratification factors as covariates: residence in a skilled nursing facility (yes or no), age (<60 years or ≥60 years), and country (United States or outside the United States). Analyses with stratification according to Hispanic or Latinx ethnic group were conducted post hoc. Hazard ratios and 95% confidence intervals for subgroups with no events in the remdesivir group could not be accurately calculated and are therefore omitted. The dashed line indicates a hazard ratio of 1.0.

occurred in the placebo group. Results of a post hoc analysis involving patients who completed the FLU-PRO Plus questionnaire on day 1 either before or after the infusion showed that patients in the remdesivir group had a faster time to alleviation of baseline symptoms by day 14 than those in the placebo group. Treatment with remdesivir had an acceptable safety profile in a variety of outpatient settings, and the incidence of adverse events was similar to that in the placebo group.

The results of this trial should be considered in the context of other randomized trials of remdesivir. The first stage of the Adaptive Covid-19 Treatment Trial (ACTT-1) showed that patients with moderate-to-severe Covid-19 who were treated with remdesivir had a shorter time to recovery and a lower risk of progression to more severe respiratory disease than patients who received placebo.[6] One of the SIMPLE trials (GS-US-540-5774) showed that patients with moderate Covid-19 who received remdesivir for up to 5 days had significantly higher odds of having a better clinical status on day 11.[16] The ACTT-1 and SIMPLE trials also showed that the safety profile of remdesivir was similar to that of placebo, a finding consistent with the results of this trial.[6,7,16] Although some open-label, randomized trials involving hospitalized patients showed conflicting results with respect to clinical efficacy,[17,18] data from the ACTT-1 and SIMPLE trials and real-world studies,[19] together with the results of the current trial, support the clinical benefit of remdesivir across the Covid-19 spectrum. In addition, these data show that remdesivir may have greater efficacy when initiated earlier in the course of Covid-19, a finding consistent with post hoc analyses of data from the ACTT-1 trial that showed that remdesivir prevented disease progression.[20]

Despite its beneficial clinical effects, the upper airway viral load was not lower in patients who received remdesivir than in those who received placebo, as measured by nasopharyngeal RT-PCR testing. Similar discordant findings between clinical effectiveness and viral loads in the upper respiratory tract were reported in rhesus macaques that were infected with SARS-CoV-2.[21] In that study, remdesivir resulted in a clinical benefit and reduced SARS-CoV-2 replication in the lower respiratory tract compartment but did not significantly reduce viral load in the upper respiratory tract. These data support the hypothesis that SARS-CoV-2 nasopharyngeal vi-

The New England Journal of Medicine is produced by NEJM Group, a division of the Massachusetts Medical Society.
Downloaded from nejm.org at Gilead Sciences on June 25, 2025. For personal use only.
No other uses without permission. Copyright © 2022 Massachusetts Medical Society. All rights reserved.

ral loads do not reliably predict treatment outcomes in Covid-19.[22]

Remdesivir is another important option for outpatients with Covid-19. The efficacy of a 3-day course of remdesivir for nonhospitalized patients with Covid-19 is qualitatively similar to that of single-dose neutralizing monoclonal antibody therapies.[23-26] Like most neutralizing monoclonal antibody treatments, remdesivir is administered intravenously; however, it is a direct-acting antiviral agent that can be shipped and stored lyophilized at room temperature and does not require prolonged infusion times or post-infusion safety monitoring. Several countries lack access to monoclonal antibodies, and even in the United States, where monoclonal antibodies are authorized, shortages have transiently limited patient access. In addition, combination therapies that include a direct-acting antiviral agent at an early stage of disease may be synergistic in patients at highest risk (e.g., those who are immunocompromised). Given that remdesivir targets the highly conserved viral RNA-dependent RNA polymerase, it is likely to maintain efficacy against emerging SARS-CoV-2 variants of concern, a potential limitation of neutralizing monoclonal antibody therapy.[27] In vitro testing has shown that all variants of concern remain susceptible to remdesivir.[28] Finally, recent in vitro and in vivo data from the testing of an orally bioavailable prodrug of the parent nucleoside of remdesivir showed antiviral activity in lung cell lines and significantly lower viral load in the lungs of mice infected with SARS-CoV-2 that received this prodrug than in control mice.[29] Beyond supporting the use of readily available intravenous formulations, this trial provides a proof of concept for the study of new prodrugs of the active metabolite of remdesivir and provides support for the development of remdesivir for nonhospitalized patients with Covid-19 who are at risk for disease progression.

Our trial has several limitations. Although characteristics such as older age, certain high-risk coexisting conditions, and Hispanic or Latinx or American Indian or Alaska Native ethnic group were strongly represented in the trial population, Black or Asian race, chronic liver disease, chronic kidney disease, immunocompromised status, and cancer were underrepresented (Table 1 and Table S2). This trial was conducted primarily in the United States (94.5% of patients lived in the United States), and only 8 patients (1.4%) were adolescents. Like other trials, this trial excluded patients who had received SARS-CoV-2 vaccines; thus, it is difficult to generalize these data to the vaccinated population. However, for patients in regions of the world that do not yet have access to vaccines or for patients who do not have a good response to vaccination (e.g., those who are immunocompromised), outpatient remdesivir may play an important role in the management of Covid-19. This trial was also conducted before the emergence of the B.1.617.2 (delta) variant of SARS-CoV-2 as the dominant circulating strain. Finally, the trial was stopped for administrative reasons, and less than half of the planned enrollment was achieved. Nonetheless, we observed significantly better clinical out-

Table 3. Adverse Events.*

Event	Remdesivir (N=279)	Placebo (N=283)
	no. of patients (%)	
Primary safety end point: any adverse event	118 (42.3)	131 (46.3)
Adverse events		
Nausea	30 (10.8)	21 (7.4)
Headache	16 (5.7)	17 (6.0)
Cough	10 (3.6)	18 (6.4)
Diarrhea	11 (3.9)	11 (3.9)
Dyspnea	7 (2.5)	15 (5.3)
Fatigue	10 (3.6)	11 (3.9)
Ageusia	8 (2.9)	7 (2.5)
Anosmia	9 (3.2)	6 (2.1)
Dizziness	5 (1.8)	10 (3.5)
Chills	6 (2.2)	8 (2.8)
Pyrexia	1 (0.4)	11 (3.9)
Covid-19 pneumonia	2 (0.7)	8 (2.8)
Adverse event related to trial regimen	34 (12.2)	25 (8.8)
Serious adverse event†	5 (1.8)	19 (6.7)
Adverse event leading to discontinuation of trial regimen	2 (0.7)	5 (1.8)
Death	0	0

* Of the eight patients who were adolescents, one patient in the placebo group reported an adverse event (mild fatigue).
† Severity grades were defined according to the Division of AIDS Table for Grading the Severity of Adult and Pediatric Adverse Events, version 2.1.

The New England Journal of Medicine is produced by NEJM Group, a division of the Massachusetts Medical Society.
Downloaded from nejm.org at Gilead Sciences on June 25, 2025. For personal use only.
No other uses without permission. Copyright © 2022 Massachusetts Medical Society. All rights reserved.

comes among patients who received remdesivir than among those who received placebo. The discontinuation of the trial because of administrative reasons was unlikely to have introduced bias because no interim statistical analyses were performed, and double blinding was maintained until the data were finalized.

A 3-day course of remdesivir had an acceptable safety profile and prevented disease progression resulting in hospitalization among high-risk patients with Covid-19. In the campaign toward ending the Covid-19 pandemic, these data add yet another option to the armamentarium for the treatment of vulnerable patients who are at high risk for progression to severe Covid-19.

Supported by Gilead Sciences.

Disclosure forms provided by the authors are available with the full text of this article at NEJM.org.

A data sharing statement provided by the authors is available with the full text of this article at NEJM.org.

This article is dedicated to the memory of Dr. Marty, whose rigor and passion for science and his dedicated practice in the fields of transplant infectious diseases and Covid-19 were an inspiration to us all. We thank the patients who participated in this trial, their families, the support staff, and Sandra Chen (Gilead Sciences) for writing assistance.

APPENDIX

The authors' full names and academic degrees are as follows: Robert L. Gottlieb, M.D., Ph.D., Carlos E. Vaca, M.D., Roger Paredes, M.D., Ph.D., Jorge Mera, M.D., Brandon J. Webb, M.D., Gilberto Perez, M.D., Godson Oguchi, M.D., Pablo Ryan, M.D., Ph.D., Bibi U. Nielsen, M.D., Michael Brown, Ph.D., F.R.C.P., Ausberto Hidalgo, M.D., Yessica Sachdeva, M.D., Shilpi Mittal, M.D., Olayemi Osiyemi, M.D., Jacek Skarbinski, M.D., Kavita Juneja, M.D., Robert H. Hyland, D.Phil., Anu Osinusi, M.D., Shuguang Chen, Ph.D., Gregory Camus, Ph.D., Mazin Abdelghany, M.D., Santosh Davies, M.D., Nicole Behenna-Renton, Frank Duff, M.D., Francisco M. Marty, M.D., Morgan J. Katz, M.D., Adit A. Ginde, M.D., M.P.H., Samuel M. Brown, M.D., Joshua T. Schiffer, M.D., and Joshua A. Hill, M.D.

The authors' affiliations are as follows: Baylor University Medical Center and Baylor Scott and White Research Institute, Dallas (R.L.G.), and Care United Research, Forney (S.M.) — all in Texas; the Nuren Medical and Research Center, Miami (C.E.V.), Evolution Clinical Trials, Hialeah Gardens (G.P.), the Midland Florida Clinical Research Center, DeLand (G.O.), Luminous Clinical Research–South Florida Urgent Care, Pembroke Pines (A.H.), and Triple O Research Institute Professional Association, West Palm Beach (O.O.) — all in Florida; Hospital Universitari Germans Trias i Pujol and IrsiCaixa AIDS Research Institute, Barcelona (R.P.), and Hospital Universitario Infanta Leonor and Gregorio Marañón Health Research Institute, Madrid (P.R.) — all in Spain; the Cherokee Nation Outpatient Health Center, Tahlequah, OK (J.M.); Intermountain Healthcare (B.J.W., S.M.B.) and the University of Utah School of Medicine (S.M.B.) — both in Murray; Copenhagen University Hospital–Rigshospitalet, Copenhagen (B.U.N.); University College London Hospitals NHS Foundation Trust and the London School of Hygiene and Tropical Medicine — both in London (M.B.); the Institute of Liver Health, Mesa, AZ (Y.S.); Kaiser Permanente, Oakland (J.S.), and Gilead Sciences, Foster City (K.J., R.H.H., A.O., S.C., G.C., M.A., S.D., N.B.-R., F.D.) — both in California; Brigham and Women's Hospital and Harvard Medical School — both in Boston (F.M.M.); Johns Hopkins University School of Medicine, Baltimore (M.J.K.); the University of Colorado School of Medicine, Aurora (A.A.G.); and the Fred Hutchinson Cancer Research Center and the University of Washington School of Medicine — both in Seattle (J.T.S., J.A.H.).

REFERENCES

1. WHO Director-General's opening remarks at the media briefing on COVID-19. Geneva: World Health Organization, March 11, 2020 (https://www.who.int/director-general/speeches/detail/who-director-general-s-opening-remarks-at-the-media-briefing-on-covid-19---11-march-2020).
2. Gallo Marin B, Aghagoli G, Lavine K, et al. Predictors of COVID-19 severity: a literature review. Rev Med Virol 2021;31:1-10.
3. Stokes EK, Zambrano LD, Anderson KN, et al. Coronavirus disease 2019 case surveillance — United States, January 22–May 30, 2020. MMWR Morb Mortal Wkly Rep 2020;69:759-65.
4. Pizzorno A, Padey B, Julien T, et al. Characterization and treatment of SARS-CoV-2 in nasal and bronchial human airway epithelia. Cell Rep Med 2020;1(4):100059.
5. Sheahan TP, Sims AC, Graham RL, et al. Broad-spectrum antiviral GS-5734 inhibits both epidemic and zoonotic coronaviruses. Sci Transl Med 2017;9:eaal3653.
6. Beigel JH, Tomashek KM, Dodd LE, et al. Remdesivir for the treatment of Covid-19 — final report. N Engl J Med 2020;383:1813-26.
7. Goldman JD, Lye DCB, Hui DS, et al. Remdesivir for 5 or 10 days in patients with severe Covid-19. N Engl J Med 2020;383:1827-37.
8. Mulangu S, Dodd LE, Davey RT Jr, et al. A randomized, controlled trial of Ebola virus disease therapeutics. N Engl J Med 2019;381:2293-303.
9. Muthuri SG, Venkatesan S, Myles PR, et al. Effectiveness of neuraminidase inhibitors in reducing mortality in patients admitted to hospital with influenza A H1N1pdm09 virus infection: a meta-analysis of individual participant data. Lancet Respir Med 2014;2:395-404.
10. Nicholson KG, Aoki FY, Osterhaus AD, et al. Efficacy and safety of oseltamivir in treatment of acute influenza: a randomised controlled trial. Lancet 2000;355:1845-50.
11. The INSIGHT START Study Group. Initiation of antiretroviral therapy in early asymptomatic HIV infection. N Engl J Med 2015;373:795-807.
12. Siddiqi HK, Mehra MR. COVID-19 illness in native and immunosuppressed states: a clinical-therapeutic staging proposal. J Heart Lung Transplant 2020;39:405-7.
13. Huang C, Wang Y, Li X, et al. Clinical features of patients infected with 2019 novel coronavirus in Wuhan, China. Lancet 2020;395:497-506.
14. Wang D, Hu B, Hu C, et al. Clinical characteristics of 138 hospitalized patients with 2019 novel coronavirus-infected pneumonia in Wuhan, China. JAMA 2020;323:1061-9.
15. Cevik M, Kuppalli K, Kindrachuk J, Peiris M. Virology, transmission, and pathogenesis of SARS-CoV-2. BMJ 2020;371:m3862.
16. Spinner CD, Gottlieb RL, Criner GJ, et al. Effect of remdesivir vs standard care on clinical status at 11 days in patients

The New England Journal of Medicine is produced by NEJM Group, a division of the Massachusetts Medical Society.
Downloaded from nejm.org at Gilead Sciences on June 25, 2025. For personal use only.
No other uses without permission. Copyright © 2022 Massachusetts Medical Society. All rights reserved.

with moderate COVID-19: a randomized clinical trial. JAMA 2020;324:1048-57.
17. Ader F, Bouscambert-Duchamp M, Hites M, et al. Remdesivir plus standard of care versus standard of care alone for the treatment of patients admitted to hospital with COVID-19 (DisCoVeRy): a phase 3, randomised, controlled, open-label trial. Lancet Infect Dis 2021 September 14 (Epub ahead of print).
18. The WHO Solidarity Trial Consortium. Repurposed antiviral drugs for Covid-19 — interim WHO Solidarity trial results. N Engl J Med 2021;384:497-511.
19. Mozaffari E, Chandak A, Zhang Z, et al. Remdesivir treatment in hospitalized patients with COVID-19: a comparative analysis of in-hospital all-cause mortality in a large multi-center observational cohort. Clin Infect Dis 2021 October 1 (Epub ahead of print).
20. Fintzi J, Bonnett T, Sweeney DA, et al. Deconstructing the treatment effect of remdesivir in the Adaptive COVID-19 Treatment Trial-1: implications for critical care resource utilization. Clin Infect Dis 2021 August 19 (Epub ahead of print).
21. Williamson BN, Feldmann F, Schwarz B, et al. Clinical benefit of remdesivir in rhesus macaques infected with SARS-CoV-2. Nature 2020;585:273-6.
22. COVID-19: developing drugs and biological products for treatment or prevention. Guidance for industry. Silver Spring, MD: Food and Drug Administration, February 2021 (https://www.fda.gov/media/137926/download).
23. Dougan M, Nirula A, Azizad M, et al. Bamlanivimab plus etesevimab in mild or moderate Covid-19. N Engl J Med 2021;385:1382-92.
24. Gottlieb RL, Nirula A, Chen P, et al. Effect of bamlanivimab as monotherapy or in combination with etesevimab on viral load in patients with mild to moderate COVID-19: a randomized clinical trial. JAMA 2021;325:632-44.
25. Gupta A, Gonzalez-Rojas Y, Juarez E, et al. Early treatment for Covid-19 with SARS-CoV-2 neutralizing antibody sotrovimab. N Engl J Med 2021;385:1941-50.
26. Weinreich DM, Sivapalasingam S, Norton T, et al. REGN-COV2, a neutralizing antibody cocktail, in outpatients with Covid-19. N Engl J Med 2021;384:238-51.
27. Chen RE, Winkler ES, Case JB, et al. In vivo monoclonal antibody efficacy against SARS-CoV-2 variant strains. Nature 2021;596:103-8.
28. Pitts JD, Lu X, Du Pont V, et al. Remdesivir retains potent antiviral activity against the SARS-CoV-2 delta variant and other variants of concern. Presented at the International Society for Influenza and Other Respiratory Viruses (ISIRV), ISIRV–WHO Conference on COVID-19, Influenza and RSV: Surveillance-Informed Prevention and Treatment virtual conference, October 19–21, 2021. poster.
29. Schäfer A, Martinez DR, Won JJ, et al. Therapeutic efficacy of an oral nucleoside analog of remdesivir against SARS-CoV-2 pathogenesis in mice. September 17, 2021 (https://www.biorxiv.org/content/10.1101/2021.09.13.460111v3). preprint.
Copyright © 2021 Massachusetts Medical Society.

ARTICLE METRICS NOW AVAILABLE
Visit the article page at NEJM.org and click on Metrics to view comprehensive and cumulative article metrics compiled from multiple sources, including Altmetrics. NEJM.org/about-nejm/article-metrics.

The New England Journal of Medicine is produced by NEJM Group, a division of the Massachusetts Medical Society.
Downloaded from nejm.org at Gilead Sciences on June 25, 2025. For personal use only.
No other uses without permission. Copyright © 2022 Massachusetts Medical Society. All rights reserved.
