# Supplementary material for: Bayesian reanalysis of early remdesivir for the treatment of COVID-19 in outpatients with high risk of progression to severe disease
Source: PLoS One. 2026 Apr 16;21(4):e0346878. doi: 10.1371/journal.pone.0346878 (PMC13086435; doi:10.1371/journal.pone.0346878)
Supplement: S5 Appendix — The appendix accompanying The New England Journal of Medicine publication of the PINETREE trial titled “Early Remdesivir to Prevent Progression to Severe Covid-19 in Outpatients” (reference 9). (PDF) [file pone.0346878.s006.pdf]

Supplementary Appendix

Supplement to: Gottlieb RL, Vaca CE, Paredes R, et al. Early remdesivir to prevent progression to severe Covid-19 in outpatients. N Engl J Med 2022;386:305-15. DOI: 10.1056/NEJMoa2116846

This appendix has been provided by the authors to give readers additional information about the work.

Supplementary Appendix to: **Early Outpatient Remdesivir to Prevent Progression to Severe Covid-19**

Table of Contents:

GS-US-540-9012 Study Investigators by Country 2

GS-US-540-9012 Research and Support Team Members 6

Figure S1. Patient Disposition 9

Figure S2. Kaplan-Meier Estimate of Time to Symptom Alleviation as Reported by Covid-19-adapted FLU-PRO Questionnaire....................10

Figure S3. Mean (95% CI) Change from Baseline in Nasopharyngeal SARS-CoV-2 Viral Load by Visit Day....................12

Table S1. Patient demographics, hospitalization data, and outcomes over the course of available follow-up 13

Table S2. Representativeness of Study Participants 15

References....................16

GS-US-540-9012 Study investigators by country

Denmark

Principal Investigator	Site
Thomas Benfield	Amager Hvidovre Hospital<br>Hvidovre, Denmark
Jan Gerstoft	Copenhagen University Hospital - Rigshospitalet<br>Copenhagen, Denmark
Isik Somuncu Johansen	Odense University Hospital and University of Southern Denmark<br>Odense C, South Denmark
Henrik Nielsen	Aalborg Universitetshospital<br>Aalborg, Denmark
Nina Stærke	Aarhus Universitetshospital<br>Aarhus, Denmark

Spain

Principal Investigator	Site
Roger Paredes Deiros	Hospital Universitari Germans Trias i Pujol<br>Barcelona, Spain
Pablo Ryan Murua	Hospital Universitario Infanta Leonor<br>Madrid, Spain

United Kingdom

Principal Investigator	Site
Michael Brown	University College London Hospitals<br>London, United Kingdom
Dinesh Saralaya	Patient Recruitment Centres (PRCs) - Bradford<br>Bradford, United Kingdom

United States

Principal Investigator	Site
Matthew Abinante	Elevated Health<br>Huntington Beach, California
Igor Abolnik	St. Joseph Heritage Healthcare<br>Eureka, CA
Atoya Adams	AB Clinical Trials - Hunt - PPDS<br>Las Vegas, Nevada
Paul Benson	Be Well Medical Center<br>Berkeley, Michigan
Daniel Berger	Northstar Medical Center<br>Chicago, Illinois

United States (continued)

Principal Investigator	Site
Cynthia Brinson	Central Texas Clinical Research<br>Austin, Texas
John Cafardi	The Christ Hospital<br>Cincinnati, Ohio
Vasundhara Cheekati	Agile Clinical Research Trials, LLC<br>Atlanta, Georgia
Paul Cook	East Carolina University<br>Greenville, North Carolina
Gordon Crofoot	The Crofoot Research Facility<br>Houston, Texas
Frederick Cruickshank	Rosedale Infectious Diseases<br>Huntersville, North Carolina
Marcia Epstein	Northwell Health<br>Manhasset, New York
Augusto Focil	FOMAT Medical Research - FOMAT - HyperCore - PPDS<br>Oxnard, California
Brad Frandsen	Sound Medical Research<br>Port Orchard, Washington
Bernard Garcia	Invesclinic, LLC<br>Fort Lauderdale, Florida
Juan Garza	VIP Trials<br>San Antonio, Texas
Erika Gonzalez	STAAMP Research, LLC - CRN - PPDS<br>San Antonio, Texas
Linda Gorgos	AXCES Research Group<br>Santa Fe, New Mexico
Robert L Gottlieb	Baylor University Medical Center<br>Dallas, Texas<br><br>Baylor Scott & White Medical Center – Hillcrest<br>Waco, Texas<br><br>Baylor Scott & White Medical Center – Irving<br>Irving, Texas
Enrique Hanabergh	L&C Professional Medical Research Institute<br>Miami, Florida
Ausberto Hidalgo	Luminous Clinical Research<br>Miami, Florida

United States (continued)

**Principal Investigator**	**Site**
Joshua Hill	Fred Hutchinson Cancer Research Center<br>Seattle, Washington
Rudolf Kotula	Quality Clinical Research - HyperCore - PPDS<br>Omaga, Nebraska
Kelly Lannutti	South Shore Hospital<br>South Weymouth, Massachusetts
Thomas Ledbetter	ClinPoint Trials LLC - ClinEdge - PPDS<br>Waxahachie, Texas
Ramon Leon	IMIC Inc.<br>Palmetto Bay, Florida
Michael Marsh	Med Partners, Inc. dba Premiere Medical<br>Toluca Lake, California
Henry Mendoza	Onyx Clinical Research<br>Flint, Michigan
Jorge Mera	Cherokee Nation<br>Talequah, Oklahoma
Anthony Mills	Mills Clinical Research at Men's Health Foundation<br>Los Angeles, California
Shilpi Mittal	Care United Research, LLC<br>Forney, Texas
Godson Oguchi	Midland Florida Clinical Research Center LLC<br>Deland, Florida
Olayemi Osiyemi	Triple O Research Institute PA<br>West Palm Beach, Florida
David Park	St. Joseph Heritage Healthcare<br>Fullerton, California
Jesse Penico	Memorial Hospital at Gulfport<br>Gulfport, Mississippi
Gilberto Perez	Evolution Clinical Research (Hialeah Gardens)<br>Hialeah Gardens, Florida
Rodolfo Perez	Encore Medical Research, LLC<br>Hollywood, Florida
Gerald Pierone, Jr.	AIDS Research and Treatment Center of the Treasure Coast<br>Vero Beach, Florida
Christopher Polk	Atrium Health<br>Charlotte, North Carolina
Moti Ramgopal	Midway Immunology and Research Center<br>Fort Pierce, Texas

United States (continued)

**Principal Investigator**	**Site**
Yessica Sachdeva	Arizona Liver Health<br>Mesa, Arizona
Arun Sanyal	Virginia Commonwealth University<br>Richmond, Virginia
Sorana Segal-Maurer	New York-Presbyterian-Queens<br>Flushing, New York
Nirav Shah	NorthShore University HealthSystem<br>Evanston, Illinois
Jacek Skarbinski	Kaiser Permanente Oakland Medical Center<br>Oakland, California
Kathryn Springer	Centura Health Research Center<br>Devner, Colorado
Chad Thury	Avera Research Institute<br>Sioux Falls, South Dakota
Bruce Torkan	LA Universal Research Center, Inc.<br>Los Angeles, California
Carlos Vaca	Nuren Medical and Research Center<br>Miami, Florida
Brandon Webb	Intermountain Healthcare<br>Murray, Utah

GS-US-540-9012 Research and Support Team Members

The study authors and Gilead Sciences express our enormous gratitude to the staff at each hospital who risked their lives to care for patients with Covid-19. This study would not have been possible without their heroic efforts. We regret that we are unable to list the support staff at every hospital which took part in this study.

DENMARK

Copenhagen University Hospital - Rigshospitalet, Copenhagen, Denmark

Jonathan Atilla Koefoed Starling, Bibi Uhre Nielsen, Anne Arentoft Bayer, Christian Leo Hansen, Jan Gerstoft, Dorthe Hass, Lene Pors Jensen,

SPAIN

Hospital Universitari Germans Trias i Pujol, Barcelona, Spain & irsiCaixa AIDS Research Institute

Lourdes Mateu, MD, PhD; Constanza Lucero, MD, PhD; Anna Masferrer, MD; Maria Jose Dominguez, MD; Pere Toran, MD, PhD; Noemí Lamonja, MD; Silvia Roure, MD, PhD; Laura Soldevila, MD; Adrian Siles, MD; David Figueroa, BSc; Anna Chamorro, BSc; Anna Martinez, BSc; Jordi Puig, BSc.

Hospital Universitario Infanta Leonor, Barcelona, Spain

Belen Escribano, Pablo Ryan Murua, Jose Luise Fraile Gonzalez, Maria Gonzalez del Pozuelo, Ismael Escobar, Victorino Diez Viñas, Juan Ignacio Lazo Alvarez, Jorge Antonio Valencia La Rosa, Elsa Izquierdo Garcia

UNITED KINGDOM

University College London Hospitals, London, United Kingdom

Ellie-Mae Young, Gabriella Bidwell, Diana Mabayoje, Tim Cutfileld, Mohammed Sakib Rokadiya, Naina McCann, Hugh Kingston, Deirdre Sally, Nina Bason, Sarah Logan, Novin Zahedi Fard, Richard Gilson, Chi Yee Chung, Pushpsen Joshi, Eyoanwan Simon-Modebe, Nicola Stewart, Odunola Akinyemi, Ewa Zatyka, Holly Baker, Anastasiya Deda, Pooja Patel, Robin Bailey, Zsofia Papp, Nayema Akther Tahmin, Michael Brown, Temi Olusi, Aileen Ni Chaoilte, Alessandro Borca, Abraham Roodt, Tracy-Lee Beukman, Marzia Fiorino, Gabriella Bidwell, Diana Mabayoje, Tim Cutfield, Mohammed Sakib Rokadiya, Deirdre Sally, Sarah Logan, Richard Gilson, Nicola Stewart, Ewa Zatyka, Robin Bailey, Aileen Ni Chaoilte, Alessandro Borca, Marzia Fiorino; James Burns, Veronica Barrett, Diana Phillips, Sneha Balhekar, Chi Yee Chung Nina Bason, Zsofia Papp, Temi Olusi, Novin Zahedi Fard, Odunola Akinyemi, Abraham Roodt, Tracy-Lee Beukman

UNITED STATES

Arizona Liver Health, Mesa, Arizona

Yessica Sachdeva, Kushal Doshi, Dina Gibson, Clarissa Zigelstein, Juanita Lingle, Sandy Billingsley, Anita Spring, Anita Kohli, Marissa Kopp, Carey Goldsmith, Kara Piper, Veronica Garcia, Jennifer Jones, Mario D'Gyves, , Jessica Bradley, Rajinder Kaur, Amanda McFarland, Deborah Gil Del Real, Sindy Gutierrez, Alisha Goyal, Dana Robledo, Kyle Bondy, Mary Goode, Kamilla Ojeda

Baylor University Medical Center, Dallas, Texas
Robert L Gottlieb MD PhD, John S. Garrett MD, Marcus Holmes DO, Mark Hupert MD, Cedric W. Spak MD, Samantha Adler, Eva Patel, Mostapha Balon RN, Amanda Doss, Rania El Tomi, Julianne Garnett, Shelby Shull RN, Sondra Skelly RN, Charity Smith, Adrian Palacios PharmD, Aaron Killian PharmD, Edilia Solis CPhT, Aayla Jamil, Joost Felius, Janet Jerrow, Leslie Jacob, Amy Barnette

Baylor Scott & White Medical Center – Hillcrest, Waco, Texas
Allison Crawford DO, Ronald Wilson, MD, Robert L Gottlieb MD PhD, Nancy Canada LVN, Maria Gorschboth, Amy Powers, Whitney "Taylor" Roznos, Pamela Zook LVN, Michele Richardson, Natalie Settele, Jessica Christian PharmD, Dianna Robinson LVN

Baylor Scott & White Medical Center – Irving, Irving, Texas
Steven G. Davis MD, Robert L. Gottlieb, MD PhD, Tania Purkayastha MD, Brittany Garcia, Jon Thammavong, Alyssa Boudreau, Johna Gilbert RN, Susan Ferdosian PharmD, Hemangini Joshi PharmD, Chad Samuelson PharmD, Keri Simon PharmD, Amy C. Thomas PharmD, Tammy Fisher

Care United Research, LLC, Forney, Texas
Shilpi Mittal, Patricia Church, Valerie Apodaca, Natalie Perini, Bilal Khan, Erin Clayton, Richard Slaughter, Nichole Alegria, Megan Followwell, Dimple Varughese, Sara Lembke, Anjelica Martinez, Katharine Daniel, Jennifer Jasso, Paula Smith

Cherokee Nation, Talequah, Oklahoma
Jorge Mera, Whitney Essex, Dwayne David, Stacey Thornton, Linzi Allen, Alena Korbut Clayton Myers, Kristen Adams, Emma Harp, Kristina Ramsey, Sabrina Brown, Elizabeth Odell, Ami Sams, Stephanie Hammons, Rebecca Albarran, Robin Rishinghawk, Kristin Walker, Stephanie Wisdom, and Carrie Garces.

Evolution Clinical Research (Hialeah Gardens), Hialeah Gardens, Florida
Gilberto Perez, Romel Figueredo, Michael Rones, Laura Fumero, Gianeya Mateu

Fred Hutchinson Cancer Research Center, Seattle, Washington
Joshua Hill, Rachel Blazevic, Jennifer Wright, Melissa Louthain, Jeffrey Gregory, Fatima Ranjbaran, Lauren Watson

Intermountain Healthcare, Murray, Utah
Brandon Webb, Sarah Kator, Anthony Putich, Erin Stahl, Alisa Thomas, Albert Kemp, Shannon Sell, Carlos Barbagelata, Jake Krong, David Tomer, Jenny Simmonds, Mark Crosby, Kaylene Russell, Elisa Montgomery

Kaiser Permanente Oakland Medical Center, Oakland, California

Jacek Skarbinski, Tashia Orr, Bernadette Aragon, Svetlana Siniy, Jewel Perry, Korenna Sinn, Kennette Yoshimura, Swain Bretton, Olivia Anaya, Ryan Pavlovich, Regina Linetskaya, Renee Cunanan, Jenny Yip, Ann Rose Downing, Isabel Perez, Pablo Sarmiento, Jade Ramos-Poblete, Kevin Won, Victoria Khomits-Koval, Kelly Sullivan, Wendy Evangelista, Susan Miranda, Kathy Cabassa, Susan Campbell, Cheyenne Lang, Sarah McLachlen, Hallie Seegal, Zara Fatima, Marina Rumore, Arin Urban, Somalee Banerjee, Brittany Ashlock, Monica Taylor, Alan Go, Linh Nguyen, Phenius Lathon, Angelique Tjen-A-Looi, Asaf Shor, Sriharsha Rao, Niti Mann, Lisa Liu, Sannaporn Sereenvinyayut, Jon Oide, Dana Sax, Dustin Mark, Jason Flamm, Charles Hare, Jonathon Volk, Paul Waldron, Meena Pai, Kevin Ernsting, Mayura Suryanarayan, Ingrid Stram-Doll, Gurpreet Rihal, Saroja Sripathi, Connie Park, Susan Jacobson, Rachna Bali, Mamata Kene, Calvin Kong, Matthew Eldridge, Andrew McNeil, Rodger Siemens, Dana Clutter, Jamila Champsi, George Minowada, Babak Kharazzi, Leila Ganjehei, Maziar Shirazi, Victoria Peckham, Shinh Sanal, Richard Joven, Bretton Swain, Maggie Turner, Victor Chen

Luminous Clinical Research, Miami, Florida
Ausberto Hidalgo, Jose Reinaldo Pinero, Lys Robles, Leighton Parkins, Carlos Batista

Midland Florida Clinical Research Center LLC, Deland, Florida
Godson Oguchi, Jack Dienes, Kahla Damianik, Shawn Massaro, Monique Mathis, Mauver Skipper, Jaime Norales, Mary Michelle Arsenault, Judepatrick Onyema, Ryan Thompson, Stacey-Ann Villaruel

Nuren Medical and Research Center Miami, Florida
Carlos Vaca, Milagros Nava ,Ivan Canas, Patricia Flores, Yudemis Lau Barredo, Ivan Banguela, Humberto Hernandez

Triple O Research Institute PA, West Palm Beach, Florida
Olayemi Osiyemi, Alexandra Vargas, Ayana Campos, Paulesha Isaacs, Stephanie Sirof, Myriam Izquierdo, Nadine Davis, Christina Campbell, Jose Menajovsky-Chaves, Stephanie Martinez, Odelsy Torna, Michelle McDow, Praewpailin Rich

University of Colorado – PPDS, Aurora, Colorado
Adit Ginde

Figure S1. Patient Disposition

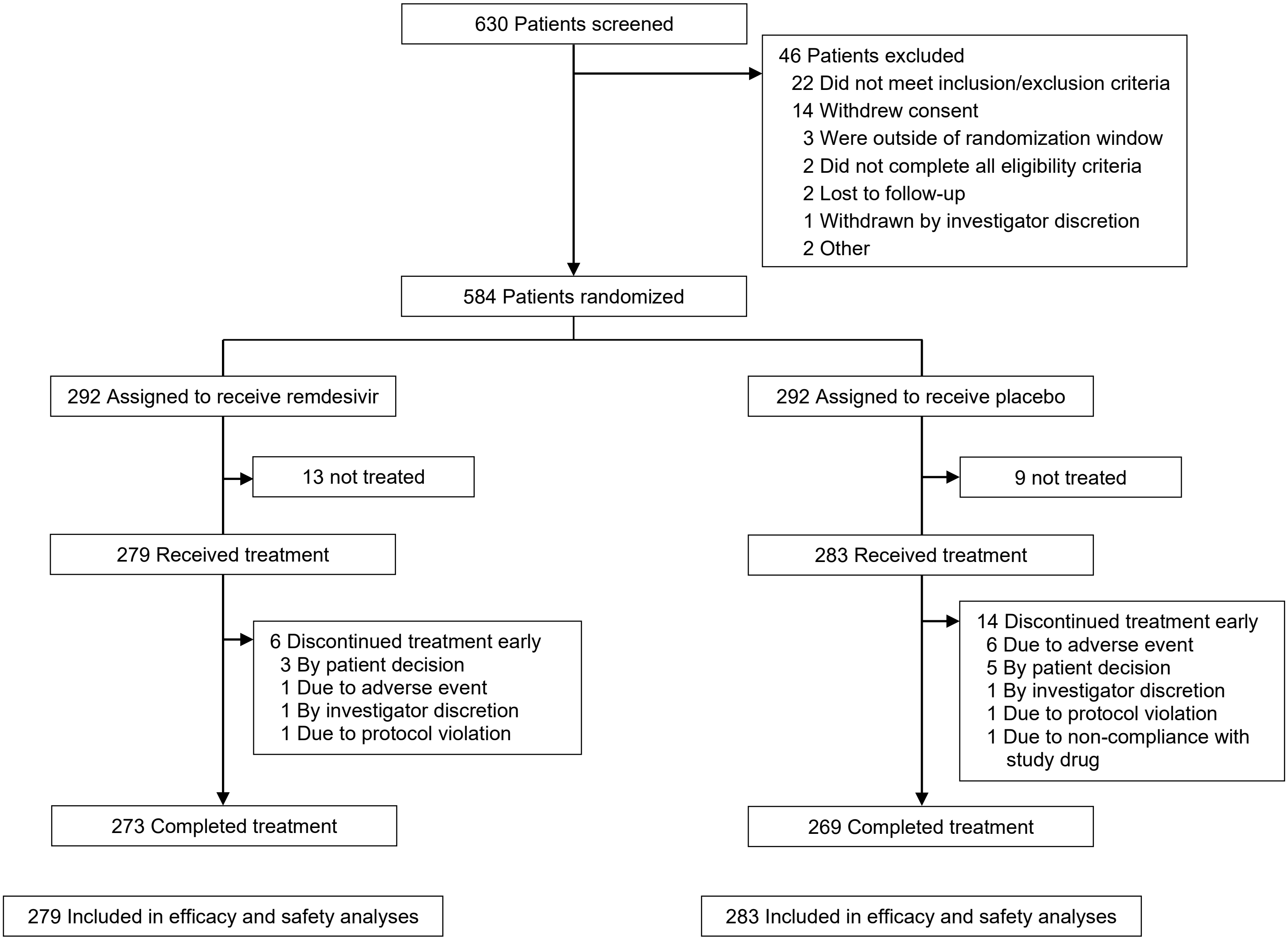

Figure S2. Kaplan-Meier Estimate of Time to Symptom Alleviation as Reported by Covid-19-adapted FLU-PRO Questionnaire

A. Per protocol analysis, including patients who completed the baseline questionnaire before the first treatment dose

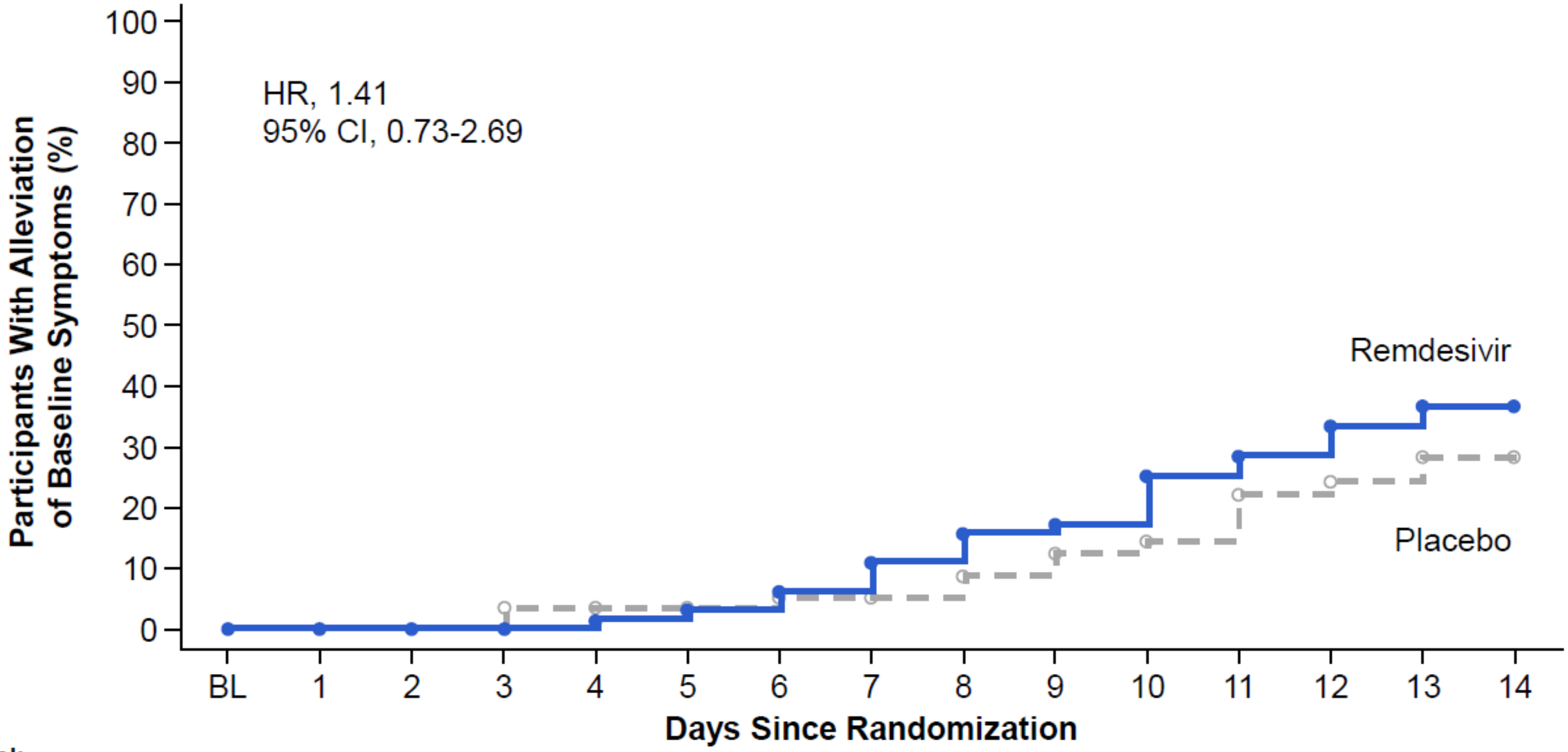

No. at risk

Remdesivir IV for 3 days	66	66	66	66	64	63	62	60	57	53	52	46	44	41	36
Placebo	60	60	60	59	57	57	56	55	52	49	47	44	38	37	32

Hazard Ratio and two-sided 95% CI were estimated using the Cox regression with baseline stratification factors (residence in a skilled nursing facility yes vs no, age <60 vs ≥60 years, and US vs outside US) as covariates.
N represents the number of participants at risk at the beginning of the interval.
BL, baseline; HR, hazard ratio.

B. Post-hoc analysis, including patients who completed the baseline questionnaire prior to or on the same day as the first treatment dose

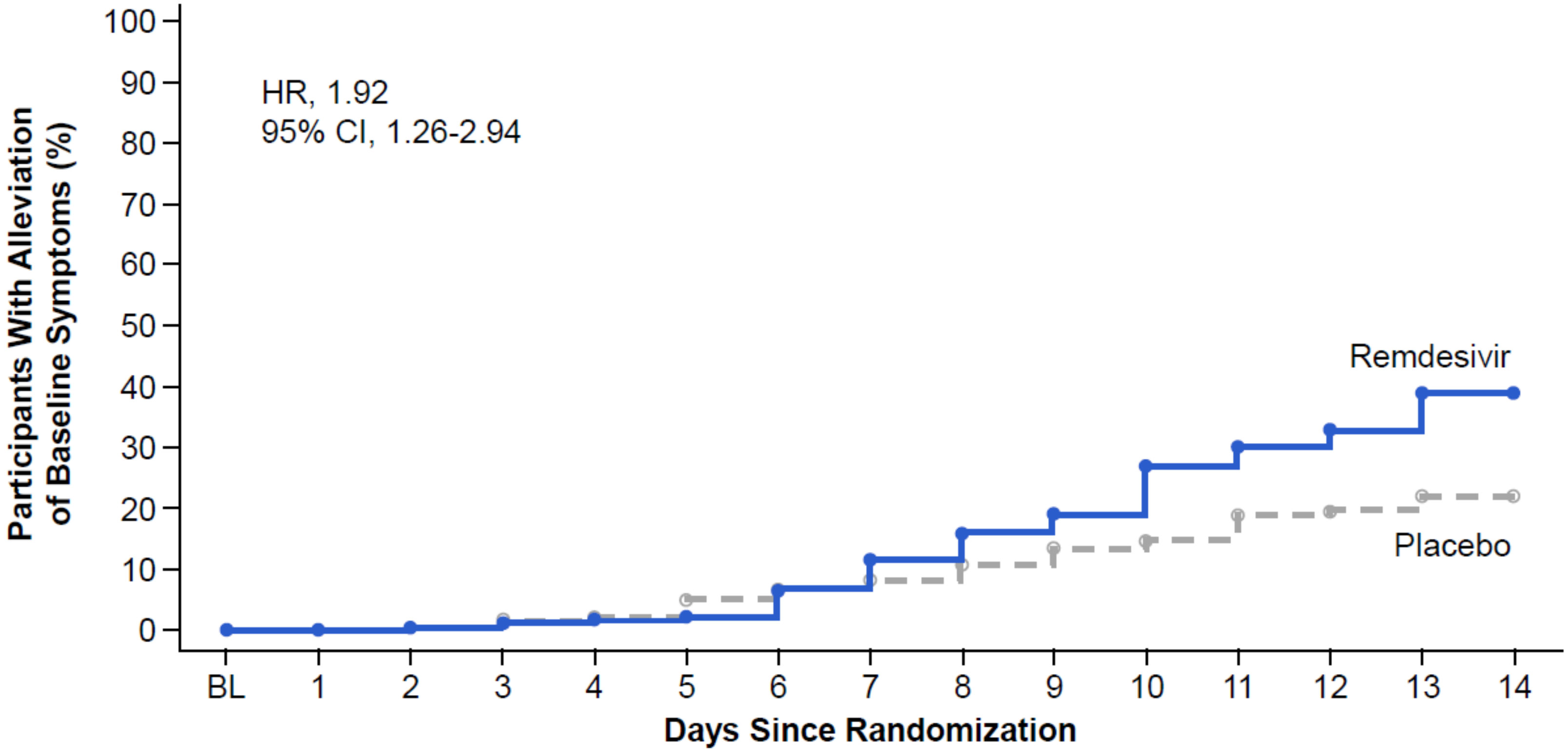

No. at risk															
Remdesivir IV for 3 days	169	169	169	167	162	161	159	152	141	133	125	112	106	99	80
Placebo	165	165	165	162	156	154	149	145	138	132	127	123	112	109	93

Participants who had baseline symptoms scored as 1 or higher and did not have alleviation were censored at the last assessment day.
Hazard ratio and two-sided 95% CI were estimated using the Cox regression with baseline stratification factors (residence in a skilled nursing facility yes vs no, age <60 vs ≥60 years, and US vs outside US) as covariates.
N represents the number of participants at risk at the beginning of the interval.
BL, baseline; HR, hazard ratio.

Figure S3. Mean (95% CI) Change from Baseline in Nasopharyngeal SARS-CoV-2 Viral Load by Visit Day.

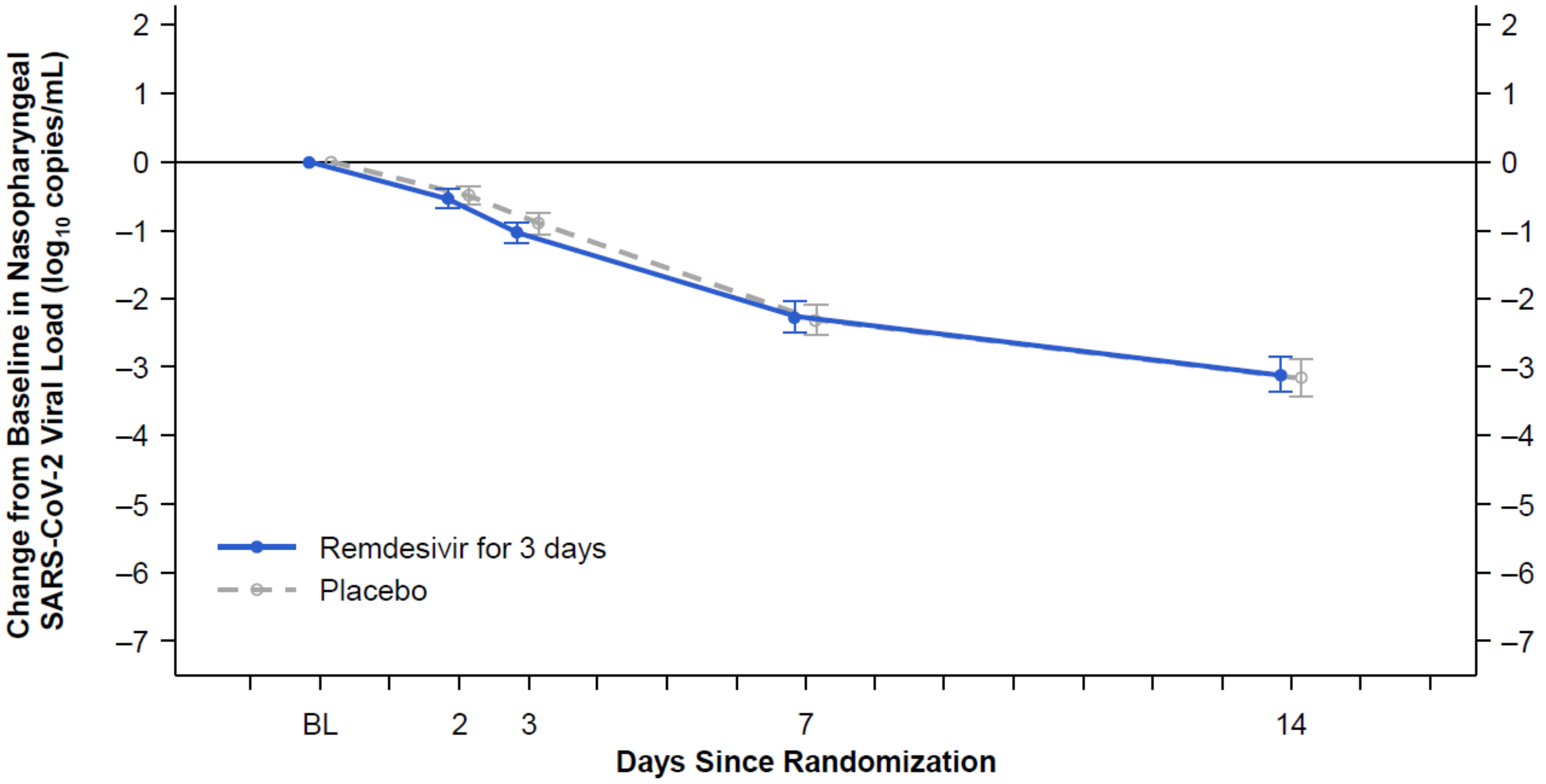

	BL	2	3	7	14
Remdesivir IV for 3 days	215	195	195	192	184
Placebo	213	192	187	190	169

Table S1. Patient demographics, hospitalization data, and outcomes over the course of available follow-up

Patient	Treatment	Age	Sex	Day of hospitalization (from time of randomization)	Reason for hospitalization	Covid-19 related Yes/no	ICU Yes/no	Outcome (transfer/discharged/death)
**Remdesivir group**								
1	Remdesivir	81	F	3	Respiratory failure	Yes	Unknown	Discharged
2	Remdesivir	40	M	2	Pneumonia	Yes	Yes	Discharged
3	Remdesivir	60	F	8	Atrial fibrillation	No	Yes	Discharged
4	Remdesivir	71	M	7	Cardiac failure congestive, Atrial fibrillation	No	Yes	Discharged
5	Remdesivir	49	M	4	Angina pectoris	No	No	Discharged
**Placebo group**								
6	Placebo	53	F	2	Covid-19 pneumonia	Yes	Yes	Discharged
7	Placebo	62	F	3	Pneumonia	Yes	No	Discharged
8	Placebo	71	M	9	Covid-19 pneumonia	Yes	Yes	Discharged
9	Placebo	50	M	3	Hypoxia	Yes	No	Discharged
10	Placebo	68	M	14	Pneumonia	Yes	No	Discharged
11	Placebo	66	M	7	Fibrin D-dimer increased	Yes	No	Discharged
12	Placebo	68	M	2	Acute respiratory failure, Covid-19 pneumonia	Yes	No	Discharged
13	Placebo	57	M	7	Covid-19 pneumonia	Yes	No	Discharged
14	Placebo	63	F	7	Covid-19 pneumonia	Yes	No	Discharged
15	Placebo	69	M	7	Covid-19	Yes	Yes	Death at study day 59

16	Placebo	74	F	2	Covid-19 pneumonia	Yes	No	Transferred
17	Placebo	60	F	1	Dyspnea	Yes	No	Discharged
18	Placebo	54	M	2	Covid-19	Yes	No	Discharged
19	Placebo	55	M	6	Covid-19 pneumonia, Respiratory failure	Yes	No	Discharged
20	Placebo	56	F	3	Hypoxia, Pneumonia	Yes	No	Discharged
21	Placebo	63	F	21	Lumbar vertebral fracture, road traffic accident	No	No	Discharged
22	Placebo	56	M	26	Angina pectoris	No	Unknown	Discharged
23	Placebo	48	F	19	Acute myocardial infarction	No	No	Discharged

Shaded rows indicate non-Covid-19-related hospitalizations.

Table S2. Representativeness of Study Participants

Category	
**Disease, problem, or condition under investigation**	Covid-19 in high-risk, non-hospitalized patients.
**Special considerations related to:**	
**Sex and gender**	Male patients have a significantly higher risk of Covid-19 disease progression.[1]
**Age**	Older adults aged 60 years or greater are at higher risk of severe illness due to Covid-19.[2,3]
**Race or ethnic group**	Covid-19 does not generally impact racial or ethnic groups differently, but due to systemic health and social inequities, some racial and ethnic minorities, such as Hispanic/Latinx and Black persons in the United States, are at increased risk for Covid-19.[2]<br><br>This trial is noteworthy for high enrollment among Hispanic/Latinx and American Indian/Alaskan Native population relative to the general population of the US.
**Geography**	Covid-19 is a global pandemic.
**Other considerations**	The risk of severe Covid-19 increases as the number of underlying medical conditions increases in a person.[3]
**Overall representativeness of this trial**	The patient population enrolled was balanced between male and female sex. About one third (30%) of patients were over the age of 60. No patients under the age of 12 were enrolled. Relative to the general United States population, of 562 patients, a higher proportion of patients with Hispanic/Latinx ethnicity (235, 44%) and American Indian or Alaskan Native race were enrolled (36, 7%), and a lower proportion of Black (42, 8%) or Asian race (13, 2%) were enrolled. Most patients enrolled (94%) were from the United States. Patients with severe renal disease and patients who were not vaccinated were excluded from enrollment. A lower proportion of patients who were immunocompromised (23, 5%) and patients with active cancer (30, 4%) were enrolled. Although the pediatric population does not generally contain high-risk patients, and vaccinated individuals have low rates of hospitalization, the lack of these patients in the study population precludes generalizability to these groups. All patient characteristics described here were self-reported by patients and collected at screening and were entered into the electronic database.

REFERENCES

1. Peckham H, de Gruijter NM, Raine C, et al. Male sex identified by global COVID-19 meta-analysis as a risk factor for death and ITU admission. Nat Commun 2020;11:6317.

2. Risk for COVID-19 Infection, Hospitalization, and Death by Race/Ethnicity. Atlanta, GA: Centers for Disease Control and Prevention, September 2021. (https://www.cdc.gov/coronavirus/2019-ncov/covid-data/investigations-discovery/hospitalization-death-by-race-ethnicity.html).

3. Assessing Risk Factors for Severe COVID-19 Illness. Atlanta, GA; Centers for Disease Control and Prevention., November 2020. (https://www.cdc.gov/coronavirus/2019-ncov/covid-data/investigations-discovery/assessing-risk-factors.html).
