# Supplementary material for: Bayesian reanalysis of early remdesivir for the treatment of COVID-19 in outpatients with high risk of progression to severe disease
Source: PLoS One. 2026 Apr 16;21(4):e0346878. doi: 10.1371/journal.pone.0346878 (PMC13086435; doi:10.1371/journal.pone.0346878)
Supplement: S6 Appendix — (PDF) [file pone.0346878.s007.pdf]

Data Sharing Statement

Question	Authors' Response
Will the data collected for your study be made available to others?	Yes
Would you like to offer context for your decision?	—
Which data?	Complete de-identified patient data set
Additional information about data	—
How or where can the data be obtained?	datarequest@gilead.com
When will data availability begin?	Six months after FDA and EMA approval of the compound studied.
When will data availability end?	N/A
Will any supporting documents be available?	—
Which supporting documents?	Other
Additional information about supporting documents	Clinical study report synopsis
How or where can supporting documents be obtained?	datarequest@gilead.com
When will supporting documents availability begin?	Six months after FDA and EMA approval of the compound studied.
When will supporting documents availability end?	N/A
To whom will data be available?	Qualified external researchers
For what type of analysis or purpose?	Requests are at Gilead's discretion and dependent on the nature of the request, the merit of the research proposed, availability of the data and the intended use of the data.
By what mechanism?	If Gilead agrees to the release of clinical data for research purposes, the requestor will be required to sign a data sharing agreement (DSA) in order to ensure protection of patient confidentiality prior to the release of any data.
Any other restrictions?	Upon execution of the DSA, Gilead will provide access to a patient-level clinical trial analysis datasets in a secured analysis environment.
Additional information	—

This statement was posted on December 22, 2021, at NEJM.org.
