# Supplementary material for: Bayesian reanalysis of early remdesivir for the treatment of COVID-19 in outpatients with high risk of progression to severe disease
Source: PLoS One. 2026 Apr 16;21(4):e0346878. doi: 10.1371/journal.pone.0346878 (PMC13086435; doi:10.1371/journal.pone.0346878)
Supplement: Attachment — Submitted filename: Abdelghany_PINETREE-trial_response to reviewers_FINAL.docx [file pone.0346878.s009.docx]

Mazin Abdelghany, MD, MS

PhD Student, Biostatistics

MRC Biostatistics Unit, East Forvie Building

Forvie Site, Robinson Way

Cambridge Biomedical Campus

Cambridge, UK CB2 0SR

Email: [mazin.abdelghany@mrc-bsu.cam.ac.uk](mailto:mazin.abdelghany@mrc-bsu.cam.ac.uk)

Tel: (617) 530-0032

11 February 2025

Dear PLOS ONE Academic Editor and reviewers,

We thank you for the time and effort taken to evaluate our paper titled “Post hoc Bayesian analysis of early remdesivir for the treatment of COVID-19 in outpatients with high risk of progression to severe disease”. We acknowledge the editors’ comment that (1) “our paper lack[s] . . . new focus compared to previously published studies” and (2) that there are “concerns on analysis methods.”

First, we emphasize that our analysis does indeed present novel research that will quickly gain traction given the recent publication of draft U.S. Food and Drug Administration guidance on the “Use of Bayesian Methodology in Clinical Trials of Drug and Biological Products.” Clinical trials applying Bayesian methods, especially in Phase 3, are rare. Fewer than 5 such publications were found in a PubMed search of articles from 2025 and only 4 clinical trials were reanalyzed with Bayesian statistical methods in 2025. Exposure to a statistical method that will gain popularity over the next decade is critical to PLOS ONE readers, and our paper is an excellent example of these methods.

Second, reviewers did not articulate specific concerns about our analysis methods in their comments. Thus, to clarify our statistical approach, we have added supplementary material that presents—in detail—our rigorous methodology and mathematical models. Further, our code has been published to a private Github repository that can be made available to PLOS ONE editors to share with reviewers interested in reviewing it for consistency. This repository will then be made public on publication.

Lastly, we greatly appreciate the insightful comments provided by reviewers and have carefully addressed each point below, making major revisions to the manuscript accordingly. With their help, we have significantly improved the rigor and clarity of the paper. Our point-by-point responses to each of their comments is in the table below, and the corresponding revisions to the original manuscript are discussed in the summary of manuscript changes section below.

The aim of this Bayesian reanalysis was to provide a more nuanced understanding of remdesivir’s treatment effect, quantify uncertainty more explicitly, incorporate historical randomized clinical trial data into the analysis, showcase its many advantages, and model best practices for future researchers.

As before, all authors have seen and approved of the updated manuscript, contributed significantly to the work, and the manuscript has not been previously published nor is it being considered for publication elsewhere. Our conflict statement is also revised: Dr. Mazin Abdelghany is a former employee of Gilead Sciences, Inc. Drs. Fang Yu, Stephen Rennard, and Yeongim Gwon have no conflicts of interest to disclose.

With appreciation,

Mazin Abdelghany, MD, MS

Table of Contents

[Summary of manuscript changes	4](#_Toc221694633)

[Point-by-point response to reviewers’ comments	6](#_Toc221694634)

## **Summary of manuscript changes**

1. The results section of the **Abstract** was revised to emphasize that (1) a minimally informative prior recovered frequentist estimates, (2) results were robust to choice of prior and (3) precision of the HR estimate was improved using data-driven priors.
2. Clarifying edits were made to the **Introduction** section. Content remains the same.
3. Clarifying edits were made to the section in the **Methods** titled Bayesian statistical methodologies to ensure that readers understand that Bayesian methods estimate a posterior distribution that can then be queried for specific probabilities of interest. A new mathematical Supplemental Appendix A was also added and referenced in the Methods section.
4. A paragraph that was originally in the Discussion section was moved into the **Results** section for clarity.
5. Another example of Bayesian methods used in the regulatory approval of a drug (REBYOTA) was added to the **Discussion**. During review and revision of the manuscript, the U.S. FDA published new draft guidance for the use of Bayesian methodology in clinical trials of drug and biological products. This new draft guidance was referenced and added to the Discussion.
6. In response to reviewer comments, our **Limitations** section now also discusses the caution needed when using multiple prior distributions and suggests a framework for approaching these types of analyses. We also added a discussion of the pitfalls of incorporating historical RCT data in Bayesian analyses.
7. Finally, a **Future research** section was added.

## **Point-by-point response to reviewers’ comments**

Reviewer #1

|  | **Comment** | **Author response** |
| --- | --- | --- |
| 1 | This is a retrospective Bayesian analysis of the PINETREE trial. What is the new focus of this reanalysis compared to the NEJM publication? | This analysis provides two new insights into the PINETREE data: (1) it allows clinicians with specific prior beliefs to understand how these data should fit into that context, and (2) incorporates the large number of prior COVID-19 trials into the PINETREE data analysis.  (1) Bayesian methods directly calculate probabilities for the hypothesis of interest. In this case, we directly calculate the probability that the hazard ratio for treatment with remdesivir (RDV) indicates benefit. This intuitive interpretation helps better contextualize the meaning of the trial results for clinicians. Also, by including a range of prior beliefs in the analysis, we allow flexibility of interpretation. Each clinician may have her/his notion of the benefit of RDV. By aligning her/his belief with the prior closest to that belief, s/he can then understand how these data should change those beliefs.  (2) Moreover, this analysis uses the wealth of data available in COVID-19 trials to inform on the analysis of PINETREE. We incorporate these data, by mechanism of drug action, to calculate the treatment effect of RDV in the wider context of other trials with the same therapeutic target and goal. |
| 2 | Why do you think the Bayesian approach is still underutilized? Could the authors explain the reasons for not applying it prospectively to the PINETREE trial and provide reasons why a post hoc analysis was used? | The Bayesian approach to clinical trial analysis remains underutilized for several reasons. First, statistical pedagogy nearly universally teaches frequentist methods of analysis. Second, and more importantly, in drug trials meant for registration of a new investigational medicinal product, the statistical analysis must be approved by regulatory authorities. Regulatory guidance documents and regulatory agencies continue to recommend frequentist analytical methods for clinical trials meant for drug registration. For this reason, the PINETREE trial was analyzed using the Cox proportional hazards method in a frequentist paradigm. This analysis method was approved by the regulatory authorities and allowed Gilead to proceed with the trial and submit label updates to regulatory authorities across the globe based on its results.  To popularize Bayesian methodology, influence regulatory guidance, and develop best practices, more clinical trials need to be analyzed (both prospectively and post hoc) with Bayesian methods and published to showcase the many advantages of such an approaches. This was one of our aims in this trial reanalysis.  Lastly, the U.S. FDA has recently published draft guidance discussing Bayesian methods of analysis for use in therapeutic drug trials (see letter above), which we believe will help these methods proliferate in industry trials in the coming years. We have added a discussion and reference to this new guidance in the Discussion section. |
| 3 | What reference and data-derived priors do you suggest in the analysis? | Tables 1 and 2 in the manuscript detail the reference and data-driven priors that were used in the analysis. A total of 5 reference priors and 3 data-driven priors were used to analyze the PINETREE data. This exhibits the flexibility of Bayesian methods.  Each prior represents a particular belief about the treatment effect of RDV. These beliefs are detailed using the probabilities calculated in Tables 1 and 2 and the rationale for selection of the reference priors is also discussed in detail in Table 1. These tables were provided to demonstrate the best practices for Bayesian analyses of clinical trials as a model for other practitioners interested in applying these methods in their research.  A paragraph was added to the discussion explaining the challenges of interpreting and communicating results when multiple prior distributions are used for an analysis. We highlight that one prior could be considered a “primary” prior while other priors could be considered sensitivity analyses. It is the authors’ opinion that the data-driven prior of DAA trials could be considered the primary prior in this analysis. |
| 4 | Can you further explain the use of data-driven priors in this paper and the combination of HRs, ORs, and RRs? | The data-driven priors were generated using the publicly available data from randomized clinical trials that were similar to PINETREE both methodologically and from a patient population standpoint. Because different trials use different statistical methodologies, in order to ensure a wide representation of clinical trials in the prior distributions, methods that calculated hazard ratios, odds ratios, and risk ratios were included in the prior distributions as these are compatible with our methods. Once the trials were selected, their information about the outcome of interest was combined using a mixture distribution. The mixtures were weighted based on the total number of participants in each trial.  These details were further clarified in the Methods section (Bayesian statistical methodology and support for prior distribution selection). We have also added a Supplementary Appendix with example mathematical calculations to clarify how mixture prior distributions were generated. |
| 5 | What estimations do you have for the Bayesian model that estimates the number of events that did not occur and the average events after a particular time in relation to the hypothesized number of events in relation to the obtained number of censored observations? | We thank the reviewer for this insight. Censored observations were handled using the prototypical Cox proportional hazards model. We have added a Supplementary Appendix with a discussion of the model used and its mathematical formalism and referenced this in the Methods section.  Causal inference methods can be used to estimate counterfactual scenarios in which events that happened during the trial are compared with a situation in which they did not happen. As we were performing a simple Bayesian reanalysis of the PINETREE study, we did not perform any causal inference analyses. In randomized clinical trials, the assumption is made that the control group is the facsimile of the counterfactual. This question could be the topic of future research. |
| 6 | It is possible to ignore a huge number of sample criteria that is related to the confidence limits of the events that happen in geometric transformations and the time periods of the geometric intervals. | We thank the reviewer for bringing this to our attention. The prior distribution that was utilized for these analyses was a normal distribution on the logarithm transformed hazard ratio. This allows for negative values of the normal distribution to be exponentiated to values restricted to the positive real numbers, which is consistent with hazard ratio values. We did not perform any geometric transformations. |
| 7 | Was consideration given to the quality of the trials and assessments of risk of bias? | The quality of the trials and the risk of bias for including the trials were considered (see Methods – Selection of RCTs for data-driven priors). ClinicalTrials.gov was used to search for relevant trials with the following criteria:   (1) Adult interventional (2) Older adult interventional (3) Phase 3 (4) Start: 1 November 2019 (5) Stop: 31 December 2021   273 trials matched the search criteria. The following criteria were used to exclude trials:   (1) Recruited less than 300 participants (2) Prevention trials (3) Non-systemic treatments (4) No published results (5) Incompatible outcome measure (i.e., did not include hospitalization and/or death as an outcome or within a composite outcome) (6) Statistical method not suitable for incorporation (i.e., calculated something other than a hazard ratio, risk ratio, or odds ratio)   These criteria were selected to ensure that the studies were as close to PINETREE as possible. However, as discussed in the limitations section, there will always be a risk of bias when selecting trials for inclusion (similar to risks that occur in meta-analyses). Formal assessment of risk of bias was out of the scope of this manuscript. |
| 8 | The authors report probabilities for HR < 0.2, HR < 0.1, etc. Why are these specific cut-offs considered clinically appropriate thresholds? | Our aim was to estimate the posterior probability distribution of the HR under a range of both reference and data-driven priors, affording us the flexibility to calculate probabilities of a minimally clinically important treatment effect. Posterior probabilities were calculated for HR < 1, HR < 0.9, HR < 0.8, . . . , HR < 0.2, HR < 0.1. Selection of HR < 1 was made because of the frequentist convention that the null hypothesis is usually that of no treatment effect (HR = 1). Thus, the probability that HR < 1 is a natural first choice.  The other cutoffs were chosen based on the results of prior clinical trials. The clinical trials that were selected for the data-driven priors calculated point estimates that ranged from 0.14 to 1.11. These hazard ratios were all considered plausible when planning table 3 and therefore HRs from 0.1 to 1 at equally spaced intervals of 0.1 were selected.  Clarifications were made to the Methods section titled “Bayesian statistical methodology and support for prior distribution selection”.  We are also happy to include other cutoffs that the reviewer deems as clinically appropriate thresholds. |
| 9 | Table 3 has posterior probabilities, yet the manuscript doesn’t always clarify the ways to interpret these probabilities in clinical matters. Could the authors illustrate how these probabilities can be practically applicable to clinicians? | We gave two examples of how a clinician may utilize the posterior probabilities in Table 3 in the Discussion section. Based on this comment, we have made some clarifying edits to this section of the Discussion.  In more detail, in order for a clinician to use these probabilities in Table 3, the following steps must be taken:   (1) Select the prior belief that is closest to the clinician’s belief   This can be done, roughly, by reviewing Tables 1 and 2. For example, a clinician may be skeptical of remdesivir’s effect on hospitalization or death. She may believe that there is a one third (33%) chance that remdesivir increases the risk of hospitalization by 25% (corresponding to a hazard ratio of 1.25 or more). This belief is similar to the moderately skeptical prior.   (2) Read the row in Table 3 that corresponds to this prior.   For example, whereas the clinician may have believed that there is a 33% chance of harm from RDV administration, after incorporating the information from the PINETREE trial into this belief, she can now say that there is a 1.1% chance of any harm (HR > 1). This is calculated by noting that the posterior probability of the hazard ratio being < 1 in the moderately skeptical row is 0.989. Therefore, the probability of the HR being >1 is 1 – 0.989 = 0.011. |
| 10 | The results seem to concentrate on the hospitalization/death outcome within 28 days. Were the secondary endpoints of PINETREE (symptom resolution, viral load, etc.) incorporated into the Bayesian analysis? | There were several secondary endpoints in PINETREE including the composite of Covid-19–related medically attended visits or death from any cause by day 28 and the time to alleviation of baseline Covid-19 symptoms based on FLU-PRO Plus questionnaire. Given the scope of the manuscript and the word count limitations, we chose to focus on the primary efficacy endpoint. This may be considered as an area of future research and a section to this effect was added to the discussion. |
| 11 | In the Discussion, it is said that Bayesian analysis could enable smaller sample sizes in future studies. Why do the authors think their findings support this? | Bayesian analyses allow for the incorporation of historical data. When information from prior RCTs is incorporate in an analysis, the Bayesian credible interval is narrower than the frequentist confidence interval in cases of the mixture of all 9 RCTs and the mixture of the direct-acting antivirals. The narrower credible interval indicates that there is higher confidence in the estimate of the hazard ratio when historical information is included (Discussion lines 232 – 237 from unrevised manuscript).  In trial design situations, prospective specification of priors utilizing historical data “allow to reduce the number of subjects. This decreases costs and trial duration, facilitates recruitment, and may be more ethical.” (reference 24 of the manuscript and also <https://doi.org/10.1080/19466315.2024.2342817>). |
| 12 | In the case of this analysis being an analysis of the data after the primary analysis, what attempts were made to reduce the chance of a Type I error, taking into account the multiple tests that were conducted? | We acknowledge that this is an important discussion and one that requires careful attention. Given the scope of this manuscript, we do not include discussion of type I error rate control in Bayesian contexts.  In Bayesian analyses, strict type I error rate control is not usually possible (<https://doi.org/10.1080/19466315.2024.2342817>). There have been descriptions of Bayesian designs with informative priors that evaluate the classical (frequentist or conditional) type I error rate. If defined in the traditional way (considering only the sampling distribution of the observed data), however, it cannot be strictly controlled (DOI: 10.1093/biostatistics/kxy009 and DOI: 10.1002/bimj.201800395).  It is important to note that multiple testing is not a concern when querying a posterior probability distribution. The posterior probability distribution is calculated once (based on the prior distribution and the likelihood) and then posterior probabilities can be calculated (such as the probability that the HR < 1). In this case, calculating another posterior probability does not change the distribution itself. Thus, the frequentist concern for multiple hypothesis testing is not relevant. A brief explanation of these concepts was added to the manuscript. |
| 13 | In the Discussion, the authors refer to the reduction of the sample size in Bayesian trials and the regulatory history regarding the study (e.g. the approval of belimumab). Are there other case studies, proprietary or publicly available regulatory documents that authors could use to enhance this section? | There are indeed other examples of regulatory submissions that took advantage of Bayesian designs in their regulatory submission process. An oft-cited systematic review is doi: 10.1136/bmjopen-2018-024895. In the discussion section, given the space limitations of the manuscript, we chose to focus on an approval that included a randomized controlled trial as this was felt to be most relevant to the analysis presented.  We would also like to bring attention to the new FDA guidance on utilization of Bayesian methods in clinical trials. Section III reviews situations where Bayesian methods have been used in prior submissions for drug approval (<https://www.fda.gov/media/190505/download>). We have added other representative examples from this document to the discussion section of our manuscript. |
| 14 | Some RCT studies, which form a part of the data-driven priors, are provided only in the supplement. Could the authors provide the relevant in-text citations within the Methods or Results section? | Only 3 of the 9 RCTs were published in the literature with results of the other 6 posted on ClinicalTrials.gov. We elected to include their trial identifiers rather than links to publications to ensure that data was presented systemically and uniformly for all trial data included.  As suggested, we have added the those references that are available for the 3 published trials in the Methods section as well. |

Reviewer #2

|  | **Comment** | **Author response** |
| --- | --- | --- |
| 1 | I found the results are sensitive to how prior distributions are chosen as overly optimistic priors could bias results.  Please comment | It is a feature of Bayesian analyses that the prior distribution affects the posterior distribution. Because of this explicit link between the two, we believe (along with others in the field) that it is crucial to (1) describe the prior distributions used in the analysis, (2) justify their selection, and (3) report on the use of multiple prior distributions. These three strategies help to mitigate the concern that results can be sensitive to the selection of the prior distribution.  As long as researchers are transparent about prior choice and methodology, these choices are made explicit. Moreover, if others in the field disagree with a certain prior distribution selection, Bayesian analysis allows for easy substitution of one prior for another for comparison. This can go as far as allowing regulators to select their own priors when analyzing the data for a pivotal clinical trial to perform sensitivity analyses. |
| 2 | The PINETREE population was unvaccinated and infected with early SARS-CoV-2 variants, so I believe results may not apply to later contexts. How would you justify this? | The COVID-19 pandemic has been rapidly evolving and the PINETREE population was unvaccinated and infected with early SARS-CoV-2 variants. The results of this trial may not apply to patients that are infected with SARS-CoV-2 now.  The aim of this Bayesian reanalysis was to provide a more nuanced understanding of remdesivir’s treatment effect, quantify uncertainty more explicitly, incorporate historical RCT data into the analysis and showcase its many advantages, and model best practices for future analyses. While it is possible that the effects of remdesivir have changed over time given changes in the population, we were unable to address this question as it was beyond the scope of this manuscript. |
| 3 | Why No new data? can we combine the old data with some new one for future work? | There have not been further assessments of remdesivir’s efficacy for the treatment of COVID-19 because, as COVID-19 became a disease affecting outpatients, RDV’s intravenous administration route became more logistically challenging. Development efforts across the industry therefore shifted away from intravenous medications and towards medications administered orally (e.g., nirmatrelvir-ritonavir).  Because there are no more available data, no new analyses can be performed. |
| 4 | How Posterior probability distributions were used to calculate the probability of HR? Can you explain the used Posterior,  Likelihood and Prior? Also, can you attach the derivations if you have done any? | Bayes theorem was used to calculate the posterior distributions using the prior and the likelihood. Briefly,  $P\left( \theta\vert D \right)=\frac{P\left( D \vert\theta\right)\cdot P(\theta)}{P(D)}$  where P(θ \| D) is the posterior probability, P(D \| θ) is the likelihood (calculated using the proportional hazard introduced by Breslow), P(θ) is the prior probability (the belief), and P(D) is a normalizing constant that is used to ensure that P(θ \| D) (the posterior probability distribution) sums to 1.  The prior distribution was a normal distribution on the logarithm of the hazard ratio (as this allows for negative values of the normal distribution to be exponentiated to values restricted to the positive real numbers consistent with hazard ratios).  A Supplementary Appendix was added laying out the mathematical formalism for the prior, likelihood, and posterior probability calculations. |
