# Supplementary material for: Bayesian reanalysis of early remdesivir for the treatment of COVID-19 in outpatients with high risk of progression to severe disease
Source: PLoS One. 2026 Apr 16;21(4):e0346878. doi: 10.1371/journal.pone.0346878 (PMC13086435; doi:10.1371/journal.pone.0346878)
Supplement: Attachment — Submitted filename: Response to Reviewers.docx [file pone.0346878.s010.docx]

**Point-by-point response to editor and reviewers’ comments**

Academic Editor

|  | **Comment** | **Author response** |
| --- | --- | --- |
| 1 | Please ensure that your manuscript meets PLOS ONE's style requirements, including those for file naming. | We have updated the manuscript formatting to adhere to PLOS ONE’s style and file naming requirements. |
| 2 | If there are restrictions on sharing of data and/or materials, please state these. Please note that we cannot proceed with consideration of your article until this information has been declared.  Please include your updated Competing Interests statement in your cover letter; we will change the online submission form on your behalf. | We have noted the restrictions on sharing of data in the cover letter. It is reproduced here for convenience:  Data for the PINETREE trial are owned by Gilead Sciences, Inc.  Data collected for the study can be made available to others as a complete de-identified patient dataset by contacting [datarequest@gilead.com](mailto:datarequest@gilead.com). Requests are at Gilead’s discretion and dependent on the nature of the request, the merit of the research proposed, availability of the data and the intended use of the data. If Gilead agrees to the release of clinical data for research purposes, the requestor will be required to sign a data sharing agreement (DSA) in order to ensure protection of patient confidentiality prior to the release of any data. Upon execution of the DSA, Gilead will provide access to a patient‐level clinical trial analysis datasets in a secured analysis environment.  Please see the cover letter for the updated Competing Interests statement. It is reproduced here for convenience:  I have read the journal's policy, and the authors of this manuscript have the following competing interests: I am a former employee of Gilead Sciences, Inc. Drs. Yeongin Gwon, Stephen Rennard, and Fang Yu have no conflicts of interest to declare. |
| 3 | Please clarify whether this [conference proceeding or publication] was peer-reviewed and formally published. If this work was previously peer-reviewed and published, in the cover letter please provide the reason that this work does not constitute dual publication and should be included in the current manuscript. | As previously stated in my upload, Figure 1, the CONSORT diagram for the PINETREE clinical trial, was previously published in the Supplement of the original NEJM article reporting on this clinical trial (<https://www.nejm.org/doi/10.1056/NEJMoa2116846>). NEJM is a peer-reviewed, formally published journal.  As part of all clinical trial reporting, inclusion of a CONSORT diagram is considered best practice. It was reproduced as is for the readers’ convenience. If the editor deems it necessary, either (1) the figure can be removed from the submission, (2) permission to reproduce the figure can be obtained from the New England Journal of Medicine, or (3) a new, original figure can be generated that describes patient disposition in less detail. We are happy to comply with any of the above suggest resolutions. |
| 4 | We note that you have indicated that there are restrictions to data sharing for this study. PLOS only allows data to be available upon request if there are legal or ethical restrictions on sharing data publicly. | There are legal restrictions on sharing a de-identified dataset. There was a data sharing statement published alongside the original manuscript that can be found at <https://www.nejm.org/doi/10.1056/NEJMoa2116846> at the bottom of the page.  Data for the PINETREE trial are owned by Gilead Sciences, Inc. Data collected for the study can be made available to others as a complete de-identified patient dataset by contacting [datarequest@gilead.com](mailto:datarequest@gilead.com). Requests are at Gilead’s discretion and dependent on the nature of the request, the merit of the research proposed, availability of the data and the intended use of the data. If Gilead agrees to the release of clinical data for research purposes, the requestor will be required to sign a data sharing agreement (DSA) in order to ensure protection of patient confidentiality prior to the release of any data. Upon execution of the DSA, Gilead will provide access to a patient‐level clinical trial analysis datasets in a secured analysis environment. |
| 5 | Please include captions for your Supporting Information files at the end of your manuscript, and update any in-text citations to match accordingly. | We have included captions for the Supporting information files and updated the in-text citations accordingly. |
| 6 | To ensure your figures meet our technical requirements, please review our figure guidelines: <https://journals.plos.org/plosone/s/figures> | We have reviewed the figure requirements and our figures meet the technical requirements as assessed by the NAAS tool provided at <https://journals.plos.org/plosone/s/figures>. |

Reviewer #1

|  | **Comment** | **Author response** |
| --- | --- | --- |
| 1 | The authors have carefully addressed all comments and queries raised during the review process. I have no further comments or concerns regarding dual publication, research ethics, or publication ethics. The manuscript is now suitable for publication in its current form. | We thank Reviewer #1 for their time and effort in reviewing or manuscript. It has been significantly improved as a result of their criticism. |

Reviewer #3

|  | **Comment** | **Author response** |
| --- | --- | --- |
| 1 | The manuscript presents a Bayesian statistical framework implemented through the Pinetree approach to evaluate treatment effects using large clinical datasets. Although Remdesivir is used as the illustrative case study, the primary contribution of the work lies in demonstrating the methodological framework and its potential application for incorporating prior information and clinically relevant patient-level parameters in statistical inference.  The authors selected studies with sufficiently large sample sizes and appropriate control groups, which strengthens the robustness of the dataset used for the Bayesian analysis. In this context, the manuscript appears to focus primarily on methodological development rather than on drawing definitive clinical conclusions regarding the efficacy of Remdesivir.  After carefully reviewing the revised version of the manuscript and the responses provided to previous reviewers, I believe that the authors have already addressed the main methodological and structural concerns raised during the review process. The manuscript is now clearer regarding its objective of presenting a statistical framework rather than a clinical efficacy analysis.  At this stage, I do not have additional substantive comments to add. The manuscript appears suitable for publication in its current form from a methodological perspective. | We thank Reviewer #2 for their time and effort in reviewing or manuscript. It has been significantly improved as a result of their criticism. |

Reviewer #4

|  | **Comment** | **Author response** |
| --- | --- | --- |
| 1 | The authors present a Bayesian analysis of early remdesivir for the treatment of COVID-19 and have used data-driven priors to reach the results. The Bayesian approach is regarded as a modern technique in the sense that, unlike the classical approach, it incorporates expert opinion in the analysis. So it's suggested the posterior analysis be included with an informative prior eliciting the prior parameters (hyperparameters) to highlight the impact of prior information. It is also suggested the classical results may also be included for comparing the two techniques for the readers at large. | We thank Reviewer #2 for their time and effort in reviewing or manuscript.  We note that the posterior probability distributions were presented in Table 3 and Figure 4 of the manuscript. Moreover, the original results of the trial are discussed in our Results section under the subsection Original PINETREE trial results. |
